# Development of tethered dual catalysts: synergy between photo- and transition metal catalysts for enhanced catalysis†
†Electronic supplementary information (ESI) available: Synthetic procedures; characterisation data; crystallographic data; NMR spectra; details of the photophysical, singlet oxygen quantum yield, cyclic voltammetry and XAS measurements; catalytic procedures and control experiments. CCDC 1955141–1955144. For ESI and crystallographic data in CIF or other electronic format see DOI: 10.1039/d0sc02703k


**DOI:** 10.1039/d0sc02703k

**Published:** 2020-06-05

**Authors:** Danfeng Wang, Robert Malmberg, Indrek Pernik, Shyamal K. K. Prasad, Max Roemer, Koushik Venkatesan, Timothy W. Schmidt, Sinead T. Keaveney, Barbara A. Messerle

**Affiliations:** a Department of Molecular Sciences , Macquarie University , North Ryde , NSW 2109 , Australia . Email: barbara.messerle@sydney.edu.au ; Email: sinead.keaveney@mq.edu.au; b ARC Centre of Excellence in Exciton Science , School of Chemistry , University of New South Wales , Kensington , NSW 2052 , Australia

## Abstract

While dual photocatalysis–transition metal catalysis strategies are extensively reported, the majority of systems feature two separate catalysts, limiting the potential for synergistic interactions between the catalytic centres. In this work we synthesised a series of tethered dual catalysts allowing us to investigate this underexplored area of dual catalysis. In particular, Ir(i) or Ir(iii) complexes were tethered to a BODIPY photocatalyst through different tethering modes. Extensive characterisation, including transient absorption spectroscopy, cyclic voltammetry and X-ray absorption spectroscopy, suggest that there are synergistic interactions between the catalysts. The tethered dual catalysts were more effective at promoting photocatalytic oxidation and Ir-catalysed dihydroalkoxylation, relative to the un-tethered species, highlighting that increases in both photocatalysis and Ir catalysis can be achieved. The potential of these catalysts was further demonstrated through novel sequential reactivity, and through switchable reactivity that is controlled by external stimuli (heat or light).

## Abstract 2

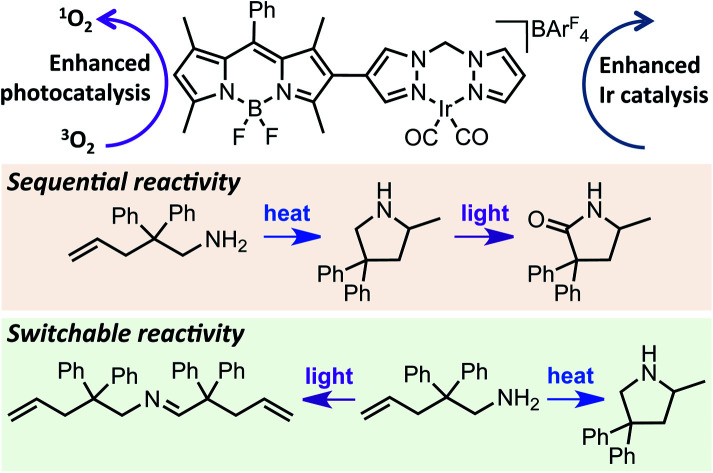
A series of tethered dual catalysts were developed, with catalytic investigations demonstrating that tethering enhances photocatalysis and thermally activated Ir catalysis. In addition, sequential and switchable catalytic reactivity was achieved.

## Introduction

Catalysts are essential tools in modern synthetic chemistry, with 90% of industrial chemicals synthesised through a sequence that involves at least one catalytic step.^1^,^2^ This extensive use of catalysts arises from their ability to make synthetic processes more efficient, reducing energy consumption and waste generation, as well as their ability to promote otherwise unachievable transformations. Despite their widespread use, there is a continuing need to advance previous methodologies, with targeted catalyst design allowing more efficient and practical chemical transformations to be realised.

While the majority of well-established catalytic processes utilise a single catalyst to facilitate the desired chemical transformation, recently there has been a surge in interest in merging different types of catalysis to permit new chemical reactivity.^3^–^6^ In particular, there is a growing interest in developing dual-catalytic systems, where cooperation between different catalysts can increase reaction efficiency, or promote reactivity that is not possible using a single catalyst. Significant advances in this emergent field have included the development of a variety of dual-catalytic systems, such as combining: (1) metal catalysis and organocatalysis;^5^,^7^,^8^ (2) photocatalysis and organocatalysis;^9^–^14^ and (3) photocatalysis and metal catalysis.^15^–^26^ While dual catalysis has emerged as an excellent synthetic platform for discovering new reactivity, most reported dual catalytic systems feature individual catalysts added as independent species to the reaction mixture, with comparatively little focus on single compounds that feature two distinct catalytic sites.^27^–^33^ Recent examples of these ‘bifunctional’ catalysts include a photo-palladium catalyst for Sonogashira cross coupling^27^ and a chiral copper catalyst for enantioselective imine alkylation.^30^

**Fig. 1:**
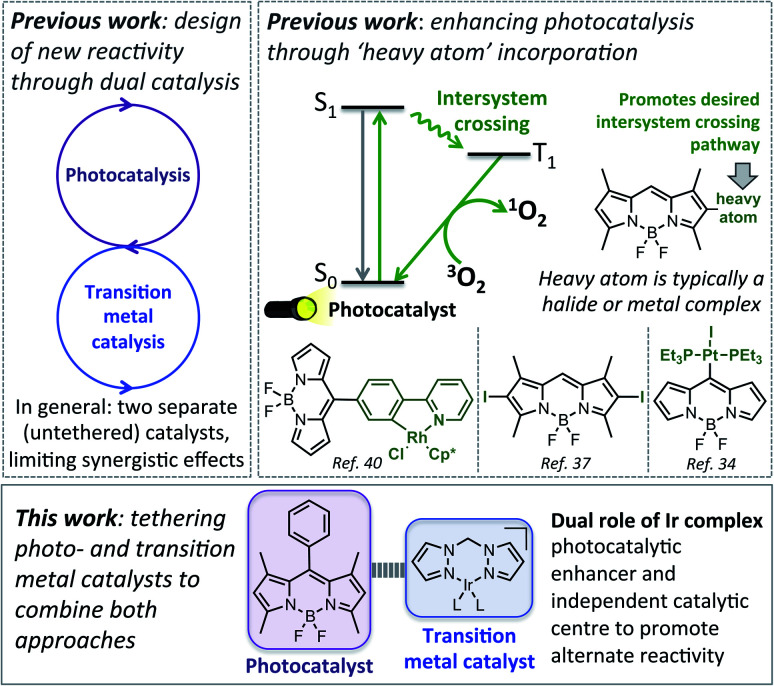
Top: Previous approaches to designing new reactivity using dual catalysis, and more efficient photocatalysts. Bottom: The aim of this work.

Chemically tethering different catalysts could permit unique synergy between the catalytic centres, with this approach anticipated to be particularly advantageous for photocatalysis, as ‘heavy atom’ (*e.g.* halide or metal) incorporation can enhance photocatalytic activity.^34^–^39^ In particular, tethering metal complexes to the widely used 4,4-difluoro-4-bora-3*a*,4*a*-diaza-*s*-indacene (BODIPY) type dyes can promote intersystem crossing (ISC) from the singlet to the triplet excited state, leading to generation of reactive singlet oxygen which is often key to photocatalysis (Fig. 1).^40^,^41^ While tethered BODIPY–metal complex species have been applied as photocatalysts,^42^ therapeutics,^43^–^46^ gas sensing^47^–^51^ and as mechanistic probes,^48^,^52^–^54^ their use in dual catalysis is limited.^27^ As such, if a catalytically active ‘heavy atom’ unit was tethered to BODIPY, this species could have the dual role of enhancing photocatalysis, as well as providing an independent catalytic site to promote alternative reactivity.

**Fig. 2:**
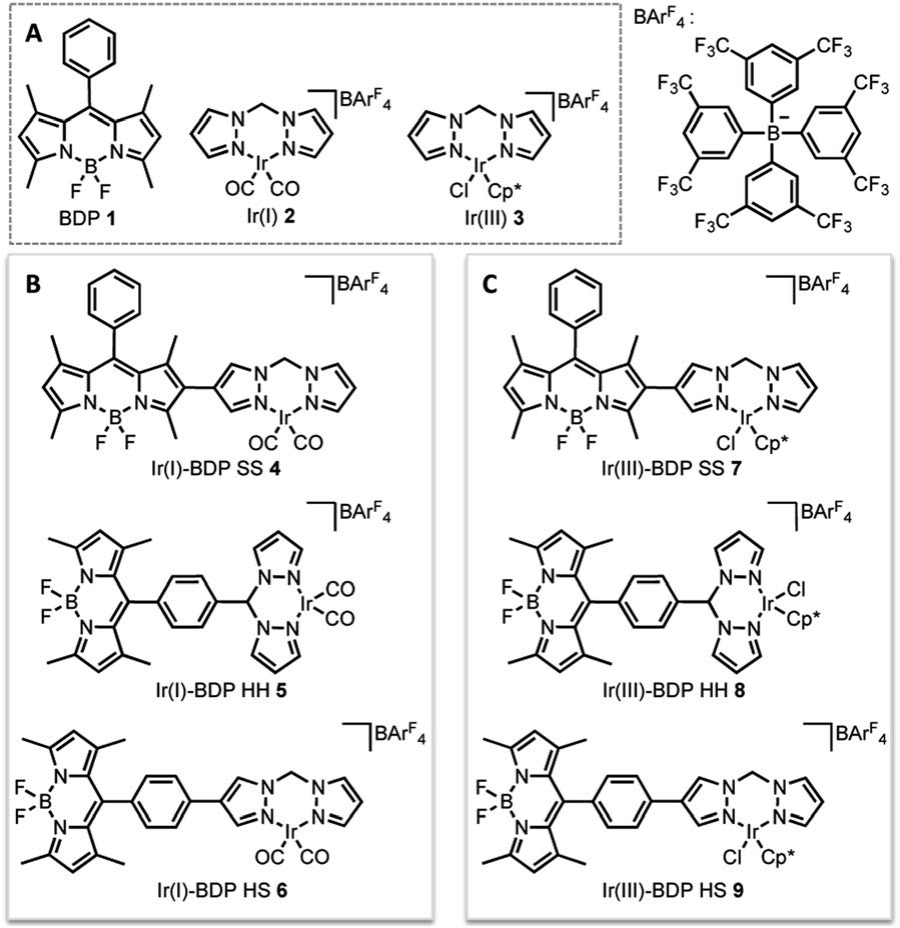
The parent catalysts on which the bifunctional catalysts are based (A); the bifunctional catalysts featuring an Ir(i) (B) or an Ir(iii) catalyst (C).

In this current work we explored this emergent area of dual catalysis using bifunctional catalysts that feature a photocatalyst tethered to a thermally activated transition metal catalyst. In particular, the BODIPY-type photocatalyst, BDP **1** (Fig. 2) was chosen due to its excellent stability, strong ground-state absorption and ease of modification.^35^,^39^,^41^ The iridium bis(pyrazole)methane based complexes Ir(i) **2** and Ir(iii) **3** were chosen due to their high stability, including air tolerance, ease of synthesis and their ability to promote diverse reactivity, including hydroamination,^55^,^56^ dihydroalkoxylation^57^,^58^ and hydrosilylation.^56^,^59^ These properties make Ir(i) **2** and Ir(iii) **3** ideal candidates as the ‘heavy atom surrogate’ attached to BDP **1**, where it will act as both a photocatalytic enhancer and an independent catalytic site. As there are many different ways to tether the catalysts, three different linking modes were targeted to gain insight into how the tethering mode affects catalytic cooperatively (Fig. 2).

Herein we report a series of tethered dual catalysts, together with a thorough analysis of the structural and physical properties of the new catalysts, providing particular insight into the photophysical properties and catalytic outcomes. This work clearly demonstrates for the first time that chemically tethering heat and light activated catalysts together can allow efficient dual catalytic strategies to be developed, marking a substantial advancement in dual catalyst design.

## Results and discussion

### Design and synthesis of the bifunctional catalysts

**Scheme 1:**
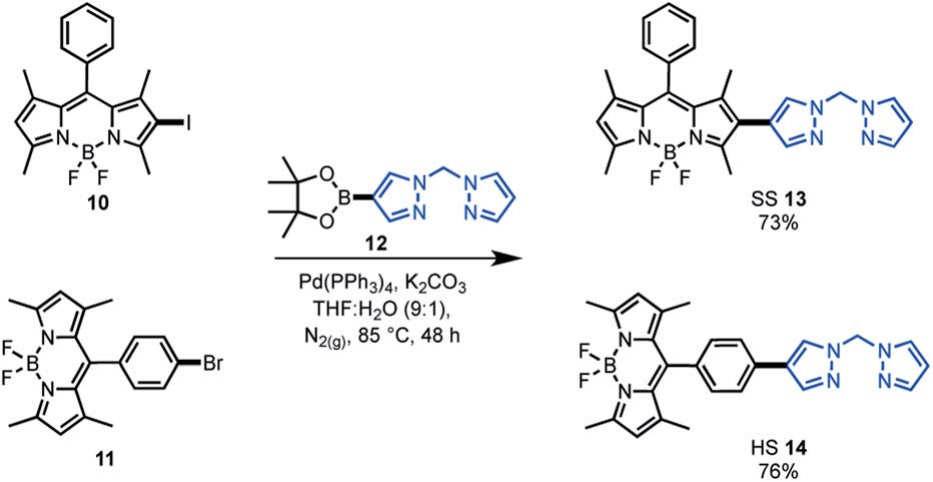
Synthesis of the bifunctional catalyst frameworks SS **13** and HS **14** through Suzuki cross-coupling reactions.

As the target was to assess bifunctional catalysts featuring different tethering modes, a modular synthetic approach was desirable. In addition, an approach that is tolerant to a wide range of functional groups could permit extension to other ligand frameworks and photocatalysts in the future. As such, Suzuki cross-coupling was utilised as the key step in constructing our bifunctional catalysts. This approach is particularly useful as there are numerous synthetic reports of halogenated BDP **1** derivatives.^36^–^38^,^60^ As the BDP motif will feature a halogen, the boronic ester substituted bis(pyrazole)methane **12** was synthesised over two steps from bis(pyrazole)methane. With these building blocks in hand, Suzuki cross-coupling reactions were performed, generating two bifunctional catalyst frameworks in good yield (Scheme 1). These species have different tethering modes between BDP **1** and the ligand that will support iridium, with the connecting modes termed as side–side (SS **13**) and head–side (HS **14**).

**Scheme 2:**
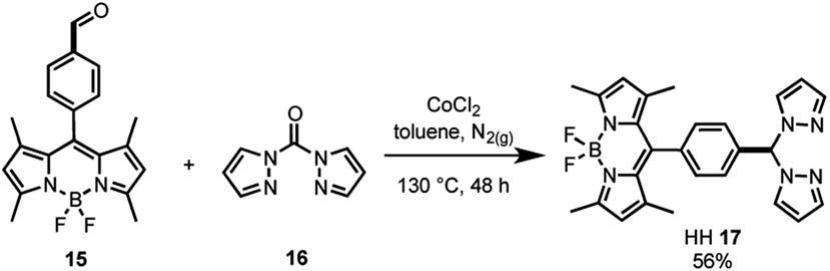
Synthesis of the bifunctional catalyst framework HH **17**.

As the synthesis of bis(pyrazole)methane **16** is well-known,^61^ the bifunctional catalyst framework for the head–head (HH **17**) tethering mode was synthesised through a cobalt catalysed condensation reaction between compounds **15** and **16** (Scheme 2). The HH framework **17** was of particular interest due to the sp^3^ hybridised CH moiety that disrupts conjugation between the BDP and Ir centre, unlike the fully conjugated SS **13** and HS **14** frameworks.

**Scheme 3:**
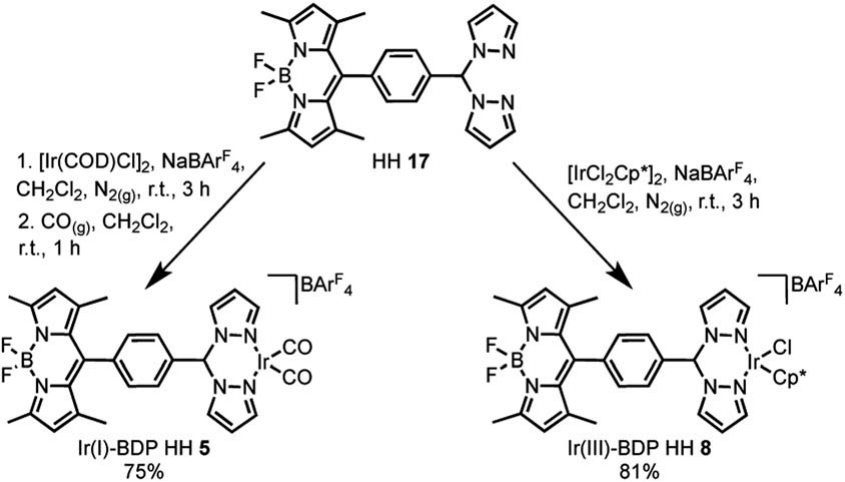
The coordination of Ir(i) or Ir(iii) to the bifunctional ligand, with the synthesis of Ir(i)–BDP HH **5** and Ir(iii)–BDP HH **8** shown as representative examples.

The final step of the synthetic strategy was coordination of the Ir(i) or Ir(iii) species to the bis(pyrazole)methane moiety in compounds **13**, **14** and **17**, and formation of the cationic iridium complex through addition of sodium tetrakis[3,5-bis(trifluoromethyl)phenyl]borate (NaBArF4). The same methods were used for forming the Ir(i) and Ir(iii) derivatives for each framework, with representative reactions with **17** shown in Scheme 3. Overall, these synthetic strategies allowed access to all six of the bifunctional catalysts **4–9**.

### Structural characterisation

**Fig. 3:**
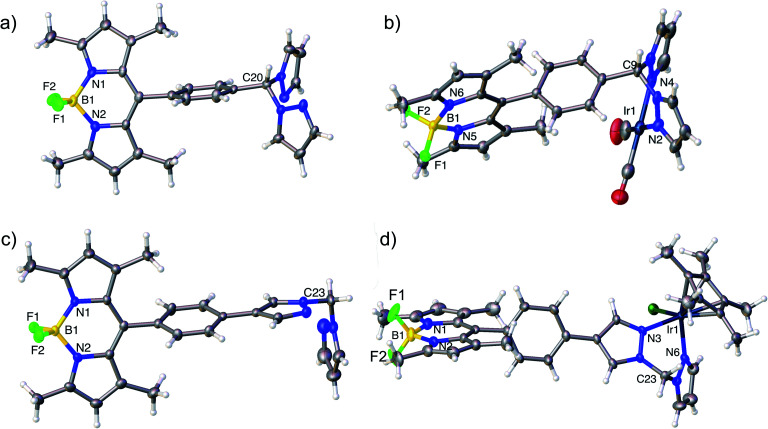
Molecular structures derived from X-ray single crystal diffraction: (a) HH ligand **17** (CCDC: ; 1955143); (b) Ir(i)–BDP HH **5** (CCDC: ; 1955144), (c) HS ligand **14** (CCDC: ; 1955141); (d) an analogue of Ir(iii)–BDP HS **9** containing a BPh_4_ counterion (see ESI† for details, CCDC: ; 1955142). Thermal ellipsoids are shown at the 50% probability level. BArF4 and BPh_4_ counterions have been omitted for clarity.

Nuclear Magnetic Resonance (NMR) spectroscopy, high resolution mass spectrometry and elemental analysis confirmed the formation of complexes **4–9**. Crystals suitable for X-ray crystallography were obtained for two ligands (**14** and **17**) and two complexes (**5** and a derivative of **9** featuring a BPh_4_ counterion) (Fig. 3). The structural data, in combination with the NMR spectral data, confirmed the structures and tethering modes of these novel bifunctional catalysts, and indicated that tethering BDP **1** to Ir(i) **2** or Ir(iii) **3** led to no significant structural changes in the BDP or iridium units regardless of the level of conjugation between the iridium and boron centre (Tables S1 and S2†).^62^–^64^ This is important as it indicates that the photophysical or electrochemical properties of **4–9**, or changes in catalytic activity relative to the two catalyst components, are not simply due to structural differences that occur upon tethering.

Infrared spectroscopy allowed evaluation of the electronic environment of Ir(i) in the carbonyl ligated species **4–6**, relative to the mononuclear complex Ir(i) **2**. When moving from **2** to the bifunctional species **4–6** there was a slight decrease in the carbonyl stretching frequencies {**2** (2100, 2035)^65^ > **4** (2098, 2035) > **5** (2093, 2029) > **6** (2091, 2028 cm^–1^)}. These data are indicative of increased electron donation from the ligand to Ir in the bifunctional catalysts. While this effect was minor, it suggests that tethering Ir(i) **2** and BDP **1** results in increased electron donation to Ir, likely due to electron transfer from the BDP moiety.

### Photophysical properties

To understand how the Ir centre influences the properties of BDP **1**, detailed photophysical investigations were performed (Table 1). Firstly, analysis of the parent iridium catalysts **2** and **3** showed that these species have very low extinction coefficients (*ε*) in the visible region (350–650 nm) and do not emit light. This is important as it highlights that any differences in the photophysical behaviour of BDP **1** and the bifunctional catalysts **4–9** are not simply due to the Ir moiety acting as a photocatalyst itself.

**Table 1:** Photophysical and electrochemical properties of catalysts **1–9** and the ligand frameworks **13**, **14** and **17**

Complex	*λ*_abs_/nm (*ε*/×10^5^ M^–1^ cm^–1^)^*a*^	*λ*_em_ ^*a*^/nm	*τ*_F_ ^*a*^/ns	*Φ*_F_ ^*a*^ ^,^ ^*b*^/%	*k*_r_ ^*c*^/10^8^ s^–1^	*k*_nr_ ^*c*^/10^8^ s^–1^	*τ*_S_ ^*d*^/ns	*τ*_T_ ^*d*^/μs	*τ* air T ^*e*^/μs	*Φ*_ISC_ ^*d*^/%	*Φ* air ISC ^*e*^/%	*E*_1/2_ ^*f*^ ^,^ ^*g*^/V		*E* ^red^ ^*f*^ ^,^ ^*h*^/V
												ox	red	
BDP **1**	504 (1.00)	513	3.26	99	3.04	0.03	3.8	—	—	<2	<2	0.70	–1.72	–1.43^*i*^
Ir(i) **2**^*j*^	370 (0.03)													–1.65,–1.28
BDP **1** + Ir(i) **2**	503 (0.91)	514	3.29	67	2.04	1.00	3.6	—	—	<2	<2			
Ir(i)–BDP SS **4**	507 (0.76)	526	3.58	63	1.76	1.03	3.8	>500	1.6	7.0	10.4	0.88	–1.69	–1.55
Ir(i)–BDP HH **5**	509 (0.76)	519	3.03	23	0.76	2.54	3.6	—	—	—		0.81	–1.67	–1.61^*i*^
Ir(i)–BDP HS **6**	506 (0.21)	516	2.57	48	1.87	2.02	2.1	160	1.0	4.3	7.3	0.75	–1.72	–1.58
Ir(iii) **3**^*j*^	^*k*^													–1.55
BDP **1** + Ir(iii) **3**	503 (0.91)	514	3.26	65	1.99	1.07	3.7	—	—	<2	<2			
Ir(iii)–BDP SS **7**	510 (0.83)	533	2.70	49	1.81	1.89	2.8	>500	1.1	7.2	7.3	0.69	–1.69	–1.15
Ir(iii)–BDP HH **8**	509 (0.81)	524	1.80	61	3.39	2.17	1.9	—	—	—	—	0.84		–1.91, –1.62
Ir(iii)–BDP HS **9**	506 (0.47)	517	2.45	47	1.92	2.16	2.8	—	—	—	—	0.74		–1.71
SS **13**	519 (0.61)	565	4.93	79	1.60	0.43						0.65	–1.68	
HH **17**	505 (0.85)	515	2.69	64	2.38	1.34						0.75	–1.66	
HS **14**	504 (0.83)	514	2.73	81	2.97	0.70						0.71	–1.70	

^*a*^Measured in toluene (1 × 10^–5^ mol L^–1^) at 298 K. Uncertainty for *λ*_abs_ and *λ*_em_: ±1 nm. Uncertainty for *τ*_F_: ±0.3 ns.

^*b*^Absolute quantum yield measured with an integrated sphere, uncertainty for *Φ*_F_: ±5%.

^*c*^Rates constants of radiative (*k*_r_) and non-radiative (*k*_nr_) decay calculated using the formula *k*_r_ = *Φ*_F_/*τ*_F_ and *k*_nr_ =(1 – *Φ*_F_)/*τ*_F_.

^*d*^Singlet (*τ*_S_) and triplet (*τ*_T_) lifetimes, and intersystem crossing quantum yields (*Φ*_ISC_) measured using transient absorption spectroscopy in toluene, under an inert atmosphere. Uncertainty for *τ*_s_: ±0.1 ns, and *τ*_T_: ±0.1 μs. Uncertainty for *Φ*_ISC_: ±0.1%.

^*e*^ *τ*_T_ and *Φ*_ISC_ measurements in air.

^*f*^Oxidation and reduction potentials determined using cyclic voltammetry in CH_2_Cl_2_ (0.1 mol L^–1^) using TBA-BArF4 as electrolyte, and calibrated using ferrocene.

^*g*^Half-width potentials, assigned to the BDP moiety.

^*h*^Irreversible potential of the main cathodic peak reported.

^*i*^Reported potential is a shoulder on the main BDP-centred reduction.

^*j*^Ir(i) **2** and Ir(iii) **3** have a very weak absorption and no emission, thus limited photophysical data could be obtained. In addition, no clear oxidation wave was observed.

^*k*^Absorption is outside the wavelength range examined.

**Fig. 4:**
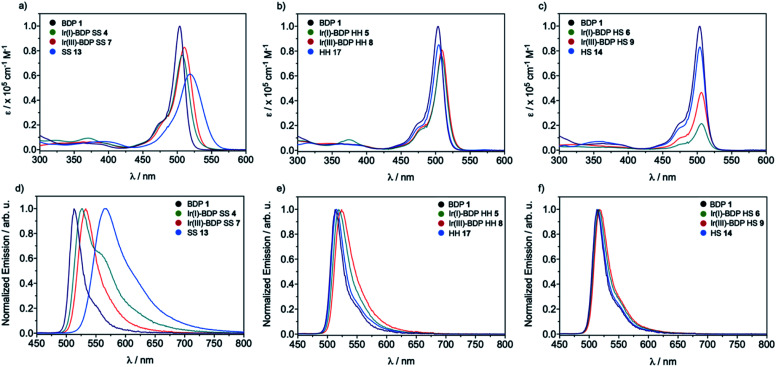
The UV-Vis absorption spectra ((a–c), 1 × 10^–5^ mol L^–1^) and normalised emission spectra (d–f) for BDP **1**, catalysts **4–9** and ligands **13**, **14** and **17** in toluene.

In general, the extinction coefficients of the compounds were found to decrease on moving from BDP **1** to the bifunctional catalysts (Table 1), indicating that catalysts **4–9** are less effective at absorbing light than BDP **1**, with the HS tethered catalysts **6** and **9** being the weakest absorbers. The absorption spectra of the HS **14** and HH **17** ligands have similar profiles to BDP **1** (Fig. 4), with a typical absorption near 504 nm, likely due to a ligand centred (LC) 
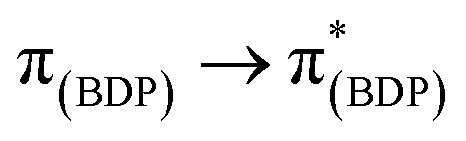
 transition.^38^,^39^ The HS **14** and HH **17** ligands also had similar absorption profiles to those of the HH and HS tethered bifunctional catalysts **5**, **6**, **8** and **9**, indicating that Ir coordination doesn't affect absorbance maxima (*λ*_abs_). In contrast, the SS **13** ligand had *λ*_abs_ at 519 nm, with this significant bathochromic shift, relative to BDP **1**, suggesting that the pyrazole moiety is involved in the ^1^LC 
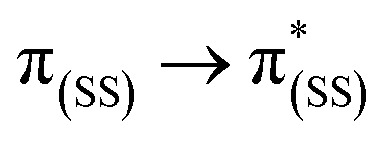
 transition for this ligand. In addition, the SS based bifunctional catalysts **4** and **7** had a hypsochromic shift of 9–12 nm, relative to the SS **13** ligand, suggesting that there is a significant electronic interaction between the BDP moiety and the Ir centre, which is consistent with that reported previously for similar species.^66^

The emission spectra of BDP **1** and the bifunctional catalysts **4–9** all feature one main band near 500 nm, with a shoulder at lower energy that is most pronounced for catalyst **4** (Fig. 4). In conjunction with the measured lifetimes (*τ*_F_), this band can be assigned as fluorescence, likely due to a ^1^LC 
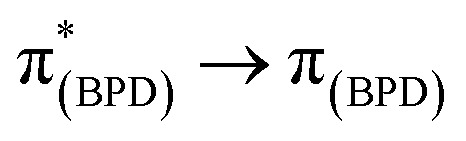
 transition. The emission maxima (*λ*_em_) of the HH and HS based catalysts **5**, **6**, **8** and **9** were comparable to BDP **1** and the ligands HS **14** and HH **17**. However, a significant bathochromic shift (52 nm) was observed for SS **13**, relative to BDP **1**, suggesting that the pyrazole moiety is involved in the ^1^LC 
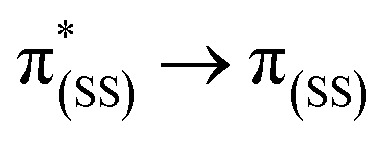
 transition for this ligand. In addition, significant hypsochromic shifts of 39 nm and 32 nm for *λ*_em_ were observed for the SS tethered catalysts **4** and **7**, relative to SS **13** (Fig. 4 and Table 1). Once again, these data indicate that the excited state of BDP **1** is altered the most when it is tethered to the Ir moiety through the SS tethering mode.

**Fig. 5:**
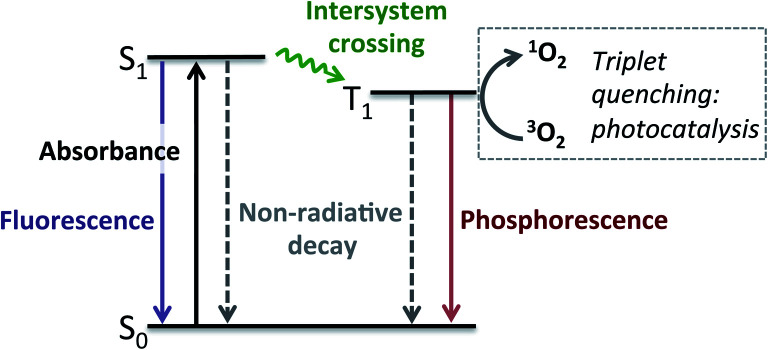
Simplified Jablonski diagram showing the possible excited state pathways.

Having established that BDP **1** and the bifunctional catalysts **4–9** are effective at absorbing light, it was important to consider the pathway(s) through which the excited species decay. Following light absorption into the first singlet excited state (S_1_), the excited photocatalyst can either: (1) decay to the singlet ground state (S_0_) *via* radiative (fluorescence) or non-radiative decay; or (2) undergo ISC to the first triplet excited state (T_1_), followed by radiative (phosphorescence) or non-radiative decay to S_0_ (Fig. 5). Importantly, for photocatalysis the ISC pathway is desired as this allows singlet oxygen to be generated. As such, the preference for these competing pathways is key for assessing photocatalytic potential.

As discussed above, all catalysts undergo fluorescent decay, which is undesired for photocatalysis, with no phosphorescence observed in our measurements. The fluorescence lifetimes (*τ*_F_) were all found to be in the lower nanosecond region (2–5 ns), with shorter lifetimes generally observed for the bifunctional catalysts **4–9**, relative to BDP **1** (Table 1). Importantly, the fluorescence quantum yield for BDP **1** was very high (*Φ*_F_ = 99%), indicating that the desired ISC pathway to T_1_ does not readily occur (<1%), making BDP **1** a poor photocatalyst. Pleasingly, the fluorescence quantum yields were significantly lower for catalysts **4–9** (23–63%), and ligands **13**, **14** and **17** (64–81%), indicating that undesired fluorescent decay from S_1_ is significantly reduced for these compounds. However, these lower *Φ*_F_ could be due to an increase in the desired ISC to T_1_,^38^,^48^,^67^ or undesired pathways such as non-radiative decay from S_1_ to S_0_ or fluorescence quenching due to intermolecular interactions, as observed for the BDP **1** + Ir(i) **2** and BDP **1** + Ir(iii) **3** mixtures.

**Fig. 6:**
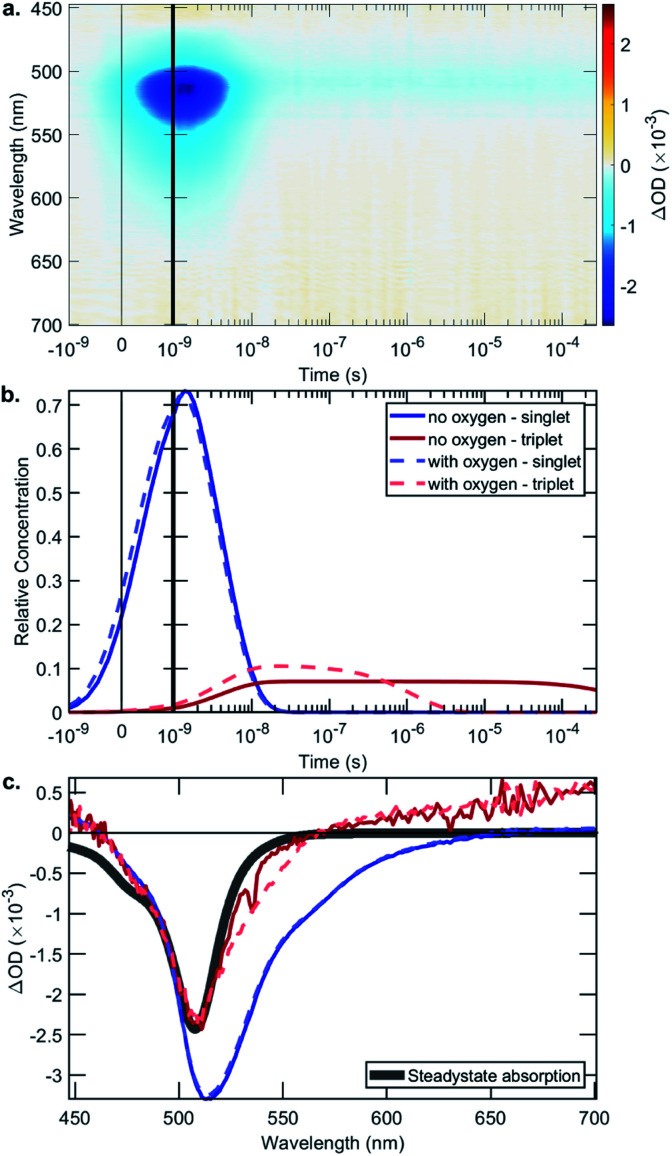
The data from the transient absorption spectroscopy measurements for Ir(i)–BDP SS **4**, chosen as a representative example: (a) the collected data, in the absence of oxygen, using an excitation wavelength of 355 nm; (b) fitted exponential decay showing the time dependent behaviour of the singlet and triplet excited states; and (c) the species associated spectra.

The rate constant data show that in general the bifunctional catalysts **4–9**, and ligands **13**, **14** and **17**, have lower rates of radiative decay (*k*_r_), and higher rates of non-radiative decay (*k*_nr_), relative to BDP **1**. This indicates that non-radiative decay pathways contribute significantly to the photophysical behaviour of species **4–9**. This is likely due to a large extent of thermal energy loss through rotation of the tethered Ir catalyst about the C–C bond that links BDP **1** and Ir(i) **2**/Ir(iii) **3**.^47^,^68^–^70^ Overall, the increased non-radiative decay from S_1_ to S_0_ observed for **4–9**, relative to BDP **1**, contributes to their lower *Φ*_F_. To determine if increased ISC from S_1_ to T_1_ is also contributing to these lower *Φ*_F_, transient absorption (TA) spectroscopy was used to examine the excited states of BDP **1** and catalysts **4–9** (Table 1, Fig. 6 and S24–S44†).

The TA signal comprises a negative ΔOD (Optical Density) in the 500–550 nm region, which could be caused by ground-state bleaching (GSB) or stimulated emission (SE). A GSB signal is indicative of there being molecules in an excited state, while the presence of SE indicates a singlet state. The surface shown in Fig. 6a is decomposed into relative concentrations (Fig. 6b) and spectra (Fig. 6c) by using a sequential model fit with two exponentials. Fig. 6c shows a comparison of the steady-state absorption, which mirrors the GSB, where it is evident that the first species has an additional lower energy region due to SE, which is not present in the triplet species.

The singlet lifetimes (*τ*_S_) were comparable to the previously measured *τ*_F_, as expected (Table 1). A long-lived T_1_ was not detected for BDP **1**, as anticipated based on the reported lifetime of 0.02 μs in acetonitrile.^71^ Interestingly, triplet excited states were only detected for three of the six bifunctional catalysts, indicating that the mode of tethering the Ir moiety to BDP **1** plays an important role in populating the triplet states (Table 1). The SS based bifunctional catalysts **4** and **7** were found to have the highest extent of ISC from S_1_ to T_1_, with ISC quantum yields (*Φ*_ISC_) of 7.0 and 7.2%, respectively. This is important as it clearly demonstrates that tethering of Ir(i) **2** or Ir(iii) **3** to BDP **1** promotes the desired ISC pathway, with the SS tethering mode being most effective. Interestingly, in the presence of air the *Φ*airISC for the Ir(i) based bifunctional catalysts **4** and **6** increased, whereas *Φ*airISC for Ir(iii)–BDP SS **7** was unchanged.

The triplet state lifetimes (*τ*_T_) were remarkably high for catalysts **4** and **7** (>500 μs) with Ir(i)–BDP HS **6** also having a long-lived triplet state (160 μs). To the best of our knowledge, the highest reported *τ*_T_ for a BDP-type compound is 539 μs,^72^ thus catalysts **4** and **7** represent one of the longest reported triplet state lifetimes for BDP-type compounds. These long triplet lifetimes are important for photocatalysis, as they increase the likelihood of productive triplet energy transfer, leading to ^1^O_2_ generation. This was confirmed through transient absorption measurements in the presence of air, which resulted in much shorter lifetimes (<2 μs), indicating that quantitative triplet quenching by oxygen occurs (>99%). Overall, these data suggest that Ir(i)–BDP SS **4** and Ir(iii)–BDP SS **7** have more desirable photophysical properties than BDP **1** and catalysts **5**, **6**, **8** and **9**, and thus should be more effective photocatalysts.

### Cyclic voltammetry

**Fig. 7:**
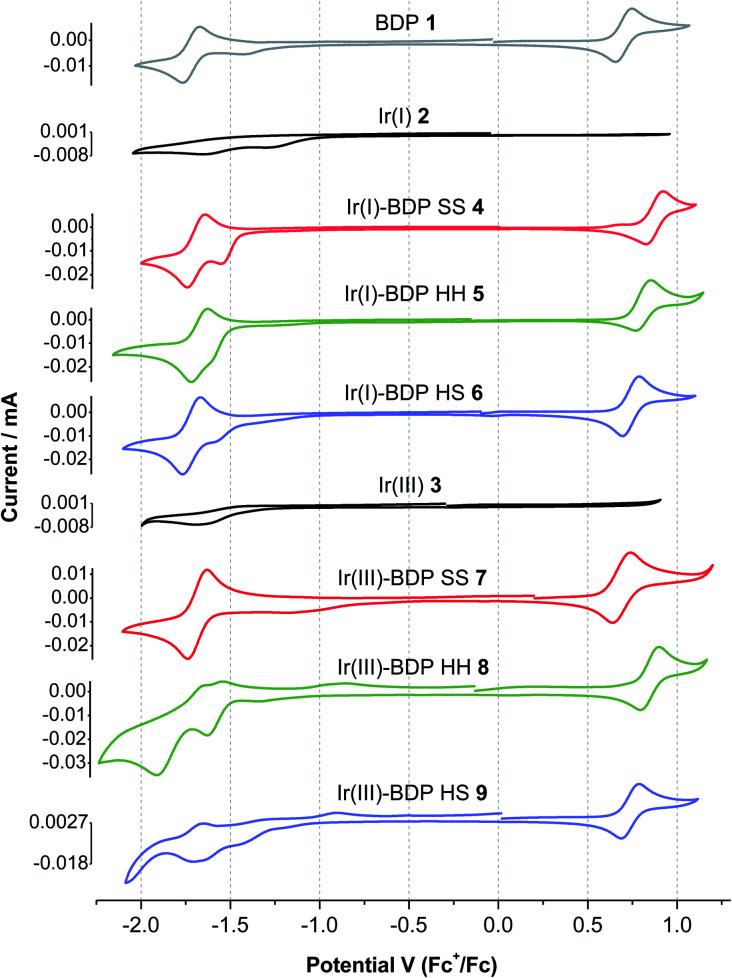
Cyclic voltammograms of the parent compounds BDP **1**, Ir(i) **2** and Ir(iii) **3**, and the bifunctional catalysts **4–9**, measured in a 0.1 M solution of TBA-BArF4 in dichloromethane under argon. Ferrocene was used as internal standard. Scan rate: 100 mV s^–1^.

To provide insight into electrochemical behaviour and the potential for redox applications, cyclic voltammetry measurements were performed on catalysts **1–9** and the ligands **13**, **14** and **17** (Table 1, Fig. 7 and S45†). Cyclic voltammograms (CVs) were recorded using a three-electrode setup, with a glassy carbon working electrode, in a 0.1 M solution of tetrabutylammonium BArF4 (TBA-BArF4) in dichloromethane. TBA-BArF4 was chosen as the supporting electrolyte as catalysts **2–9** all contain the BArF4 counterion, and as TBA-BArF4 can allow multiple oxidation processes of the analytes to be resolved due to its weakly coordinating nature.^73^ All potentials are reported *vs.* the ferrocene/ferrocenium couple.

The parent BDP **1** species was found to undergo well-resolved one electron oxidation (0.70 V) and reduction (–1.72 V) events, typical of BDP-type compounds.^38^,^70^,^74^ Good reversibility was observed, with the quotient of the anodic and cathodic peak currents close to unity and an approximately linear relationship between the peak currents and scan rate (Tables S5 and S6†). Similar reversible behaviour was observed for the frameworks **13**, **14** and **17**, however better reversibility was observed at higher scan rates. In addition, the oxidation and reduction potentials for **13**, **14** and **17** were comparable to BDP **1**, suggesting that the pyrazole moiety has no significant effect on the redox behaviour of the BDP **1** moiety (Table 1). While BDP **1** and the ligands **13**, **14** and **17** exhibited electrochemical reversibility, both the parent Ir(i) **2** and Ir(iii) **3** catalysts showed only weakly pronounced irreversible reduction events near –1.5 V, with no distinct oxidation events.

In general, the electrochemical behaviour of catalysts **4–9** was dominated by the BDP **1** fragment, with reversible oxidation and reduction events near 0.7 and –1.7 V, respectively. Poorly defined reduction peaks due to the Ir(i) **2** or Ir(iii) **3** moieties were also present in the CVs (Fig. 7). The splitting between the major oxidation and reduction events varies from 2.3 to 2.6 V, which is comparable to reported alkyl,^75^ phenyl^70^ and platinum^34^ substituted BDP derivatives. Comparison of the ligand frameworks **13**, **14** and **17** with the Ir(i)-based catalysts **4–6** indicate that coordination of Ir(i) to the ligand has no significant effect on the reduction potential, however the oxidation potential increased, with this increase most pronounced for the SS framework (+0.23 V). This suggests that the Ir(i) species affects the BDP moiety most when it is tethered through the conjugated SS tethering mode, which is consistent with our photophysical measurements.

Coordination of the Ir(iii) moiety to the ligand frameworks **13**, **14** or **17** generally led to more complex electrochemical behaviour, with multiple reduction events observed for catalysts **8** and **9**. The Ir(iii)-based catalysts **7–9** had higher oxidation potentials than the corresponding ligand frameworks, as seen for Ir(i), however the increases were less pronounced. In addition, the maximum increase was now seen for the HH catalyst **8** (+0.09 V). This data indicates that coordination of Ir(iii)ClCp* to the ligand frameworks leads to different electrochemical behaviour than that seen upon Ir(i)(CO)_2_ coordination. Overall, catalysts **4–9** exhibit reversible electrochemical behaviour, that is dominated by the BDP **1** fragment, highlighting their potential for use as catalysts for redox processes. There were some interesting trends in potentials observed when changing the Ir species and tethering mode, demonstrating the possibility to tune the redox potential of the catalysts through tethering modes.

### X-ray absorption spectroscopy

**Fig. 8:**
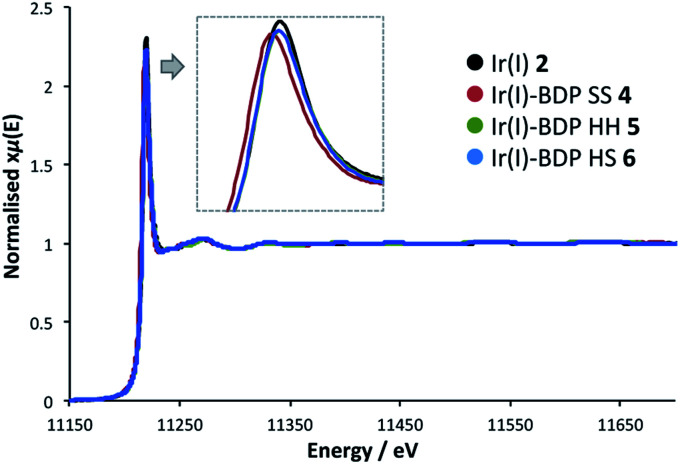
The XAS spectra at the Ir L_3_ edge for the Ir(i) based complexes.

To probe whether BDP is affecting the local electronic structure of Ir in the bifunctional catalysts **4–9**, X-ray Absorption Spectroscopy (XAS) measurements were performed at the Ir L_3_ edge (11–12.5 keV) in transmission mode. The XAS spectra indicate that there is a decrease in the absorption edge energy on moving from Ir(i) **2** to the bifunctional catalysts **4–6** (Fig. 8). This decrease in edge energy is smaller for Ir(i)–BDP HH **5** and Ir(i)–BDP HS **6** (<0.5 eV), with a more significant decrease observed for Ir(i)–BDP SS **4** (1 eV). This decrease in edge energy is characteristic of the metal centre becoming more negative, with a decrease of 1 eV being significant (for example, a difference of 1.6 eV between Ir(iv) and Ir(iii) has been reported^76^). Thus, the trend observed suggests that BDP is transferring electron density to Ir(i), making it less positive, with this effect most pronounced for Ir(i)–BDP SS **4**. This is consistent with the IR data, which also suggests electron transfer from BDP to Ir(i) is occurring, and our photophysical and electrochemical studies which suggest that the greatest interaction between Ir and BDP occurs for the SS tethering mode.

**Fig. 9:**
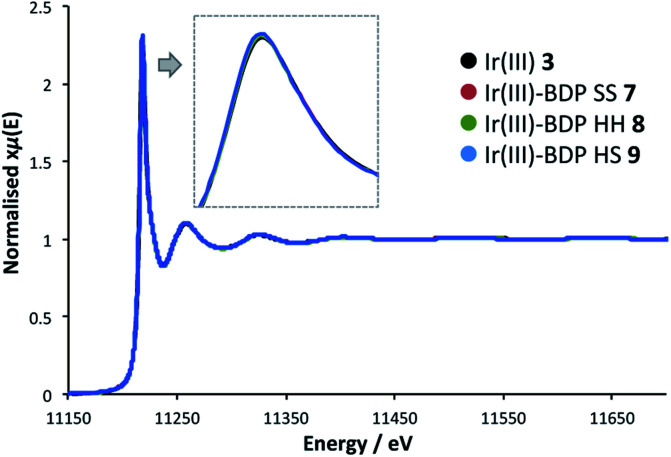
The XAS spectra at the Ir L_3_ edge for the Ir(iii) based complexes.

Interestingly, no shift in edge energy was observed for the Ir(iii)-based catalysts **3** and **7–9** (Fig. 9), suggesting that transfer of electron density from BDP to Ir(iii) does not occur. Overall, these data indicate that the local electronic structure of Ir(i) **2** becomes less positive when tethered to BDP, while Ir(iii) **3** is unaffected.

### Catalytic investigations

Photocatalytic applications of BDP-type dyes generally rely on the generation of reactive singlet oxygen,^35^,^37^,^46^,^77^,^78^ thus singlet oxygen quantum yields (*Φ*_Δ_) are a good indication of photocatalytic potential. Incorporation of heavy atoms into BDP can increase *Φ*_Δ_ due to enhanced intersystem crossing from S_1_ to T_1_.^35^ This phenomenon was a key part of our bifunctional catalyst design, as we sought to enhance the photocatalytic competency of BDP **1** through incorporation of a ‘heavy atom’, whilst also providing a separate catalytic site that can facilitate complimentary catalytic reactivity. To determine whether our bifunctional catalysts are superior singlet oxygen generators to BDP **1**, *Φ*_Δ_ was determined for each catalyst under green LED irradiation (max wavelength = 510 nm) using 1,3-diphenylisobenzofuran as a singlet oxygen trap (Table 2).^36^,^37^,^79^,^80^ As alcoholic solvents are often used for photocatalysis, the high boiling tertiary amyl alcohol solvent was used.

**Table 2:** The singlet oxygen quantum yield (*Φ*_Δ_) for each catalyst, measured in tertiary amyl alcohol. Average and error (half the range) of 2–3 replicate experiments reported. See ESI for experimental details (Fig. S11–S23)

Complex	*Φ*_Δ_/%
BDP **1**	2.6 ± 0.1
BDP **1** + Ir(i) **2**	3.2 ± 0.1
Ir(i)–BDP SS **4**	12.3 ± 0.4
Ir(i)–BDP HH **5**	3.6 ± 0.1
Ir(i)–BDP HS **6**	3.0 ± 0.2
BDP **1** + Ir(iii) **3**	3.9 ± 0.2
Ir(iii)–BDP SS **7**	7.5 ± 1.7
Ir(iii)–BDP HH **8**	1.2 ± 0.1
Ir(iii)–BDP HS **9**	2.4 ± 0.2

It was found that BDP **1** had a singlet oxygen quantum yield of 2.6% in tertiary amyl alcohol, which is comparable to the reported value of 1% for BDP **1** in dichloromethane.^37^ Pleasingly, *Φ*_Δ_ for the side–side tethered bifunctional catalysts **4** and **7** were higher than that for BDP **1**, suggesting that tethering Ir(i) **2** or Ir(iii) **3** to BDP **1** can promote ISC. The most efficient singlet oxygen generator was Ir(i)–BDP SS **4**, with a 5-fold increase in *Φ*_Δ_ relative to BDP **1**. It should be noted that greater increases in *Φ*_Δ_ would likely be observed if the heavy atom was attached directly to the BDP **1** core,^34^,^35^,^81^ and thus the smaller changes in *Φ*_Δ_ observed here are likely due to the Ir centre being separated from BDP **1** by the tether. Despite this, the variation in *Φ*_Δ_ between the bifunctional catalysts indicates that both the tethering mode and the nature of the Ir centre (Ir(i) **2***vs.* Ir(iii) **3**) affects singlet oxygen generation. This is important as it clearly highlights the need to consider the tethering mode when developing tethered dual catalysts.

To determine how singlet oxygen quantum yield affects photocatalytic efficiency, the catalytic competency of a representative selection of catalysts was examined using the oxidation of benzylamine **18** as the model reaction. This reaction was chosen as iodo-substituted BDP compounds have previously been shown to effectively promote this oxidation reaction.^81^ The complexes BDP **1**, Ir(i)–BDP SS **4**, Ir(i)–BDP HH **5** and Ir(iii)–BDP SS **7** were tested as these catalysts cover a range of *Φ*_Δ_. It was found that the bifunctional catalysts **4**, **5** and **7** were significantly better photocatalysts than BDP **1** (Table 3). Control experiments in the presence of the singlet oxygen scavenger 1,4-diazabicyclo[2.2.2]octane (DABCO) confirm that singlet oxygen is involved in the reaction mechanism for all catalysts (Table S8†). Further control reactions indicate that Ir(i) **2** and Ir(iii) **3** are inefficient photocatalysts; this is important as it demonstrates that tethering a transition metal catalyst, that isn't photocatalytically active, to an organic photocatalyst can significantly enhance photocatalytic activity.

**Table 3:** The efficacy of the different catalysts at promoting photocatalytic oxidation of benzylamine **18** to the product **19**^*a*^

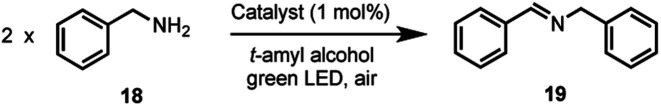			
Catalyst	Conversion to product **19**/%		
	4 h	16 h	24 h
BDP **1**	9 ± 3	24 ± 5	32 ± 7
Ir(i)–BDP SS **4**	23 ± 4	59 ± 7	79 ± 1
Ir(i)–BDP HH **5**	18 ± 1	50 ± 3	73 ± 5
Ir(iii)–BDP SS **7**	18 ± 4	46 ± 5	72 ± 1
Ir(i) **2**	0	6	10
Ir(iii) **3**	0	0	1
BDP **1** + Ir(i) **2**	12 ± 1	42 ± 1	59 ± 2
BDP **1** + Ir(iii) **3**	12 ± 1	41 ± 3	57 ± 3
BDP **1** + NaBArF4	8 ± 4	24 ± 2	42 ± 3
BDP **1** + NaCl	6 ± 3	21 ± 2	35 ± 2

^*a*^Conditions: benzylamine (0.4 mmol), catalyst (0.002 mmol), additive, where appropriate (0.002 mmol) 2,4,6-trimethoxybenzene (internal standard, 0.2 mmol), *t*-amyl alcohol (0.5 mL) in a vial open to air, with aliquots taken at different time points. Average and error (half the range) of 2 replicate experiments reported.

Comparison of the extent of conversion to the product **19** with *Φ*_Δ_ (Fig. S53†) gives a moderate correlation (*R*^2^ = 0.58), indicating that singlet oxygen generation is not rate-determining for this reaction, as seen for other processes involving singlet oxygen.^37^ This is supported by the catalytic enhancements observed when using untethered mixtures of ‘BDP **1** + Ir(i) **2**’ or ‘BDP **1** + Ir(iii) **3**’, relative to BDP **1** (Table 3); this was unexpected as these mixtures have *Φ*_Δ_ similar to BDP **1** (Table 2). These data suggest that factors, other than simply *Φ*_Δ_, contribute to the synergistic effects observed when using dual BDP **1**–Ir(i) **2**/Ir(iii) **3** systems in photocatalysis. Control reactions using a 1 : 1 mixture of BDP **1** and NaBArF4 suggest that the BArF4 anion isn't contributing to the enhancements seen, while reactions using a 1 : 1 mixture of BDP **1** and NaCl confirm that it is not simply a salt effect (Table 3). Therefore, we postulate that the Ir centre is interacting with specie(s) along the reaction coordinate, contributing to the catalytic enhancements observed when using either tethered or untethered BDP **1**–Ir(i) **2**/Ir(iii) **3** systems (Table 3). While mechanistic investigations into this phenomenon are ongoing, it is likely that the effect of the Ir centre in the tethered dual catalysts is two-fold; it increases *Φ*_Δ_ and is also directly involved in the benzylamine **18** oxidation reaction mechanism.

**Fig. 10:**
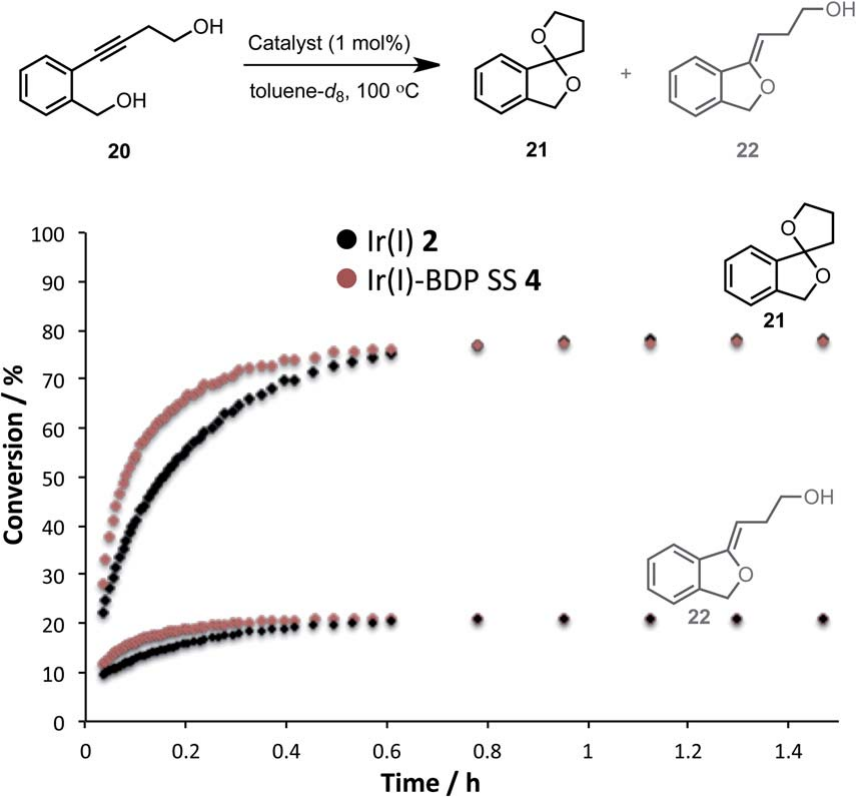
Formation of the products **21** and **22** over time, monitored using *in situ*^1^H NMR spectroscopy. Conditions: diol **20** (0.2 mmol), catalyst (0.002 mmol), toluene-*d*_8_ (0.5 mL) under argon. Conversion calculated relative to the starting material **20**.

Having established that the bifunctional species are superior photocatalysts to BDP **1**, attention will turn to the Ir(i) and Ir(iii) moieties. In this section the parent catalysts **2** and **3** were compared with the SS based complexes **4** and **7**, as the SS framework was most favourable for photocatalysis. The catalytic reactivity of the Ir(i) moiety was assessed for promoting the dihydroalkoxylation of 4-(2-(hydroxymethyl)-phenyl)but-3-yn-1-ol **20** to produce products **21** and **22**. Kinetic analyses using *in situ*^1^H NMR spectroscopy indicate that both Ir(i) **2** and Ir(i)–BDP SS **4** are effective at facilitating this reaction, and comparable product ratios were observed (Fig. 10). However, Ir(i)–BDP SS **4** was more efficient than Ir(i) **2**, indicating that tethering BDP to Ir(i) enhances the catalytic reactivity of the Ir(i) moiety, possibly due to electron transfer from BDP to Ir(i) as suggested by the XAS data. This is important as it indicates that there are two types of synergy between the catalytic centres in Ir(i)–BDP SS **4**: Ir(i) **2** enhances the photocatalytic ability of BDP **1** (Table 3) and BDP **1** enhances the catalytic reactivity of Ir(i) **2** (Fig. 10).

**Fig. 11:**
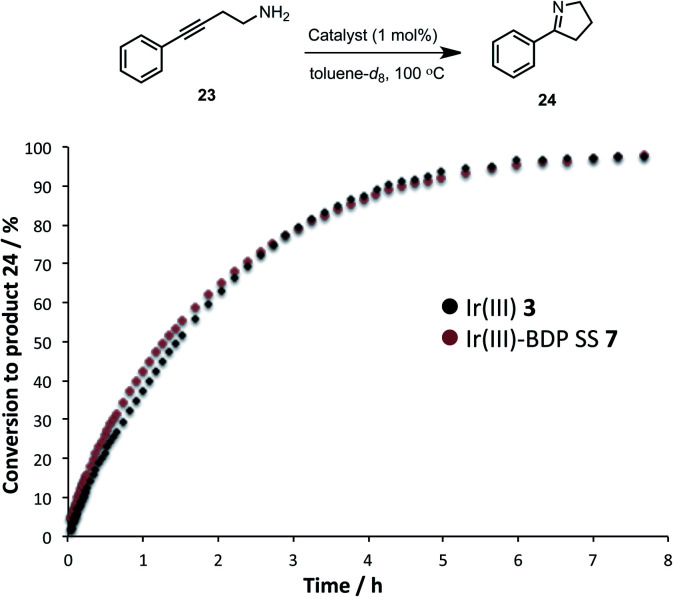
Formation of the product **24** over time, monitored using *in situ*^1^H NMR spectroscopy. Conditions: amine **23** (0.2 mmol), catalyst (0.002 mmol), toluene-*d*_8_ (0.5 mL) under argon. Conversion calculated relative to starting material **23**.

The activity of the Ir(iii) based compounds **3** and **7** were assessed for promoting the intramolecular hydroamination of 4-phenylbut-3-yn-1-amine **23** (Fig. 11). Kinetic analyses using *in situ*^1^H NMR spectroscopy indicate that Ir(iii)–BDP SS **7** can effectively promote hydroamination, with identical reaction profiles obtained for Ir(iii) **3** and Ir(iii)–BDP SS **7**. This indicates that the Ir(iii) **3** moiety remains catalytically active in Ir(iii)–BDP SS **7**. Overall, these data indicate that the reactivity of the Ir(i) **2** and Ir(iii) **3** moieties are not inhibited when tethered to BDP **1**, with comparable, or better, catalytic results obtained for the reactions considered. This is important, as it demonstrates that the Ir centre is still able to act as a competent catalyst when incorporated into the bifunctional catalyst, highlighting the dual role of Ir as both a photocatalytic enhancer and a unique reaction centre available to promote alternate reactivity.

**Scheme 4:**
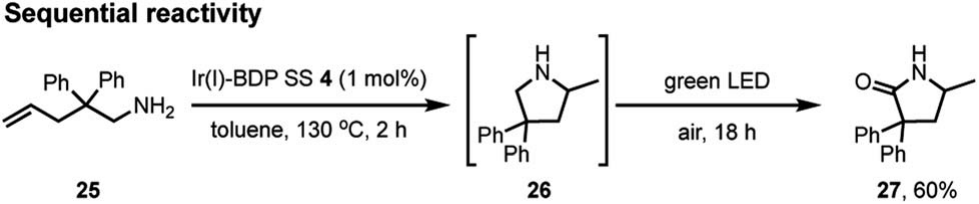
The sequential hydroamination – oxidation reaction of compound **25** to produce the lactam **27**. Conditions: aminoalkene **25** (0.4 mmol), catalyst **4** (0.004 mmol), toluene (2 mL) under argon. Heated for 2 hours at 100 °C, then cooled to room temperature, opened to air and irradiated for 18 hours. Isolated yield reported.

To further validate the bifunctional character of the novel tethered catalysts **4–9**, their ability to promote both sequential and stimuli-responsive chemical reactivity was demonstrated using Ir(i)–BDP SS **4** as a representative catalyst. This bifunctional catalyst could promote a novel tandem reaction, where the amine **25** first undergoes Ir(i) catalysed intramolecular hydroamination to produce the intermediate **26**, followed by BDP promoted photocatalytic oxidation to generate product **27** in 60% isolated yield over two steps (Scheme 4). This is important as it provides an alternative synthetic approach to the medicinally important lactam framework **27**. In addition, there was a significant advantage to chemically tethering the catalysts together, as demonstrated by the much lower yield of the product **27** (17%) obtained when using a mixture of BDP **1** and Ir(i) **2**.

**Scheme 5:**
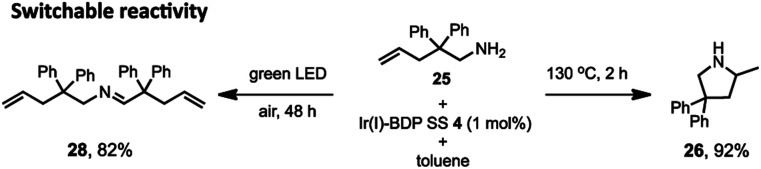
The switchable reactivity of compound **25**, where heat leads to generation of compound **26** (right) and light irradiation produces compound **28** (left). Conditions: aminoalkene **25** (0.4 mmol), catalyst **4** (0.004 mmol), toluene (2 mL). Either heated under argon to give product **26**, or irradiated under air to give product **28**. Conversion determined using ^1^H NMR spectroscopy, relative to the internal standard 2,4,6-trimethoxybenzene (0.4 mmol).

Lastly, switchable chemical reactivity was demonstrated using the amine **25** under different external stimuli, where use of heat activated the Ir(i) moiety in Ir(i)–BDP SS **4**, resulting in hydroamination to compound **26**. Conversely, light irradiation activated the BDP moiety in catalyst **4**, leading to photocatalytic oxidation of **25** to the product **28** (Scheme 5). This stimuli-responsive behaviour of the tethered dual catalyst is significant, as controlling reactivity through external stimuli is an emergent field as it is central to the development of programmable and adaptive materials, and controllable sequential reactions.^82^,^83^ Overall, these are the first reported examples of sequential and switchable reactivity using BDP-based tethered dual catalysts.

## Conclusions

In summary, we have synthesised a series of novel tethered dual catalysts that feature a BDP photocatalyst and a thermally activated iridium catalyst, with synergistic interactions between the catalysts examined using a range of techniques. Absorption and emission spectroscopy revealed that the excited state is centred on the BDP **1** moiety of the bifunctional catalysts, with the excited state altered most when the SS tethering mode is used. Interaction between BDP and Ir(i) was also observed in the XAS data for catalysts **4–6**, with the greatest extent of electron transfer from BDP to Ir(i) seen for the SS-tethered catalyst **4**. Interestingly, the XAS data suggest that electron transfer from BDP to Ir(iii) does not occur in catalysts **7–9**.

Transient absorption spectroscopy indicated that tethering Ir(i) **2** or Ir(iii) **3** to BDP **1** can increase intersystem crossing from the singlet to the triplet excited state, with long lived triplet states located for Ir(i)–BDP SS **4**, Ir(i)–BDP HS **6** and Ir(iii)–BDP SS **7**. The highest extent of ISC and the longest triplet lifetimes (>500 ns) were observed for the SS-tethered catalysts **4** and **7**, suggesting that the SS tethering mode will be most effective for photocatalysis. The superior photocatalytic ability of catalysts **4** and **7** was confirmed through singlet oxygen quantum yield measurements and photocatalytic investigations. In addition, cyclic voltammetry indicated that catalysts **4–9** exhibit reversible electrochemical behaviour that is dominated by the BDP moiety. The oxidation and reduction potentials varied depending on the nature of the Ir species and tethering mode, highlighting the tunability of the bifunctional catalysts' redox potentials.

Importantly, Ir(i) and Ir(iii) were shown to remain catalytically active in the bifunctional catalysts **4** and **7** for representative hydroamination and dihydroalkoxylation reactions. This allowed the first demonstration of tethered photo–transition metal dual catalysts to promote both sequential and stimuli-responsive chemical reactivity. The key fundamental insight into catalytic cooperatively presented in this manuscript lays the groundwork for rationally designing tethered photo–transition metal dual catalysts in the future, and utilising these species to develop novel chemical reactivity.

## Conflicts of interest

There are no conflicts to declare.

## Supplementary Material

Supplementary information

Crystal structure data
