# Supplementary material for: Development of tethered dual catalysts: synergy between photo- and transition metal catalysts for enhanced catalysis
Source: Chem Sci. 2020 Jun 5;11(24):6256–67. doi: 10.1039/d0sc02703k (PMC7480183; doi:10.1039/d0sc02703k)
Supplement: Supplementary file 1 [file SC-011-D0SC02703K-s001.pdf]

Electronic Supplementary Material (ESI) for Chemical Science.
This journal is © The Royal Society of Chemistry 2020

Supporting information

Development of Tethered Dual Catalysts: Synergy between Photo- and Transition Metal Catalysts for Enhanced Catalysis

Danfeng Wang, Robert Malmberg, Indrek Pernik, Shyamal K. K. Prasad, Max Roemer, Koushik Venkatesan, Timothy W. Schmidt, Sinead T. Keaveney* and Barbara A. Messerle*

Table of Contents

General Information 2

Experimental Procedures 4

- Synthesis of catalysts **1**-**3** 4
- Synthesis of the ligand frameworks SS **13**, HS **14** and HH **17** 6
- Synthesis of the bifunctional catalysts **4**-**9** 10
- Synthesis of substrate **25** 14
- Variable temperature ^{1}H NMR experiments for complex **4** 15

X-ray Crystallographic Data 16

Infrared Spectroscopy Measurements 21

Photophysical Measurements 23

- The absorption and emission spectra of the untethered catalyst mixture 23
- Fluorescent lifetime measurements 24
- Singlet oxygen quantum yield measurements 26
- Transient absorption measurements 39

Cyclic Voltammetry 55

X-Ray Absorption Spectroscopy 58

Catalytic Investigations and Control Reactions 60

- Photocataysis set-up 60
- Phooxidation of benzylamine 61
- Dihydroalkoxylation 64
- Intramolecular hydroamination 66
- Sequential reaction 69
- Switchable reaction 72

References 74

NMR Spectra 75

- Catalysts **4**-**9** 75
- Ligands **13**, **14**, **17** 96
- Intermediates **12**, **30** and substrate **25** 105
- Products from the tandem reaction and switchable reaction 108

High Resolution Mass Spectra 111

Elemental Microanalysis Reports 116

General Information

Reagents and solvents

In general, all compounds were commercially available (Sigma Aldrich/Merck, Alfa Aesar and Combi Blocks) and used as received unless otherwise stated, and $IrCl_3{\cdot}xH_2O$ was purchased from Precious Metals Online. Di(1H-pyrazol-1-yl)methanone **16**,[1] 4-(2-(hydroxymethyl)phenyl)but-3-yn-1-ol **20**[2] and 4-phenylbut-3-yn-1-amine **23**[3] were synthesised according to literature procedures. For all air- or moisture-sensitive procedures the solvents (toluene, dichloromethane, tetrahydrofuran, pentane or diethyl ether) were from a LC Technology solvent purification system and stored under an inert atmosphere in glass ampoules fitted with a J. Young's Teflon valve. *t*-Amyl alcohol was purchased from Sigma Aldrich and saturated with air before use. Solvents for extractions and columns such as dichloromethane, methanol, n-hexane and ethyl acetate were technical grade. Deuterated solvents were purchased from Cambridge Stable Isotopes and used as received. The compressed gases argon (> 99.999%) and carbon monoxide (> 99.5%) were obtained from Air Liquide and used as received. The nitrogen gas for the Schlenk line is from in-house liquid nitrogen boil-off.

Experimental techniques

Whether an inert or air atmosphere was used for a reaction is specified in the experimental section. All manipulations that were performed under an inert atmosphere were done so using standard Schlenk techniques[4] or in a glovebox (LC Technology Solutions Inc.). Unless otherwise stated, all reaction work-ups were carried out in air. The term 'under reduced pressure' refers to use of a rotary evaporator, and '*in vacuo*' indicates use of a high vacuum pump attached to a Schlenk line.

The photocatalysis experiments were performed using a green LED light strip (12 V green 5 M 3528 SMD 300 LED strips). The spectra of the green LED had a maximum wavelength of 510 nm.

Characterisation

All ^{1}H, $^{13}C\{^{1}H\}$, ^{11}B and ^{19}F NMR spectra were recorded on either a Bruker 400, 500 or 600 MHz (^{1}H) spectrometer. ^{1}H and $^{13}C\{^{1}H\}$ NMR chemical shifts were referenced internally using the residual solvent resonance. Unless otherwise stated, spectra were recorded at 298 K, and chemical shifts (δ), are quoted in parts per million (ppm). Multiplicity is abbreviated as: s (singlet); d (doublet); t (triplet); q (quartet); m (multiplet) and br (broad). The annotations of the complexes **4-9** and ligands SS **13**, HH **14**, HS **17** were determined by two-dimension NMR spectroscopy experiments, including homonuclear correlation spectroscopy (COSY), nuclear overhauser effect spectroscopy (NOESY), heteronuclear single-quantum correlation spectroscopy (HSQC) or heteronuclear multiple-bond correlation spectroscopy (HMBC). Air sensitive NMR samples were prepared in an inert atmosphere glovebox or by vacuum transfer of deuterated solvents into NMR tubes fitted with a J. Young's Teflon valve.

Infrared data were acquired on Nicolet iS5 with ATR accessory (iD5) manufactured by ThermoScientific and interpreted using the OMNIC8 software.

High Resolution Mass Spectrometric (HRMS) analyses were performed on a Q Exactive™ Plus (Thermo Scientific), spray voltage: 4.5 kV.

UV-Vis measurements were carried out in spectrophotometric grade solvent on a Cary 8458 UV-Vis spectrophotometer from Agilent Technologies, using a step size of 1 nm. Emission spectra were acquired on an

Edinburgh FLS980 spectrophotometer using 450 W Xenon lamp for excitation, using λ_{ex} = 371 nm, step size = 1 nm, integration time = 0.3 s. Absolute quantum yields were measured using an integrating sphere from Edinburgh Instruments, using an integration time of 0.3 s. The quantum yield was measured once for each sample, with an estimated instrumental error of ± 5%.

Fluorescence lifetimes (τ_F) in solution were measured with an EPL-VIS Picosecond Pulsed Diode Laser (λ_{max} = 371.8 nm, pulse width = 58.8 ps, pulse period = 1 µs) from Edinburgh Instruments. All data was tail fit, or reconvolution fit, to a mono-exponential term, with all fitting having χ^2 between 1.0 and 1.3, resulting in a maximum standard deviation of 0.004 ns. LUDOX HS-40 colloidal silica (40 wt. % suspension in H_2O) was used to measure the instrument response function (IRF).

Transient absorption (TA) spectroscopy was carried out using a custom home-built system. The probe was a white light supercontinuum generated by focusing 780 nm pulse (1 kHz, Clark-MXR CPA 2210, 150 fs, Ti:sapphire regenerative amplifier) into an undoped YAG crystal. A home built prism spectrometer was used to disperse the light onto a line-scan camera and allow simultaneous collection across the visible spectrum. The excitation pulse was produced by an electronically triggered pulsed laser (355 nm, 500 Hz, InnoLas picolo 1), this pulse energy was attenuated by neutral density filter to give ~300 µJ cm^{-2} pulse at the sample. The delay between the probe and excitation pulses controlled by a delay generator (Highland Technology T560). The generated supercontinuum was collected shot-to-shot and processed after each measurement was completed. The collected probe intensity *vs* wavelength spectrum was converted to differential transmission by comparing the shots collected with excitation as indicated by readout of a photodiode which had residual excitation light directed towards it. At each wavelength, any data point greater than 2.5 standard deviations from the mean were removed, and the remaining shots averaged. The above process was completed multiple times for each measured excitation-probe delay. The final dataset was produced by averaging the multiple collected data points at each excitation-probe delay, to give a 2D time-wavelength dataset. All samples were prepared and stored in a nitrogen atmosphere glovebox. For measurements, samples were transferred to a 1 mm pathlength cuvette with a J. Youngs tap attached to maintain an oxygen-free environment. For measurements with oxygen, the sample was exposed to ambient air and then shaken vigorously three times. Samples were prepared in toluene at various concentrations to maintain an optical density of ~0.09 at 355 nm.

Cyclic voltammetry (CV) measurements were performed using an Autolab PGSTAT 302N potentiostat (Eco Chemie, Netherlands) that was interfaced with a computer running Nova 1.11 software. A conventional three-electrode system was used, in which the glassy carbon working electrode was purchased from CH Instruments Inc. (TX, USA), the Pt/Ti rod electrodes working as the counter electrode and reference electrode. The glassy carbon electrode was polished with a slurry of alumina (0.05 µm) in distilled water using a micropolishing cloth (Buehler, IL, USA), rinsed with distilled water and methanol, and then dried under a flow of nitrogen before use. Ferrocene was used as the internal standard to calibrate the measured potentials.

The solid state XAS measurements were performed at the Australian Synchrotron using the 'XAS Beamline' at the Ir L_3 edge (ca. 11.2 keV) in transmission mode, using a Si(111) monochromator and a 1.9T Wiggler. The solid state samples were prepared as mixtures with cellulose as binding agent, and pressed into a 7 mm pellet, with the XAS measurements performed at room temperature under an air atmosphere. Data analysis was performed using the IFEFFIT data analysis package (Demeter),[5] using the Athena and Artemis programs.

Experimental Procedures

Synthesis of catalysts 1-3

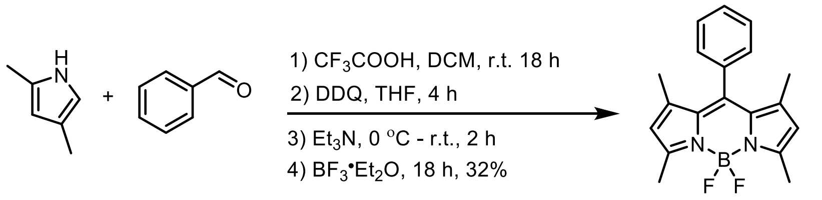

BDP **1**

BDP **1** was synthesised according to a literature procedure.[6] A mixture of 2,4-dimethylpyrrole (3.80 g, 40 mmol), benzaldehyde (2.12 g, 20 mmol) and trifluoroacetic acid (60 µL, 0.8 mmol) in dry CH_2Cl_2 (200 mL) was stirred at room temperature under a nitrogen atmosphere for 18 hours. 2,3-Dichloro-5,6-dicyano-1,4-benzoquinone (DDQ, 4.54 g, 20 mmol) in dry THF (20 mL) was then added slowly to the reaction mixture and stirred for an additional 4 hours. The mixture was then cooled to 0 °C, triethylamine (Et_3N, 60 mL) was added, and then the mixture was warmed to room temperature and stirred for 2 hours. $BF_3{\cdot}Et_2O$ (60 mL) was then added, and the reaction mixture stirred for additional 18 hours at room temperature. Saturated sodium bicarbonate solution (200 mL) was then added into the mixture. The organic layer was separated and concentrated under reduced pressure to approximately 50 mL followed by addition of water (200 mL). The mixture was exacted with Et_2O (200 mL x 3), the combined organic extracts were dried over anhydrous Na_2SO_4 and the solvent was evaporated under reduced pressure. The crude product was purified using flash column chromatography on silica gel eluting with CH_2Cl_2-hexane (1:1, v/v, R_f = 0.3) to give the title compound as an orange powder in 32% yield (2.06 g). ^{1}H NMR (400 MHz, Methylene Chloride-d_2) δ 7.54 – 7.43 (m, 3H), 7.34 – 7.22 (m, 2H), 5.99 (s, 2H), 2.49 (s, 6H), 1.37 (s, 6H) ppm. ^{11}B NMR (128 MHz, Methylene Chloride-d_2) δ 0.72 (t, J = 33.0 Hz) ppm. ^{19}F NMR (376 MHz, Methylene Chloride-d_2) δ -146.29 (q, J = 32.7 Hz) ppm. The data matches that reported in the literature (in Chloroform-d).[6]

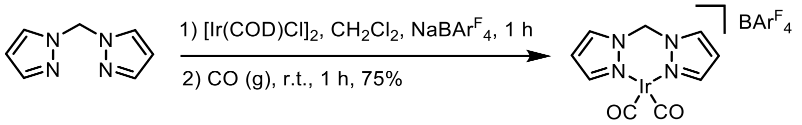

29 **2**

Complex **2** was synthesised according to a modified literature procedure.[7] Di(1H-pyrazol-1-yl)methane **29** (148 mg, 1.0 mmol), $NaBAr^F_4$ (886 mg, 1.0 mmol) and $[Ir(COD)Cl]_2$ (336 mg, 0.50 mmol) was dissolved in 30 mL of dry CH_2Cl_2 under a nitrogen atmosphere, and the mixture stirred at room temperature for 1 hour. The formed COD-intermediate was then converted to the desired product under an absolute CO atmosphere (*via* three freeze-pump-thaw cycles, the flask then backfilled with nitrogen, and finally a balloon of CO gas was attached) and allowed to react at room temperature for 1 hour. The reaction mixture was filtered through Celite under a nitrogen atmosphere and the solvent was removed *in vacuo*. The crude product was recrystallised from pentane-CH_2Cl_2 under air to afford the product as a light-yellow solid in 75% yield (945 mg). ^{1}H NMR (400 MHz, Chloroform-d) δ 7.83 (d, J = 2.5 Hz, 2H), 7.62 (br s, 8H), 7.49 (d, J = 2.5 Hz, 2H), 7.47 – 7.43 (br s, 4H), 6.43 (t, J = 2.5 Hz, 2H), 6.08 (s, 2H) ppm. The data matches that reported in the literature (in THF-d_8).[7]

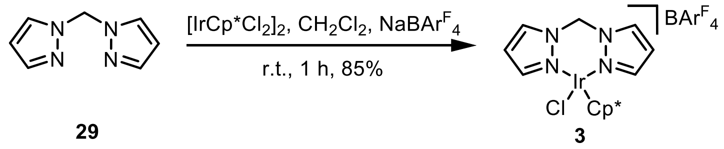

Complex **3** was synthesised according a modified literature procedure.[8] Di(1H-pyrazol-1-yl)methane **29** (148 mg, 1.0 mmol), $NaBAr^F_4$ (886 mg, 1.0 mmol) and $[IrCp^*Cl_2]_2$ (398 mg, 0.50 mmol) was dissolved in 30 mL of dry CH_2Cl_2 under a nitrogen atmosphere, and the mixture stirred at room temperature for 1 hour. The mixture was then filtered through Celite under a nitrogen atmosphere, and the solvent was removed *in vacuo*. The crude product was recrystallised from pentane-CH_2Cl_2 under air to afford the product as a light-yellow solid in 85% yield (117 mg). ^{1}H NMR (400 MHz, acetone-*d₆*): δ 8.25 (d, *J* = 2.4 Hz, 2H), 7.94 (d, *J* = 1.8 Hz, 2H), 7.79 (br s, 8H), 7.67 (br s, 4H), 7.28 (d, *J* = 14.5 Hz, 1H), 6.67 (t, *J* = 1.8 Hz, 2H), 6.20 (d, *J* = 14.5 Hz, 1H), 1.78 (s, 15H) ppm. The data matches that reported in the literature.[8]

Synthesis of the ligand frameworks SS 13, HS 14 and HH 17

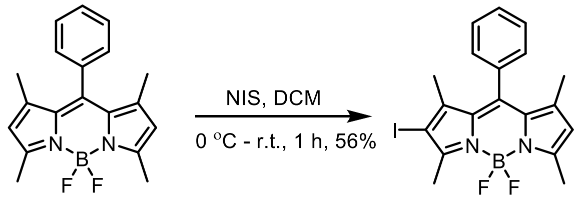

BDP **1** **10**

Compound **10** was synthesised according to a literature procedure.[9] Compound **1** (243 mg, 0.75 mmol) was dissolved in 200 mL of dry CH_2Cl_2 and cooled to 0 °C, followed by slow addition of *N*-iodosuccinimide (NIS, 112 mg, 0.5 mmol) in dry CH_2Cl_2 (100 mL) under a nitrogen atmosphere. After addition was completed, the mixture was stirred at room temperature for 1 hour. A saturated solution of NaCl (50 mL) was added to the reaction mixture, and the organic layer was separated and dried over anhydrous Na_2SO_4. The solvent was removed under reduced pressure. The crude product was purified by using flash column chromatography on silica gel using CH_2Cl_2-hexane (1:2, v/v, R_f = 0.4). The second band was collected to give the product **10** as an orange-red solid in 56% yield (188 mg). ^{1}H NMR (400 MHz, Chloroform-*d*) δ 7.64 – 7.40 (m, 3H), 7.28 - 7.25 (m, 2H), 6.04 (s, 1H), 2.63 (s, 3H), 2.57 (s, 3H), 1.38 (s, 6H) ppm. ^{11}B NMR (128 MHz, Chloroform-*d*) δ 0.67 (t, *J* = 32.8 Hz) ppm. ^{19}F NMR (376 MHz, Chloroform-*d*) δ -145.99 (q, *J* = 32.6 Hz) ppm. The data matches that reported in the literature.[9]

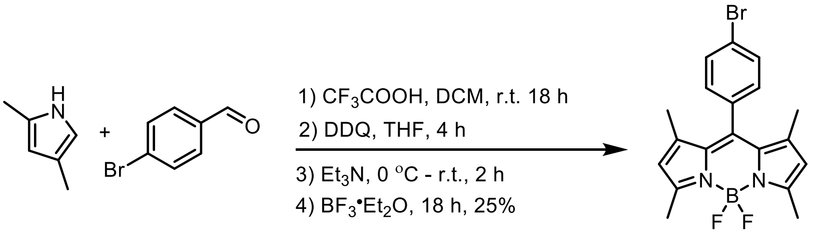

11

Compound **11** was synthesised following the same procedure as BDP **1** at the scale of 1.0 mmol of 4-bromo-benzaldehyde. The crude product was purified by flash column chromatography on silica gel eluting with CH_2Cl_2-hexane (1:1, v/v, R_f = 0.4) to give the product as a dark-orange solid in 25% yield (101 mg). ^{1}H NMR (400 MHz, Chloroform-*d*) δ 7.65 (d, *J* = 8.4 Hz, 2H), 7.18 (d, *J* = 8.4 Hz, 2H), 5.99 (s, 2H), 2.55 (s, 6H), 1.42 (s, 6H) ppm. ^{11}B NMR (128 MHz, Chloroform-*d*) δ 0.74 (t, *J* = 32.9 Hz) ppm. ^{19}F NMR (376 MHz, Chloroform-*d*) δ -146.29 (q, *J* = 32.9 Hz) ppm. The data matches that reported in the literature.[10]

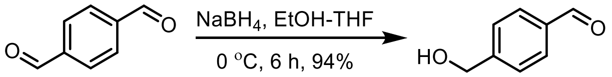

32

Compound **32** was synthesised according to a modified literature procedure.[11] $NaBH_4$ (0.85 g, 0.0185 mol) was added slowly to a solution of terephthalaldehyde (10.00 g, 0.075 mol) in a mixture of ethanol (100 mL) and THF (140 mL) at 0 °C under a nitrogen atmosphere, and stirred for 6 hours at 0 °C. HCl solution (2 M) was added to the reaction mixture to adjust to pH = 5. The majority of the solvent was removed under reduced pressure, water (150 mL) was added to the residue, and the product was extracted with EtOAc (100 ml x 3). The combined organic extracts were washed with brine and dried over anhydrous Na_2SO_4, and then concentrated to dryness. The crude product was obtained as a colourless oil in 94% yield (9.59 g), of >95% purity (by ^{1}H NMR), which was used in the synthesis of compound **31** without further purification. ^{1}H NMR (400 MHz, Chloroform-*d*) δ 9.99 (s, 1H), 7.87 (d,

J = 8.2 Hz, 2H), 7.53 (d, *J* = 8.2 Hz, 2H), 4.80 (d, *J* = 5.9 Hz, 2H), 2.10 (br s, 1H) ppm. The data matches that reported in the literature.[11]

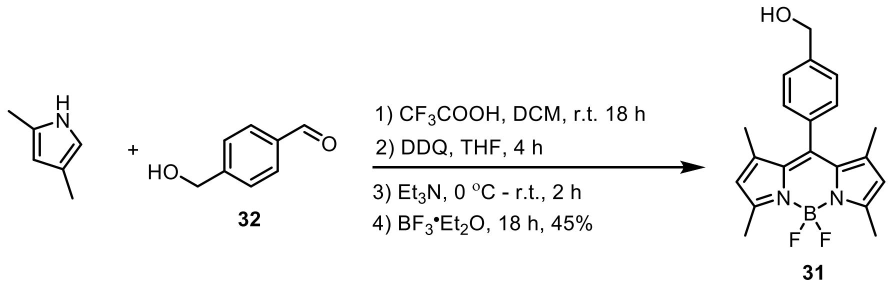

Compound **31** was synthesised following the same procedure as BDP **1**, at the scale of 1 mmol of 4-(hydroxymethyl)benzaldehyde **32**. The crude product was purified by flash column chromatography on silica gel eluting with CH_2Cl_2 (R_f = 0.2) to give the product as an orange solid in 45% yield (159 mg). ^{1}H NMR (400 MHz, Chloroform-*d*) δ 7.49 (d, *J* = 8.4 Hz, 2H), 7.27 (d, *J* = 8.4 Hz, 2H), 5.98 (s, 2H), 4.81 (s, 2H), 2.55 (s, 6H), 1.42 (s, 6H) ppm. The data matches that reported in the literature.[12]

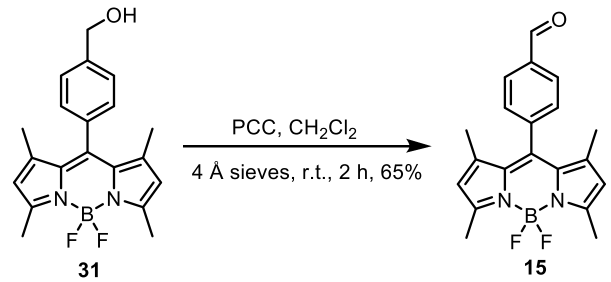

Compound **15** was synthesised according to a literature procedure.[12] Compound **31** (100 mg, 0.28 mmol) was dissolved in 50 mL of CH_2Cl_2 under a nitrogen atmosphere and pyridinium chlorochromate (PCC, 120 mg, 0.56 mmol) was added slowly, followed by 4 Å sieves (0.2 g). The reaction mixture was stirred at room temperature under a nitrogen atmosphere for 2 hours. The mixture was then filtered through Celite and purified by flash column chromatography on silica gel eluting with CH_2Cl_2 (R_f = 0.4) to give the product as an orange solid in 65% yield (64 mg). ^{1}H NMR (400 MHz, Chloroform-*d*) δ 10.11 (s, 1H), 8.03 (d, *J* = 8.1 Hz, 2H), 7.51 (d, *J* = 8.1 Hz, 2H), 6.00 (s, 2H), 2.56 (s, 6H), 1.35 (s, 6H) ppm. The data matches that reported in the literature.[12]

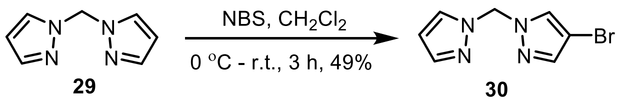

Di(1H-pyrazol-1-yl)methane **29** (3.00 g, 20.3 mmol) was dissolved in 25 mL of dry CH_2Cl_2 and cooled to 0 °C, followed by slow addition of a 25 mL dichloromethane solution of *N*-bromosuccinimide (NBS, 2.40 g, 13.6 mmol) under a nitrogen atmosphere. The reaction was warm to room temperature and reacted for 3 hours. A saturated solution of NaCl (50 mL) was added to the reaction mixture. The organic layer was separated and dried over anhydrous Na_2SO_4. The crude product was purified by flash column chromatography on silica gel eluting with hexane-EtOAc (5:1, v/v, R_f = 0.2) to give the product as a white solid in 49% yield (2.0 g, with respect to the limiting reagent NBS). ^{1}H NMR (400 MHz, Chloroform-*d*) δ 7.66 (s, 1H), 7.63 (dd, *J* = 2.5, 0.6 Hz, 1H), 7.57 (dd, *J* = 1.8, 0.6

Hz, 1H), 7.49 (s, 1H), 6.31 (dd, J = 2.5, 1.8 Hz, 1H), 6.24 (s, 2H) ppm. HRMS (ESI$^+$, MeOH) calculated for $[C_7H_7BrN_4+H]^+$: 226.99269 $[M+H]^+$, found: 226.99271.

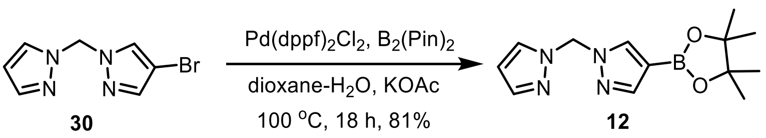

Compound **30** (0.80 g, 3.5 mmol), 4,4,4',4',5,5,5',5'-octamethyl-2,2'-bi(1,3,2-dioxaborolane) ($B_2(Pin)_2$, 1.35 g, 5.3 mmol), $Pd(dppf)_2Cl_2$ (0.26 g, 0.35 mmol) and KOAc (0.87 g, 8.9 mmol) were added to a round bottom flask, followed by addition of degassed 1,4-dioxane-H_2O (10 mL, 9:1, v/v) under a nitrogen atmosphere. The mixture was heated at 100 °C for 18 hours. The resulting mixture was filtered through Celite and washed thoroughly with EtOAc. The organic extracts were dried over anhydrous Na_2SO_4, and the solvent was removed under reduced pressure. The residual oily crude product was purified by flash column chromatography on silica gel eluting with hexane-EtOAc (2:1, v/v, R_f = 0.3, second band on column) to give the product as a white solid in 81% yield (786 mg). ^{1}H NMR (400 MHz, Chloroform-d) δ 7.91 (d, J = 0.6 Hz, 1H), 7.80 (d, J = 0.6 Hz, 1H), 7.64 (dd, J = 2.4, 0.6 Hz, 1H), 7.54 (dd, J = 1.8, 0.6 Hz, 1H), 6.32 – 6.26 (m, 3H), 1.29 (s, 12H) ppm. ^{13}C{^{1}H} NMR (101 MHz, Chloroform-d) δ 146.7, 141.0, 136.8, 129.8, 107.4, 83.6, 65.2, 24.9 ppm. HRMS (ESI$^+$, MeOH) calculated for $[C_{13}H_{19}BN_4O_2+H]^+$: 275.16741 $[M+H]^+$, found: 275.16756.

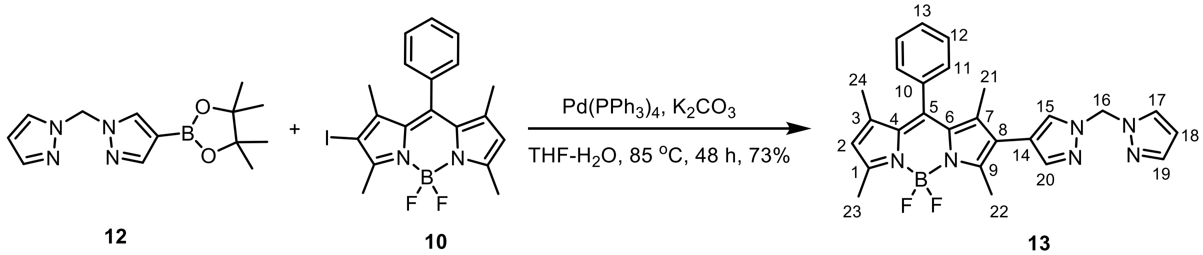

Compound **12** (89 mg, 0.33 mmol), monoiodo-BODIPY **10** (100 mg, 0.22 mmol), $Pd(PPh_3)_4$ (13 mg, 0.011 mmol) and K_2CO_3 (152 mg, 1.10 mmol) were added to the degassed solvent mixture of THF-H_2O (10 mL, 20:1, v/v) under a nitrogen atmosphere in a 25 mL glass pressure tube sealed with Teflon cap. The reaction mixture was stirred at room temperature for 5 minutes before heating up to 85 °C for 48 hours. The reaction mixture was filtered through Celite and washed thoroughly with CH_2Cl_2. The combined organic extracts were dried over anhydrous Na_2SO_4 and the solvent was removed under reduced pressure. The crude product was purified by flash column chromatography on silica gel eluting with MeOH-CH_2Cl_2 (50:1, v/v, R_f = 0.2) to give the product as a red solid. The solid was further purified by recrystallisation from methanol-pentane to afford the final product as a fluffy bright red solid in 73% yield (76 mg). ^{1}H NMR (400 MHz, Methylene Chloride-d_2) δ 7.65 (dd, J = 2.5, 0.7 Hz, 1H, **H**17), 7.54 (s, 1H, **H**20), 7.52 – 7.44 (m, 4H, **H**11, 13, 19), 7.41 (s, 1H, **H**15), 7.36 – 7.24 (m, 2H, **H**12), 6.27 (t, J = 2.5 Hz, 1H, **H**18), 6.26 (s, 2H, **H**16), 6.01 (s, 1H, **H**2), 2.50 (s, 3H, **H**23), 2.47 (s, 3H, **H**22), 1.37 (s, 3H, **H**24), 1.30 (s, 3H, **H**21) ppm. ^{13}C{^{1}H} NMR (101 MHz, Methylene Chloride-d_2) δ 156.6 (**C**1), 154.7 (**C**9), 144.5 (**C**3), 142.9 (**C**10), 141.6 (**C**15), 141.5 (**C**19), 140.2 (**C**5), 135.8 (**C**11), 132.4 (**C**4), 131.7 (**C**7), 130.5 (**C**6), 130.0 (**C**17), 129.8 (**C**13), 129.6 (**C**20), 128.9 (**C**12), 124.4 (**C**8), 122.2 (**C**2), 115.6(**C**14), 107.7 (**C**18), 66.24 (**C**16), 15.2 (**C**23), 15.0 (**C**22), 14.0 (**C**24), 13.3 (**C**21) ppm. ^{11}B NMR (128 MHz, Methylene Chloride-d_2) δ 0.77 (t, J = 32.9 Hz) ppm. ^{19}F NMR (376 MHz, Methylene Chloride-d_2) δ -146.02 (q, J = 32.7 Hz) ppm. HRMS (ESI$^+$, MeOH) calculated for $[C_{26}H_{25}BF_2N_6+Na]^+$: 493.2100 $[M+Na]^+$, found: 493.2094.

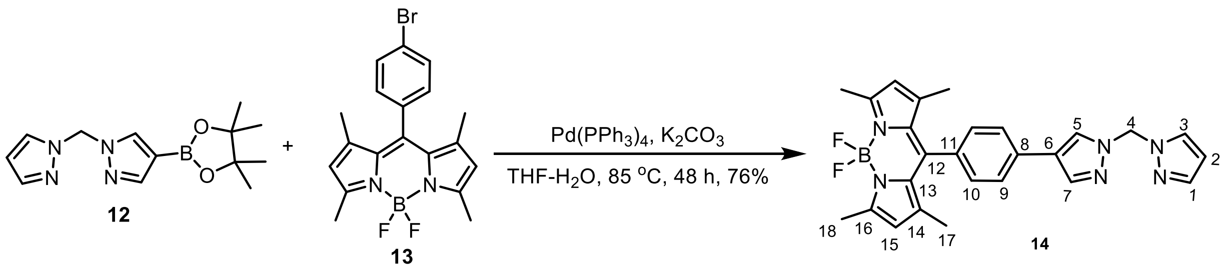

Compound **12** (89 mg, 0.22 mmol), bromo-BODIPY **13** (90 mg, 0.33 mmol), $Pd(PPh_3)_4$ (13 mg, 0.011 mmol) and K_2CO_3 (152 mg, 1.10 mmol) were added to the degassed solvent mixture of THF-H_2O (10 mL, 20:1, v/v) under a nitrogen atmosphere in a 100 mL Schlenk flask. The suspension was stirred at room temperature for 5 minutes before heating up to 85 °C for 48 hours. The reaction mixture was filtered through Celite and washed thoroughly with CH_2Cl_2. The combined organic extracts were dried over anhydrous Na_2SO_4 and the solvent was removed under reduced pressure. The crude product was purified by flash column chromatography on silica gel eluting with EtOAc-CH_2Cl_2 (1:3, v/v, R_f = 0.3) to give the product as an orange solid in 76% yield (118 mg). ^{1}H NMR (400 MHz, Methylene Chloride-*d*$_2$) δ 7.99 (s, 1H, **H**5), 7.86 (s, 1H, **H**7), 7.70 (dd, *J* = 2.5, 0.6 Hz, 1H, **H**3), 7.61 (d, *J* = 8.4 Hz, 2H, **H**9), 7.53 (dd, *J* = 1.8, 0.6 Hz, 1H, **H**1), 7.26 (d, *J* = 8.4 Hz, 2H, **H**10), 6.30 (m, 3H, **H**4 overlapped with **H**2), 5.99 (s, 2H, **H**15), 2.49 (s, 6H, **H**18), 1.43 (s, 6H, **H**17) ppm. ^{13}C{^{1}H} NMR (101 MHz, Methylene Chloride-*d*$_2$) δ 156.2 (**C**16), 144.2 (**C**13), 142.6 (**C**12), 141.6 (**C**1), 138.9 (**C**7), 133.9 (**C**8), 133.6 (**C**11), 132.2 (**C**14), 130.5(**C**3), 129.44 (**C**10), 127.4 (**C5**), 126.9 (**C**9), 124.3 (**C**6), 121.9 (**C**15), 107.8 (**C**2), 66.4 (**C**4), 15.1 (**C**17), 15.1 (**C**18) ppm. ^{11}B NMR (128 MHz, Methylene Chloride-*d*$_2$) δ 0.72 (t, *J* = 33.0 Hz) ppm. ^{19}F NMR (376 MHz, Methylene Chloride-*d*$_2$) δ -146.26 (q, *J* = 32.9 Hz) ppm. HRMS (ESI$^+$, MeOH) calculated for $[C_{26}H_{25}BF_2N_6+Na]^+$: 493.2100 $[M+Na]^+$, found: 493.2094.

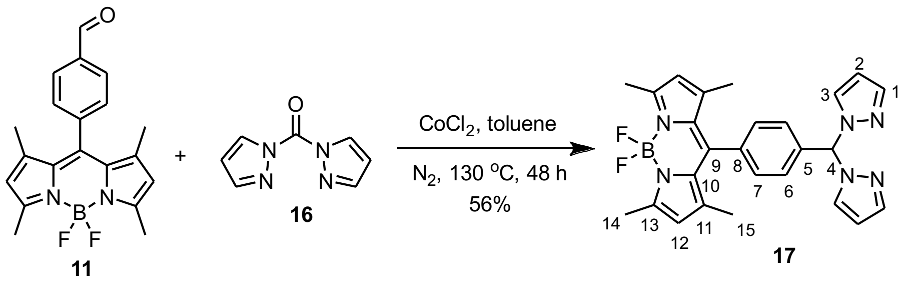

Compound **17** was synthesised according to a modified literature procedure.[13] Compound **11** (240 mg, 0.68 mmol), compound **16** (110 mg, 0.68 mmol) and $CoCl_2$ (18 mg, 0.14 mmol) and degassed anhydrous toluene (5.0 mL) was added to a 25 mL glass pressure tube, and the reaction mixture was bubbled with nitrogen for 10 minutes. The reaction mixture was then heated at 130 °C for 48 hours. The reaction mixture was concentrated to dryness under reduced pressure. The crude product was purified by flash column chromatography on silica gel eluting with MeOH-CH_2Cl_2 (50:1, v/v, R_f = 0.3) to give the product as an orange solid in 56% yield (180 mg). ^{1}H NMR (400 MHz, Methylene Chloride-*d*$_2$) δ 7.81 (s, 1H, **H**4), 7.61 (dd, *J* = 1.8, 0.6 Hz, 2H, **H**1), 7.52 (dd, *J* = 2.5, 0.6 Hz, 2H, **H**3), 7.33 (m, 2H, **H**6), 7.16 (m, 2H, **H**7), 6.35 (dd, *J* = 2.5, 1.8 Hz, 2H, **H**2), 6.01 (s, 2H, **H**12), 2.49 (s, 6H, **H**14), 1.40 (s, 6H, **H**15) ppm. ^{13}C{^{1}H} NMR (101 MHz, Methylene Chloride-*d*$_2$) δ 156.5 (**C**13), 144.0 (**C**10), 141.5 (**C**3), 138.3(**C**9), 136.8 (**C**5), 132.0 (**C**11), 130.4 (**C**8), 129.4 (**C**5), 128.8 (**C**7), 122.2 (**C**6), 122.1 (**C**12), 107.4 (**C**2), 78.2 (**C**4), 30.5 (**C**15), 15.1 (**C**14) ppm. ^{11}B NMR (128 MHz, Methylene Chloride-*d*$_2$) δ 0.70 (t, *J* = 32.9 Hz) ppm. ^{19}F NMR (376 MHz, Methylene Chloride-*d*$_2$) δ -146.27 (q, *J* = 32.9 Hz) ppm. HRMS (ESI$^+$, MeOH) calculated for $[C_{26}H_{25}BF_2N_6+H]^+$: 471.22476 $[M+H]^+$, found: 471.22749.

Synthesis of the bifunctional catalysts 4-9

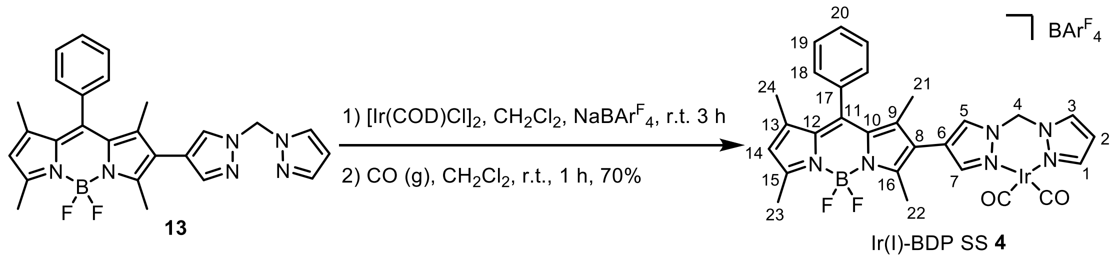

Complex **4** was synthesised by reacting ligand **13** (100 mg, 0.21 mmol) with $NaBAr^F_4$ (186 mg, 0.21 mmol) and $[Ir(COD)Cl]_2$ (71 mg, 0.105 mmol) in 30 mL of dry CH_2Cl_2 under a nitrogen atmosphere at room temperature for 6 hours. The resulting COD-intermediate was converted to the desired product under an absolute CO atmosphere (*via* three freeze-pump-thaw cycles, the flask then backfilled with nitrogen, and finally a balloon of CO gas was attached) and allowed to react at room temperature for 1 hour. The reaction mixture was filtered through Celite and the solvent was removed *in vacuo*. The crude product was then recrystallised with pentane-CH_2Cl_2 to afford the product as a red solid in 70% yield (238 mg). ^{1}H NMR (500 MHz, Methylene Chloride-d_2) δ 8.03 (s, 1H, **H**1), 7.93 (s, 1H, **H**3), 7.91 (s, 1H, **H**7), 7.81 (s, 1H, **H**5), 7.70 (br s, 8H, *ortho*-C**H** of BAr^F_4), 7.54 (br s, 4H, *para*-C**H** of BAr^F_4), 7.51 (m, 3H, **H**18 and **H**20), 7.29 (m, 2H, **H**19), 6.69 (t, J = 2.7 Hz, 1H, **H**2), 6.44 (s, 2H, **H**4), 6.12 (s, 1H, **H**14), 2.55 (s, 3H, **H**23), 2.48 (s, 3H, **H**22), 1.41 (s, 3H, **H**24), 1.30 (s, 3H, **H**21) ppm. ^{13}C{^{1}H} NMR (126 MHz, Methylene Chloride-d_2) δ 169.9 (**C**O), 162.5 (q, $^1J_{B\text{-}C}$ = 49.6 Hz, quart **C** *ipso* to B of BAr^F_4), 160.8 (**C**15), 150.9 (**C**16), 149.1 (**C**1), 148.5 (**C**7), 147.5 (**C**12), 143.6 (**C**11), 138.92 (**C**10), 136.0 (**C**3), 135.6 (*ortho*-**C**H of BAr^F_4), 135.2 (**C**17), 133.9 (**C**13), 134.4 (**C**5), 131.0 (**C**9), 130.3 (**C**18), 130.2 (**C**20), 129.6 (q, $^2J_{FC}$ = 30.2 Hz, quart **C** *ipso* to CF_3 of BAr^F_4), 125.4 (q, $^1J_{FC}$ = 273.4 Hz, CF_3 of BAr^F_4), 128.6 (**C**19), 123.9 (**C**14), 122.2 (**C**6), 118.3 (*para*-**C**H of BAr^F_4), 118.2 (**C**8), 111.0 (**C**2), 64.3 (**C**4), 15.5 (**C**23), 15.3 (**C**24), 13.6 (**C**22), 13.1 (**C**21) ppm. ^{11}B NMR (128 MHz, Methylene Chloride-d_2) δ 0.68 (t, J = 32.7 Hz), -6.67 ppm. ^{19}F NMR (376 MHz, Methylene Chloride-d_2) δ -62.85, -145.92 (q, J = 32.9 Hz) ppm. HRMS (ESI$^+$, MeOH) calculated for $[C_{60}H_{37}B_2F_{26}IrN_6O_2–BAr^F_4]^+$: 719.17296 $[M–BAr^F_4]^+$, found: 719.17277. Elemental analysis calculated for $C_{60}H_{37}B_2F_{26}IrN_6O_2$: C, 45.56; H, 2.36; N, 5.31%, found C, 44.71; H, 2.15; N, 5.28% (the minor deviations observed may be due to partial combustion of the sample during analysis). FTIR: ν_{CO} = 2098, 2035 (s) cm^{-1}.

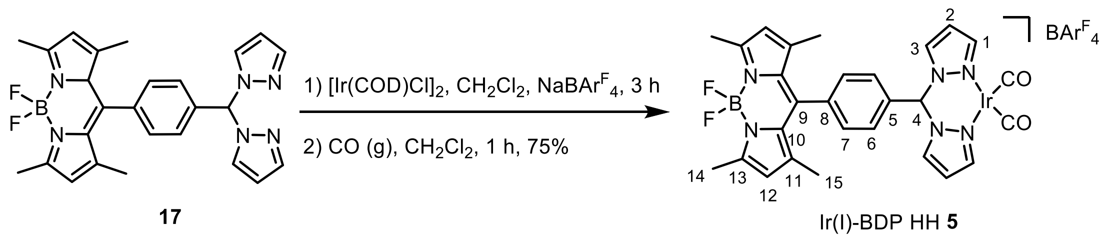

Complex **5** was synthesised by reacting ligand **17** (100 mg, 0.21 mmol) with $NaBAr^F_4$ (188 mg, 0.21 mmol) and $[Ir(COD)Cl]_2$ (71 mg, 0.105 mmol) in 30 mL of dry CH_2Cl_2 under a nitrogen atmosphere at room temperature for 3 hours. The resulted COD-intermediate was converted to desired product under absolute CO atmosphere (*via* three freeze-pump-thaw cycles, the flask then backfilled with nitrogen, and finally a balloon of CO gas was attached) and allowed to react at room temperature for 1 hour. The reaction mixture was filtered through Celite and the solvent was removed *in vacuo*. The crude product was then recrystallised with pentane-CH_2Cl_2 to afford the product as a red solid in 75% yield (249 mg). ^{1}H NMR (500 MHz, Methylene Chloride-d_2) δ 8.25 (d, J = 2.8 Hz, 2H, **H**3), 8.11 (d, J = 2.8 Hz, 2H, **H**1), 7.82 (s, 1H, **H**4), 7.70 (br s, 8H, *ortho*-C**H** of BAr^F_4), 7.54 (br s, 4H, *para*-C**H** of BAr^F_4), 7.48 (d, J = 8.5 Hz, 2H, **H**7), 6.81 (t, J = 2.8 Hz, 2H, **H**2), 6.56 (d, J = 8.5 Hz, 2H, **H**6), 6.03 (s, 2H, **H**12), 2.50 (s, 6H, **H**14), 1.42 (s, 6H, **H**15) ppm. ^{13}C{^{1}H} NMR (126 MHz, Methylene Chloride-d_2) δ 169.5 (**C**O), 162.6 (q, $^1J_{B\text{-}C}$ =

49.6 Hz, quart **C** *ipso* to B of BAr$^{F}_{4}$), 157.3 (**C**13), 149.6 (**C**1), 143.3 (**C**10), 139.7 (**C**9), 139.5 (**C**5), 137.0 (**C**3), 135.6 (*ortho*-**C**H of BAr$^{F}_{4}$), 134.6 (**C**8), 131.7 (**C**11), 131.1 (**C**7), 129.7 (q, $^{2}J_{FC}$ = 30.2 Hz, quart **C** *ipso* to CF_3 of BAr$^{F}_{4}$), 128.6 (q, $^{1}J_{FC}$ = 272.2 Hz, **C**F_3 of BAr$^{F}_{4}$), 126.7 (**C**6), 122.5 (**C**12), 118.3 (*para*-**C**H of BAr$^{F}_{4}$), 110.9 (**C**2), 77.0 (**C**4), 15.8 (**C**15), 15.2 (**C**14) ppm. ^{11}B NMR (160 MHz, Methylene Chloride-*d*$_2$) δ 0.68 (t, *J* = 32.6 Hz), -6.61 ppm. ^{19}F NMR (471 MHz, Methylene Chloride-*d*$_2$) δ -63.77, -147.25 (q, *J* = 32.1 Hz) ppm. HRMS (ESI$^+$, MeOH) calculated for $[C_{60}H_{37}B_2F_{26}IrN_6O_2–BAr^{F}_4]^+$: 719.17296 $[M–BAr^{F}_4]^+$, found: 719.17336. Elemental analysis calculated for $C_{60}H_{37}B_2F_{26}IrN_6O_2$: C, 45.56; H, 2.36; N, 5.31%, found C, 44.99; H, 2.76; N, 4.87% (the minor deviations observed may be due to partial combustion of the sample during analysis). FTIR: ν_{CO} = 2093, 2029 (s) cm^{-1}.

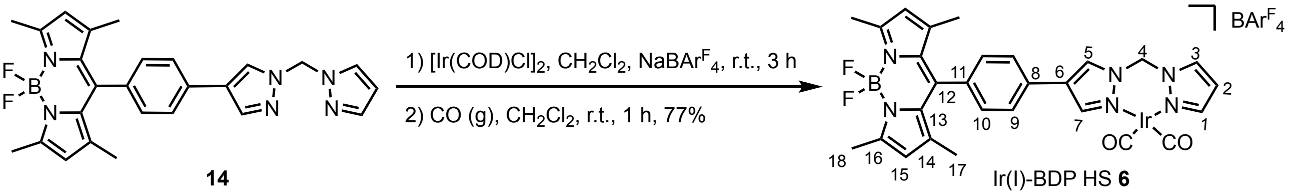

Complex **6** was synthesised by reacting ligand **14** (100 mg, 0.21 mmol) with NaBAr$^{F}_{4}$ (188 mg, 0.21 mmol) and $[Ir(COD)Cl]_2$ (71 mg, 0.105 mmol) in 30 mL of dry CH_2Cl_2 under a nitrogen atmosphere at room temperature for 3 hours. The resulting COD-intermediate was converted to the desired product under absolute CO atmosphere (*via* three freeze-pump-thaw cycles, the flask then backfilled with nitrogen, and finally a balloon of CO gas was attached) and allowed to react at room temperature for 1 hour. The reaction mixture was filtered through Celite and the solvent was removed *in vacuo*. The crude product was then recrystallised with pentane-CH_2Cl_2 to afford the product as a red solid in 77% yield (256 mg). ^{1}H NMR (500 MHz, Methylene Chloride-*d*$_2$) δ 8.33 (s, 1H, **H**7), 8.21 (s, 1H, **H**5), 8.05 (d, *J* = 2.5 Hz, 1H, **H**1), 7.95 (d, *J* = 2.5 Hz, 1H, **H**3), 7.70 (br s, 8H, *ortho*-C**H** of BAr$^{F}_{4}$), 7.63 (d, *J* = 8.1 Hz, 2H, **H**9), 7.53 (br s, 4H, *para*-C**H** of BAr$^{F}_{4}$), 7.45 (d, *J* = 8.1 Hz, 2H, **H**10), 6.70 (t, *J* = 2.5 Hz, 1H, **H**2), 6.50 (s, 2H, **H**4), 6.01 (s, 2H, **H**15), 2.50 (s, 6H, **H**18), 1.39 (s, 6H, **H**17) ppm. ^{13}C{^{1}H} NMR (126 MHz, Methylene Chloride-*d*$_2$) δ 169.8 (**C**O), 169.5 (**C**O), 162.5 (q, $^{1}J_{B\text{-}C}$ = 49.6 Hz, quart **C** *ipso* to B of BAr$^{F}_{4}$), 156.8 (**C**16), 149.2 (**C**1), 146.0 (**C**7), 143.7 (**C**13), 141.0 (**C**12), 137.1 (**C**8), 136.0 (**C**3), 135.6 (*ortho*-**C**H of BAr$^{F}_{4}$), 132.3 (**C**5), 132.0 (**C**14), 130.5 (**C**10), 129.6 (q, $^{2}J_{FC}$ = 30.2 Hz, quart **C** *ipso* to CF_3 of BAr$^{F}_{4}$), 128.8 (**C**11), 125.4 (q, $^{1}J_{FC}$ = 273.4 Hz, **C**F_3 of BAr$^{F}_{4}$), 127.7 (**C**9), 127.2 (**C**6), 122.2 (**C**15), 118.3 (*para*-**C**H of BAr$^{F}_{4}$), 111.1 (**C**2), 64.4 (**C**4), 15.1 (**C**17 and **C**18) ppm. ^{11}B NMR (128 MHz, Methylene Chloride-*d*$_2$) δ 0.64 (t, *J* = 32.7 Hz), -6.67 ppm. ^{19}F NMR (376 MHz, Methylene Chloride-*d*$_2$) δ -62.86, -146.28 (q, *J* = 32.7 Hz) ppm. HRMS (ESI$^+$, MeOH) calculated for $[C_{60}H_{37}B_2F_{26}IrN_6O_2–BAr^{F}_4]^+$: 719.17296 $[M–BAr^{F}_4]^+$, found: 719.17329. Elemental analysis calculated for $C_{60}H_{37}B_2F_{26}IrN_6O_2$: C, 45.56; H, 2.36; N, 5.31%, found C, 45.65; H, 2.12; N, 5.06%. FTIR: ν_{CO} = 2091, 2028 (s) cm^{-1}.

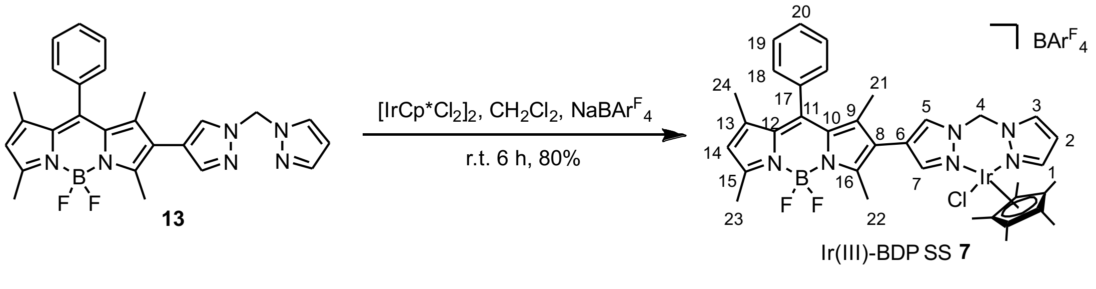

Complex **7** was synthesised by reacting ligand **13** (47 mg, 0.10 mmol) with NaBAr$^{F}_{4}$ (88 mg, 0.10 mmol) and $[IrCp^*Cl_2]_2$ (40 mg, 0.05 mmol) in 15 mL of dry CH_2Cl_2 under a nitrogen atmosphere at room temperature for 6 hours. The reaction mixture was filtered through Celite under a nitrogen atmosphere and the solvent was removed *in vacuo*. The crude product was then recrystallised from pentane-CH_2Cl_2 to afford the product as a red

solid in 80% yield (136 mg). ^{1}H NMR (500 MHz, Methylene Chloride-d_2) δ 7.88 (d, J = 2.6 Hz, 1H, **H**1), 7.78 (s, 1H, **H**7), 7.75 (d, J = 2.6 Hz, 1H, **H**3), 7.70 (br s, 8H, *ortho*-C**H** of BAr$^F{}_4$), 7.65 (s, 1H, **H**5), 7.54 (br s, 4H, *para*-C**H** of BAr$^F{}_4$), 7.52 – 7.46 (m, 3H, **H**18, 20), 7.29 (m, 2H, **H**19), 6.60 (t, J = 2.6 Hz, 1H, **H**2), 6.48 (d, J = 14.1 Hz, 1H, **H**4), 6.10 (s, 1H, **H**14), 5.80 (d, J = 14.1 Hz, 1H, **H**4), 2.53 (s, 3H, **H**23), 2.48 (s, 3H, **H**22), 1.63 (s, 15H, C**H**$_3$ of Cp*), 1.40 (s, 3H, **H**24), 1.30 (s, 3H, **H**21) ppm. ^{13}C{^{1}H} NMR (126 MHz, Methylene Chloride-d_2) δ 162.1 (q, $^1J_{B\text{-}C}$ = 49.5 Hz, quart **C** *ipso* to B of BAr$^F{}_4$), 159.3 (**C**15), 151.3 (**C**16), 146.6 (**C**5), 146.5 (**C**12), 146.4 (**C**3), 143.1 (**C**11), 138.8 (**C**10), 135.2 (*ortho*-CH of BAr$^F{}_4$), 134.9 (**C**17), 134.5 (**C**1), 133.1 (**C**13) 132.8 (**C**7), 130.8 (**C**9), 129.8 (**C**18), 129.7 (**C**20), 129.2 (q, $^2J_{FC}$ = 32.9 Hz, quart **C** *ipso* to CF$_3$ of BAr$^F{}_4$), 128.2 (**C**19), 125.0 (q, $^1J_{FC}$ = 272.2 Hz, **C**F$_3$ of BAr$^F{}_4$), 123.1 (**C**14), 119.7 (**C**8), 118.5 (**C**6), 117.9 (*para*-**C**H of BAr$^F{}_4$), 110.2 (**C**2), 89.8 (quart **C** of Cp*), 63.5 (**C**4), 15.0 (**C**23), 14.8 (**C**24), 13.3 (**C**22), 12.8 (**C**21), 9.4 (**C**H$_3$ of Cp*) ppm. ^{11}B NMR (160 MHz, Methylene Chloride-d_2) δ 0.74 (t, J = 32.6 Hz), -6.61 ppm. ^{19}F NMR (471 MHz, Methylene Chloride-d_2) δ -63.82, -146.92 (q, J = 32.2 Hz) ppm. HRMS (ESI$^+$, MeOH) calculated for [$C_{68}H_{52}B_2ClF_{26}IrN_6$–BAr$^F{}_4$]$^+$: 833.26934 [M–BAr$^F{}_4$]$^+$, found: 833.26919. Elemental analysis calculated for $C_{68}H_{52}B_2ClF_{26}IrN_6$: C, 48.14; H, 3.09; N, 4.95%, found C, 48.52; H, 3.31; N, 5.29%.

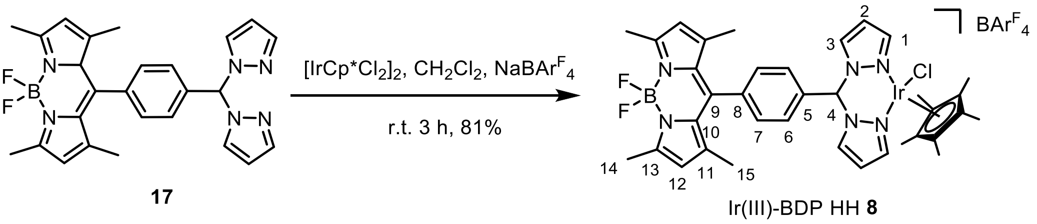

Complex **8** was synthesised by reacting ligand **17** (47 mg, 0.10 mmol) with NaBAr$^F{}_4$ (88 mg, 0.10 mmol) and [IrCp*Cl$_2$]$_2$ (40 mg, 0.05 mmol) in 15 mL of dry CH_2Cl_2 under a nitrogen atmosphere at room temperature for 3 hours. The reaction mixture was filtered through Celite under a nitrogen atmosphere and the solvent was removed *in vacuo*. The crude product was then recrystallised from pentane-CH_2Cl_2 to afford the product as an orange solid in 81% yield (137 mg). ^{1}H NMR (500 MHz, Methylene Chloride-d_2) δ 7.88 – 7.84 (m, 4H, **H**3 and **H**6), 7.82 (m, 2H, **H**7), 7.69 (br s, 8H, *ortho*-C**H** of BAr$^F{}_4$), 7.53 (br s, 4H, *para*-C**H** of BAr$^F{}_4$), 7.41 (dd, J = 2.6, 0.8 Hz, 2H, **H**1), 6.88 (s, 1H, **H**4), 6.60 (t, J = 2.6 Hz, 2H, **H**2), 6.08 (s, 2H, **H**12), 2.54 (s, 6H, **H**14), 1.68 (s, 15H, C**H**$_3$ of Cp*), 1.46 (s, 6H, **H**15) ppm. ^{13}C{^{1}H} NMR (126 MHz, Methylene Chloride-d_2) δ 162.5 (q, $^1J_{B\text{-}C}$ = 49.6 Hz, quart **C** *ipso* to B of BAr$^F{}_4$), 157.7 (**C**13), 147.5 (**C**1), 143.0 (**C**10), 142.1 (**C**8), 138.7 (**C**9), 135.6 (*ortho*-**C**H of BAr$^F{}_4$), 134.3 (**C**3), 132.4 (**C**7), 132.2 (**C**6), 131.6 (**C**11), 129.6 (q, $^2J_{FC}$ = 30.2 Hz, quart **C** *ipso* to CF$_3$ of BAr$^F{}_4$), 127.1 (**C**5), 125.4 (q, $^1J_{FC}$ = 273.4 Hz, **C**F$_3$ of BAr$^F{}_4$), 122.8 (**C**12), 118.3 (*para*-CH of BAr$^F{}_4$), 110.4 (**C**2), 90.3 (quart **C** of Cp*), 78.2 (**C**4), 15.4 (**C**14), 15.2 (**C**15), 9.8 (**C**H$_3$ of Cp*) ppm. ^{11}B NMR (128 MHz, Methylene Chloride-d_2) δ 0.71 (t, J = 32.5 Hz), -6.62 ppm. ^{19}F NMR (376 MHz, Methylene Chloride-d_2) δ -62.83, -146.24 (q, J = 32.2 Hz) ppm. HRMS (ESI$^+$, MeOH) calculated for [$C_{68}H_{52}B_2ClF_{26}IrN_6$–BAr$^F{}_4$]$^+$: 833.26934 [M–BAr$^F{}_4$]$^+$, found: 833.26879. Elemental analysis calculated for $C_{68}H_{52}B_2ClF_{26}IrN_6$: C, 48.14; H, 3.09; N, 4.95%, found C, 48.47, H, 2.34; N, 5.17% (the minor deviations observed may be due to partial combustion of the sample during analysis).

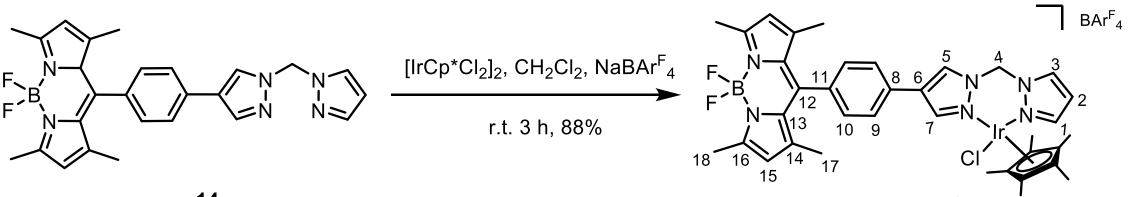

14 Ir(III)-BDP HS **9**

Complex **9** was synthesised by reacting ligand **14** (24 mg, 0.05 mmol) with $NaBAr^F_4$ (44 mg, 0.05 mmol) and $[IrCp^*Cl_2]_2$ (20 mg, 0.025 mmol) in 10 mL of dry CH_2Cl_2 under a nitrogen atmosphere at room temperature for 3 hours. The reaction mixture was filtered through Celite under a nitrogen atmosphere and the solvent was removed *in vacuo*. The crude product was then recrystallised from pentane-CH_2Cl_2 to afford the product as an orange solid in 88% yield (75 mg). ^1H NMR (400 MHz, Methylene Chloride-d_2) δ 8.12 (s, 1H, , **H**7), 8.10 (s, Hz, 1H, **H**5), 7.85 (d, J = 2.6 Hz, 1H, **H**1), 7.79 (d, J = 2.6 Hz, 1H, **H**3), 7.70 (br s, 8H, *ortho*-C**H** of BAr^F_4), 7.62 (m, 2H, **H**10), 7.54 (br s, 4H, *para*-C**H** of BAr^F_4), 7.39 (d, J = 8.3 Hz, 2H, **H**9), 6.63 (t, J = 2.6 Hz, 1H, **H**2), 6.36 (d, J = 14.3 Hz, 1H, **H**4), 6.01 (s, 2H, **H**15), 5.88 (d, J = 14.2 Hz, 1H, **H**4), 2.50 (s, 6H, **H**18), 1.69 (s, 15H, CH_3 of Cp*), 1.39 (s, 6H, **H**17) ppm. $^{13}C\{^1H\}$ NMR (101 MHz, Methylene Chloride-d_2) δ 162.50 (q, $^1J_{B\text{-}C}$ = 49.6 Hz, quart **C** *ipso* to B of BAr^F_4), 156.7 (**C**16), 147.2 (**C**3), 144.3 (**C**5), 143.9 (**C**13), 141.4 (**C**8), 136.3 (**C**11), 135.6 (*ortho*-**C**H of BAr^F_4), 134.8 (**C**1), 132.0 (**C**14), 131.2 (**C**7), 130.1 (**C**9), 129.6 (q, $^2J_{FC}$ = 30.2 Hz, quart **C** *ipso* to CF_3 of BAr^F_4), 127.5 (**C**10), 126.9 (**C**6), 126.8 (**C**12), 125.4 (q, $^1J_{FC}$ = 273.4 Hz, **C**F_3 of BAr^F_4), 122.2 (**C**15), 118.3 (*para*-**C**H of BAr^F_4), 110.8 (**C**2), 90.3 (quart **C** of Cp*), 64.1 (**C**4), 15.2 (**C**17 and **C**18), 9.8 (**C**H_3 of Cp*) ppm. ^{11}B NMR (128 MHz, Methylene Chloride-d_2) δ 0.65 (t, J = 32.7 Hz), -6.67 ppm. ^{19}F NMR (376 MHz, Methylene Chloride-d_2) δ -62.81, -146.22 (q, J = 32.3 Hz) ppm. HRMS (ESI^+, MeOH) calculated for $[C_{68}H_{52}B_2ClF_{26}IrN_6–BAr^F_4]^+$: 833.26934 $[M–BAr^F_4]^+$, found: 833.26923. Elemental analysis calculated for $C_{68}H_{52}B_2ClF_{26}IrN_6$: C, 48.14; H, 3.09; N, 4.95%, found C, 48.09; H, 2.74; N, 4.84%.

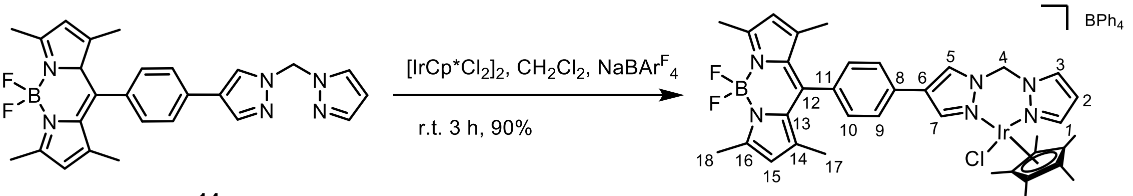

14 [Ir(III)-BDP HS][BPh_4]

[Ir(III)-BDP HS][BPh_4] was synthesised according to the same procedure above on the same reaction scale. The crude product was then recrystallised from pentane-CH_2Cl_2 to afford the product as a yellow solid in 90% yield (77 mg). ^1H NMR (400 MHz, Methylene Chloride-d_2) δ 7.87 (s, 1H, **H**7), 7.57 (d, J = 2.6 Hz, 1H, **H**1), 7.53 (d, J = 8.2 Hz, 2H, **H**9), 7.45 (br s, 8H, *ortho*-C**H** of BPh_4), 7.36 (m, 2H, **H**10), 7.05 (t, J = 7.4 Hz, 9H, *meta*-C**H** of BPh_4 overlapped with **H**3), 6.95 – 6.86 (m, 5H, *para*-C**H** of BPh_4 overlapped with **H**5), 6.39 (t, J = 2.6 Hz, 1H, **H**2), 6.02 (s, 2H, **H**15), 5.36 (d, J = 14.6 Hz, 1H, **H**4), 4.86 (d, J = 14.6 Hz, 1H, **H**4), 2.51 (s, 6H, **H**18), 1.62 (s, 15H, CH_3 of Cp*), 1.44 (s, 6H, **H**17). $^{13}C\{^1H\}$ NMR (126 MHz, Methylene Chloride- d_2) δ 164.3 (q, $^1J_{BC}$ = 49.3 Hz, quart **C** *ipso* to B of BPh_4), 156.4 (**C**16), 145.8 (**C**1), 144.0 (**C**13), 142.8 (**C**7), 141.9 (**C**8), 136.8 (br s, *ortho*-**C**H of BPh_4), 135.6 (**C**3), 135.4 (**C**12), 132.2 (**C**5), 132.1 (**C**14), 130.7 (**C**11), 129.8 (**C**10), 127.5 (**C**9), 127.0 (q, $^4J_{BC}$ = 2.8 Hz, *meta*-**C**H of BPh_4), 125.7 (**C**6), 123.4 (*para*-**C**H of BPh_4), 122.1 (**C**15), 109.7 (**C**2), 89.9 (quart **C** of Cp*), 63.1 (**C**4), 15.2 (**C**17), 15.1 (**C**18), 9.8 (**C**H_3 of Cp*). ^{11}B NMR (128 MHz, Methylene Chloride-d_2) δ 0.72 (t, J = 32.9 Hz), -6.58. ^{19}F NMR (376 MHz, Methylene Chloride-d_2) δ -146.24 (q, J = 32.4 Hz). The molecular structure was determined using X-ray diffraction, and this complex was not used in further studies.

Synthesis of substrate 25

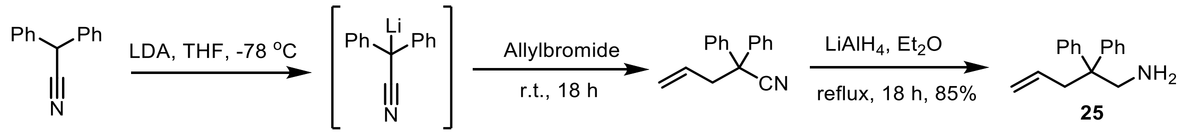

Compound **25** was synthesised according to a literature procedure with slight modifications.[14] To a suspension of lithium diisopropylamide (LDA, 1 M in THF, 46 mL, 46 mmol) in THF at -78 °C was added to 2,2-diphenylacetonitrile (8.05 g, 42 mmol). The reaction was stirred for 30 minutes at -78 °C and warmed to room temperature, allyl bromide (4.3 mL, 50 mmol) was added *via* syringe and the reaction mixture was stirred for 18 hours. The reaction was cooled to 0 °C and quenched with H_2O (50 mL). The mixture was then extracted with diethyl ether (3 x 100 mL) and dried over anhydrous $MgSO_4$ and the solvent was removed under reduced pressure. The obtained light yellow oily crude product was used in the next step without further purification. To a suspension of $LiAlH_4$ (6.38 g, 168 mmol) in dry diethyl ether (100 mL) under a nitrogen atmosphere was added the above nitrile in dry diethyl ether (30 mL) slowly. The mixture was stirred for 30 minutes at room temperature and then heated at reflux for 18 hours. A mixture of THF-H_2O (100 mL, 95:5, v/v) was added to the suspension by slow addition at 0 °C and the stirred at room temperature for 1 hour. The solid was filtered and washed thoroughly with diethyl ether. The combined organic extracts were dried over anhydrous $MgSO_4$ and the solvent removed under reduced pressure, and the crude product was purified by distillation at 150 °C *in vacuo* (0.30 mbar) to give the product as a colourless oil in 85% yield (8.46 g) and stored under a nitrogen atmosphere. ^{1}H NMR (400 MHz, Chloroform-*d*) δ 7.35 – 7.29 (m, 4H), 7.25 – 7.17 (m, 6H), 5.42 (ddt, J = 17.0, 11.0, 7.0 Hz, 1H), 5.07 (d, *J* = 17.0 Hz, 1H), 4.99 (d, *J* = 11.0 Hz, 1H), 3.35 (s, 2H), 2.95 (d, *J* = 7.0 Hz, 2H), 0.78 (br s, 2H) ppm. ^{13}C{^{1}H} NMR (101 MHz, Chloroform-*d*) δ 146.4, 134.8, 128.3, 128.2, 126.2, 117.8, 51.5, 48.7, 41.3 ppm. The data matches that reported in the literature.[14]

Variable temperature ^{1}H NMR experiments for complex 4

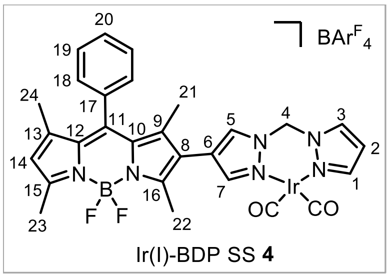

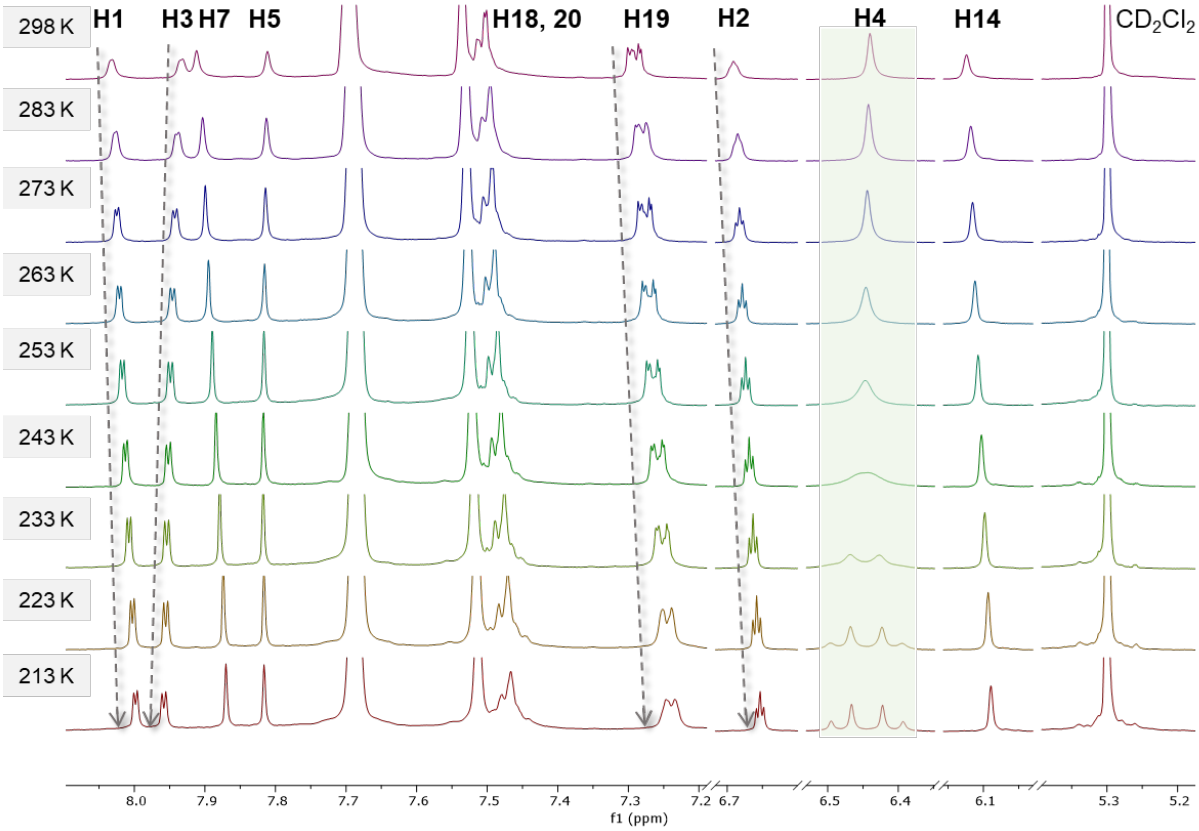

Figure S1: Stacked ^{1}H NMR spectra of Ir(I)-BDP SS **4** at temperatures ranging from 213 K to 298 K. Clear trends in chemical shifts were observed on changing temperature, with the bridging CH_2 unit (H**4**) resolving into a quartet below 223 K.

X-ray Crystallographic Data

Table S1: Crystallographic data for compounds HH **17**, Ir(I)-BDP HH **5**, HS **14** and [Ir(III)-BDP HS][BPh_4].

Complex	HH **17**	Ir(I)-BDP HH **5**	HS **14**	[Ir(III)-BDP HS][BPh_4]
CCDC number	1955143	1955144	1955141	1955142
Empirical formula	$C_{26}H_{25}BF_2N_6$	$C_{61.88}H_{40.12}B_2F_{26}IrN_6O_2$	$C_{26}H_{25}BF_2N_6$	$C_{60.5}H_{61}B_2Cl_2F_2IrN_6$
Formula weight	470.35	1607.44	470.33	1194.87
Temperature/K	99	99	150	150
Crystal system	monoclinic	triclinic	monoclinic	monoclinic
Space group	C2/c	P-1	C2/c	$P2_1/c$
a/Å	26.308(6)	18.4114(15)	28.780(3)	16.4813(8)
b/Å	7.8235(16)	18.6821(19)	10.3596(9)	27.5680(13)
c/Å	24.123(5)	22.004(2)	15.8174(13)	12.5577(7)
α/°	90	93.733(4)	90	90
β/°	112.783(14)	105.248(4)	90.308(5)	104.255(2)
γ/°	90	113.314(3)	90	90
Volume/$Å^3$	4577.6(17)	6582.4(11)	4715.9(7)	5530.0(5)
Z	8	4	8	4
$\rho_{calc}g/cm^3$	1.3648	1.622	1.325	1.435
μ/mm^{-1}	0.095	2.149	0.092	2.562
F(000)	1968.9	3162	1968	2420
Crystal size/mm^3	N/A × N/A × N/A	0.168 × 0.052 × 0.032	0.256 × 0.152 × 0.142	0.338 × 0.267 × 0.114
Radiation	Mo Kα (λ = 0.71073)	MoKα (λ = 0.71073)	MoKα (λ = 0.71073)	MoKα (λ = 0.71073)
2Θ range for data collection/°	5.48 to 50	4.434 to 55.238	4.904 to 52.912	4.464 to 54.26
Index ranges	-34 ≤ h ≤ 34, -10 ≤ k ≤ 10, -31 ≤ l ≤ 31	-23 ≤ h ≤ 23, -24 ≤ k ≤ 24, -28 ≤ l ≤ 28	-36 ≤ h ≤ 35, -12 ≤ k ≤ 12, -19 ≤ l ≤ 19	-21 ≤ h ≤ 21, -35 ≤ k ≤ 35, -16 ≤ l ≤ 14
Reflections collected	69657	445992	58472	76548
Independent reflections	4031 [R_{int} = 0.2140, R_{sigma} = 0.0922]	30333 [R_{int} = 0.1219, R_{sigma} = 0.0446]	4850 [R_{int} = 0.0463, R_{sigma} = 0.0232]	12187 [R_{int} = 0.0757, R_{sigma} = 0.0541]
Data/restraints/ parameters	4031/0/320	30333/305/1785	4850/0/320	12187/19/712
Goodness-of-fit on F^2	1.052	1.043	1.066	1.055
Final R indexes [I>=2σ (I)]	R_1 = 0.0585, wR_2 = 0.1233	R_1 = 0.0527, wR_2 = 0.1287	R_1 = 0.0395, wR_2 = 0.1003	R_1 = 0.0356, wR_2 = 0.0830
Final R indexes [all data]	R_1 = 0.0978, wR_2 = 0.1427	R_1 = 0.0767, wR_2 = 0.1440	R_1 = 0.0538, wR_2 = 0.1108	R_1 = 0.0491, wR_2 = 0.0899
Largest diff. peak/hole / e $Å^{-3}$	0.42/-0.45	6.52/-3.72	0.54/-0.24	2.30/-1.17

Table S2: Summary of selected distances between atoms and bond angles for compounds HH **17**, Ir(I)-BDP HH **5**, HS **14** and [Ir(III)-BDP HS][BPh_4].

Bond lengths (Å)	HH **17**	Ir(I)-BDP HH **5**	HS **14**	[Ir(III)-BDP HS][BPh_4]
Ir-N	-	Ir1-N2: 2.076(4)<br>Ir1-N4: 2.081(4)	-	Ir1-N3: 2.098(3)<br>Ir1-N6: 2.099(3)
Ir-C	-	Ir1-C2: 1.858(6)<br>Ir1-C1: 1.856(7)	-	Ir1-C1Cp: 2.177(4)
B-N	B1-N1: 1.540(4)<br>B1-N2: 1.543(5)	B1-N6: 1.535(6)<br>B1-N5: 1.549(10)	B1-N1: 1.537(2)<br>B1-N2: 1.542(2)	B1-N1: 1.533(6)<br>B1-N2: 1.534(7)
B-F	B1-F2: 1.394(4)<br>B1-F1: 1.361(4)	B1-F2: 1.379(8)<br>B1-F1: 1.401(7)	B1-F2: 1.3936(19)<br>B1-F1: 1.3953(19)	B1-F1: 1.389(5)<br>B1-F2: 1.377(7)
Ir-B	-	B1-Ir1: 8.749 (7)	-	B1-Ir1: 12.870(6)
**Bond angles (°)**	**HH 17**	**Ir(I)-BDP HH 5**	**HS 14**	**Ir(III)-BDP HS][BPh_4]**
N-Ir-N	-	N2-Ir1-N4: 86.52(17)	-	83.96(11)
C-Ir-C	-	C1-Ir1-C2: 89.0(3)	-	-
C-Ir-N	-	C2-Ir1-N2: 91.7(2)<br>C1-Ir1-N4: 92.8(2)	-	-
N-B-N	N1-B1-N2: 106.5(2)	N6-B1-N5: 107.2(5)	N1-B1-N2: 106.82(12)	N1-B1-N2: 107.2(4)
F-B-F	F1-B1-F2: 109.7(2)	F2-B1-F1: 108.9(5)	F1-B1-F2: 108.30(13)	F1-B1-F2: 109.9(4)
F-B-N	F2-B1-N1: 111.2(3)<br>F1-B1-N2: 109.3(3)<br>F2-B1-N2: 110.5(3)<br>F1-B1-N2: 109.6(3)	F2-B1-N6: 111.1(5)<br>F1-B1-N5: 109.1(5)<br>F2-B1-N5: 110.7(5)<br>F1-B1-N6: 109.8(4)	F1-B1-N1: 110.23(13)<br>F2-B1-N2: 110.65(12)<br>F1-B1-N2: 110.65(12)<br>F2-B1-N1: 110.20(13)	F1-B1-N1: 109.6(4)<br>F2-B1-N2: 111.0(4)<br>F1-B1-N2: 108.6(4)<br>F2-B1-N1: 110.0(4)
N-C-N	N4-C20-N6: 110.5(2)	N1-C9-N2: 109.0(5)	N1-C20-N6: 110.5(2)	N4-C20-N6: 110.0(4)

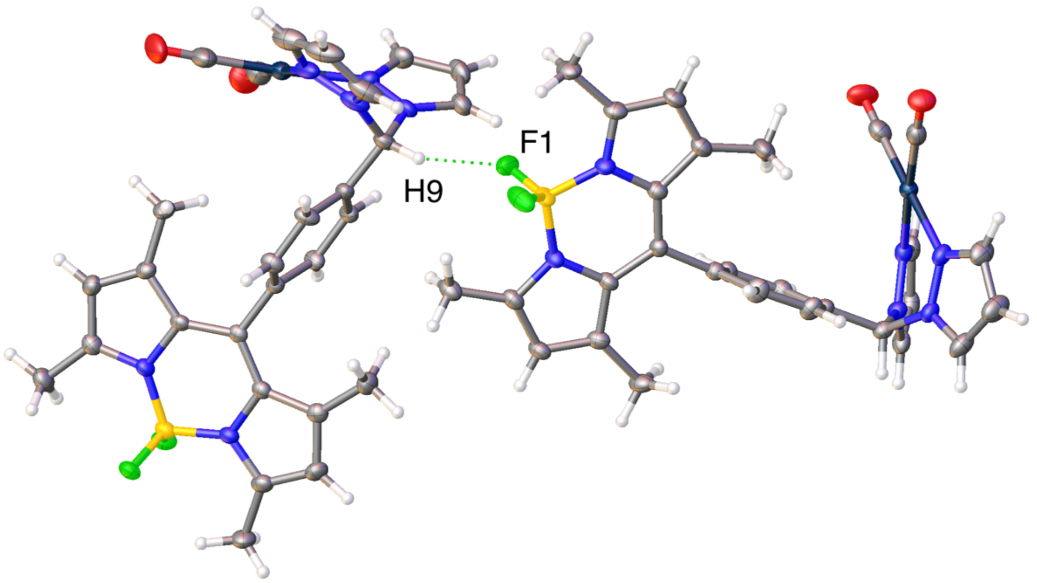

Figure S2: The molecular structure of Ir(I)-BDP HH **5**, indicating the H-F bond between two Ir(I)-BDP HH **5** complexes, with a H-F bond length 2.099 Å. The figure was analysed and exported by using the OLEX 2 program.[15]

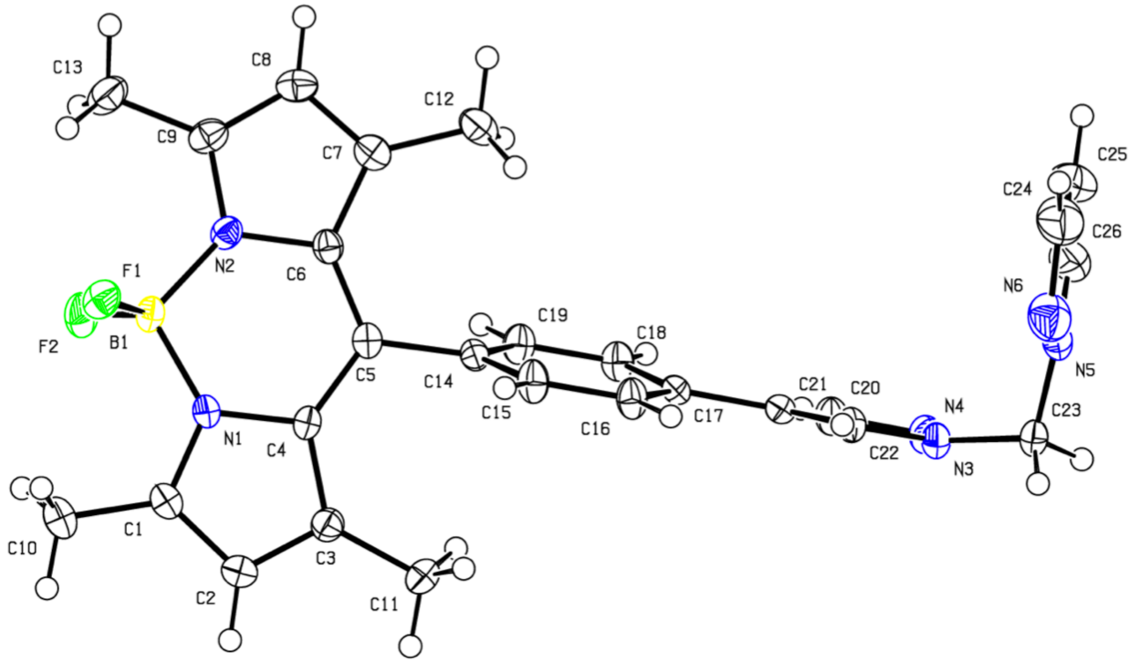

Figure S3: ORTEP[16] depiction of the molecular structure of HS **14**.

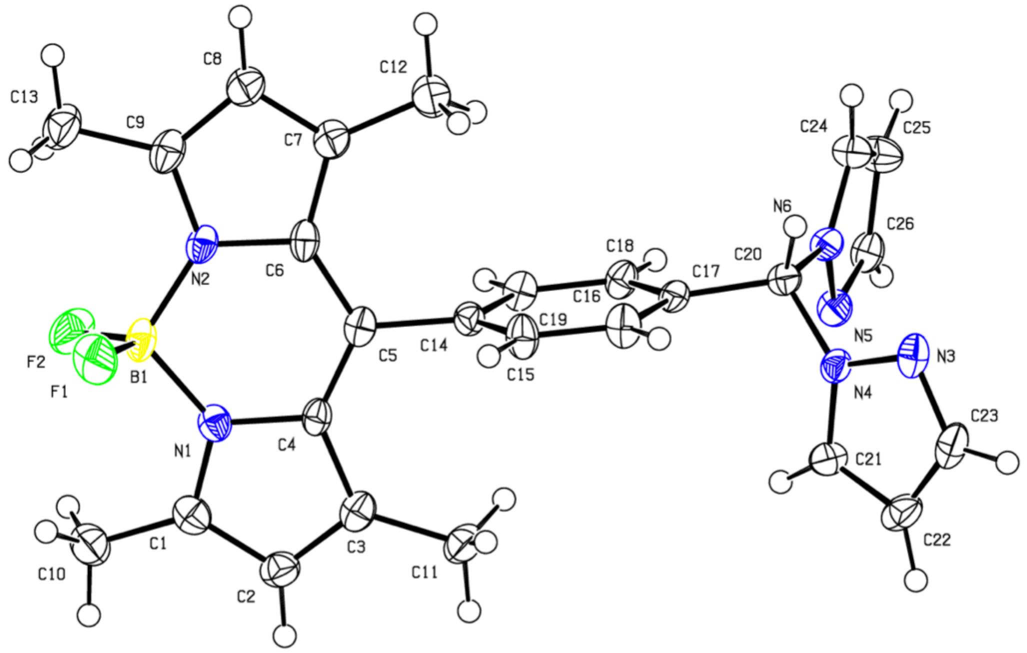

Figure S4: ORTEP[16] depiction of the molecular structure of HH **17**.

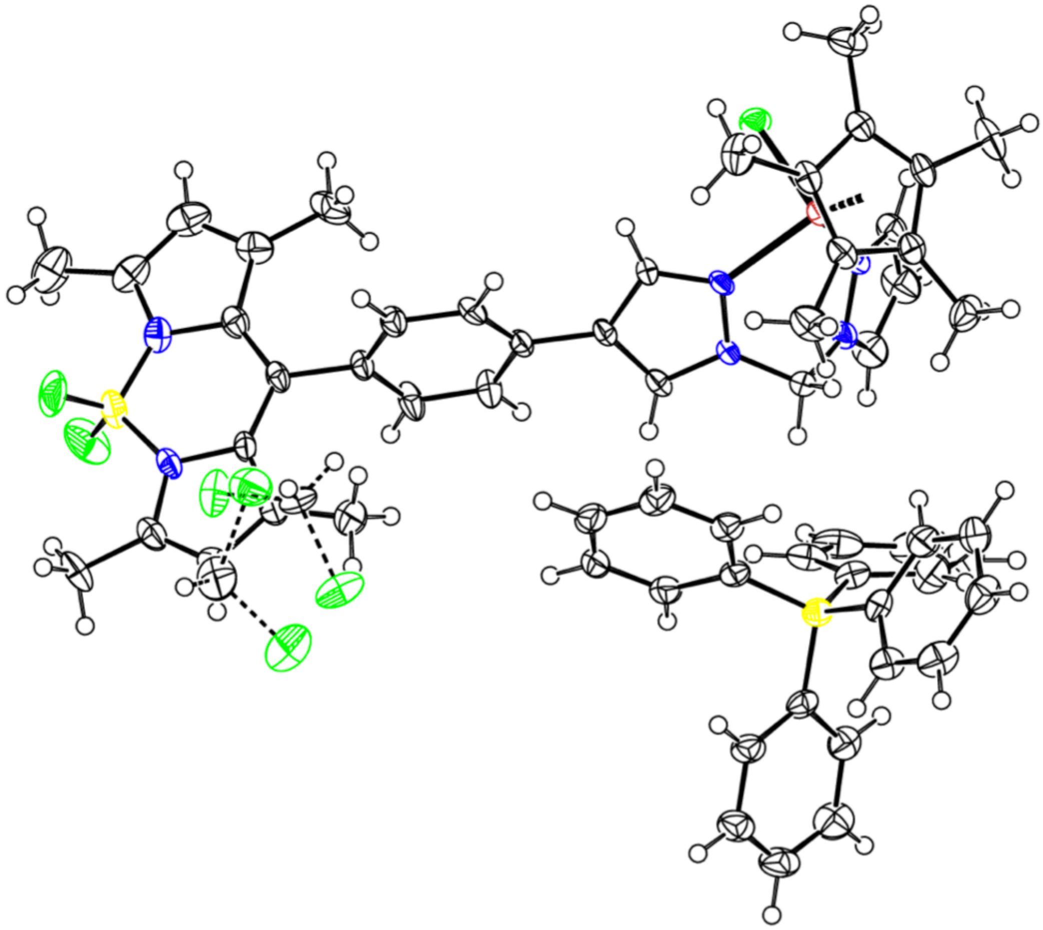

Figure S5: ORTEP[16] depiction of the molecular structure of [Ir(III)-BDP HS][BPh_4].

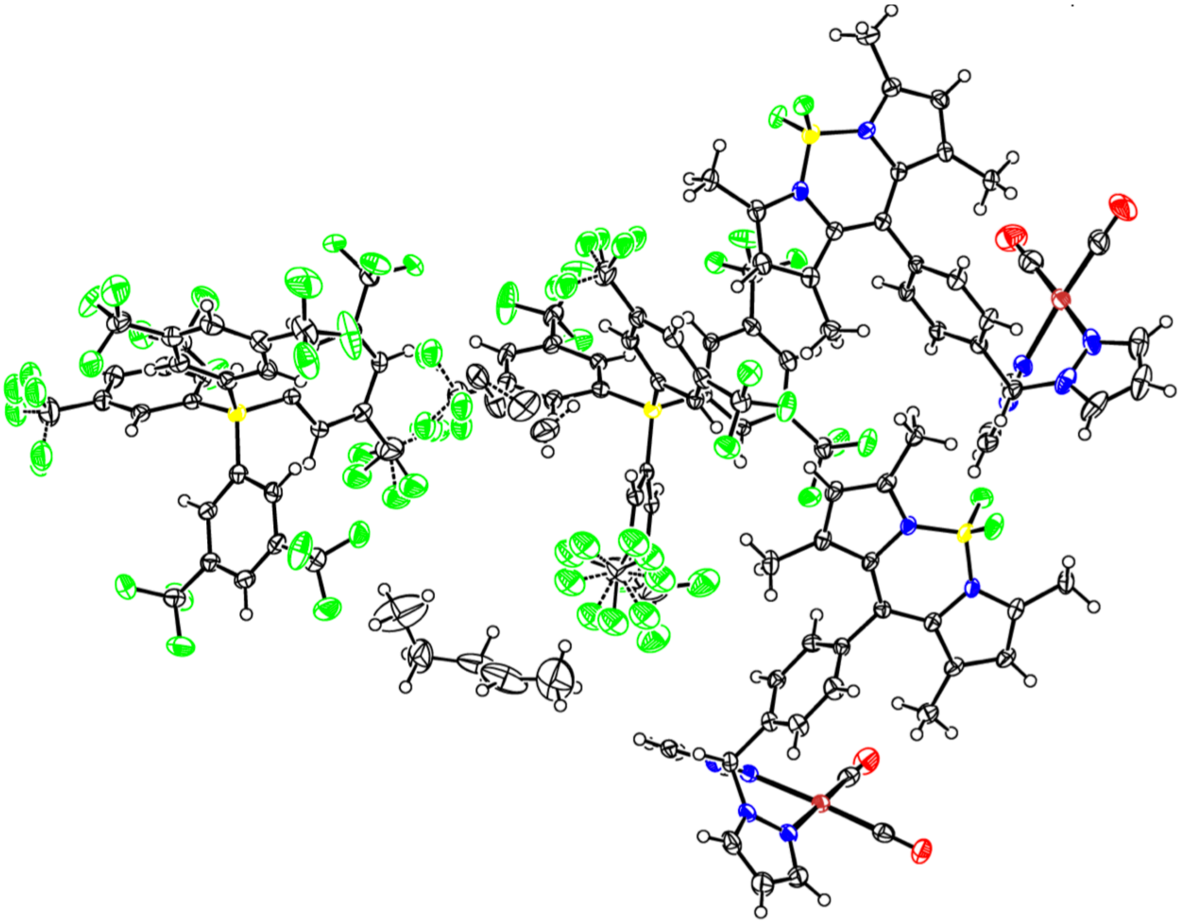

Figure S6: ORTEP[16] depiction of the molecular structure of Ir(I)-BDP HH **5**.

Infrared Spectroscopy Measurements

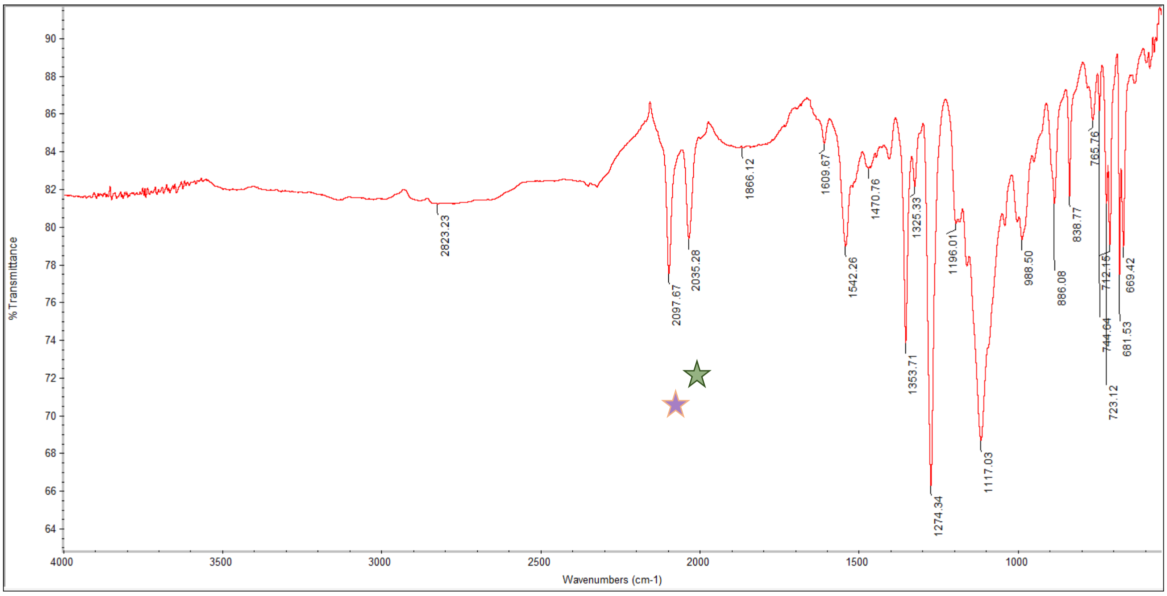

Figure S7: The infrared spectrum of Ir(I)-BDP SS **4**, with the peaks due to CO vibration indicated with a star.

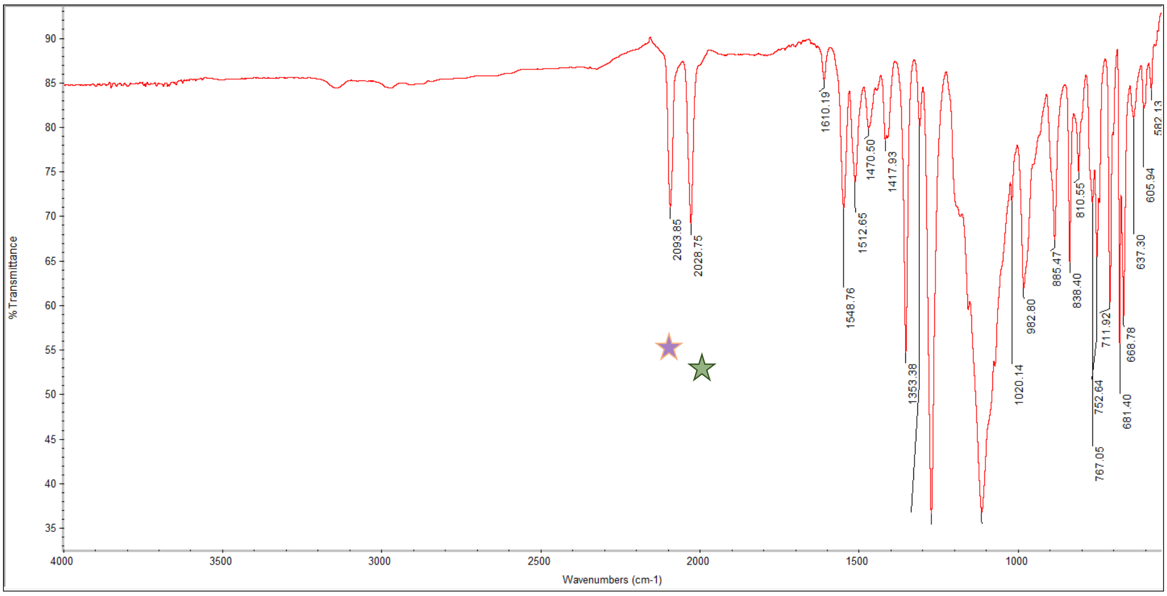

Figure S8: The infrared spectrum of Ir(I)-BDP HH **5**, with the peaks due to CO vibration indicated with a star.

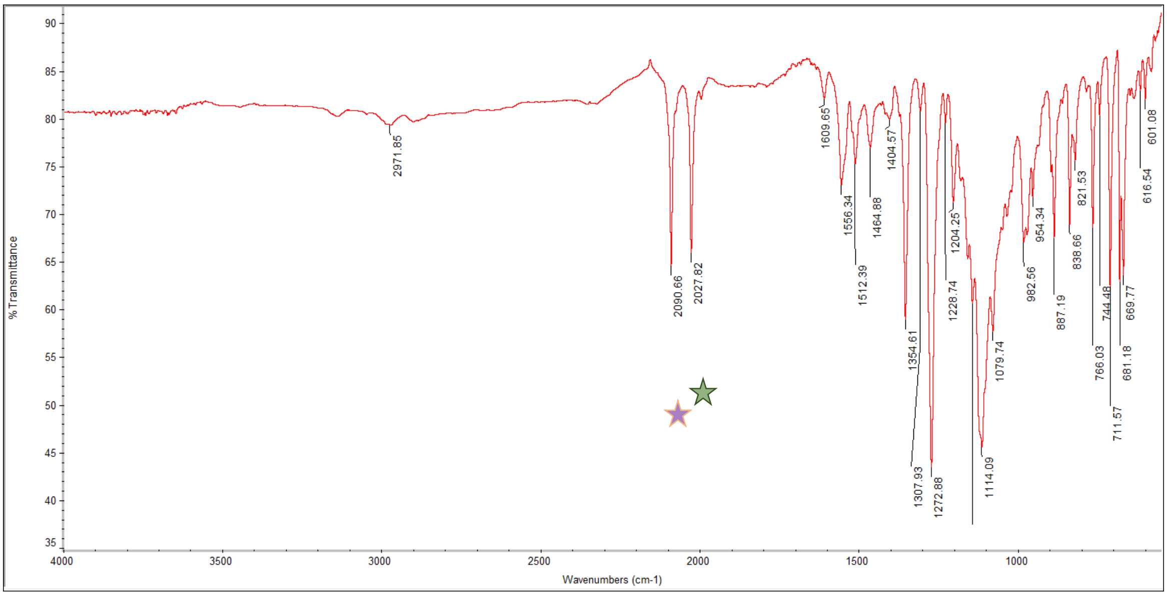

Figure S9: The infrared spectrum of Ir(I)-BDP HS **6**, with the peaks due to CO vibration indicated with a star.

Photophysical Measurements

The absorption and emission spectra of the untethered catalyst mixture

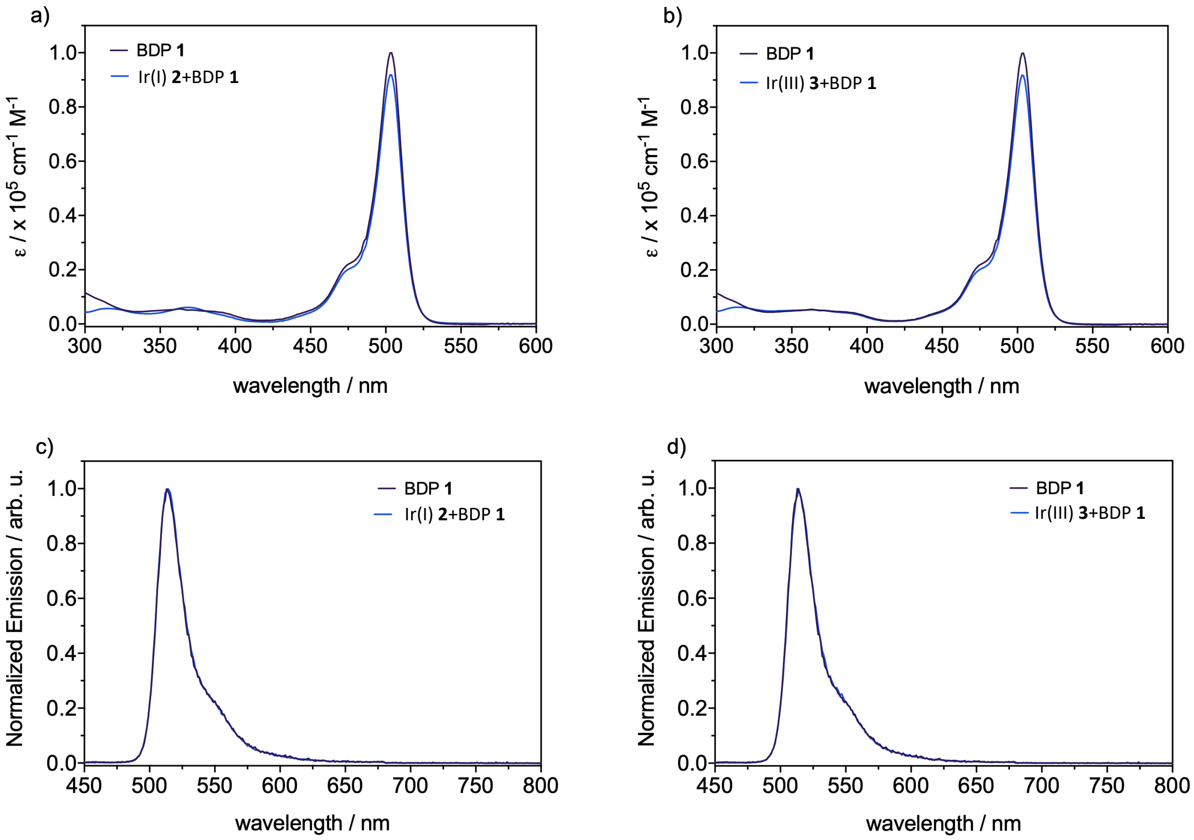

Figure S10 The UV-Vis absorption spectra (a and b, 1 x 10^{-5} mol/L) and normalised emission spectra (c and d) for BDP **1,** Ir(I) **2** + BDP **1** and BDP **1**, Ir(III) **3** + BDP **1** in toluene.

Fluorescent lifetime measurements

Table S3: Emission decay profiles for all complexes.

<table>
<tr><th>Ir(I) bifunctional catalysts 4-6</th><th>Ir(III) bifunctional catalysts 7-9</th></tr>
<tr><td>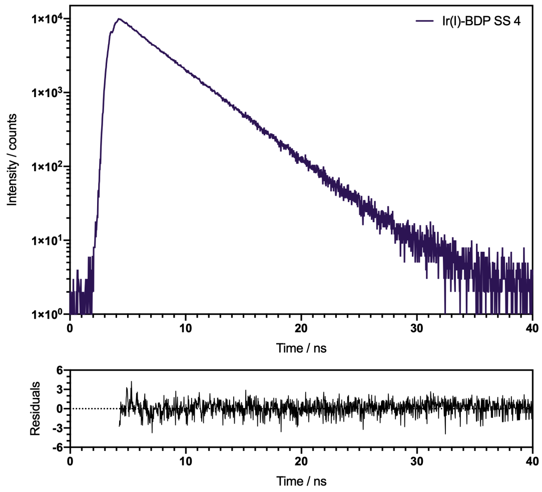
</td><td>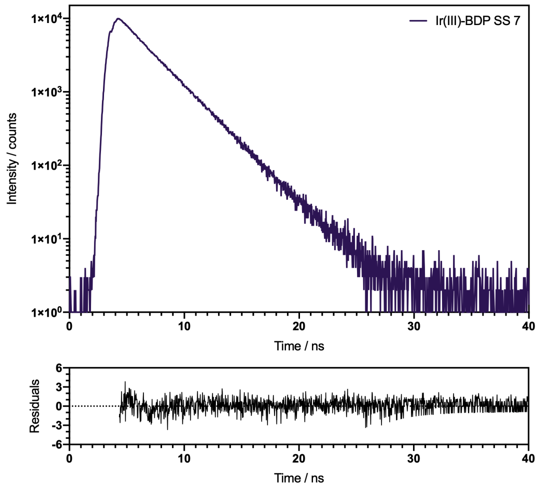
</td></tr>
<tr><td>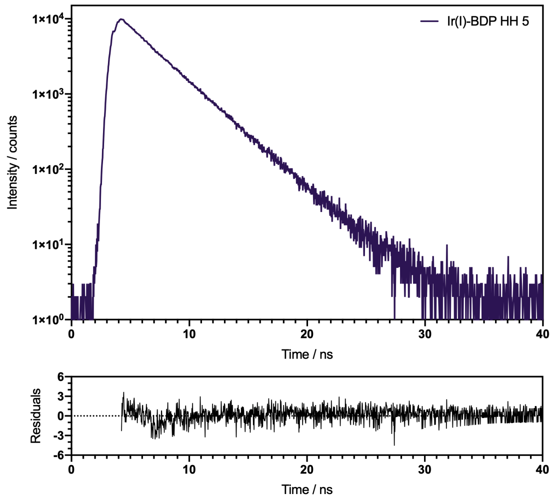
</td><td>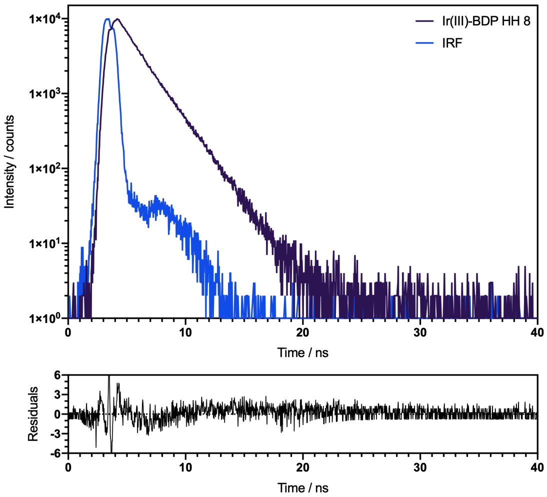
</td></tr>
<tr><td>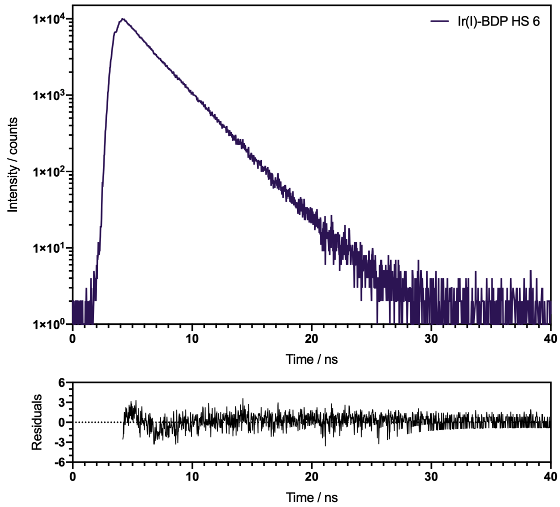
</td><td>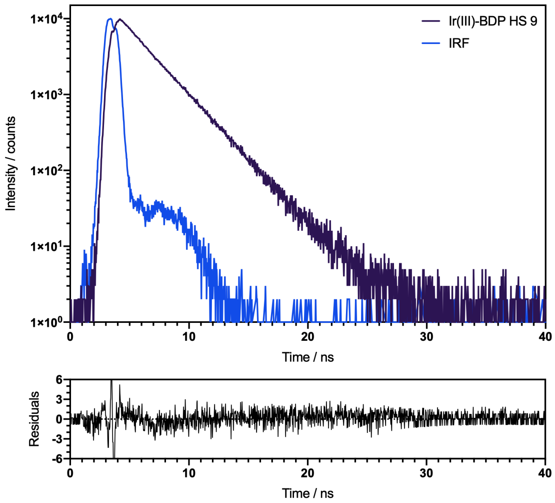
</td></tr>
</table>

Ir(I) **2**+BDP **1** | Ir(III) **3**+BDP **1**

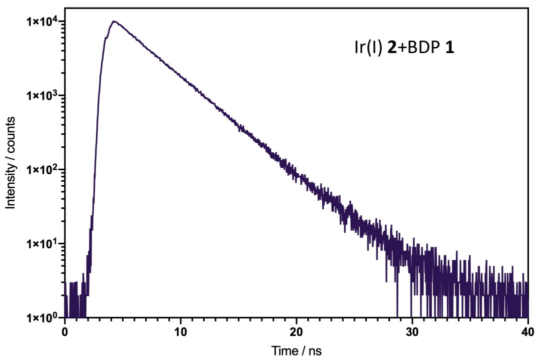

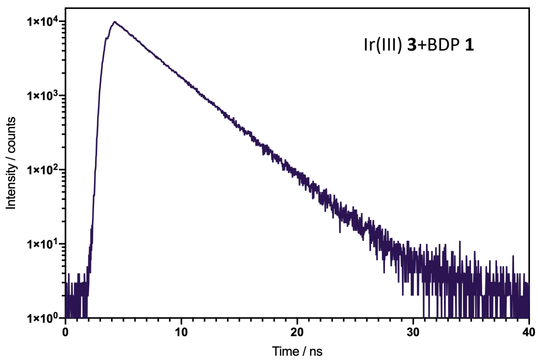

Ligands **13**, **14**, **17** and BDP **1**

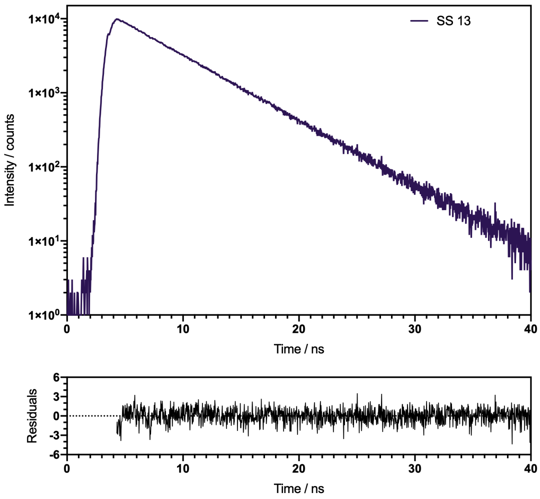

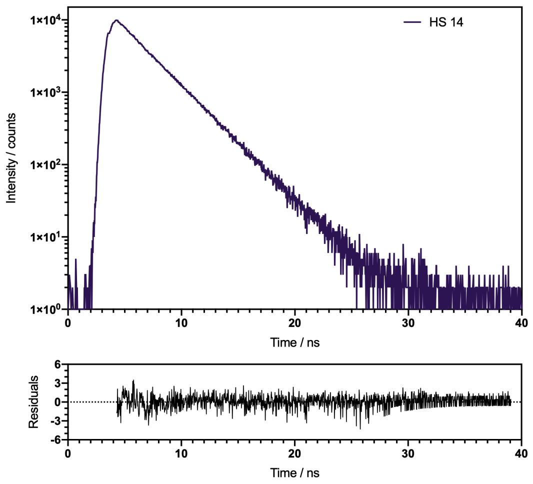

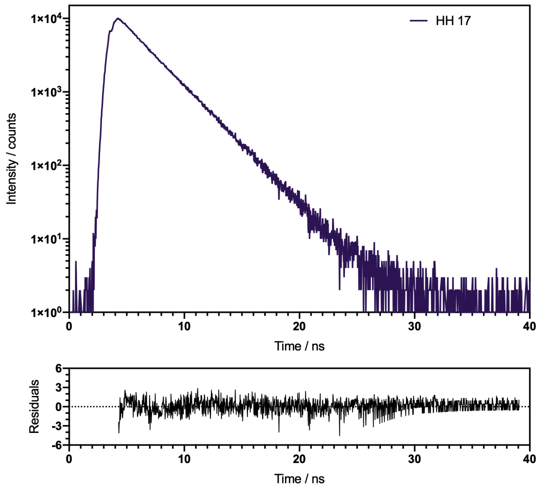

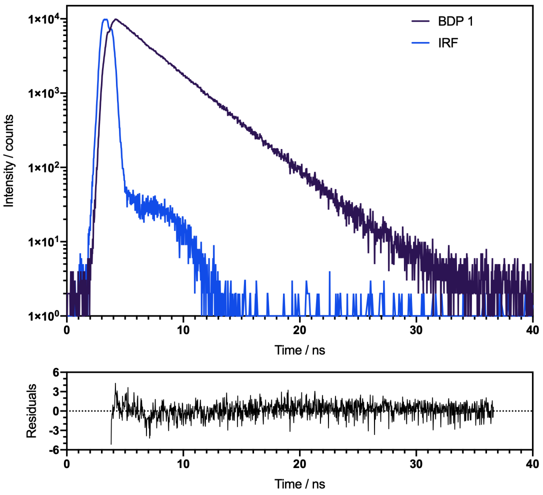

Singlet oxygen quantum yield measurements

The singlet oxygen quantum yields (Φ_Δ) were determined using the chemical trapping method, as frequently used in the literature.[17] *t*-Amyl alcohol was saturated with air by bubbling air through the solvent for 1 hour before use. A mixture of 1,3-diphenylisobenzofuran (DPBF) (3 x 10^{-5} mol/L) and the respective photocatalyst (1 x 10^{-5} mol/L) was dissolved in 2 mL of air saturated *t*-amyl alcohol, and was irradiated under green LED light (max wavelength = 510 nm). The photooxidation of DPBF was monitored over time, ranging from 5 seconds to 2 minutes depending to the efficiency of the photocatalyst. The time dependent absorption graphs showing the decrease in the DPBF signal at 410 nm, and the corresponding linear regression from which the rate constant was calculated, are shown in Figures S11 to S23.

The Φ_Δ data was obtained according to Equation 1 using methylene blue (Φ_Δ^{ref} = 0.52 in ethanol)[18] as the reference:

$$\Phi_\Delta = \Phi_\Delta^{ref} \frac{k}{k^{ref}} \frac{I_a^{ref}}{I_a} \quad (1)$$

where k and k^{ref} are the DPBF photobleaching rate constants in the presence of the either the corresponding photocatalyst or methylene blue reference (calculated from the decrease in absorbance at 410 nm), respectively; I_a and I_a^{ref} are the absorption correction factors at the irradiation of 510 nm by the samples and the standard, respectively. I_a and I_a^{ref} were calculated according to Equation 2, where A_{510} is the absorption of the species at 510 nm.

$$\frac{I_a^{ref}}{I_a} = \frac{1 - 10^{-A_{510}^{ref}}}{1 - 10^{-A_{510}}} \quad (2)$$

Methylene blue reference

Repeat 1

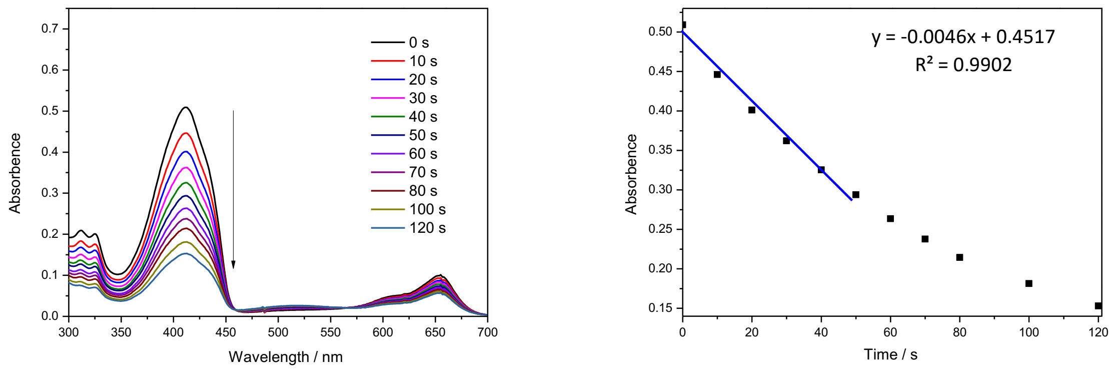

Repeat 2

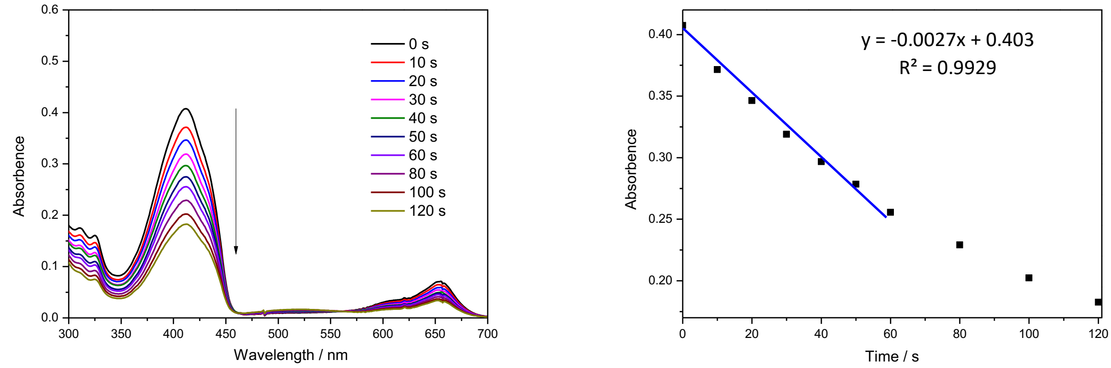

Repeat 3

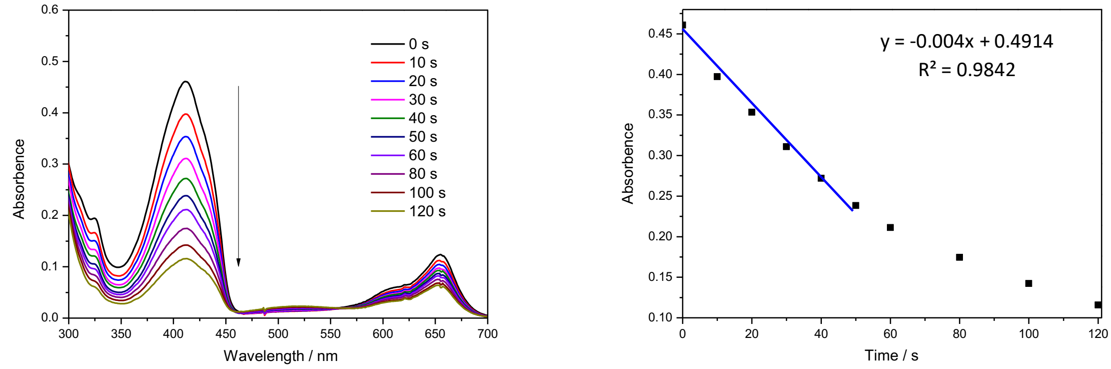

Figure S11. Left: the decay in the DPBF signal in the absorption spectra over time, as a mixture of methylene blue (1 x 10^{-5} mol/L) and DPBF (3 x 10^{-5} mol/L) in *t*-amyl alcohol was irradiated with a green LED (max wavelength = 510 nm). Right: The decrease in absorbance at 410 nm over time, showing the linear regression from which the rate constant was calculated. Experiments were performed in triplicate.

*BDP **1***

Repeat 1

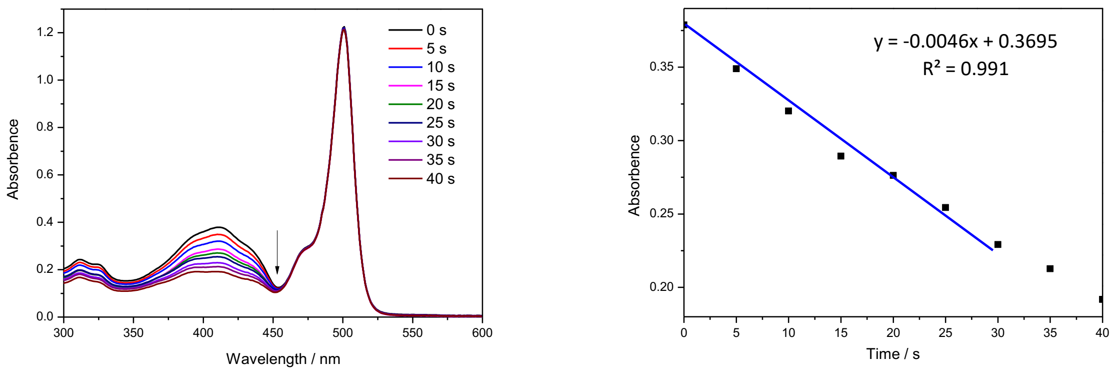

Repeat 2

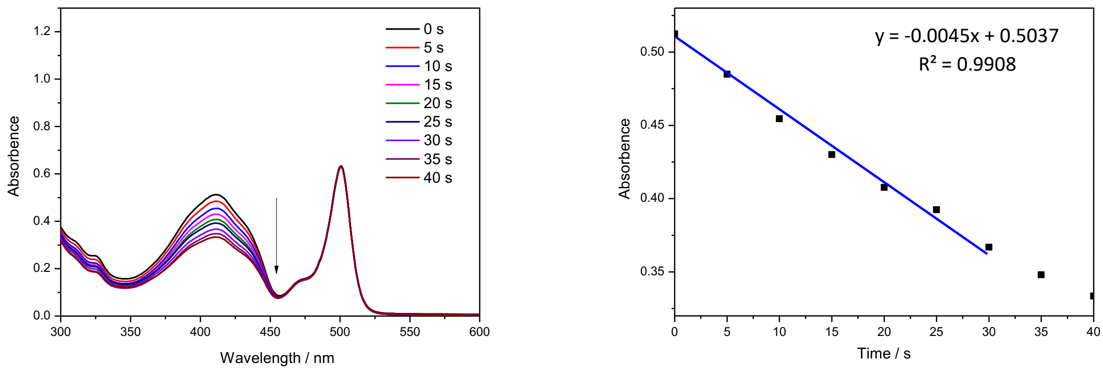

Figure S12: Left: the decay in the DPBF signal in the absorption spectra over time, as a mixture of BDP **1** (1×10^{-5} mol/L) and DPBF (3×10^{-5} mol/L) in *t*-amyl alcohol was irradiated with a green LED (max wavelength = 510 nm). Right: The decrease in absorbance at 410 nm over time, showing the linear regression from which the rate constant was calculated. Experiments were performed in duplicate.

Ir(I) ***2***

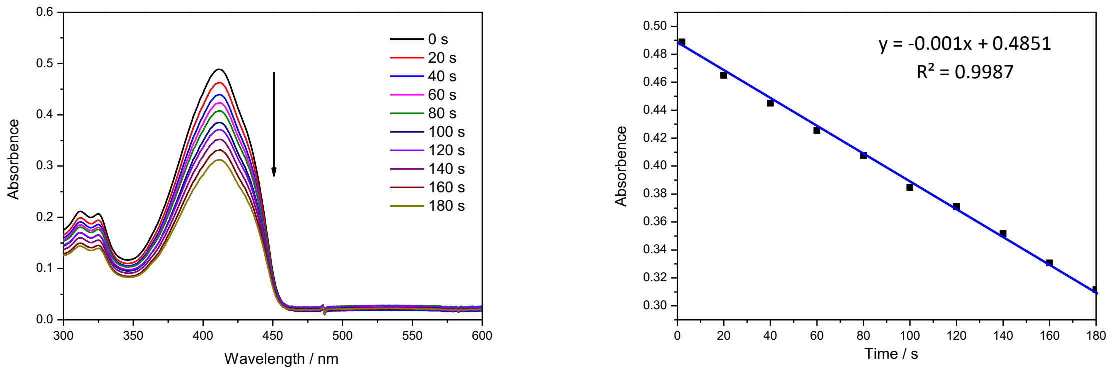

Figure S13: Left: the decay in the DPBF signal in the absorption spectra over time, as a mixture of Ir(I) **2** (1 x 10^{-5} mol/L) and DPBF (3 x 10^{-5} mol/L) in *t*-amyl alcohol was irradiated with a green LED (max wavelength = 510 nm). Right: The decrease in absorbance at 410 nm over time, showing the linear regression from which the rate constant was calculated.

Ir(III) ***3***

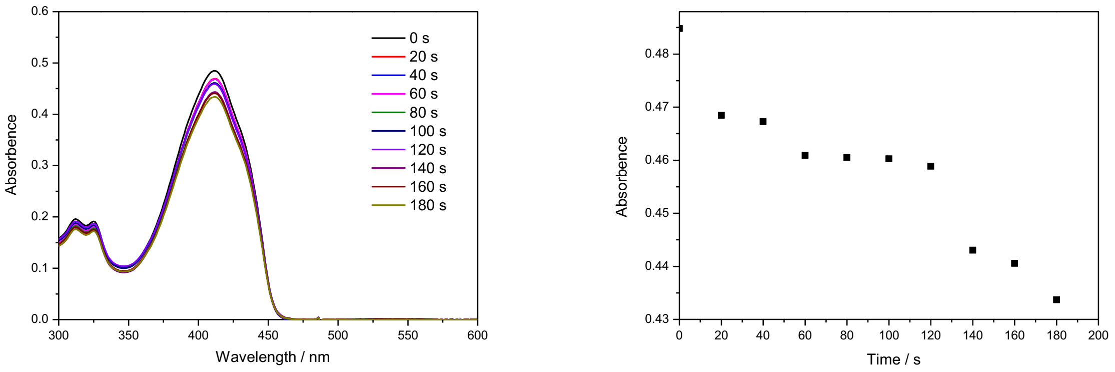

Figure S14: Left: the decay in the DPBF signal in the absorption spectra over time, as a mixture of Ir(III) **3** (1 x 10^{-5} mol/L) and DPBF (3 x 10^{-5} mol/L) in *t*-amyl alcohol was irradiated with a green LED (max wavelength = 510 nm). Right: The decrease in absorbance at 410 nm over time, with no linear regression performed as there was only a small decrease in absorbance over time.

Control without catalyst (DPBF only)

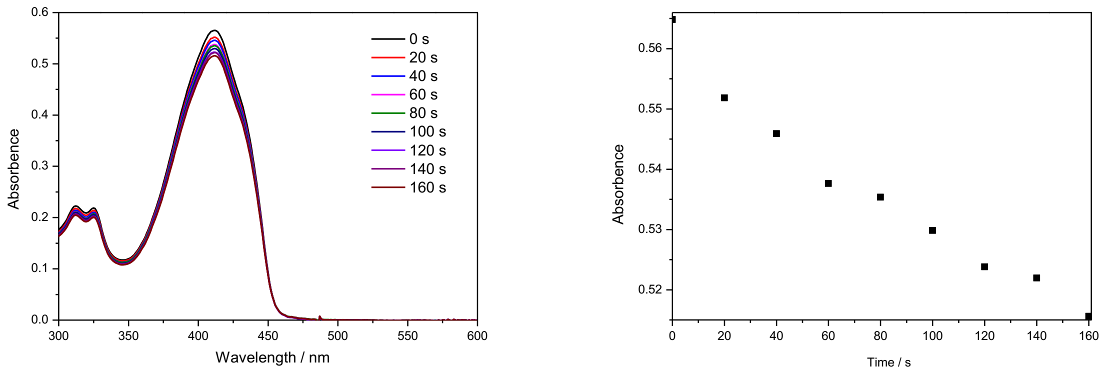

Figure S15: Left: the decay in the DPBF signal in the absorption spectra over time, as DPBF (3 x 10^{-5} mol/L) in *t*-amyl alcohol was irradiated with a green LED (max wavelength = 510 nm). Right: The decrease in absorbance at 410 nm over time, with no linear regression performed as there was only a small decrease in absorbance over time.

Ir(I)-BDP SS ***4***

Repeat 1

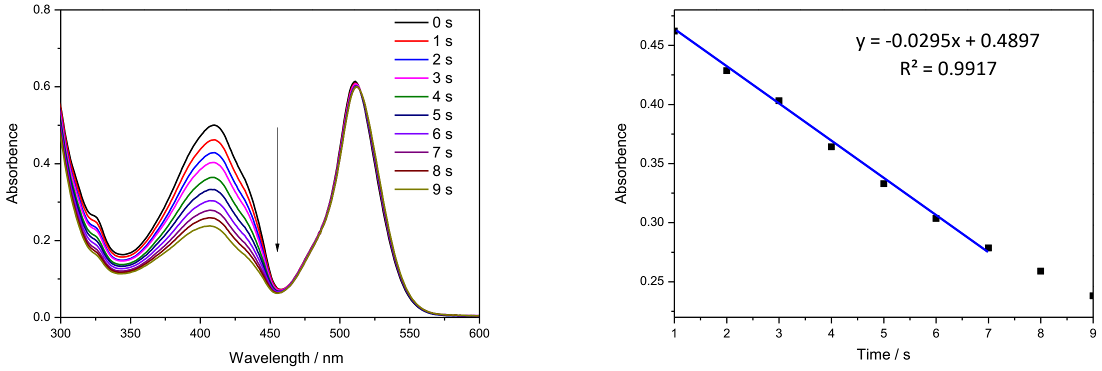

Repeat 2

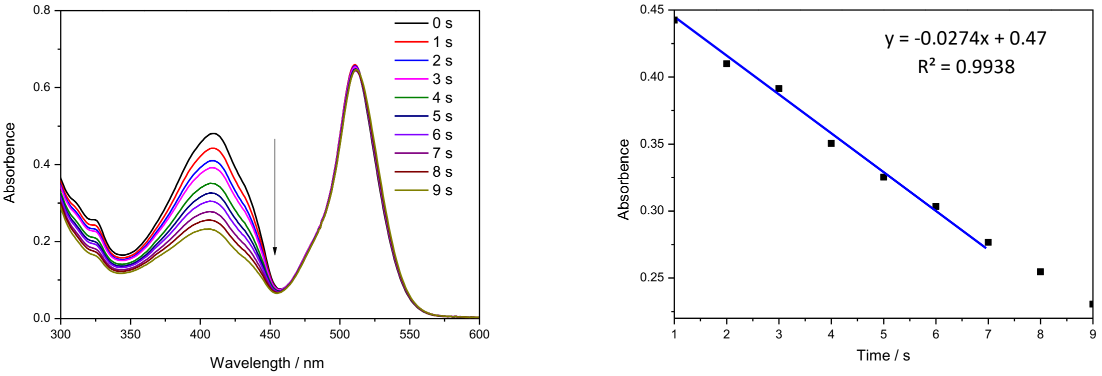

Repeat 3

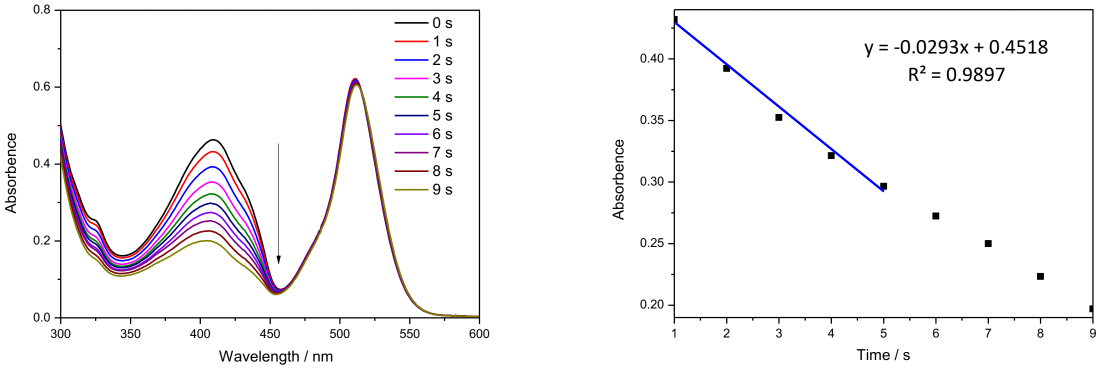

Figure S16: Left: the decay in the DPBF signal in the absorption spectra over time, as a mixture of Ir(I)-BDP SS **4** (1×10^{-5} mol/L) and DPBF (3×10^{-5} mol/L) in *t*-amyl alcohol was irradiated with a green LED (max wavelength = 510 nm). Right: The decrease in absorbance at 410 nm over time, showing the linear regression from which the rate constant was calculated. Experiments were performed in triplicate.

Ir(I)-BDP HH ***5***

Repeat 1

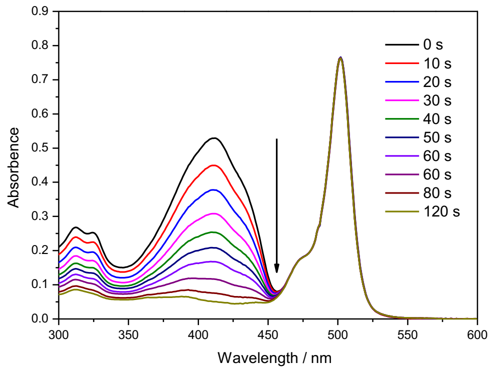

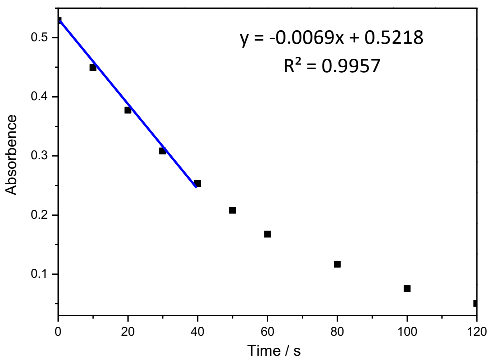

Repeat 2

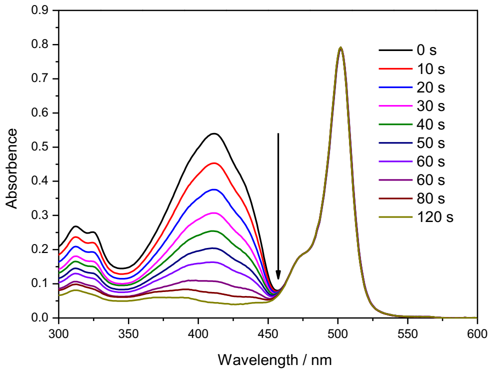

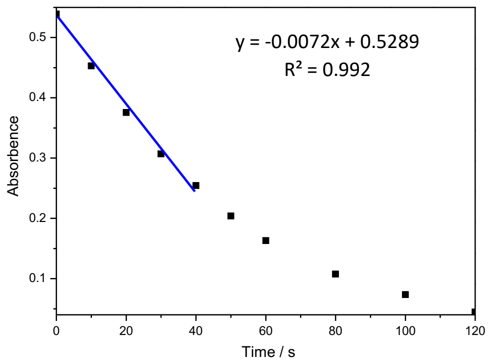

Figure S17: Left: the decay in the DPBF signal in the absorption spectra over time, as a mixture of Ir(I)-BDP HH **5** (1 x 10^{-5} mol/L) and DPBF (3 x 10^{-5} mol/L) in *t*-amyl alcohol was irradiated with a green LED (max wavelength = 510 nm). Right: The decrease in absorbance at 410 nm over time, showing the linear regression from which the rate constant was calculated. Experiments were performed in duplicate.

Ir(I)-BDP HS ***6***

Repeat 1

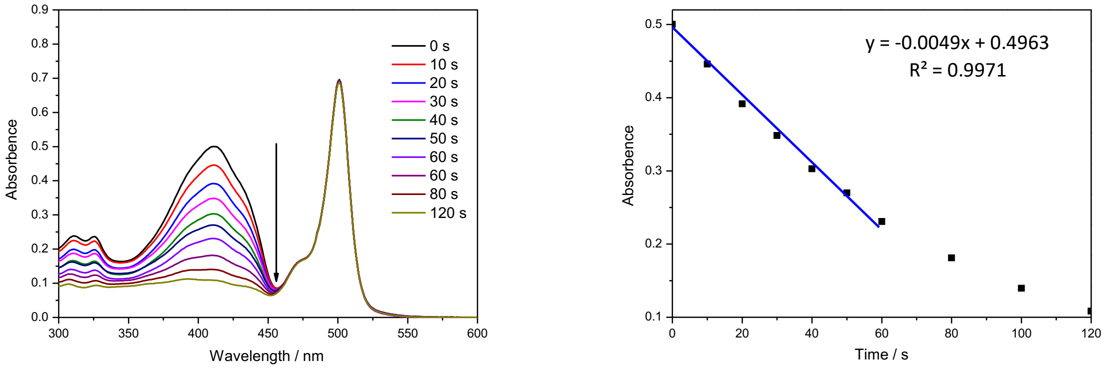

Repeat 2

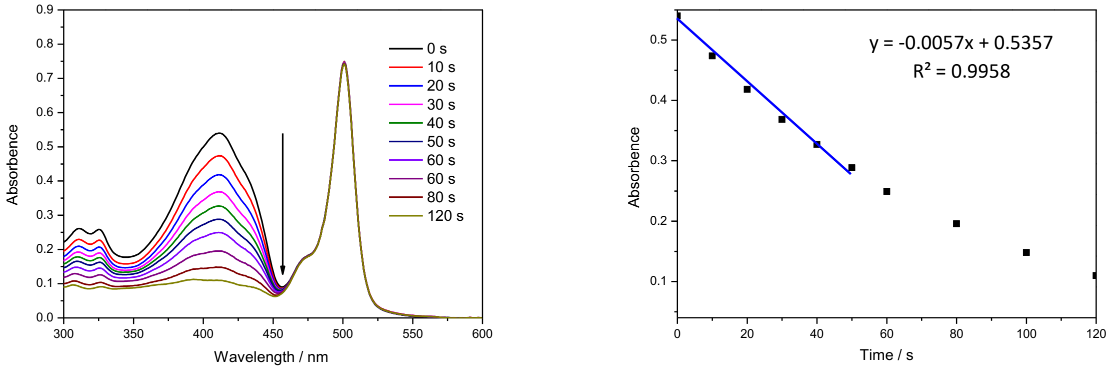

Figure S18: Left: the decay in the DPBF signal in the absorption spectra over time, as a mixture of Ir(I)-BDP HS **6** (1 x 10^{-5} mol/L) and DPBF (3 x 10^{-5} mol/L) in *t*-amyl alcohol was irradiated with a green LED (max wavelength = 510 nm). Right: The decrease in absorbance at 410 nm over time, showing the linear regression from which the rate constant was calculated. Experiments were performed in duplicate.

Ir(III)-BDP SS ***7***

Repeat 1

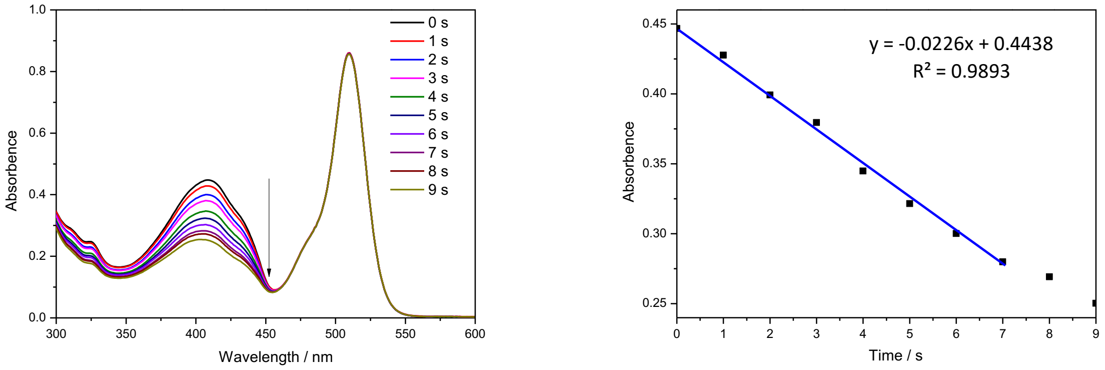

Repeat 2

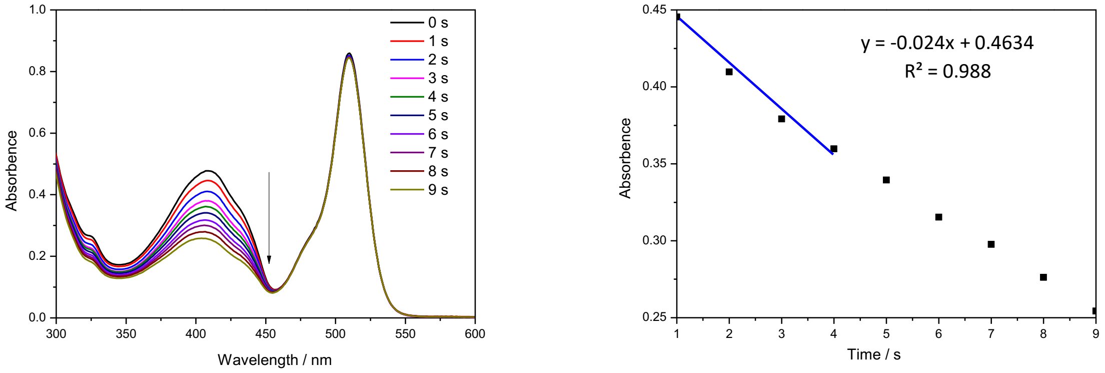

Repeat 3

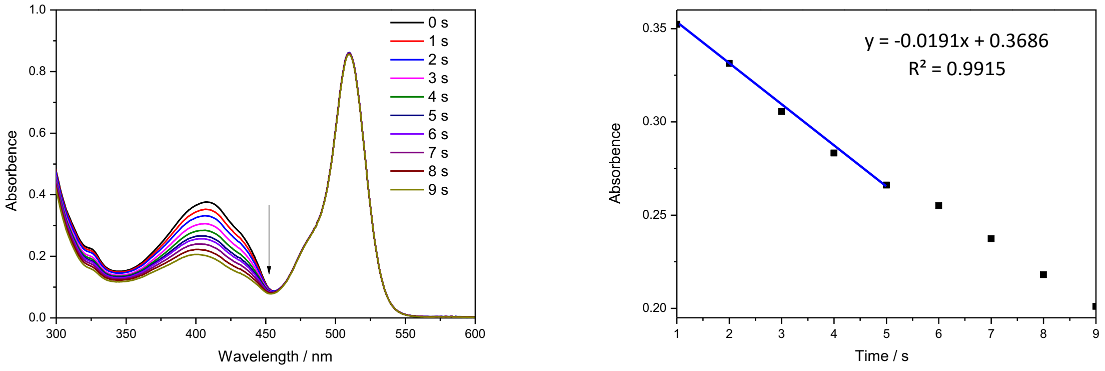

Figure S19: Left: the decay in the DPBF signal in the absorption spectra over time, as a mixture of Ir(III)-BDP SS **7** (1×10^{-5} mol/L) and DPBF (3×10^{-5} mol/L) in *t*-amyl alcohol was irradiated with a green LED (max wavelength = 510 nm). Right: The decrease in absorbance at 410 nm over time, showing the linear regression from which the rate constant was calculated. Experiments were performed in triplicate.

*Ir(III)-BDP HH **8***

Repeat 1

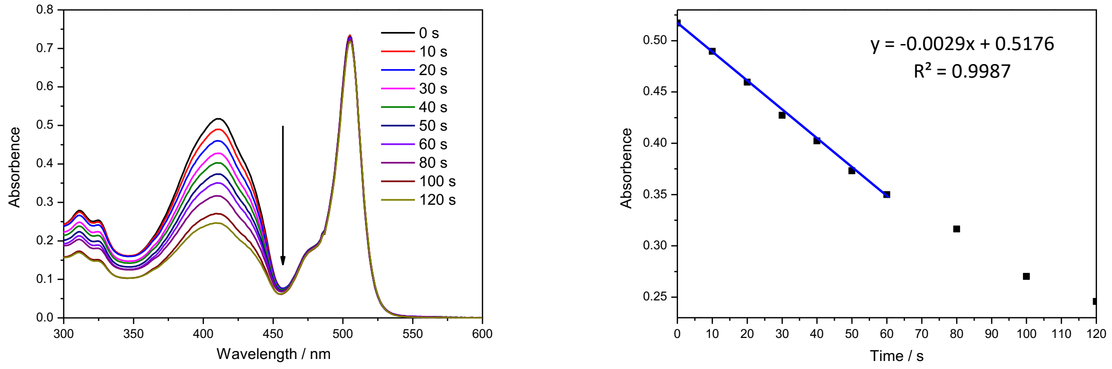

Repeat 2

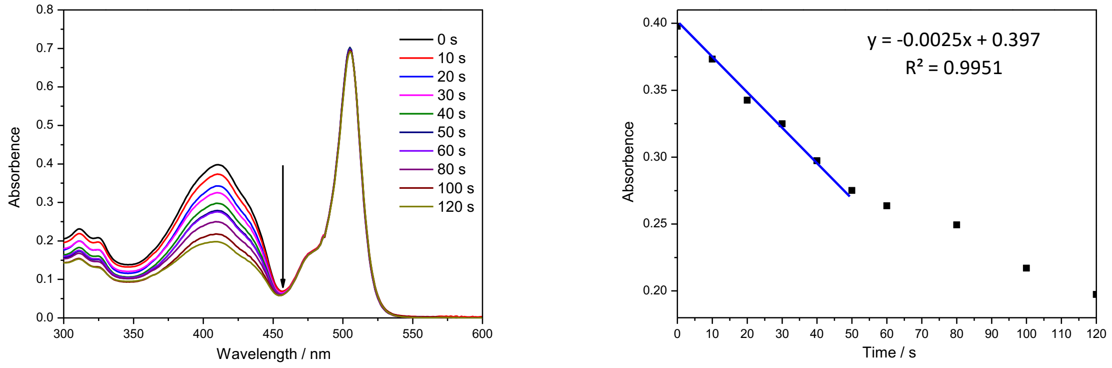

Figure S20: Left: the decay in the DPBF signal in the absorption spectra over time, as a mixture of Ir(III)-BDP HH **8** (1 x 10^{-5} mol/L) and DPBF (3 x 10^{-5} mol/L) in *t*-amyl alcohol was irradiated with a green LED (max wavelength = 510 nm). Right: The decrease in absorbance at 410 nm over time, showing the linear regression from which the rate constant was calculated. Experiments were performed in duplicate.

*Ir(III)-BDP HS **9***

Repeat 1

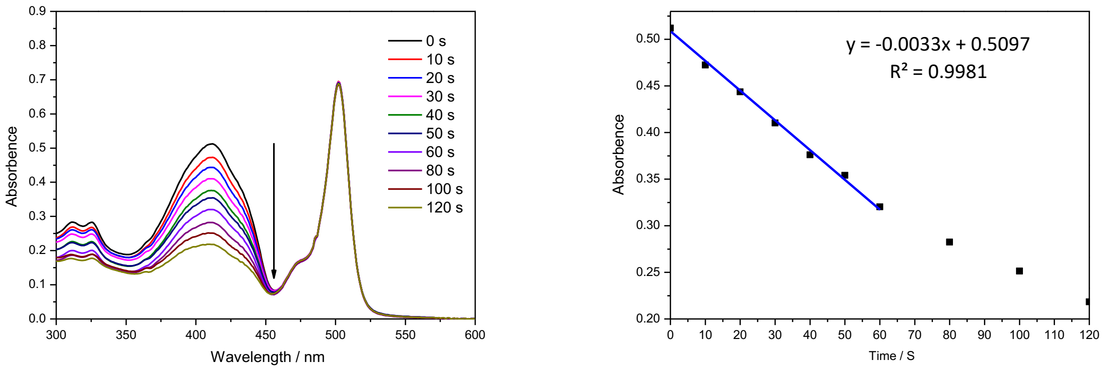

Repeat 2

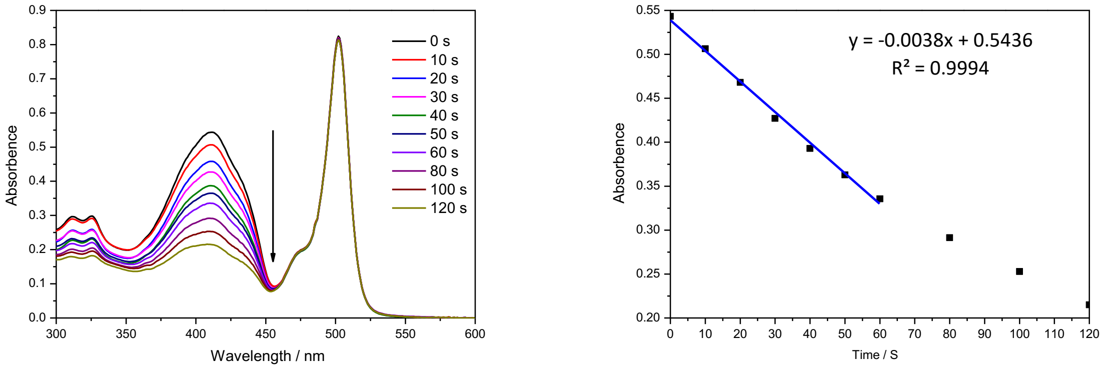

Figure S21: Left: the decay in the DPBF signal in the absorption spectra over time, as a mixture Ir(III)-BDP HS **9** (1 x 10^{-5} mol/L) and DPBF (3 x 10^{-5} mol/L) in *t*-amyl alcohol was irradiated with a green LED (max wavelength = 510 nm). Right: The decrease in absorbance at 410 nm over time, showing the linear regression from which the rate constant was calculated. Experiments were performed in duplicate.

*BDP **1** + Ir(I) **2***

Repeat 1

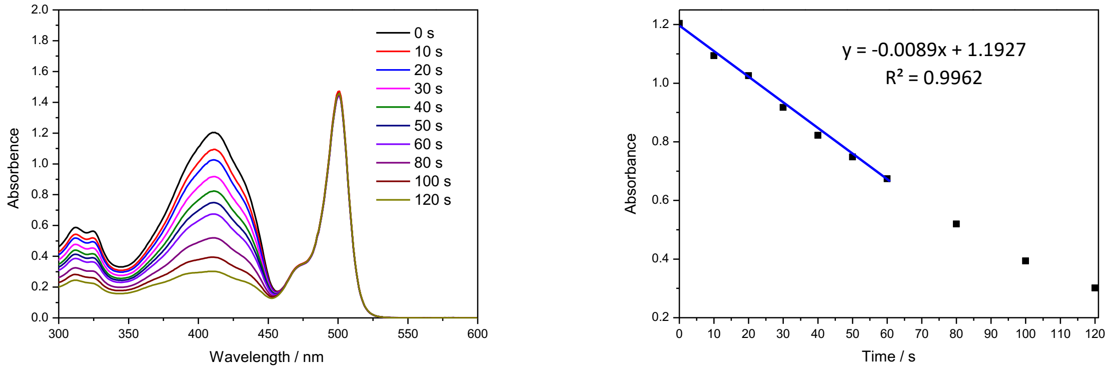

Repeat 2

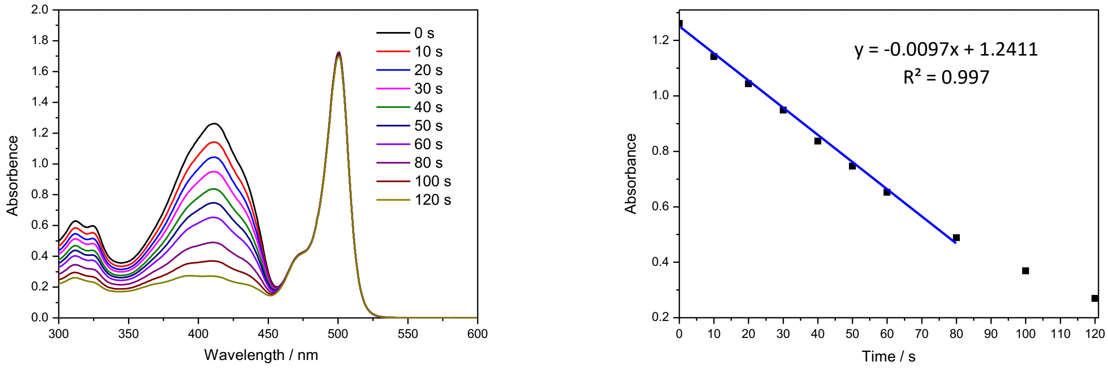

Repeat 3

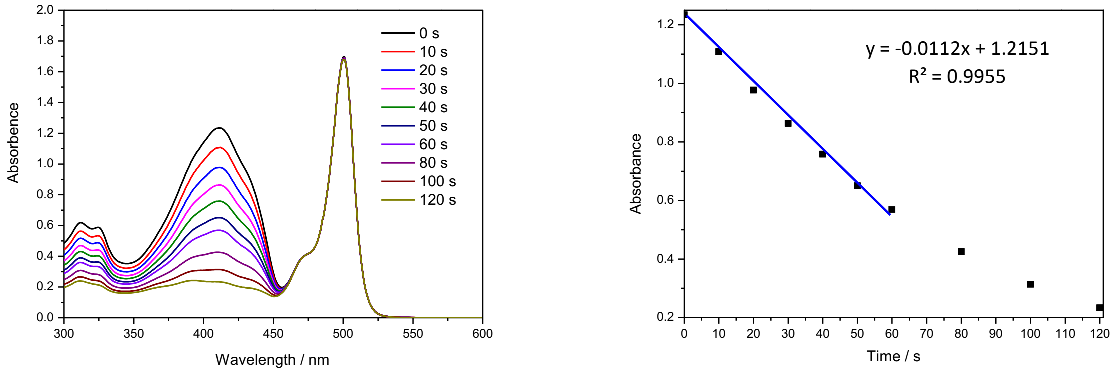

Figure S22: Left: the decay in the DPBF signal in the absorption spectra over time, as a mixture BDP **1** (1 x 10^{-5} mol/L), Ir(I) **2** (1 x 10^{-5} mol/L) and DPBF (3 x 10^{-5} mol/L) in *t*-amyl alcohol was irradiated with a green LED (max wavelength = 510 nm). Right: The decrease in absorbance at 410 nm over time, showing the linear regression from which the rate constant was calculated. Experiments were performed in triplicate.

*BDP **1** + Ir(III) **3***

Repeat 1

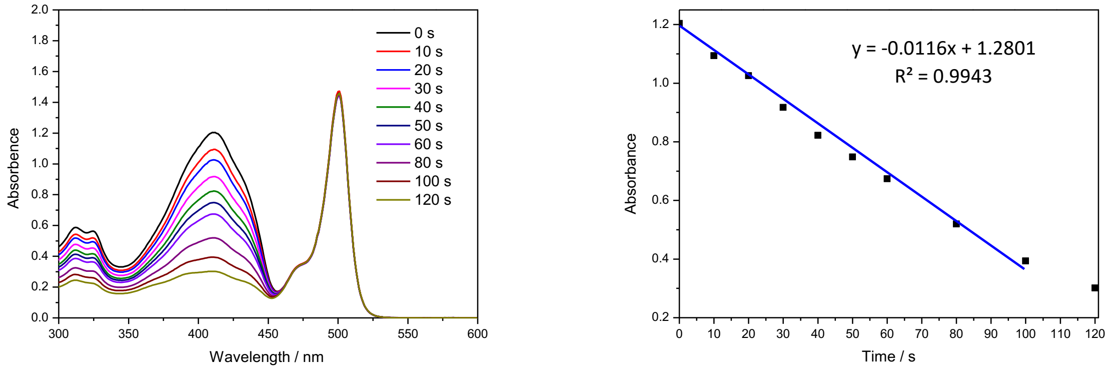

Repeat 2

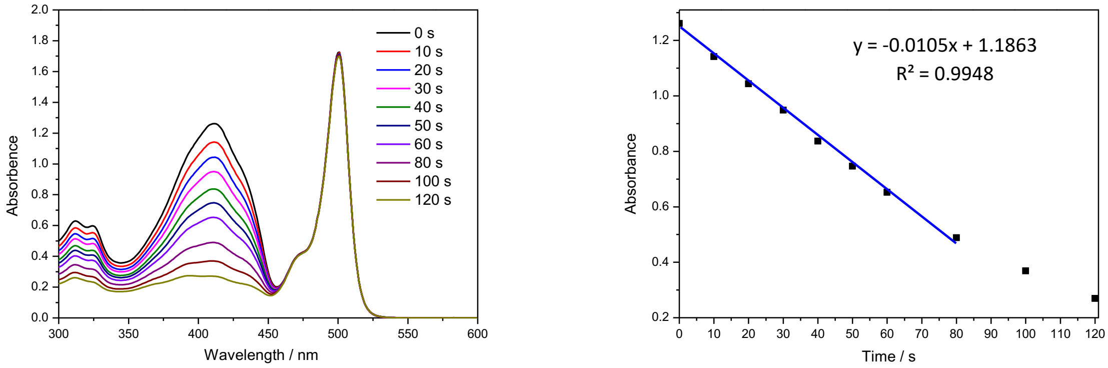

Repeat 3

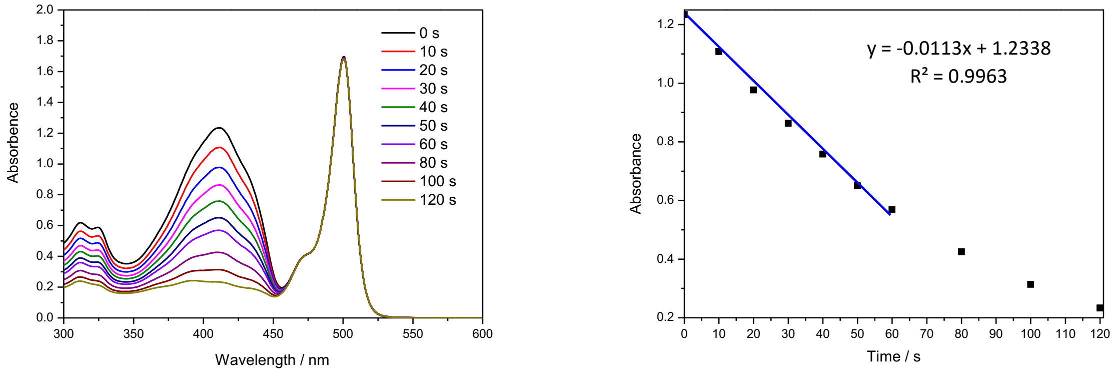

Figure S23: Left: the decay in the DPBF signal in the absorption spectra over time, as a mixture BDP **1** (1 x 10^{-5} mol/L), Ir(III) **3** (1 x 10^{-5} mol/L) and DPBF (3 x 10^{-5} mol/L) in *t*-amyl alcohol was irradiated with a green LED (max wavelength = 510 nm). Right: The decrease in absorbance at 410 nm over time, showing the linear regression from which the rate constant was calculated. Experiments were performed in triplicate.

Transient absorption measurements

Data Modelling

The absorption data (A) is globally modelled as a sum of two exponential decays (with A_2 set to zero when no long-lived signal was observed), with wavelength depended pre-exponential factors convoluted with the instrument response function (IRF), equation (3).

$$\mathrm{A}(\lambda, \mathrm{t}, \mathrm{IRF}) = \mathrm{A}_1(\lambda)\mathrm{e}^{-\tau_s \mathrm{t}} \otimes \mathrm{i}(\mathrm{IRF}) + \mathrm{A}_2(\lambda)\mathrm{e}^{-\tau_2 \mathrm{t}} \otimes \mathrm{i}(\mathrm{IRF}) \quad (3)$$

where:

$$singlet\ lifetime = \tau_s$$
$$\tau_t = \tau_2\ measured\ without\ oxygen$$
$$\tau_q = \tau_2\ measured\ with\ oxygen$$
$$i(IRF) = gaussian\ instrument\ response\ function$$

These pre-exponential factors give rise to decay associated spectra (DAS), which are then presented as species associated spectra (SAS) assuming a sequential kinetic model. Without oxygen present τ_2 is taken as the intrinsic triplet lifetime (τ_t) and with oxygen, τ_2 is the lifetime of the triplet with quencher present (τ_q). In order to calculate oxygen quenching yields equations (4) and (5) are used with the aforementioned lifetimes.

$$\tau_t = \frac{1}{k_t} \quad (4)$$

$$\tau_q = \frac{1}{k_q + k_t} \quad (5)$$

In order to deconvolute the fitted decay profiles, a dilute solution of PtOEP in toluene was prepared to measure the IRF of the experimental setup. The IRF was then fixed in the subsequent fitting of the materials of interest.

Intersystem crossing

Estimation of the intersystem crossing (ISC) yields was made by comparing the fitted SAS. The extracted decay associated spectra were scaled so that SAS-1 and SAS-2 had the same intensity and spectral shape in the 460 - 500 nm range. The scale factor then represents the ISC yield.

In all materials, the primary source of error comes from the selection of the SAS region used to calculate the scalar; it must be a region that doesn't have a photoinduced absorption from either the triplet or singlet state, or contain stimulated emission.

This region is kept consistent across all materials and chosen to be on the high energy shoulder to minimise effects from stimulated emission.

Oxygen Quenching

The efficiency of triplet transfer to oxygen was calculated by using the measured lifetimes with and without oxygen present, equation (6) and (7)

$$\Phi_{O_2} = \frac{k_q}{k_q + k_t} \qquad \text{eq (6)}$$

$$\Phi_{O_2} = \tau_q \left(\frac{1}{\tau_q} - \frac{1}{\tau_t} \right) \qquad \text{eq (7)}$$

Figures of data collected

The compounds with long-lived species have three separate datasets: 1. Short time frame (10^{-7} s or 10^{-8} s), without oxygen, 2. Longer time frame without oxygen, 3. Longer time frame (10^{-4} s) with oxygen. The short time range measurements are used to calculate the singlet lifetime.

Control experiments of the catalysts Ir (I) **2** / Ir(III) **3** as well as the 1:1 mixtures of Ir (I) **2** / Ir(III) **3** +BDP **1** were measured using a 355 nm excitation wavelength within 70 ns time frame, and presented in two separated datasets: with and without oxygen.

BDP ***1***

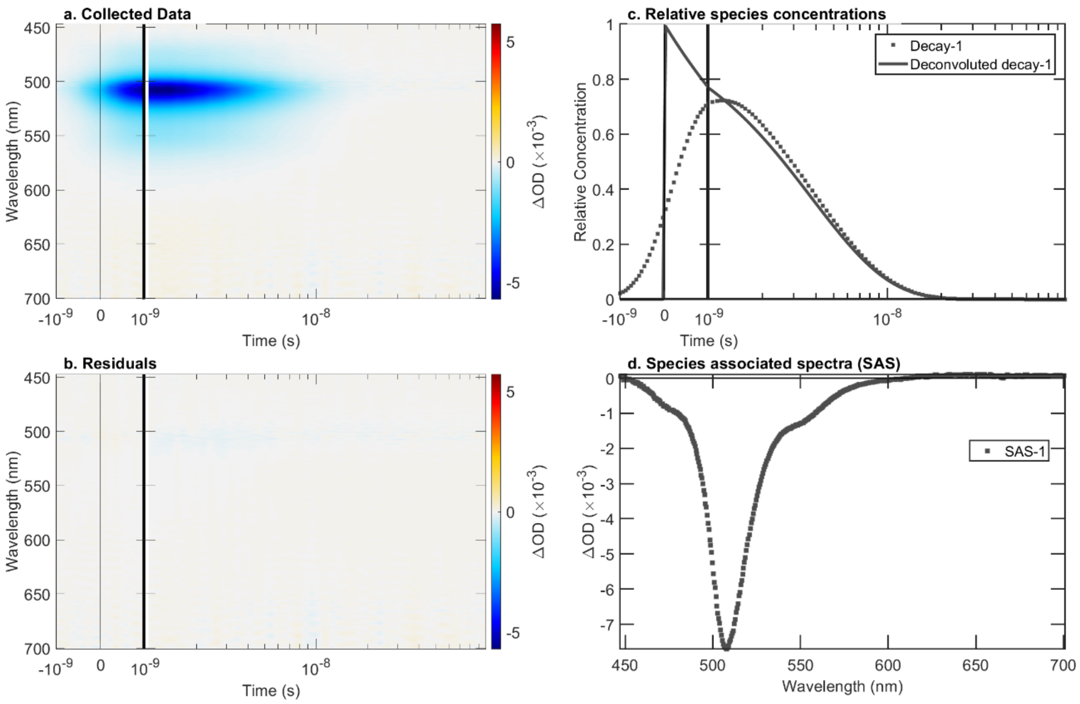

Figure S24: TA measurements (using a 355 nm excitation wavelength) of BDP **1** without oxygen present in the time range of 0 - 10^{-7} s. a) Collected data, b) residual, c) modelled concentrations, d) species associated spectra.

*Ir(I)-BDP SS **4***

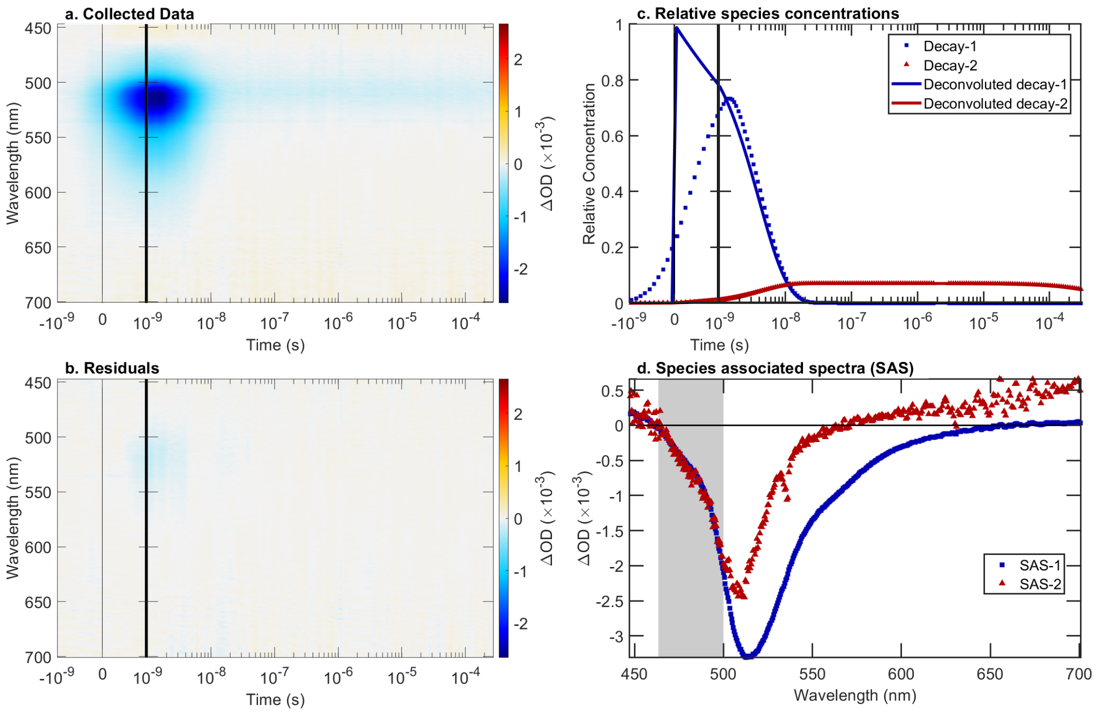

Figure S25: TA measurements (using a 355 nm excitation wavelength) of Ir(I)-BDP SS **4** without oxygen present in the time range of 0 – 10^{-4} s. a) Collected data, b) residual, c) modelled concentrations, d) species associated spectra.

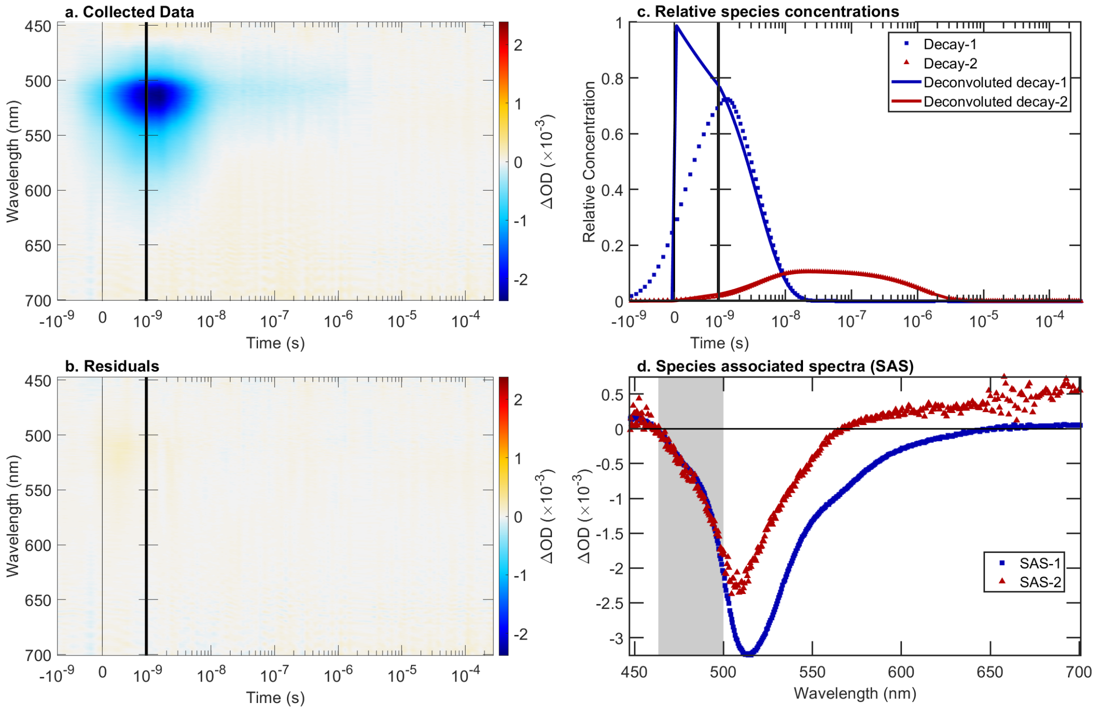

Figure S26: TA measurements (using a 355 nm excitation wavelength) of Ir(I)-BDP SS **4** with oxygen present in the time range of 0 – 10^{-4} s. a) Collected data, b) residual, c) modelled concentrations, d) species associated spectra.

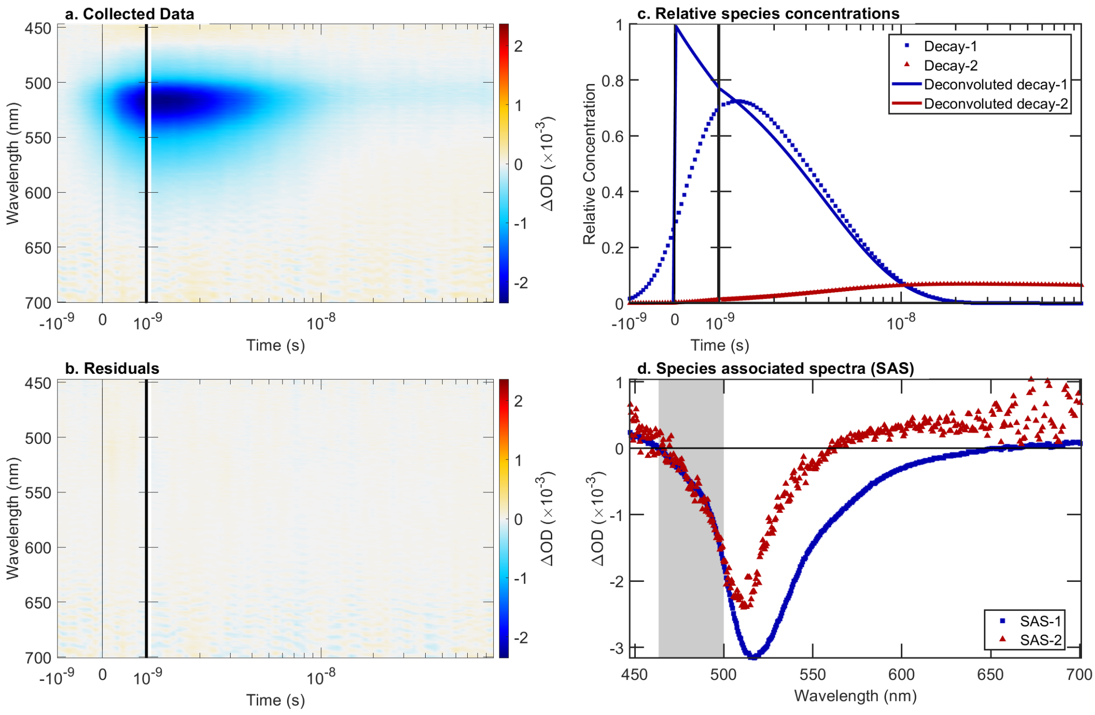

Figure S27: TA measurements (using a 355 nm excitation wavelength) of Ir(I)-BDP SS **4** without oxygen present in the time range of 0 – 10^{-8} s. a) Collected data, b) residual, c) modelled concentrations, d) species associated spectra.

*Ir(I)-BDP HH **5***

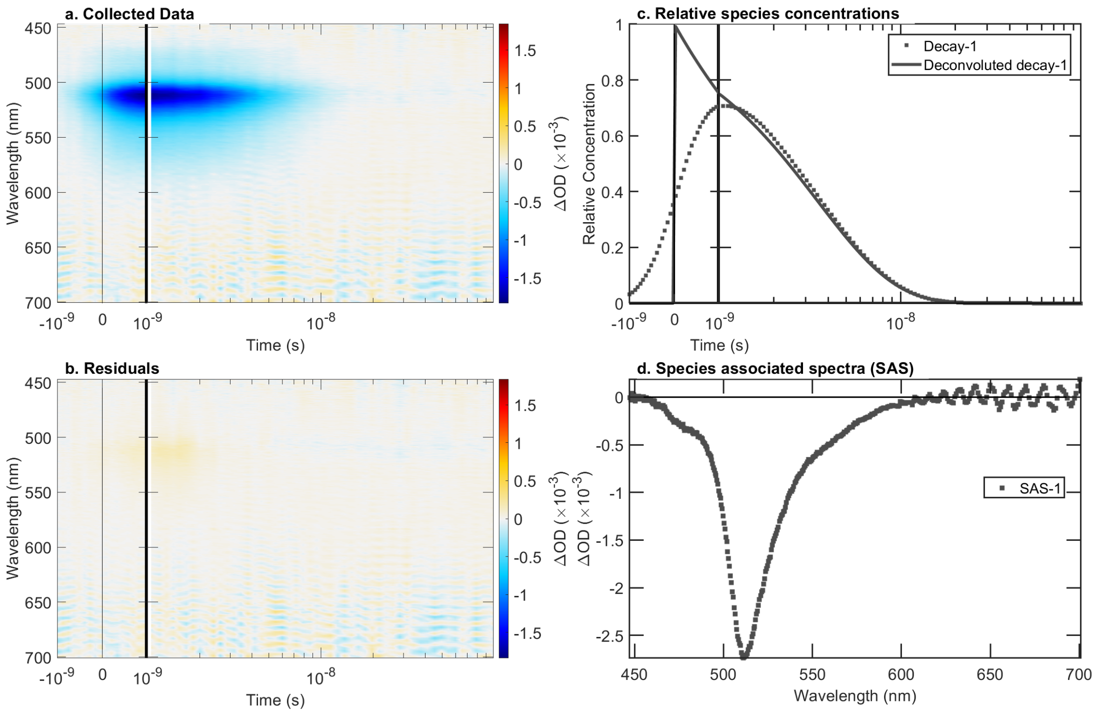

Figure S28: TA measurements (using a 355 nm excitation wavelength) of Ir(I)-BDP HH **5** without oxygen present in the time range of 0 – 10^{-8} s. a) Collected data, b) residual, c) modelled concentrations, d) species associated spectra.

*Ir(I)-BDP HS **6***

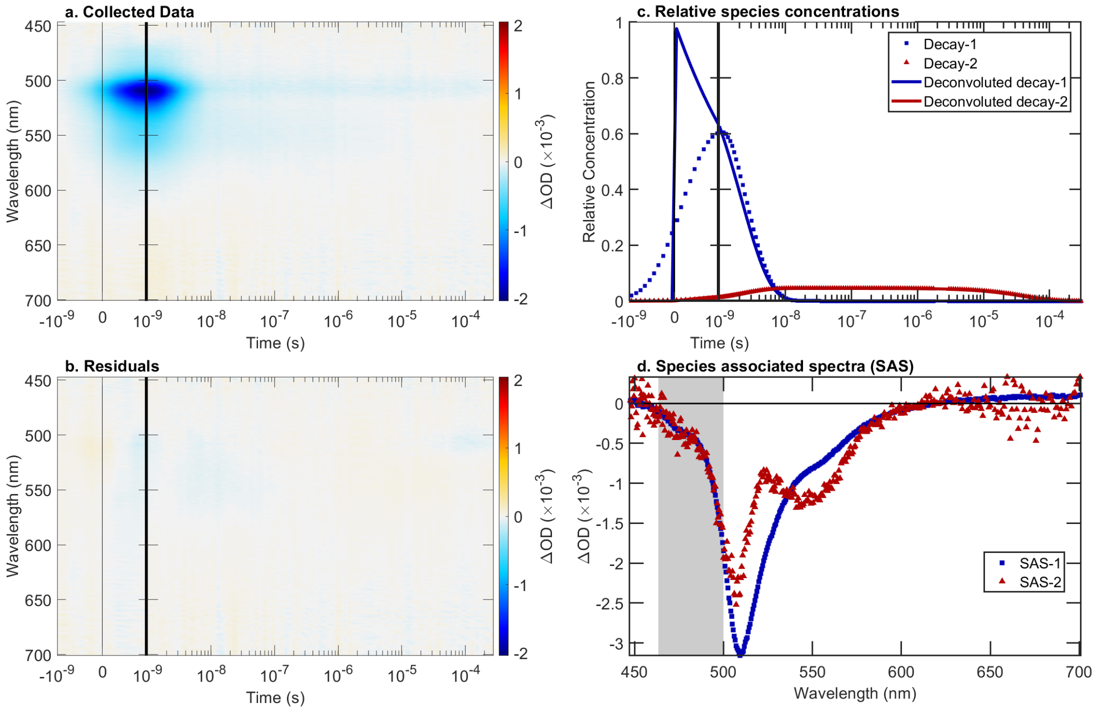

Figure S29: TA measurements (using a 355 nm excitation wavelength) of Ir(I)-BDP HS **6** without oxygen present in the time range of 0 – 10^{-4} s. a) Collected data, b) residual, c) modelled concentrations, d) species associated spectra.

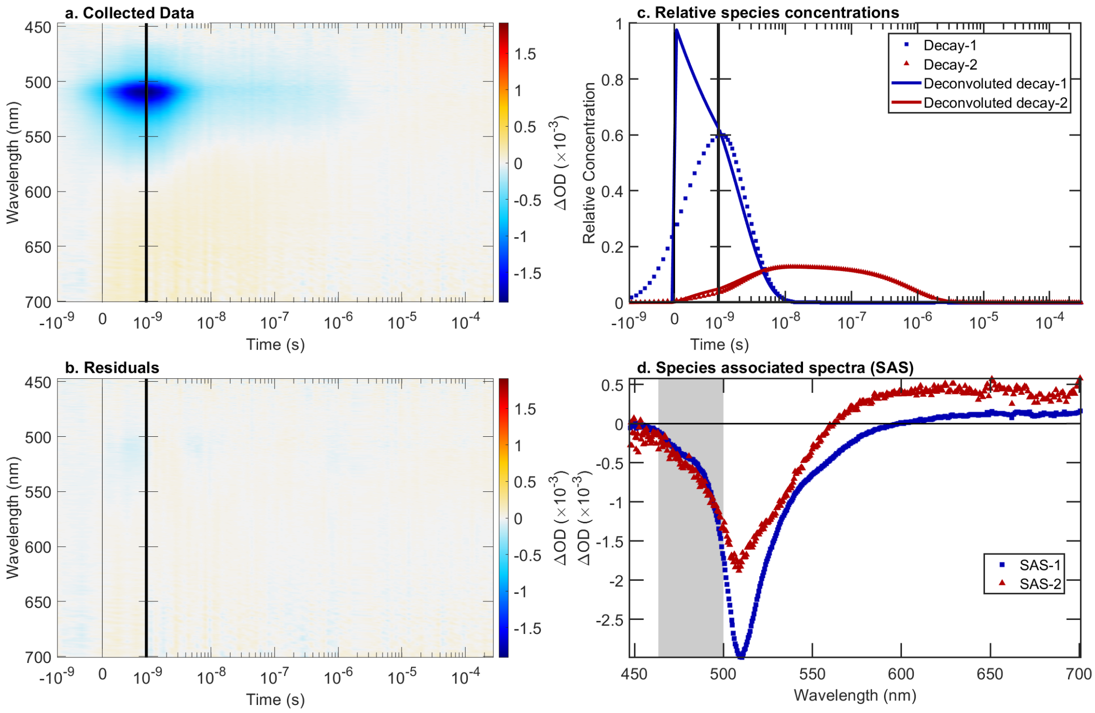

Figure S30: TA measurements (using a 355 nm excitation wavelength) of Ir(I)-BDP HS **6** with oxygen present in the time range of 0 – 10^{-4} s. a) Collected data, b) residual, c) modelled concentrations, d) species associated spectra.

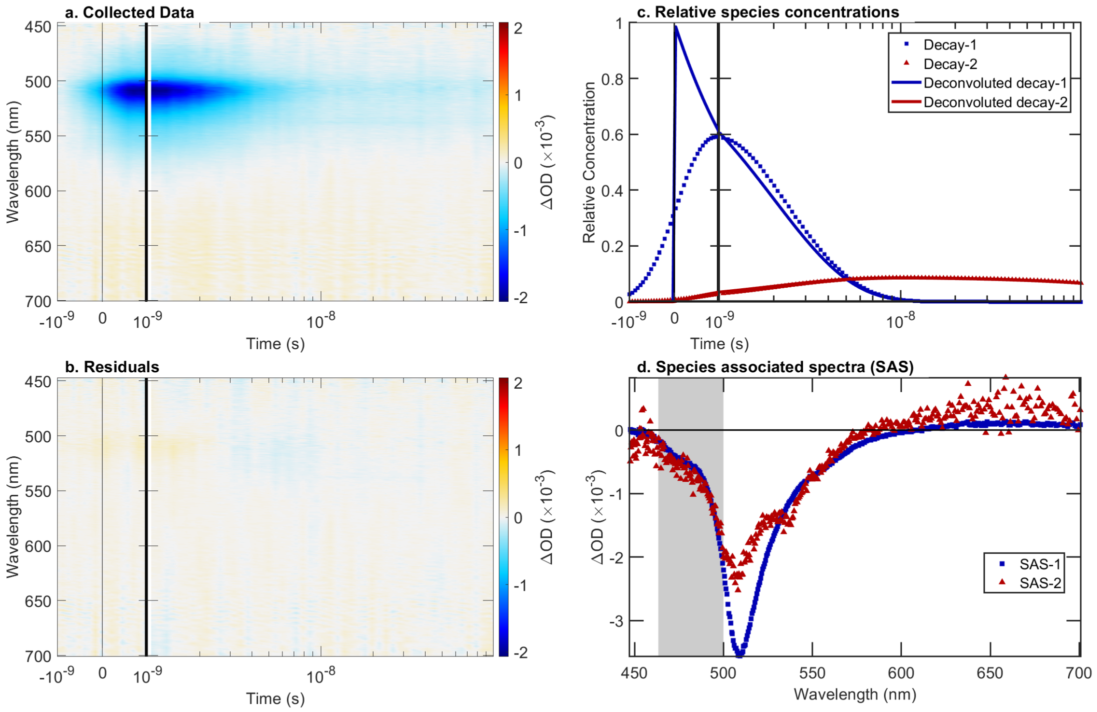

Figure S31*:* TA measurements (using a 355 nm excitation wavelength) of Ir(I)-BDP HS **6** without oxygen present in the time range of 0 – 10^{-8} s. a) Collected data, b) residual, c) modelled concentrations, d) species associated spectra.

*Ir(III)-BDP SS **7***

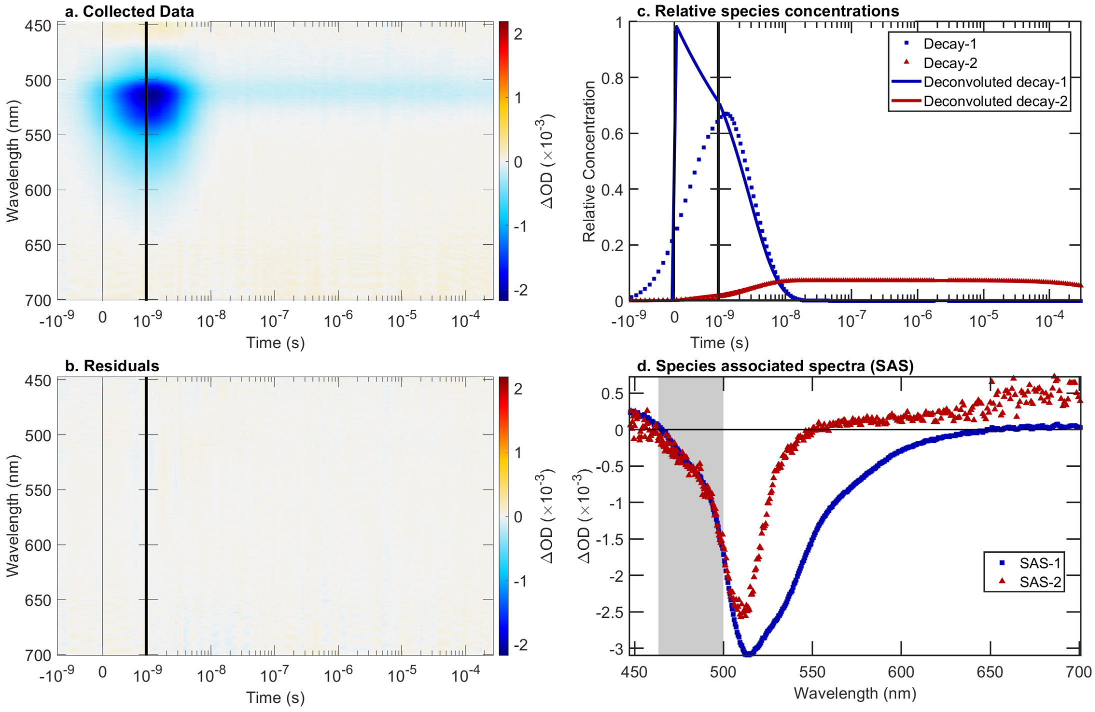

Figure S32: TA measurements (using a 355 nm excitation wavelength) of Ir(III)-BDP SS **7** without oxygen present in the time range of 0 – 10^{-4} s. a) Collected data, b) residual, c) modelled concentrations, d) species associated spectra.

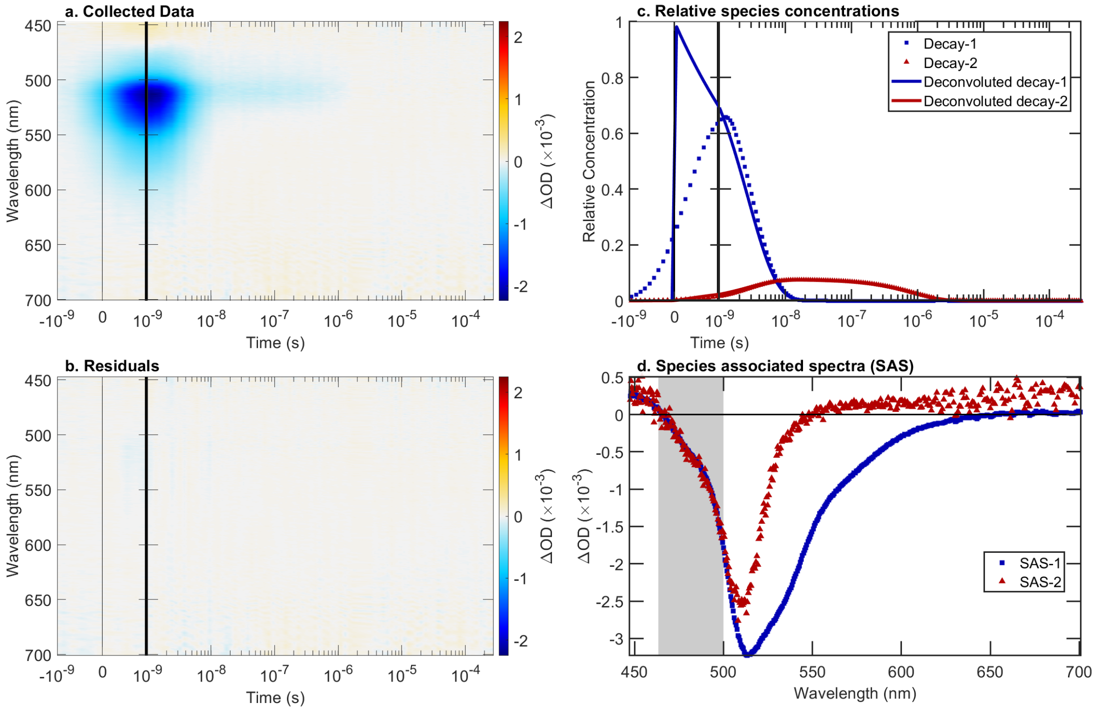

Figure S33: TA measurements (using a 355 nm excitation wavelength) of Ir(III)-BDP SS **7** with oxygen present in the time range of 0 – 10^{-4} s. a) Collected data, b) residual, c) modelled concentrations, d) species associated spectra.

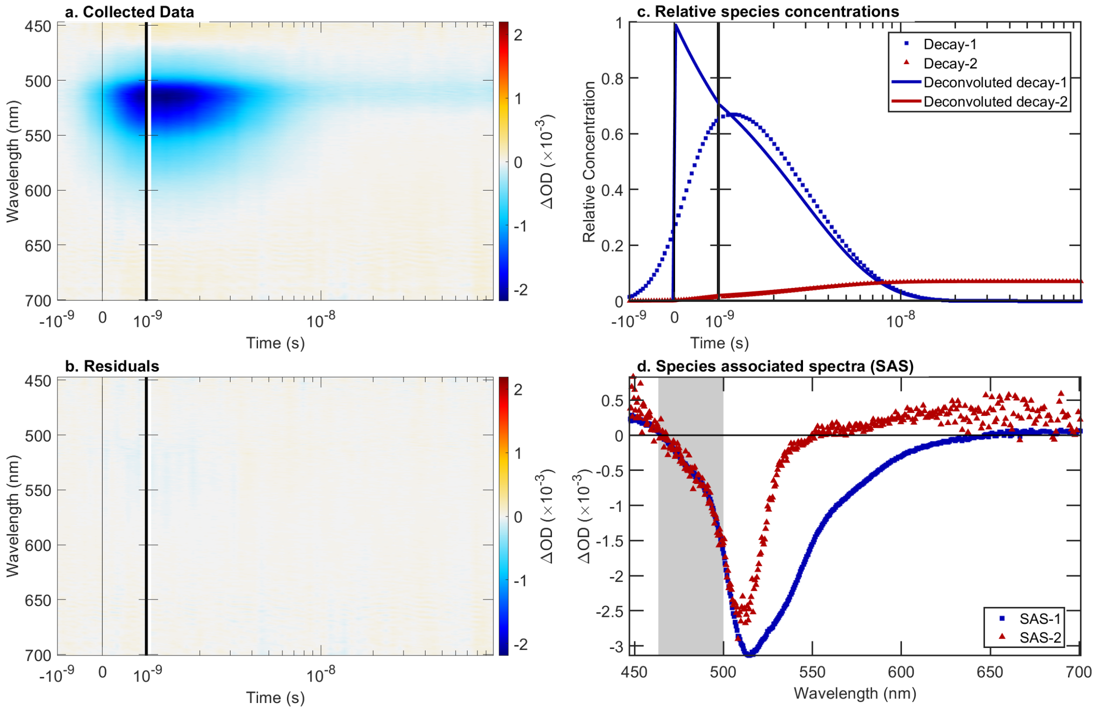

Figure S34: TA measurements (using a 355 nm excitation wavelength) of Ir(III)-BDP SS **7** without oxygen present in the time range of 0 – 10^{-8} s. a) Collected data, b) residual, c) modelled concentrations, d) species associated spectra.

*Ir(III)-BDP HH **8***

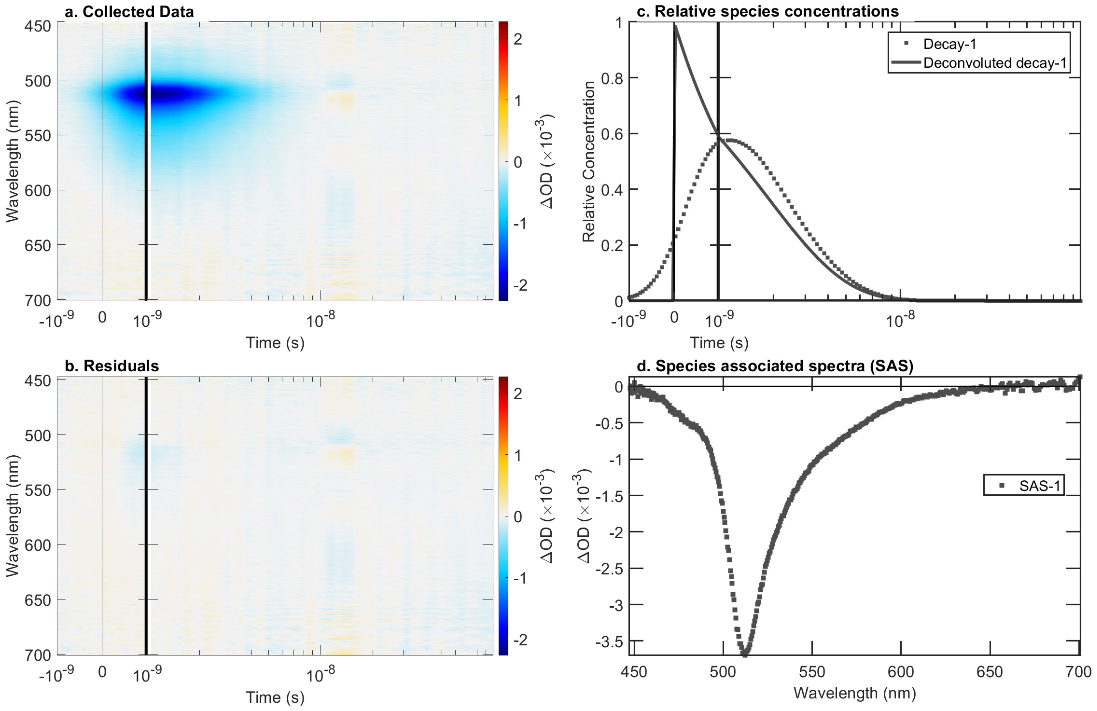

Figure S35: TA measurements (using a 355 nm excitation wavelength) of Ir(III)-BDP HH **8** without oxygen present in the time range of 0 – 10^{-8} s. a) Collected data, b) residual, c) modelled concentrations, d) species associated spectra.

*Ir(III)-BDP HS **9***

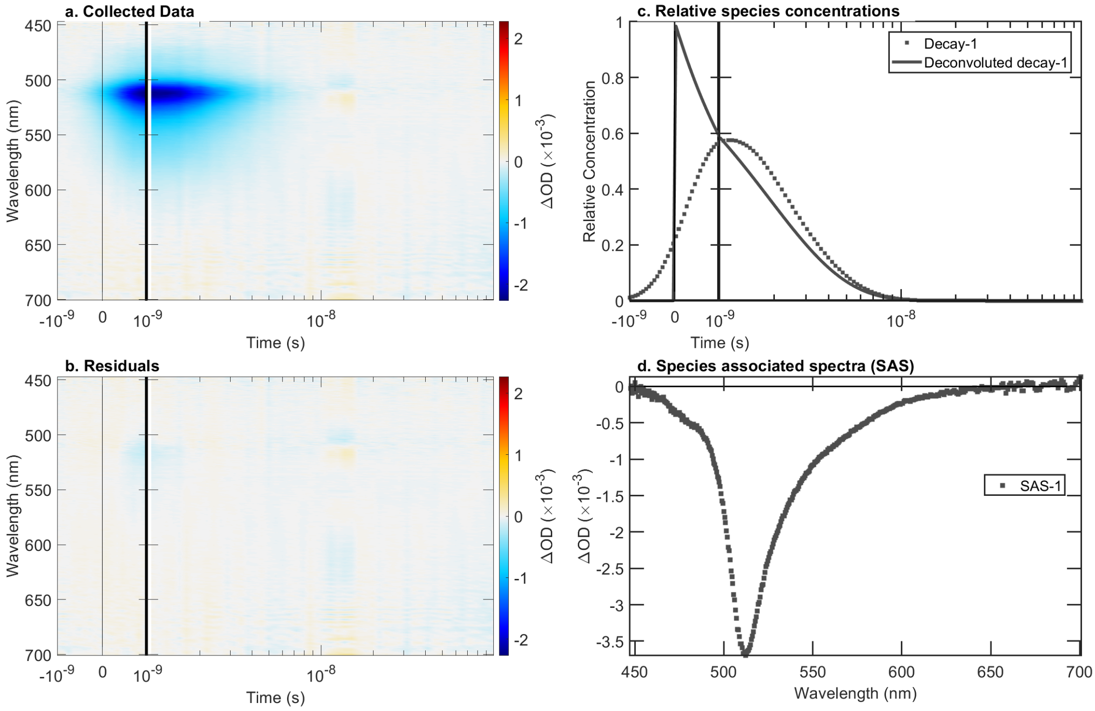

Figure S36: TA measurements (using a 355 nm excitation wavelength) of Ir(III)-BDP HS **9** without oxygen present in the time range of 0 – 10^{-8} s. a) Collected data, b) residual, c) modelled concentrations, d) species associated spectra.

Control experiments of the TA measurements

Due to the low yield of ISC in BDP **1**, and the mixtures BDP **1**+Ir(I) **2** and BDP **1**+Ir(III) **3**, deconvolution of the yield was not possible, instead, an upper bound for the triplet yield (ISC) was estimated. The ISC yield was estimated by comparing the spectral intensity in the 460 – 500 nm region, specifically, the ratio was taken of the time point with maximum amplitude, and at 60 ns. This calculation is summarized in Table S4. A correction for the IRF is made to the values under the heading "overestimate of ISC_2", this involved scaling the values by 0.72 to account for the change in maximum GSB amplitude caused by the singlet lifetime being on a similar timescale to the excitation laser pulse.

Table S4*:* The calculations of ISC yields of BDP **1**, and the mixtures BDP **1**+Ir(I) **2** and BDP **1**+Ir(III) **3**

	**Spectrum Scalar**		**Overestimate of ISC**		**Overestimate of ISC_2**	
BDP **1**	**no O2**	**with O2**	**no O2**	**with O2**	**no O2**	**with O2**
BDP **1** + Ir(I) **2**	59	36	1.69%	2.78%	1.22%	2.00%
BDP **1** + Ir(III) **3**	53	42.7	1.89%	2.34%	1.36%	1.69%

For each dataset the analysis is presented in Figures S37 - 44 below. On the left (a) is the decay of the GSB region (490 nm) as solid grey squares and an exponential decay with a lifetime of 3.7 ns shown as a red line, from this it is clear that the singlet state has decayed well before the 60 ns spectra used to estimate the ISC yield. On the right (a) are the two spectra used for the comparison, the grey shaded area represents the region where the amplitude has been used to estimate ISC yield.

BDP ***1***

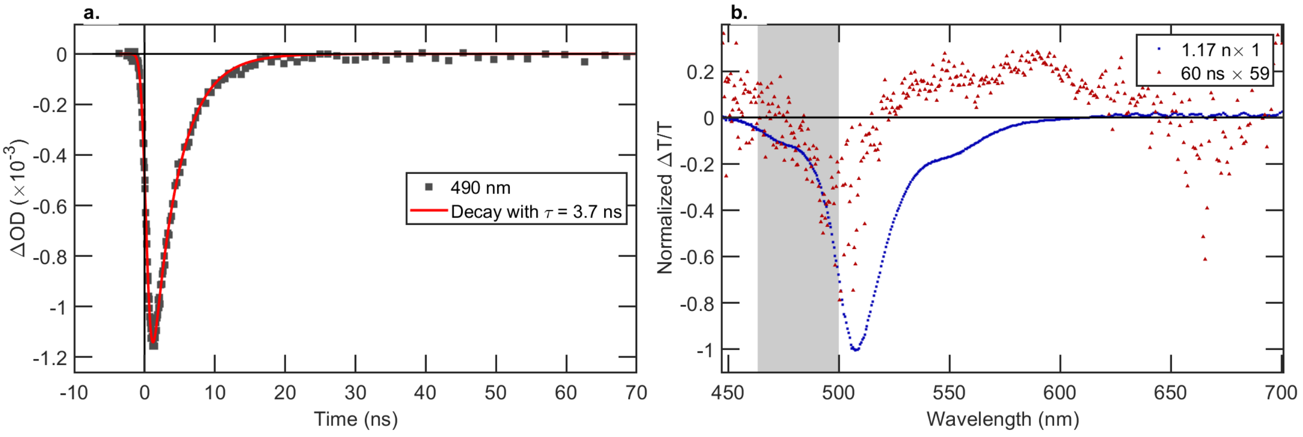

Figure S37: TA measurements (using a 355 nm excitation wavelength) of BDP **1** without oxygen present in the time range of 0 – 70 ns. a) Modelled concentrations, b) species associated spectra.

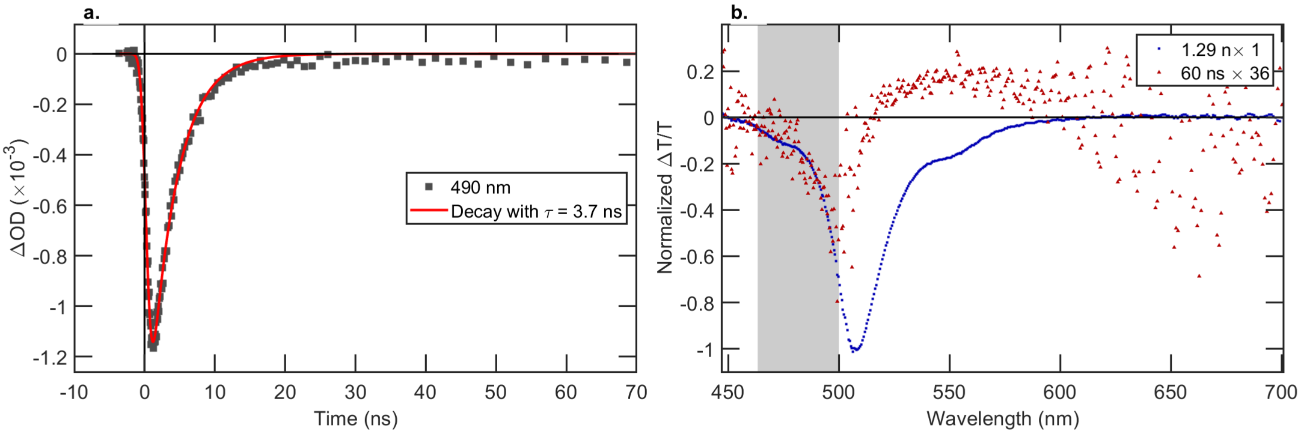

Figure S38: TA measurements (using a 355 nm excitation wavelength) of BDP **1** with oxygen present in the time range of 0 – 70 ns. a) Modelled concentrations, b) species associated spectra.

*BDP **1** + Ir(I) **2***

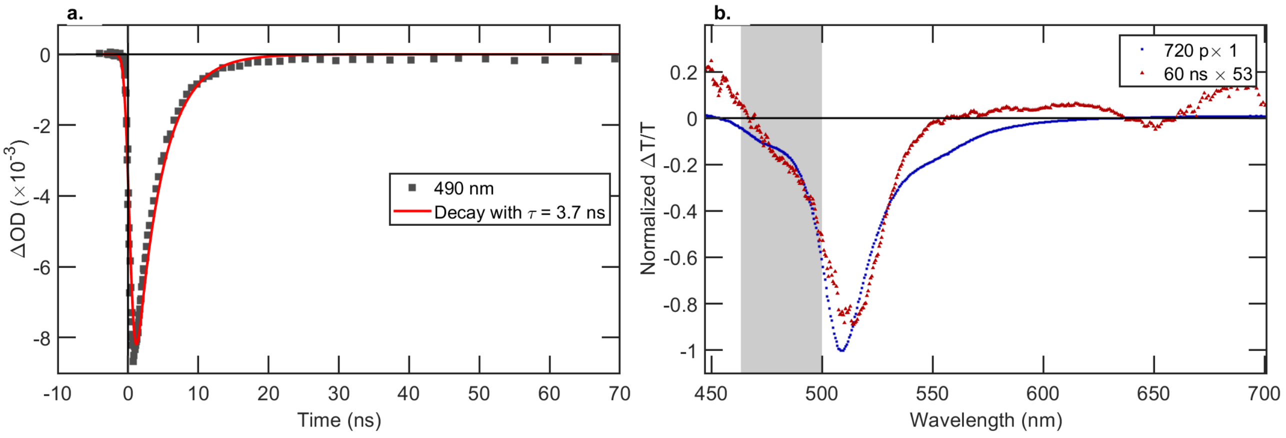

Figure S39: TA measurements (using a 355 nm excitation wavelength) of BDP **1** + Ir(I) **2** without oxygen present in the time range of 0 – 70 ns. a) Modelled concentrations, b) species associated spectra.

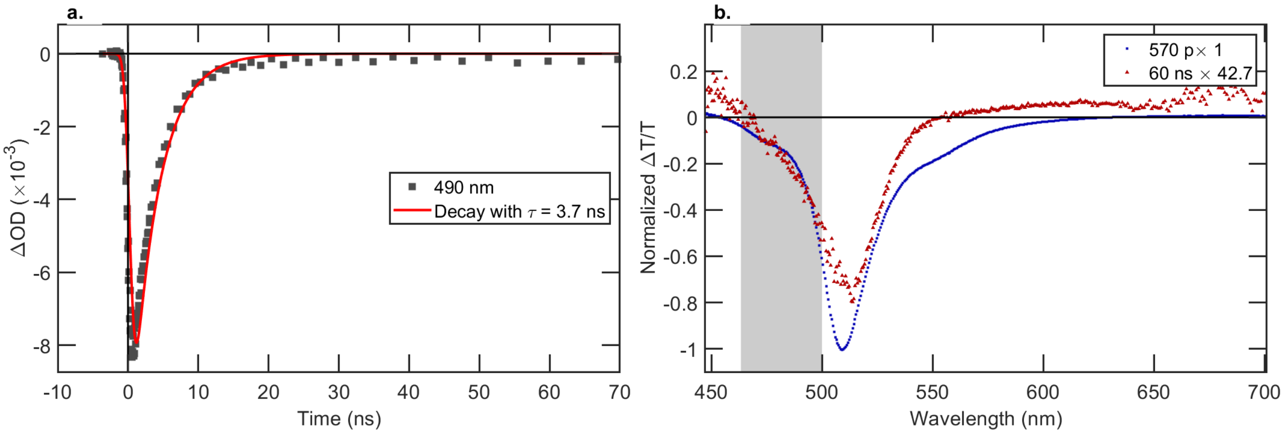

Figure S40: TA measurements (using a 355 nm excitation wavelength) of BDP **1** + Ir(I) **2** with oxygen present in the time range of 0 – 70 ns. a) Modelled concentrations, b) species associated spectra.

*BDP **1** + Ir(III) **3***

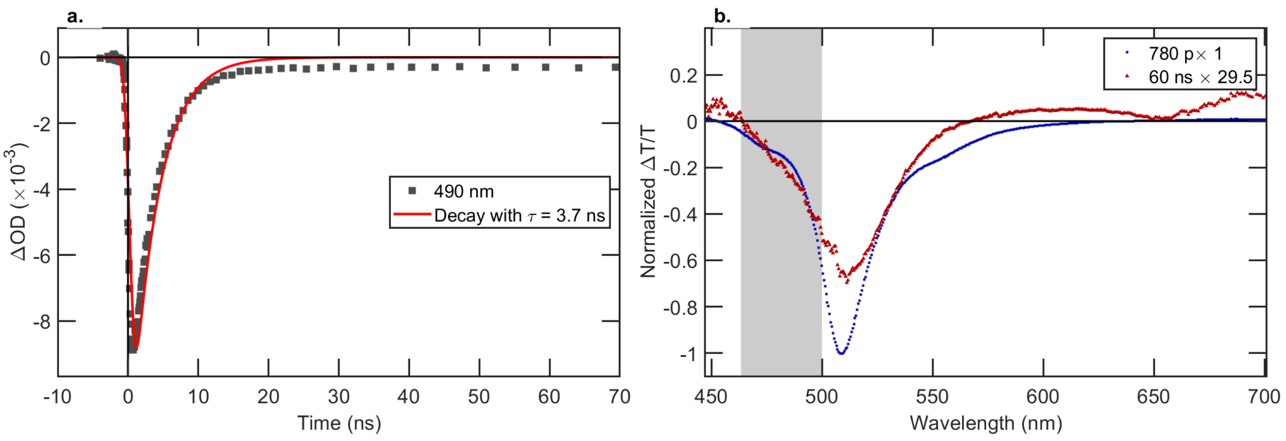

Figure S41: TA measurements (using a 355 nm excitation wavelength) of BDP **1** + Ir(III) **3** without oxygen present in the time range of 0 – 70 ns. a) Modelled concentrations, b) species associated spectra.

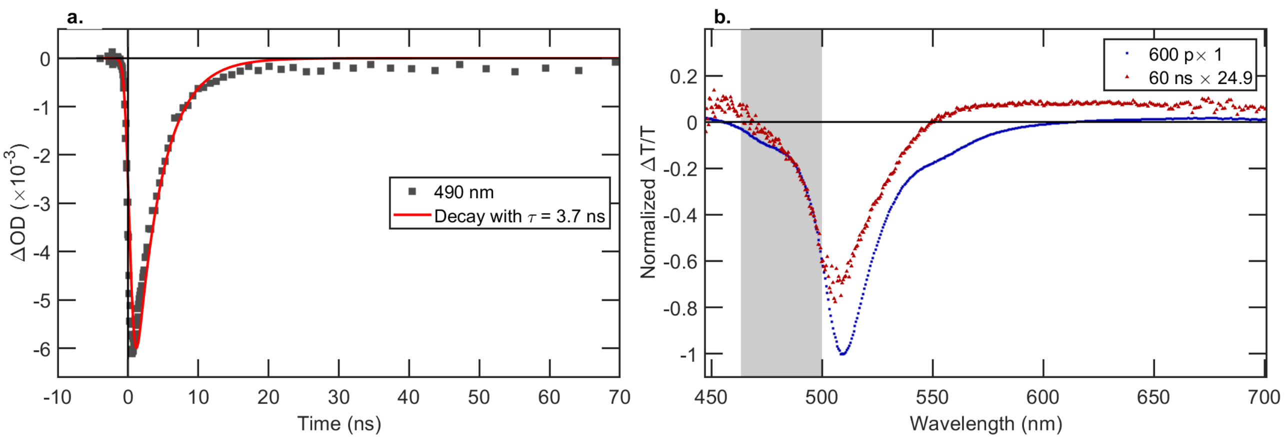

Figure S42: TA measurements (using a 355 nm excitation wavelength) of BDP **1** + Ir(III) **3** with oxygen present in the time range of 0 – 70 ns. a) Modelled concentrations, b) species associated spectra.

Comparison of TA spectra when either a 355 nm or 510 nm excitation wavelength was used

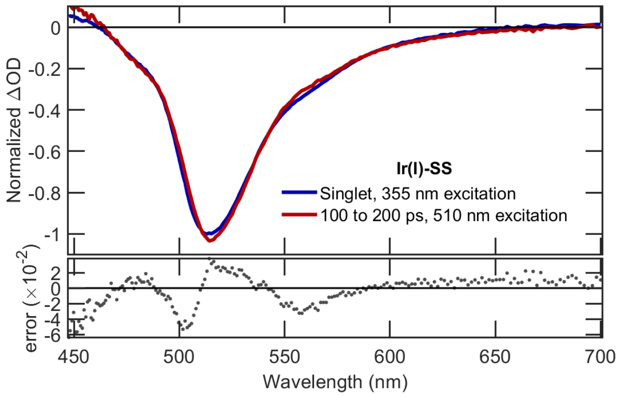

Figure S43: Comparison of the TA data for Ir(I)-BDP SS **4** when either a 355 nm or 510 nm excitation wavelength was used. This data confirms that the nature of the excited state species are the same when a 355 nm or 510 nm excitation is used, and thus Kasha's rule is obeyed.

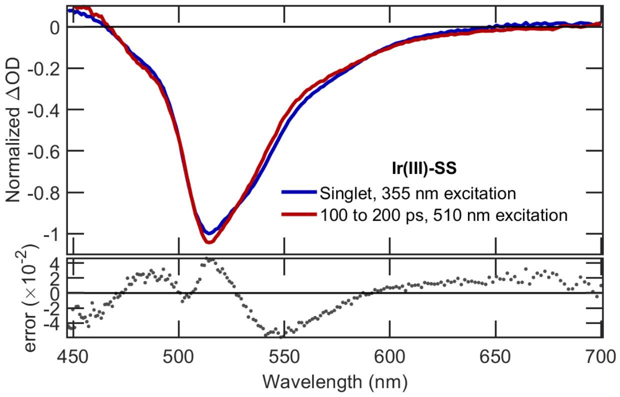

Figure S44: Comparison of the TA data for Ir(III)-BDP SS **7** when either a 355 nm or 510 nm excitation wavelength was used. This data confirms that the nature of the excited state species are the same when a 355 nm or 510 nm excitation is used, and thus Kasha's rule is obeyed.

Cyclic Voltammetry

The cyclic voltammetry data of ligands SS **13**, HH **17** and HS **14**, with the parent BDP **1** compound included for comparison (Figure S45). These data suggest that: 1) the main redox events are dominated by the BDP **1** moiety in the ligand, and 2) the side-side linking mode alters the redox potentials of the ligand (SS **13**), relative to BDP **1**, whereas the head-head (HH **17**) and head-side (HS **14**) tethering modes have minor effects on electrochemical behaviour of the ligands.

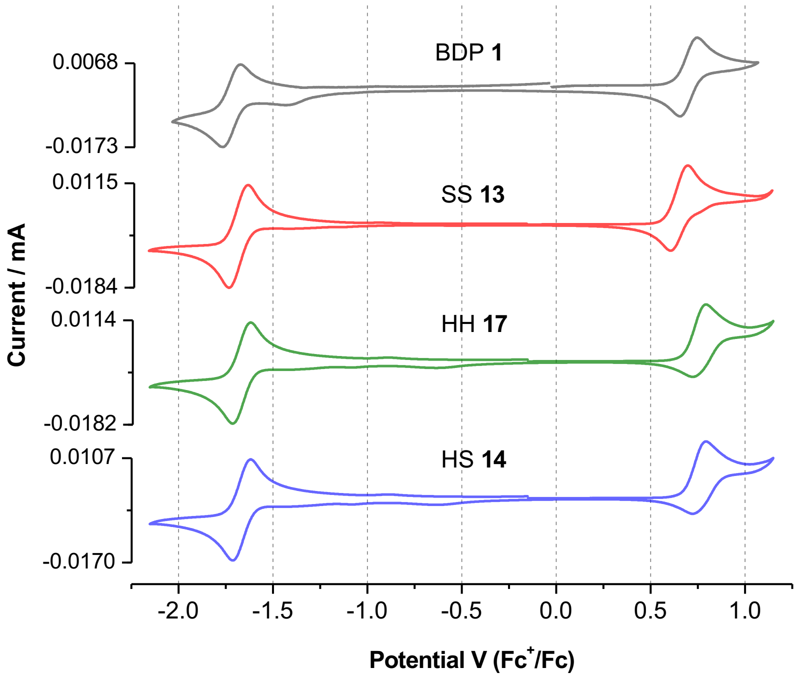

Figure S45: Cyclic voltammograms of BDP **1** and the ligands **13**, **14**, **17**, measured in a 0.1 M solution of TBA-BAr^F_4 in dichloromethane under argon. Ferrocene was used as internal standard. Scan rate: 100 mV/s.

Table S5: The scan rate dependence of the cyclic voltammograms for the bifunctional catalysts **4-9**.

Ir(I) bifunctional catalysts	Ir(III) bifunctional catalysts
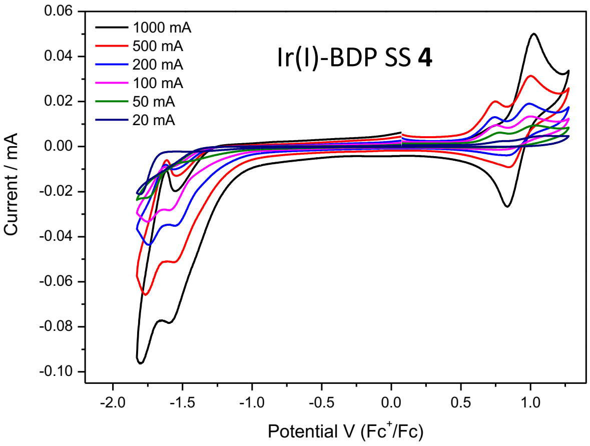	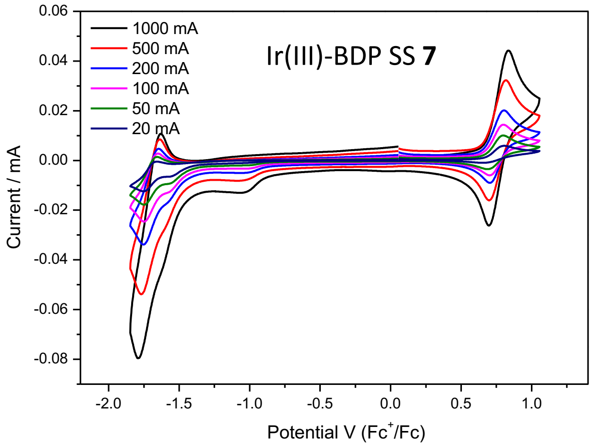
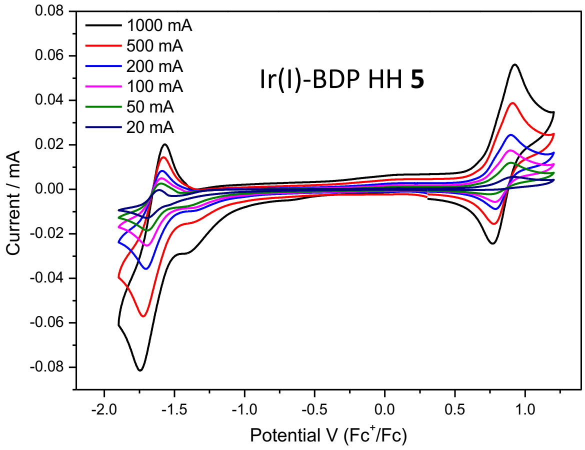	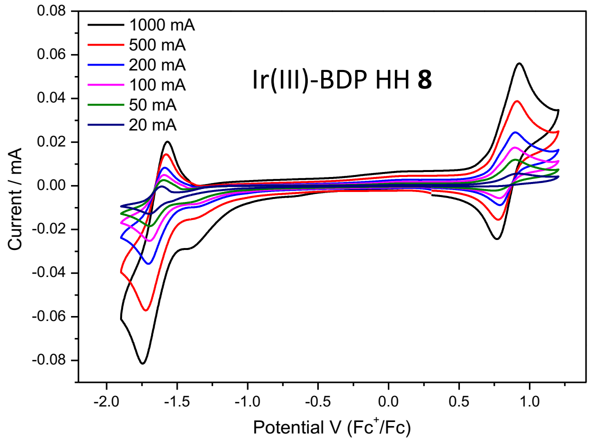
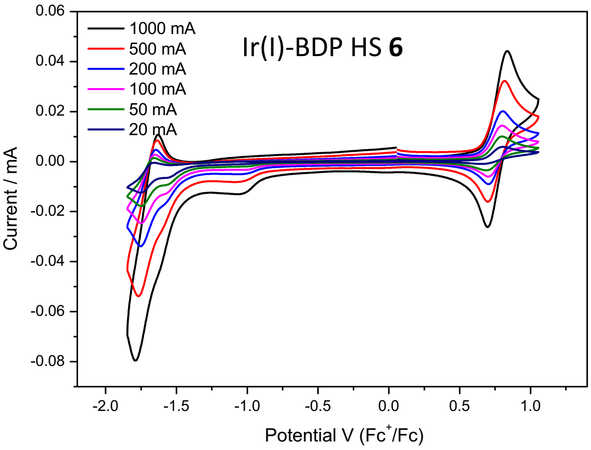	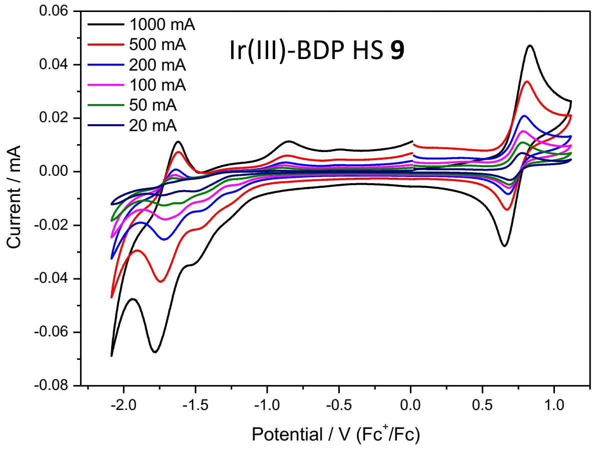

Table S6: The scan rate dependence of the cyclic voltammograms for the ligands **13**, **14**, **17** and parent catalysts **1**-**3**.

Ligands	Parent catalysts
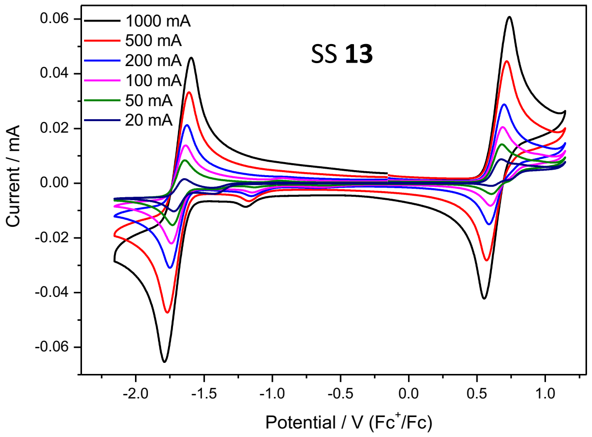	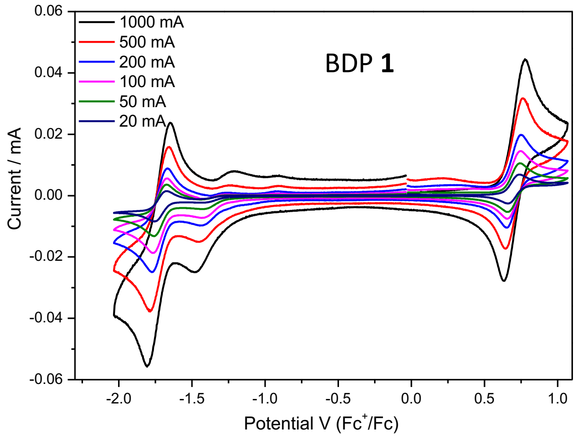
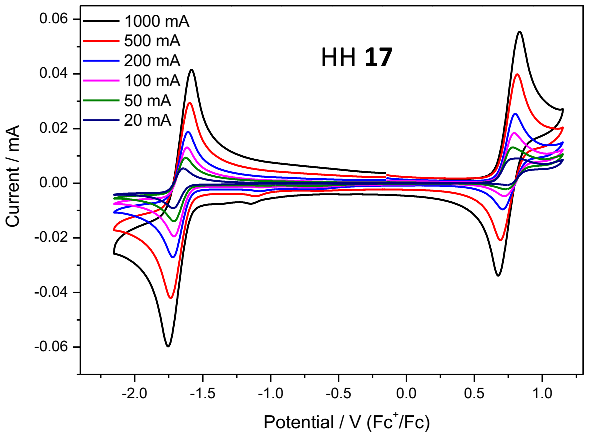	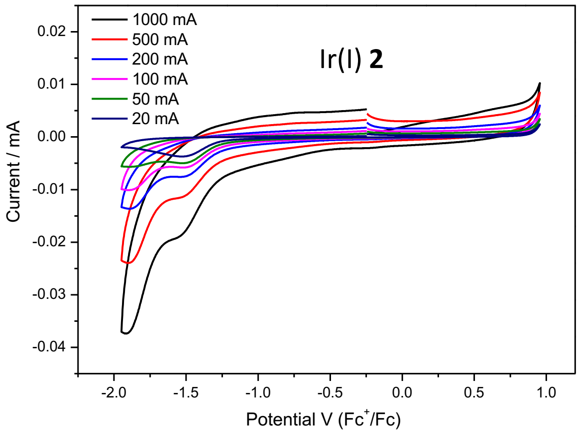
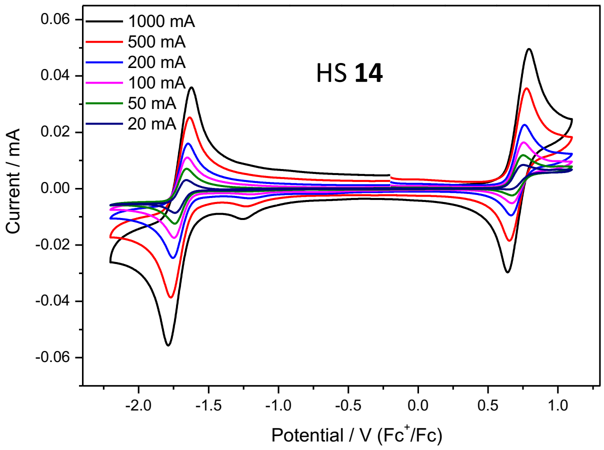	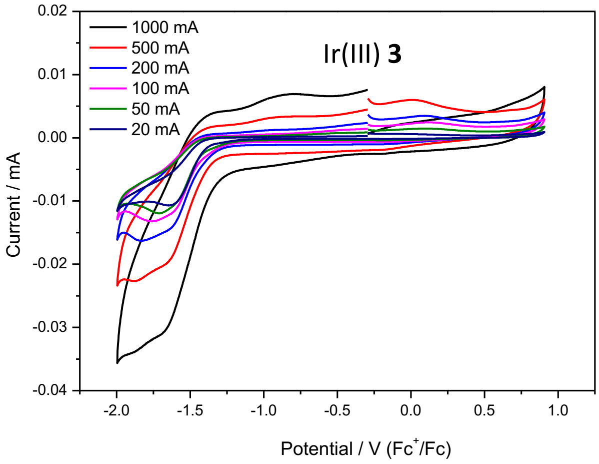

X-Ray Absorption Spectroscopy

The solid state XAS measurements were performed at the Australian Synchrotron using the 'XAS Beamline' at the Ir L_3 edge (*ca.* 11.2 keV) in transmission mode, using a Si(111) monochromator and a 1.9T Wiggler. Data analysis was performed using the IFEFFIT data analysis package (Demeter),[5] using the Athena program for data reduction and generation of the plots in E-space (Figures 8 and 9), R-space and k-space (Figure S46 and Figure S47). The Artemis program was used for further data analysis, and comparison of the obtained XAS data with reported crystal structures.

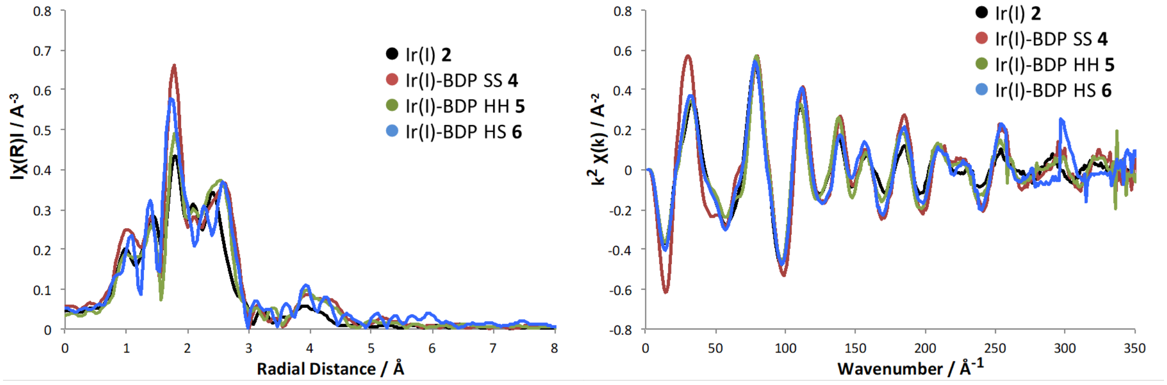

Figure S46: The XAS data for the Ir(I) based complexes **2**, **4**, **5** and **6** plotted in 'R-space' (left) and 'k-space' (right).

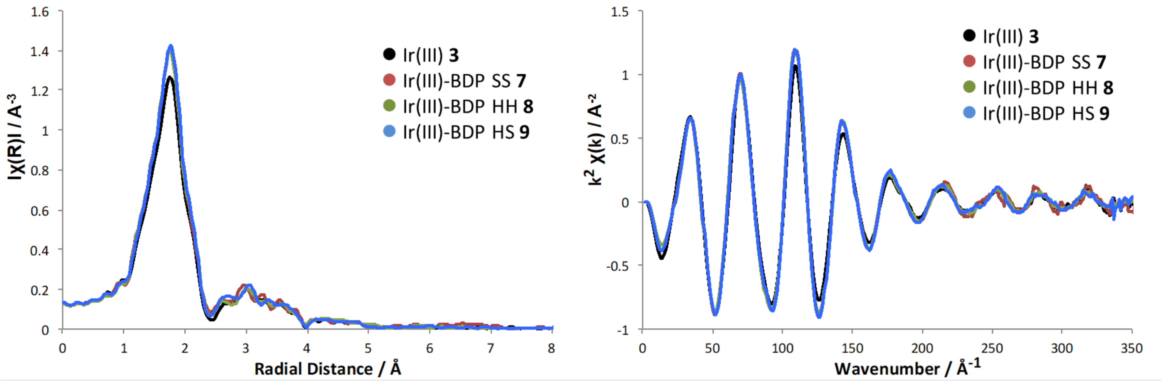

Figure S47: The XAS data for the Ir(III) based complexes **3**, **7**, **8** and **9** plotted in 'R-space' (left) and 'k-space' (right).

Detailed data analysis was performed, using Artemis, on the complexes Ir(I)-BDP HH **5** and Ir(III) **3**, to confirm that the XAS data matches with the predicted XAS data based on the crystallography data. In particular, our XAS data for Ir(I)-BDP HH **5** was compared with that predicted from the crystal structure of **5**, and our XAS data for Ir(III) **3** compared with that predicted for the reported structure of Ir(III) **3**.[8] The measured XAS data for Ir(I)-BDP HH **5** and the predicted XAS data based on the crystal structure match quite well (Figure S48), with good correlation between the predicted and measured peak positions. A number of different scattering paths were predicted from the crystal structure, with the paths that contribute most to the predicted XAS data shown in Figure S48. The peaks around 1-2 Å are mainly due to scattering off the atoms directly attached to the Ir centre (N and C(O)), however there are also peaks at higher radial distance 2-3 Å due to scattering from C(O) and O(C).

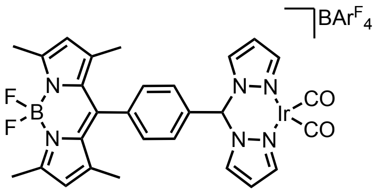

Ir(I)-BDP HH **5**

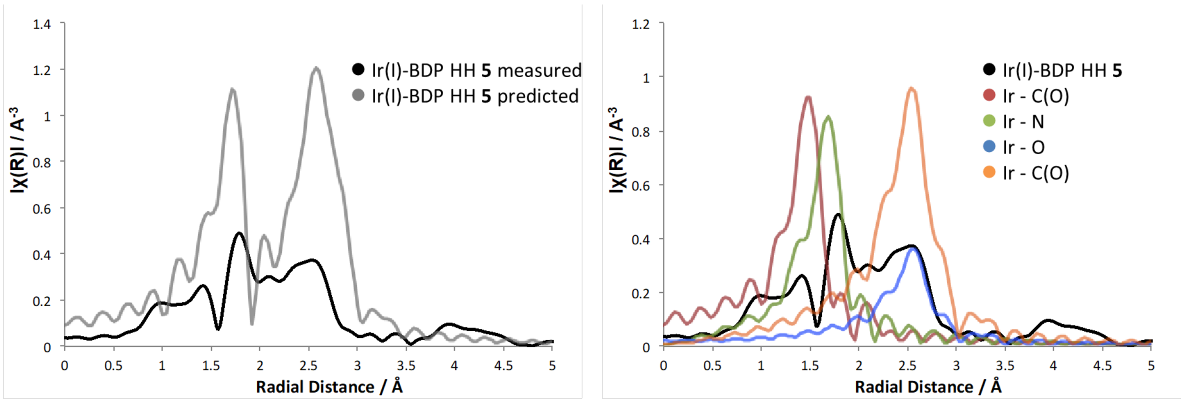

Figure S48: Left: Comparison of the measured XAS data for Ir(I)-BDP HH **5** (black) and that predicted from the crystal structure (grey). Right: Comparison of the measured XAS data for Ir(I)-BDP HH **5** (black) and some of the scattering paths due to different neighbouring atoms, predicted from the crystal structure.

The measured XAS data for Ir(III) **3** and the predicted XAS data based on the reported crystal structure[8] also match quite well (Figure S49), with good correlation between the predicted and measured peak positions. A number of different scattering paths were predicted from the crystal structure, with the paths that contribute most to the predicted XAS data shown in Figure S49. The main broad peak near 1.6 Å is due to a combination of the scattering paths due to Ir - N and Ir - C(Cp*), with the shoulder near 2 Å due to Ir – Cl.

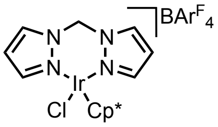

Ir(III) **3**

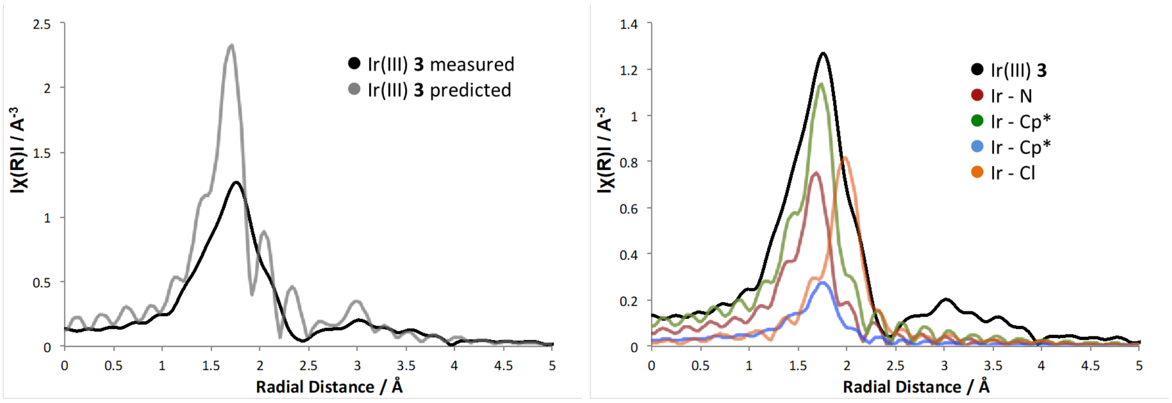

Figure S49: Left: Comparison of the measured XAS data for Ir(III) **3** (black) and that predicted from the crystal structure (grey). Right: Comparison of the measured XAS data for Ir(III) **3** (black) and some of the scattering paths due to different neighbouring atoms, predicted from the crystal structure.

Catalytic Investigations and Control Reactions

Photocataysis set-up

The photocatalytic reactor was simply made up by wrapping the LED strips around a 150 ml crystallising dish. The reaction vials are centred in the middle of the reactor, with an estimated distance of 4 cm to the edge of the reactor, as 2-4 vials were generally irradiated at the same time.

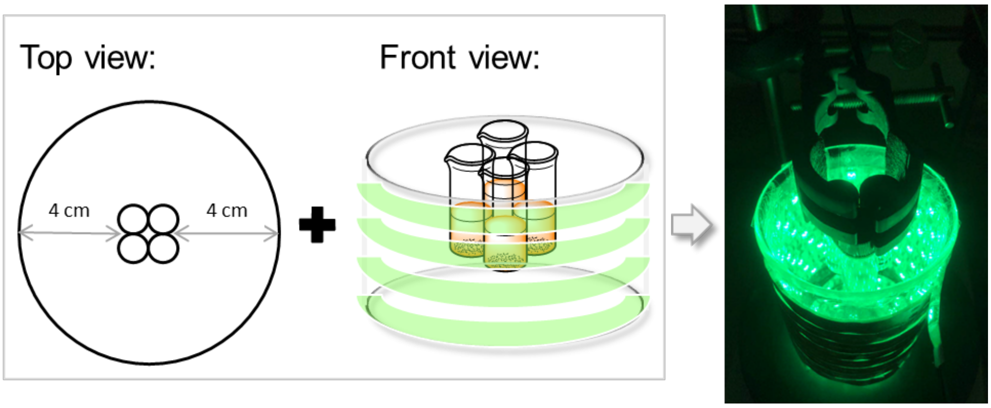

Figure S50: The self-made photoreactor used for the photocatalysis experiments.

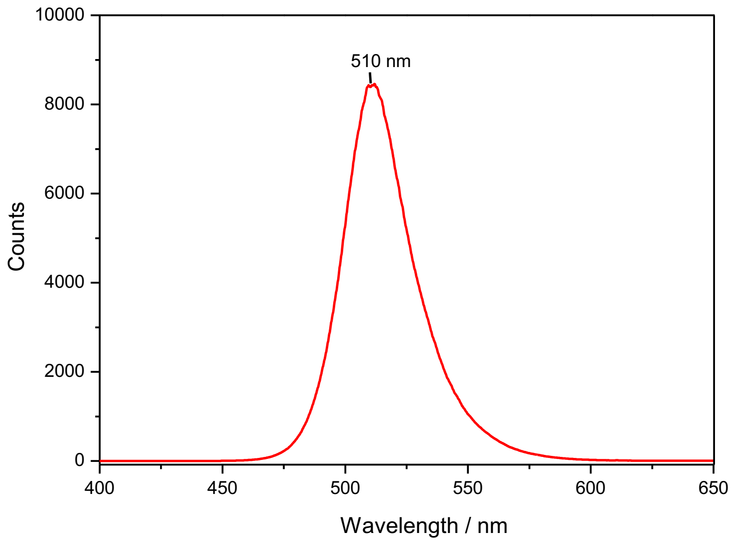

Figure S51: The measured spectrum for the green LED used in the photocatalysis experiments and the measurements of the singlet oxygen quantum yields.

Phooxidation of benzylamine

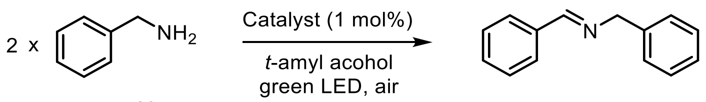

18 **19**

Procedure: benzylamine (42.8 mg, 0.4 mmol), catalyst (0.002 mmol), 2,4,6-trimethoxybenzene (internal standard, 33.6 mg, 0.2 mmol) were added into a 4 mL transparent vial and dissolved in 0.5 mL of *t*-amyl alcohol and the vial was left open to air which was then irradiated under green LED. Aliquots were taken from the reaction mixture at different time points and the conversion to product was calculated with respect to the internal standard. The results of control experiments are summarised in the following table. The ^{1}H NMR spectra showing the starting material **18** and product **19** are presented below (Figure S52).

The NMR signals used to monitor reaction progress

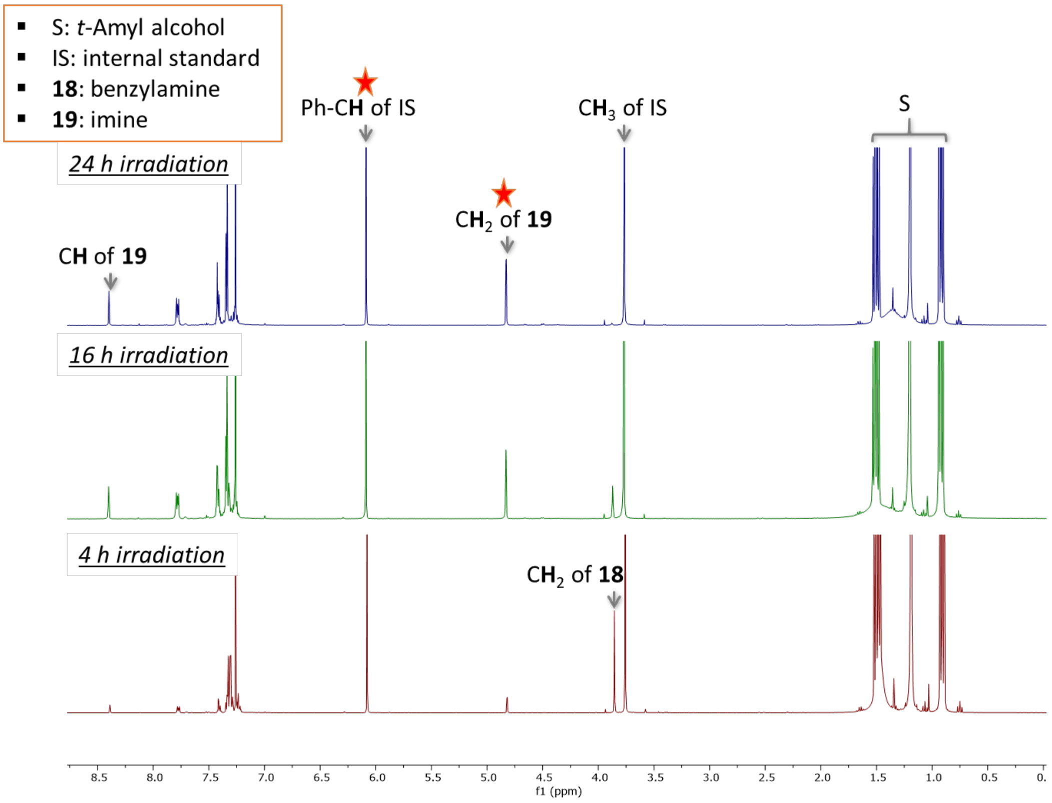

Figure S52: ^{1}H NMR spectra of the photooxidation of benzylamine promoted by Ir(I)-BDP SS **4** at different time points (4 hours, 16 hours and 24 hours). The conversions were calculated based on the ratio of the Ph-C<u>H</u> of the internal standard and the C<u>H</u>$_2$ of **19**, as indicated by the red star. Other distinct peaks of starting material **18** and product **19** were marked and assigned in the spectra.

Control reactions using untethered mixtures

To further investigate the origin of the rate enhancements observed for the photooxidation of benzylmaine **18** using tethered dual catalysts, control experiments using untethered mixtures and different additives were performed. The results suggest that the cationic Ir centre in promoting the photooxidation of benzylamine **18**, when it is presented in the reaction mixture with BDP **1**.

Procedure: benzylamine (42.8 mg, 0.4 mmol), BDP (0.002 mmol), Ir catalyst or additive (0.002 mmol), 2,4,6-trimethoxybenzene (internal standard, 33.6 mg, 0.2 mmol) were added into a 4 mL transparent vial and dissolved in 0.5 mL of *t*-amyl alcohol and the vial was left open to air which was then irradiated under green LED. Aliquots were taken from the reaction mixture at different time points and the conversion to product was calculated with respect to the internal standard. Reactions were performed in duplicate/triplicate.

Table S7: Control reactions using untethered mixtures.

Catalyst	Conversion to product **19** / %								
	4 h			16 h			24 h		
BDP **1** + Ir(I) **2**	12	12		42	42		60	57	
BDP **1** + Ir(III) **3**	11	12		38	44		54	59	
BDP **1** + NaBAr^{F}4	8	12	5	25	26	22	42	45	39
BDP **1** + NaCl	2	7	8	19	22	23	33	36	37

Control reactions using a singlet oxygen scavenger

To determine whether the photooxidation of benzylmaine **18** involves singlet oxygen, control experiments with the singlet oxygen scavenger 1,4-diazabicyclo[2.2.2]octane (DABCO) were performed. The results clearly demonstrated that the photooxidation of compound **18** involves singlet oxygen.

Procedure: benzylamine (42.8 mg, 0.4 mmol), catalyst (0.002 mmol), DABCO (24.7 mg, 0.22 mmol), 2,4,6-trimethoxybenzene (internal standard, 33.6 mg, 0.2 mmol) were added into a 4 mL transparent vial and dissolved in 0.5 mL of *t*-amyl alcohol and the vial was left open to air which was then irradiated under green LED for 24 hours. Conversion to product **19** was calculated with respect to the internal standard. Reactions were performed only once.

Table S8: Control reactions using the singlet oxygen scavenger DABCO.

Catalyst	Conversion to product **19** / %
BDP **1**	2
Ir(I)-BDP SS **4**	6
Ir(I)-BDP HH **5**	4
Ir(III)-BDP SS **7**	5

The correlation between singlet oxygen quantum yield and conversion to the product 19

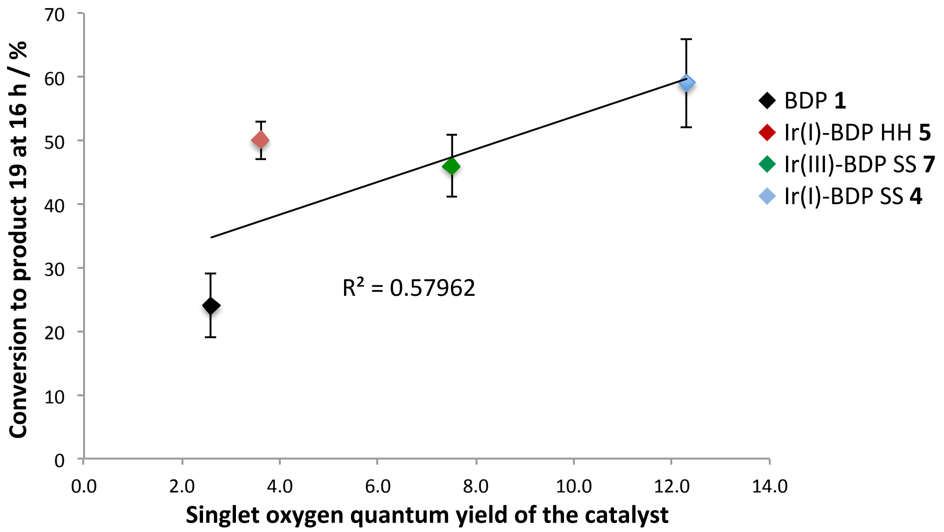

Figure S53: The correlation between the singlet oxygen quantum yield of the catalyst and the extent of conversion to the product **19**, with the 16 hour time point chosen as a representative example.

Dihydroalkoxylation

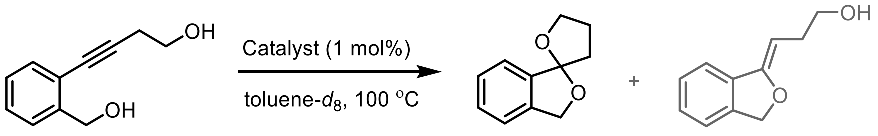

20 **21** **22**

Procedure: substrate **20** (35.2 mg, 0.2 mmol), catalyst (0.002 mmol), toluene-d_8 (0.5 mL) were added to a J. Young's tube in a glovebox under argon. The reaction was monitored by *in situ* NMR spectroscopy at 100 °C. Conversion to the products **21** and **22** was calculated relative to the starting material **20**. The ^{1}H NMR spectra showing the starting material **20** and products **21** and **22** are presented below (Figure S54).

The NMR signals used to monitor reaction progress

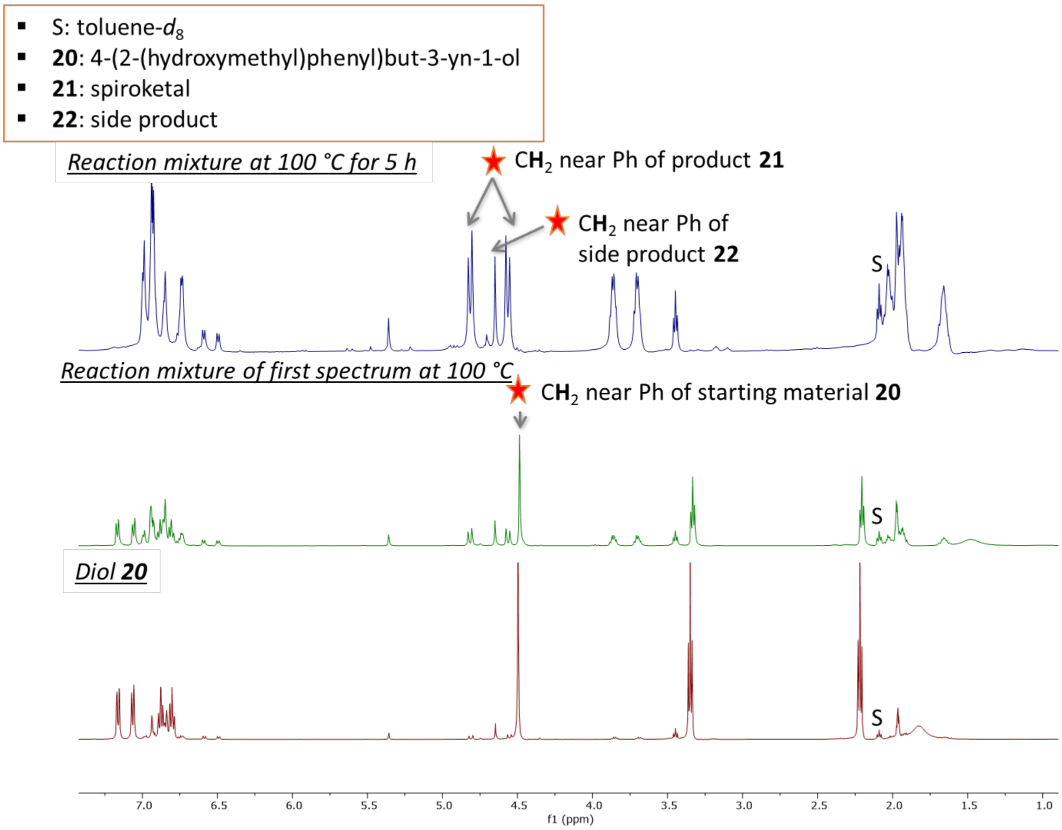

Figure S54: ^{1}H NMR spectra of the dihydroalkoxylation of compound **20** promoted by Ir(I)-BDP SS **4**, monitored using *in situ* NMR spectroscopy. The conversion to the products **21** and **22** was determined relative to the starting material **20**, with the specific signals used indicted by the red stars. The spectrum of starting material **20** is presented at the bottom for comparison.

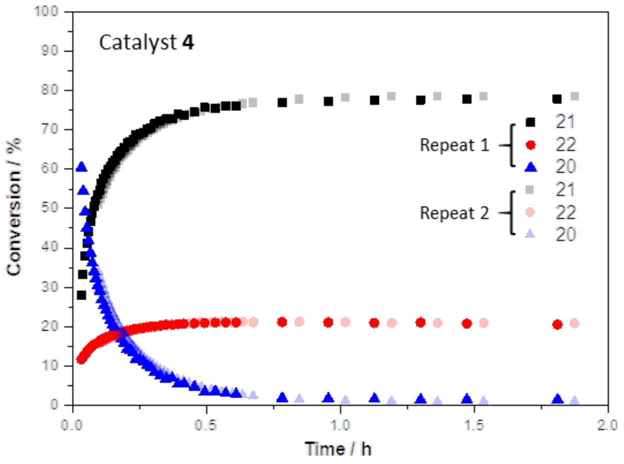

Figure S55: Reaction profiles for the intramolecular hydroamination catalysed by Ir(I)-BDP SS **4**, monitored by *in situ* NMR spectroscopy, using the reaction conditions outlined above.

Control reactions

Procedure: substrate **20** (35.2 mg, 0.2 mmol), with catalyst (0.002 mmol) or without catalyst, toluene-d_8 (0.5 mL) were added to a J. Young's tube in a glovebox under argon. The reaction was heated at 100 °C and monitored by *in situ* NMR spectroscopy. Conversion to the products **21** and **22** was calculated relative to the starting material **20**.

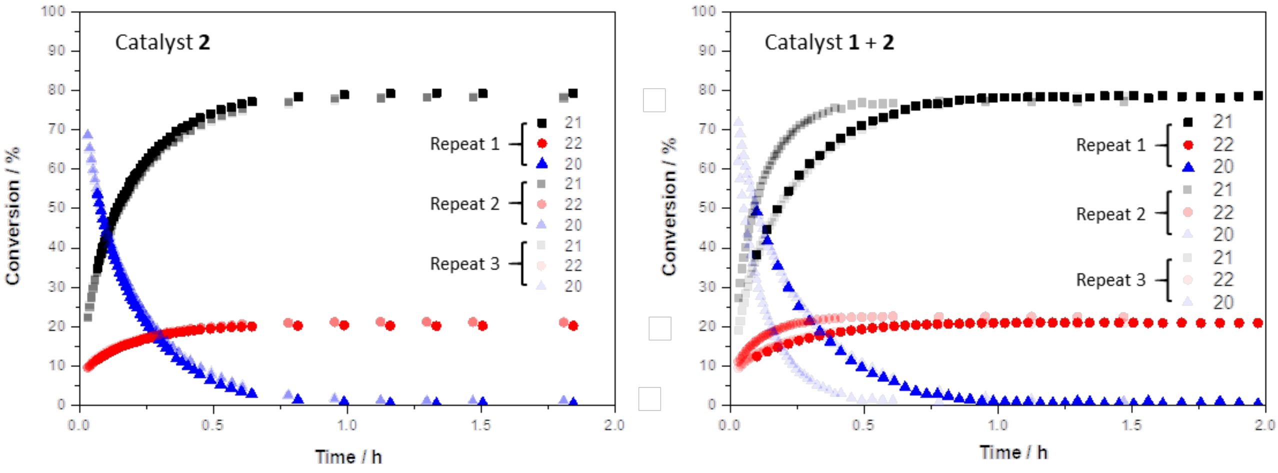

Figure S56: Reaction profiles for the intramolecular hydroamination catalysed by either Ir(I) **2** (left) or a 1:1 mixture of BDP **1** + Ir(I) **2** (right), monitored by *in situ* NMR spectroscopy, using the reaction conditions outlined above. These control reactions were performed in triplicate.

To compare with Ir(III) based catalysts, control experiments using Ir(III) **3**, Ir(III)-BDP SS **7** and the 1:1 mixture of BDP **1** and Ir(III) **3** were conducted once. The corresponding results are summarised in the following table.

Table S9: Summarised control dihydroalkoxylation reactions using different catalysts, or no catalyst.

Catalyst	Conversion to the product **21** / %
	5 h
BDP **1**	5
Ir(I) **2**	78±0
Ir(III) **3**	5
Ir(I)-BDP SS **4**	78±0
Ir(III)-BDP SS **7**	4
Ir(I) **2** + BDP **1***	78±1
Ir(III) **3** + BDP **1***	13
No catalyst	5

*BDP **1** (0.002 mmol) and Ir(I) **2** (0.002 mmol) / Ir(III) **3** (0.002 mmol) was used.

These results suggest that Ir(III) based catalysts almost do not promote the dihydroalkoxylation reaction.

Intramolecular hydroamination

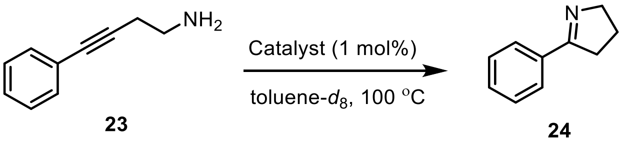

Procedure: substrate **23** (29.1 mg, 0.2 mmol), catalyst (0.002 mmol), toluene-d_8 (0.5 mL) were added into a J. Young's tube in a glovebox under argon. The reaction was heated at 100 °C and monitored by *in situ* NMR spectroscopy. Conversion to the product **24** was calculated relative to the starting material **23**.

The NMR signals used to monitor reaction progress

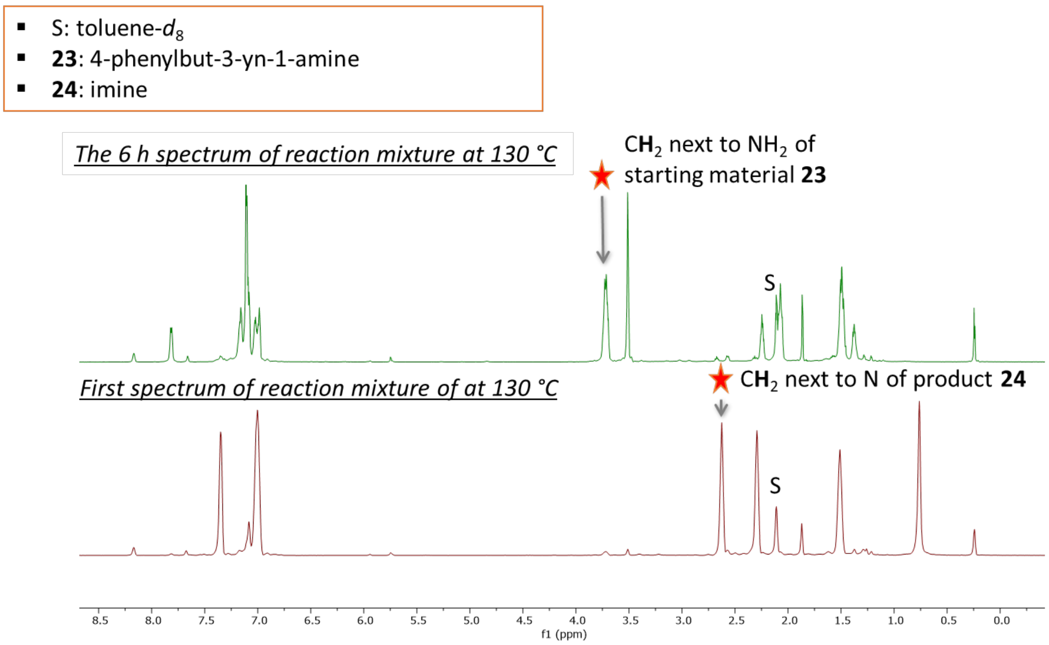

Figure S57: ^{1}H NMR spectra of the intramolecular hydroamination of compound **23** promoted by Ir(III)-BDP SS **7**, monitored by *in situ* NMR spectroscopy at 100 °C. Conversion to the product **24** was determined relative to the starting material **23**, with the specific signals used indicted by the red stars.

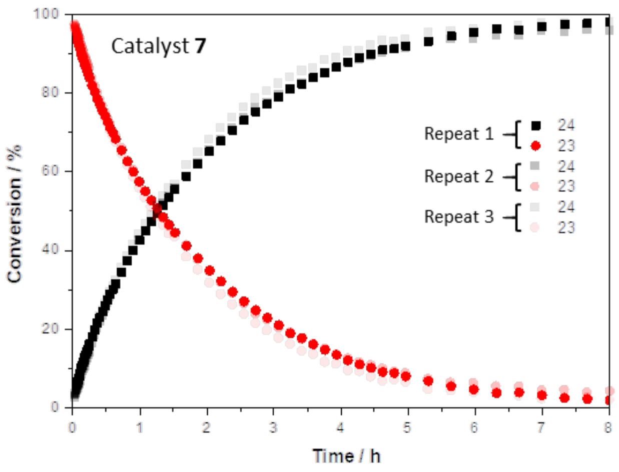

Figure S58: Reaction profiles for the intramolecular hydroamination catalysed by Ir(III)-BDP SS **7**, monitored by *in situ* NMR spectroscopy, using the reaction conditions outlined above.

Control reactions

Procedure: substrate **23** (29.1 mg, 0.2 mmol), with catalyst (0.002 mmol) or without catalyst, toluene-d_8 (0.5 mL) were added to a J. Young's tube in a glovebox under argon. The reaction was heated at 100 °C and monitored by *in situ* NMR spectroscopy. Conversion to the products **24** was calculated relative to the starting material **23**.

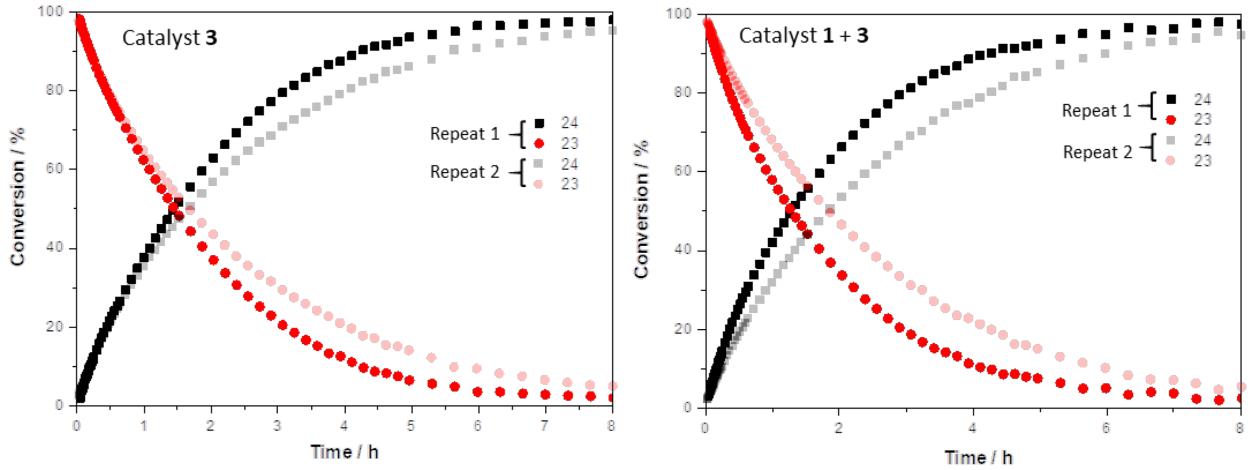

Figure S59: Reaction profiles for the intramolecular hydroamination catalysed by either Ir(III) **3** (left) or a 1:1 mixture of BDP **1** + Ir(III) **3** (right), monitored by *in situ* NMR spectroscopy, using the reaction conditions outlined above.

To compare with the Ir(III) based catalysts, control experiments using Ir(I) **2**, Ir(I)-BDP SS **4** and the 1:1 mixture of BDP **1** and Ir(I) **2** were conducted once. The corresponding results are summarised in the following table.

Table S10: Summarised control reactions of intramolecular hydroamination using different catalysts, or no catalyst.

Catalyst	Conversion to the product **24** at 6 h / %
BDP **1**	8
Ir(I) **2**	62
Ir(III) **3**	94±2
Ir(I)-BDP SS **4**	59
Ir(III)-BDP SS **7**	96±0
Ir(I) **2** + BDP **1***	98
Ir(III) **3** + BDP **1***	94±2
No catalyst	8

*BDP **1** (0.002 mmol) and Ir(I) **2** (0.002 mmol) / Ir(III) **3** (0.002 mmol) was used.

These results suggest that Ir(I) based catalysts are inferior in promoting the intramolecular hydroamination reaction.

Sequential reaction

Optimisation of the first step – intramolecular hydroamination

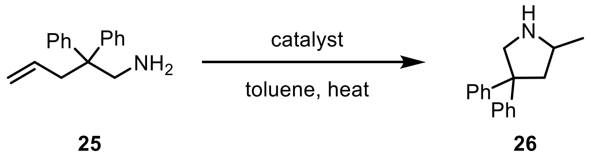

Procedure: aminoalkene **25** (23.7 mg, 0.1 mmol), catalyst (0.001 mmol) were dissolved in dry toluene (0.5 mL) in a 4 mL transparent vial in a glovebox under argon and sealed with a PTFE cap. The reaction was heated at different temperatures and for different reaction times. Aliquots were taken from the reaction mixture for ^{1}H NMR analyses. Conversion to the product **26** was calculated relative to starting material **25**.

Table S11: Optimisation of the reaction conditions, and the control reaction using no catalyst. The optimal reaction conditions are indicated in bold.

Catalyst / equiv.	Temperature / °C	Time / h	Conversion to the product **26** / %
Ir(I)-BDP SS **4** / 5 mol%	110	18	100
Ir(I)-BDP SS **4** / 2 mol%	110	18	100
Ir(I)-BDP SS **4** /1 mol%	100	18	100
Ir(I)-BDP SS **4** /1 mol%	100	3	53
Ir(I)-BDP SS **4** /1 mol%	**130**	**2**	**100**
No catalyst	130	18	0

The NMR signals used to monitor reaction progress

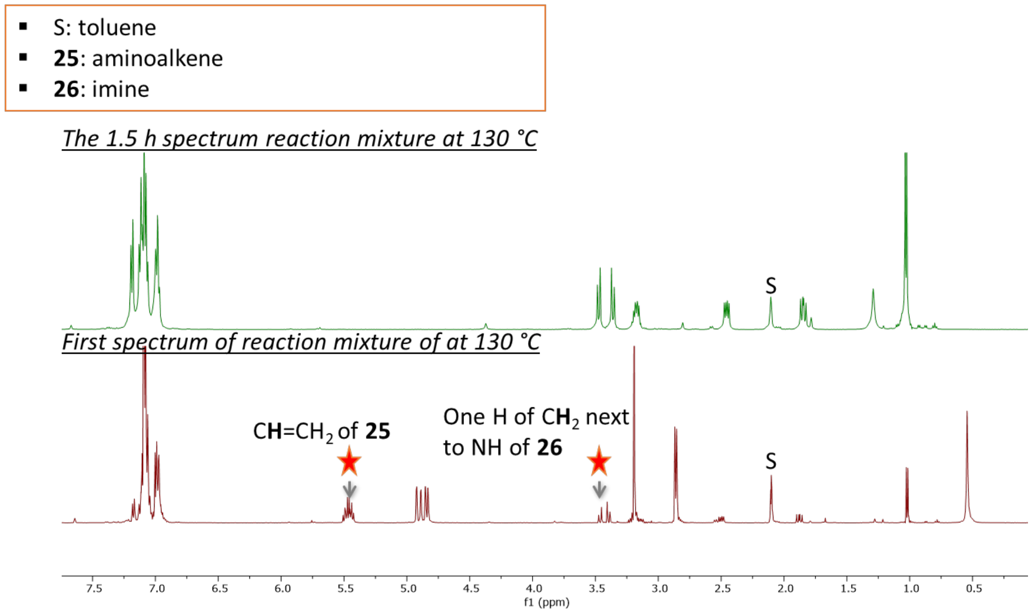

Figure S60: ^{1}H NMR spectra of the intramolecular hydroamination of compound **25** promoted by Ir(I)-BDP SS **4**, monitored by *in situ* NMR spectroscopy at 130 °C in toluene-d_8, using the same scale as outlined above. Conversion to the product **26** was determined relative to the starting material **25**, with the specific signals used indicated by the red stars.

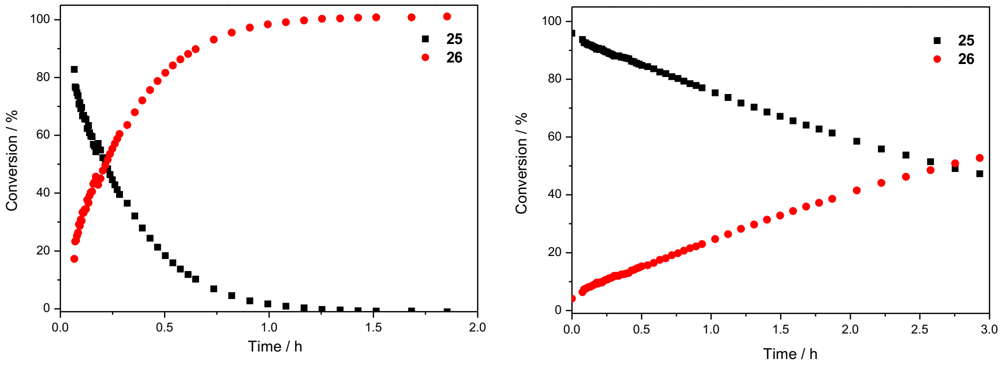

Figure S61: Reaction profile for the hydroamination of aminoalkene **25** catalysed by Ir(I)-BDP SS **4** monitored by *in situ* NMR spectroscopy at 130 °C (left) and 100 °C (right), using the reaction conditions outlined above.

Optimised procedure: aminoalkene **25** (94.8 mg, 0.4 mmol), catalyst Ir(I)-BDP SS **4** (6.3 mg, 0.004 mmol) were dissolved in dry toluene (0.5 mL) in a 4 mL transparent vial in a glovebox under argon and sealed with a PTFE cap, and the reaction mixture was heated at 130 °C for 2 hours. The internal standard trimethoxybenzene (67.2 mg, 0.4 mmol) was then added, and the crude reaction mixture was analysed by ^{1}H NMR spectroscopy, indicating that there was 92% conversion to the product **26**. The solvent was evaporated under reduced pressure and the crude product was purified by flash column chromatography on silica gel eluting with hexane-EtOAc (1:1, v/v, R_f = 0.2) to give the product **26** as a colourless oil in 86% yield (81.5 mg). ^{1}H NMR (400 MHz, Chloroform-*d*) δ 7.35 – 7.24 (m, 8H), 7.22 – 7.16 (m, 2H), 3.70 (dd, *J* =11.3, 1.4 Hz, 1H), 3.50 (d, *J* = 11.3 Hz, 1H), 3.40 (dq, *J* = 9.0, 6.4 Hz, 1H), 2.77 (ddd, *J* = 12.7, 6.6, 1.4 Hz, 1H), 2.07 (dd, *J* = 12.7, 9.0 Hz 1H), 1.89 (br s, 1H), 1.23 (d, *J* = 6.4 Hz, 3H) ppm. ^{13}C{^{1}H} NMR (101 MHz, Chloroform-*d*) δ 148.0, 147.3, 128.5, 128.4, 127.2, 127.1, 126.1, 126.1, 58.1, 57.5, 53.2, 47.3, 22.6 ppm. The data matches that reported in the literature.[14]

Optimisation of the second step – photooxidation of pyrrolidine 26

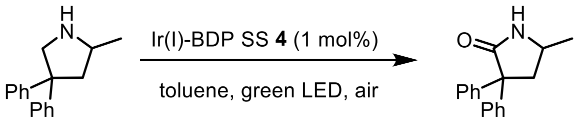

Procedure: pyrrolidine **26** (94.8 mg, 0.4 mmol), Ir(I)-BDP SS **4** (0.004 mmol) was dissolved in anhydrous toluene (2 mL) and the reaction was irradiated with green LED light under air. Aliquots were taken at different time points, dissolved in chloroform-d_1 and analysed using ^{1}H NMR spectroscopy. Conversion to the product **27** was calculated relative to starting material **26**, and the data is shown in Table S12.

Table S12: Reaction profile for photooxidation of **26** catalysed by Ir(I)-BDP SS **4** under green LED irradiation, monitored by NMR spectroscopy.

Time / h	Conversion to **27**
2	17
6	47
10	80
18	99
24	99

The NMR signals used to monitor reaction progress

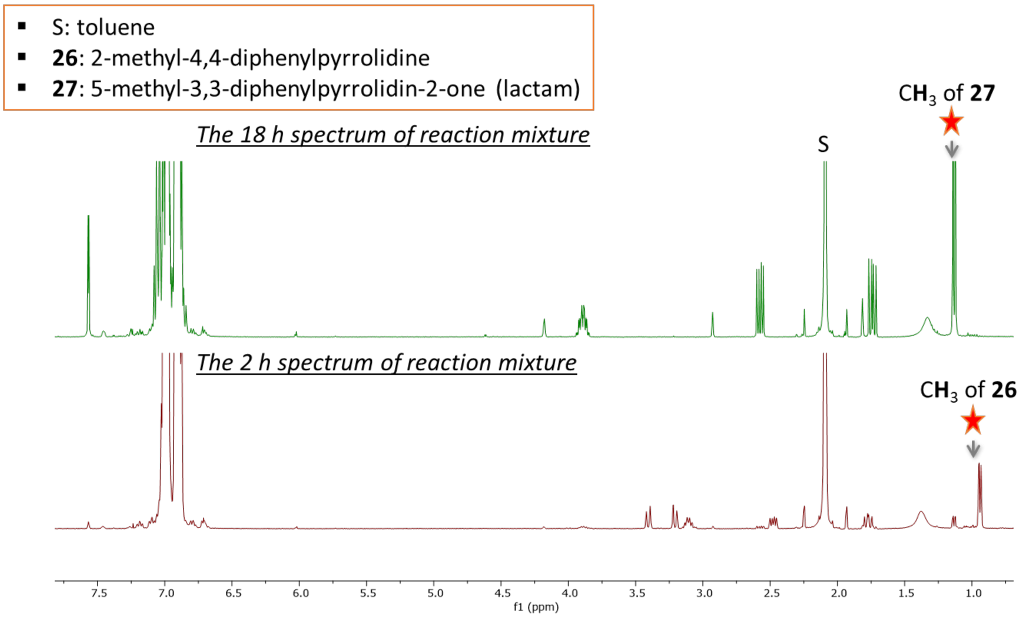

Figure S62: ^{1}H NMR spectra of the photooxidation of **26** promoted by Ir(I)-BDP SS **4** at different time points. Conversion to the product **27** was determined relative to the starting material **26**, with the specific signals used indicated by the red stars.

Table S13: Control reactions after 18 hours.

Catalyst	Conversion to the product **27** / %
BDP **1**	10
Ir(I) **2** + BDP **1**	24
No catalyst	0

One-pot sequential reaction

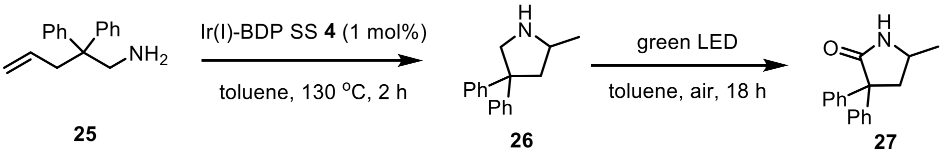

Optimised procedure: aminoalkene **25** (94.8 mg, 0.4 mmol), catalyst **4** (6.3 mg, 0.004 mmol) was dissolved in anhydrous toluene (2 mL) in a 4 mL vial sealed with polytetrafluoroethylene (PTFE) cap under argon. The reaction mixture was heated for 2 hours at 130 °C, then cooled to room temperature, opened to air and irradiated for 18 hours. The trimethoxybenzene internal standard (67.2 mg, 0.4 mmol) was added and the crude mixture was analysed by ^{1}H NMR spectroscopy, indicating that conversion to the product **27** was 70%. The solvent was removed under reduced pressure and the oily crude product was purified by flash column chromatography on silica gel eluting with hexane-EtOAc (3:1, v/v, R_f = 0.3) to give the product **27** as a colourless solid in 60% yield (60.3 mg). ^{1}H NMR (400 MHz, Chloroform-*d*) δ 7.78 (s, 1H), 7.30 – 7.20 (m, 4H), 7.20 – 7.13 (m, 2H), 7.13 – 7.05 (m, 4H), 4.10 (m, 1H), 2.78 (dd, *J* = 13.1, 6.5 Hz, 1H), 2.05 (s, 1H), 1.94 (dd, *J* = 13.1, 8.4 Hz, 1H), 1.33 (d, *J* = 6.8 Hz, 3H) ppm. $^{13}C\{^{1}H\}$ NMR (126 MHz, Chloroform-*d*) δ 168.8, 145.8, 143.6, 128.7, 127.4, 127.2, 126.9, 126.7, 67.9, 67.0, 46.5, 21.8 ppm.

The same procedure was used for the catalyst mixture of BDP **1** (0.004 mmol) and Ir(I) **2**, resulting in 24% conversion to the product **27** and an isolated yield of 17% (17.1 mg).

Switchable reaction

*Photooxidation of aminoalkene **25***

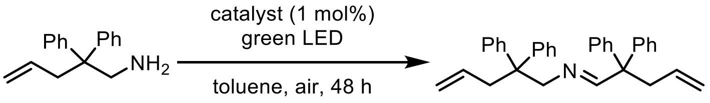

Procedure: aminoalkene **25** (23.7 mg, 0.1 mmol), catalyst (0.001 mmol) was dissolved in anhydrous toluene (0.5 mL) and the reaction was opened to air irradiated with green LED light for 18 hours. The reaction was monitored by ^{1}H NMR spectroscopy and the conversion to product **28** was calculated relative to starting material **25**, and the final conversion was calculated relative to both starting material **25** and internal standard trimethoxybenzene.

The NMR signals used to monitor reaction progress

- S: toluene
- IS: internal standard
- **25**: aminoalkene
- **28**: imine

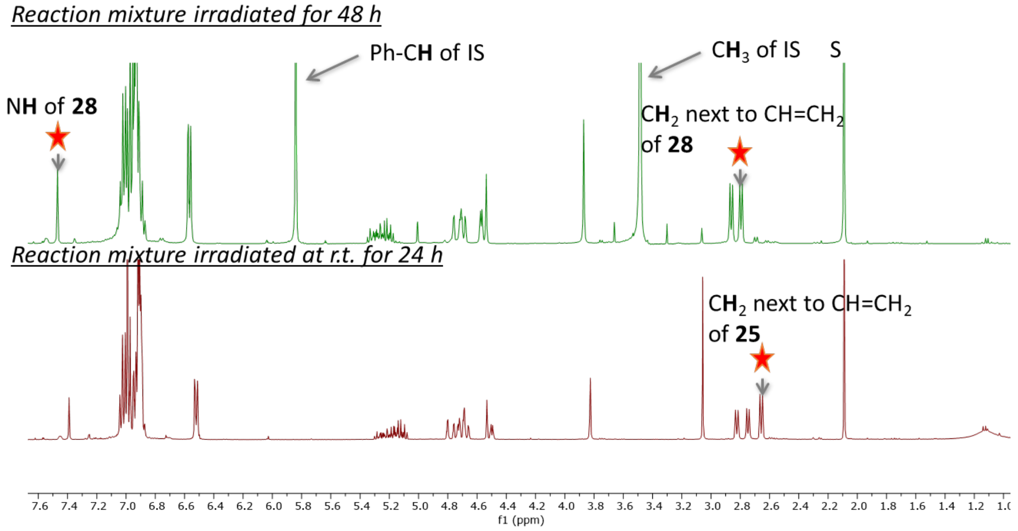

Figure S63: ^{1}H NMR spectra of the photooxidation of aminoalkene **25** promoted by Ir(I)-BDP SS **4** under green LED irradiation at different time points. Conversion to the product **28** was determined relative to internal standard or the starting material **25**, with the specific signals used indicted by the red stars.

Table S14: Optimisation of the reaction time for the photooxidation of aminoalkene **25**, with conversion to the product **28** determined relative to the starting material **25**.

Catalyst	Conversion to product **28** / %	
	24 h	48 h
Ir(I)-BDP SS **4** (1 mol%)	43	90 (82)

*Conversion relative to the internal standard is shown in parenthesis.

Table S15: Control reactions, with the conversions to the product **28** after 18 hours determined relative to the starting material **25**.

Catalyst / equiv.	Conversion to the product **28** / %
BDP **1** / 1 mol%	13
No light	0
No catalyst	0
No catalyst, no light	0

Optimised procedure: aminoalkene **25** (94.8 mg, 0.4 mmol), Ir-BDP SS **4** (6.3 mg, 0.004 mmol) was dissolved in anhydrous toluene (2 mL) and the reaction was opened to air and irradiated with green LED light for 48 hours. Isolation of the product **28** through chromatography using silica gel or neutral Al_2O_3 were unsuccessful due to the instability of the imine. Characterisation of the imine product **28** using ^1H NMR and $^{13}C\{^1H\}$ NMR spectroscopy and HRMS was performed using the crude reaction mixture. ^1H NMR (400 MHz, Methylene Chloride-d_2) δ 7.69 (s, 1H), 7.38 – 7.06 (m, 26H, contains toluene), 6.78 (d, *J* = 8.2 Hz, 4H), 5.57 – 5.37 (m, 2H), 4.99 – 4.89 (m, 2H), 4.81 – 4.76 (m, 1H), 4.75 (s, 1H), 4.10 (d, *J* = 1.3 Hz, 2H), 3.08 (d, *J* = 7.1 Hz, 2H), 3.00 (d, *J* = 7.1 Hz, 2H) ppm. $^{13}C\{^1H\}$ NMR (101 MHz, Methylene Chloride-d_2) δ 168.1, 147.5, 144.5, 138.4 (toluene), 136.2, 135.3, 129.4 (toluene), 129.1 (toluene), 128.6 (toluene), 128.5, 128.3, 126.6, 126.2, 125.6, 118.1, 116.8, 68.0, 51.0, 41.9, 41.6, 21.5 (toluene). HRMS (ESI$^+$, MeOH) calculated for $[C_{34}H_{33}N+H]^+$: 455.26130 $[M+H]^+$; found: 456.26873.

References

[1] G. Alesso, V. Tabernero, T. Cuenca, *J. Organomet. Chem.* **2012**, *717*, 202-210.
[2] X. Li, A. R. Chianese, T. Vogel, R. H. Crabtree, *Org. Lett.* **2005**, *7*, 5437-5440.
[3] C. Zheng, Q. Zang, H. Nie, W. Huang, Z. Zhao, A. Qin, R. Hu, B. Z. Tang, *Mater. Chem. Front.* **2018**, *2*, 180-188.
[4] D. F. Shriver, M. A. Drezdzon, *The manipulation of air-sensitive compounds*, John Wiley & Sons, **1986**.
[5] B. Ravel, M. Newville, *J. Synchrotron Radiat.* **2005**, *12*, 537-541.
[6] Z. Yang, W. Qin, J. W. Lam, S. Chen, H. H. Sung, I. D. Williams, B. Z. Tang, *Chem. Sci.* **2013**, *4*, 3725-3730.
[7] S. L. Dabb, J. H. Ho, R. Hodgson, B. A. Messerle, J. Wagler, *Dalton Trans.* **2009**, 634-642.
[8] C. M. Wong, K. Q. Vuong, M. R. D. Gatus, C. Hua, M. Bhadbhade, B. A. Messerle, *Organometallics* **2012**, *31*, 7500-7510.
[9] C. Zhang, J. Zhao, S. Wu, Z. Wang, W. Wu, J. Ma, S. Guo, L. Huang, *J. Am. Chem. Soc* **2013**, *135*, 10566-10578.
[10] M. Wang, Y. Zhang, T. Wang, C. Wang, D. Xue, J. Xiao, *Org. Lett.* **2016**, *18*, 1976-1979.
[11] a) N. Loim, E. Kelbyscheva, *Russ. Chem. Chem. Bull.* **2004**, *53*, 2080-2085; b) S. Chatterjee, S. Ramakrishnan, *ACS Macro Lett.* **2012**, *1*, 593-598.
[12] T. Matsumoto, Y. Urano, Y. Takahashi, Y. Mori, T. Terai, T. Nagano, *J. Org. Chem.* **2011**, *76*, 3616-3625.
[13] G. Alesso, V. Tabernero, T. Cuenca, *J. Organomet. Chem.* **2012**, *717*, 202-210.
[14] M. R. Crimmin, M. Arrowsmith, A. G. Barrett, I. J. Casely, M. S. Hill, P. A. Procopiou, *J. Am. Chem. Soc* **2009**, *131*, 9670-9685.
[15] O. V. Dolomanov, L. J. Bourhis, R. J. Gildea, J. A. Howard, H. Puschmann, *J. Appl. Cryst.* **2009**, *42*, 339-341.
[16] L. J. Farrugia, *J. Appl. Cryst.* **2012**, *45*, 849-854.
[17] a) X.-F. Zhang, X. Yang, *J. Phys. Chem. B,* **2013**, *117*, 5533-5539; b) T. Yogo, Y. Urano, Y. Ishitsuka, F. Maniwa, T. Nagano, *J. Am. Chem. Soc.* **2005**, *127*, 12162-12163; c) W. Hu, Y. Lin, X.-F. Zhang, M. Feng, S. Zhao, J. Zhang, *Dyes Pigments* **2019**, *164*, 139-147; d) W. Li, L. Li, H. Xiao, R. Qi, Y. Huang, Z. Xie, X. Jing, H. Zhang, *RSC Adv.* **2013**, *3*, 13417-13421.
[18] M. C. DeRosa, R. J. Crutchley, *Coord. Chem. Rev.* **2002**, *233*, 351-371.
[19] S. Matar, L. F. Hatch, *Chemistry of petrochemical processes*, Elsevier, **2001**, p148.

NMR Spectra

Catalysts 4-9

^{1}H (400 MHz, Methylene Chloride-d_2)

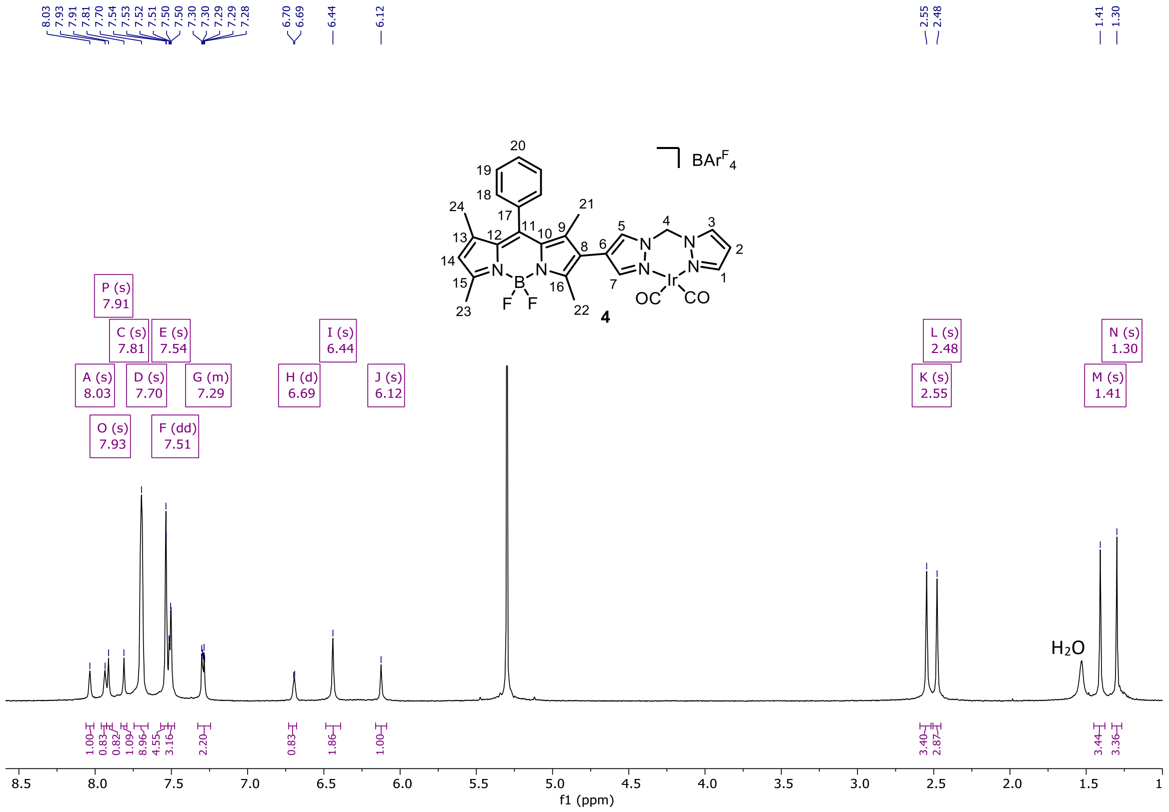

$^{13}C\{^{1}H\}$ (126 MHz, Methylene Chloride-d_2)

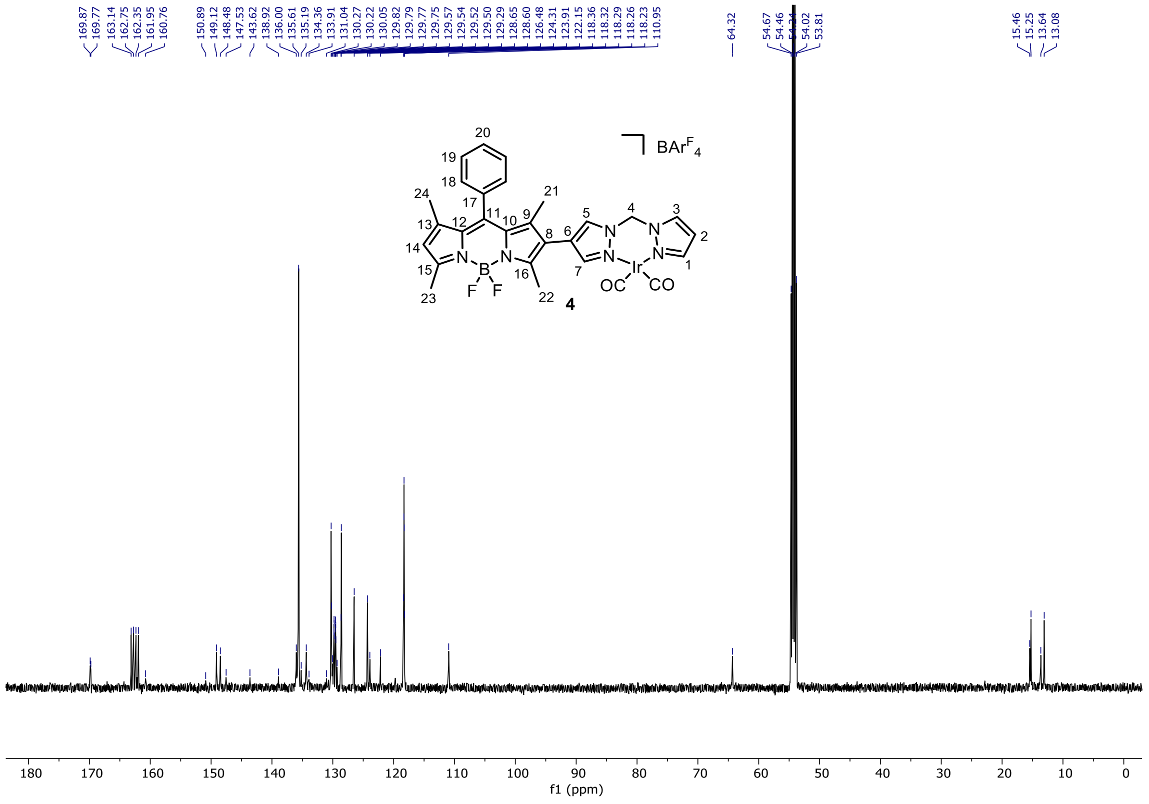

COSY - Ir(I)-BDP SS **4** in Methylene Chloride-d_2

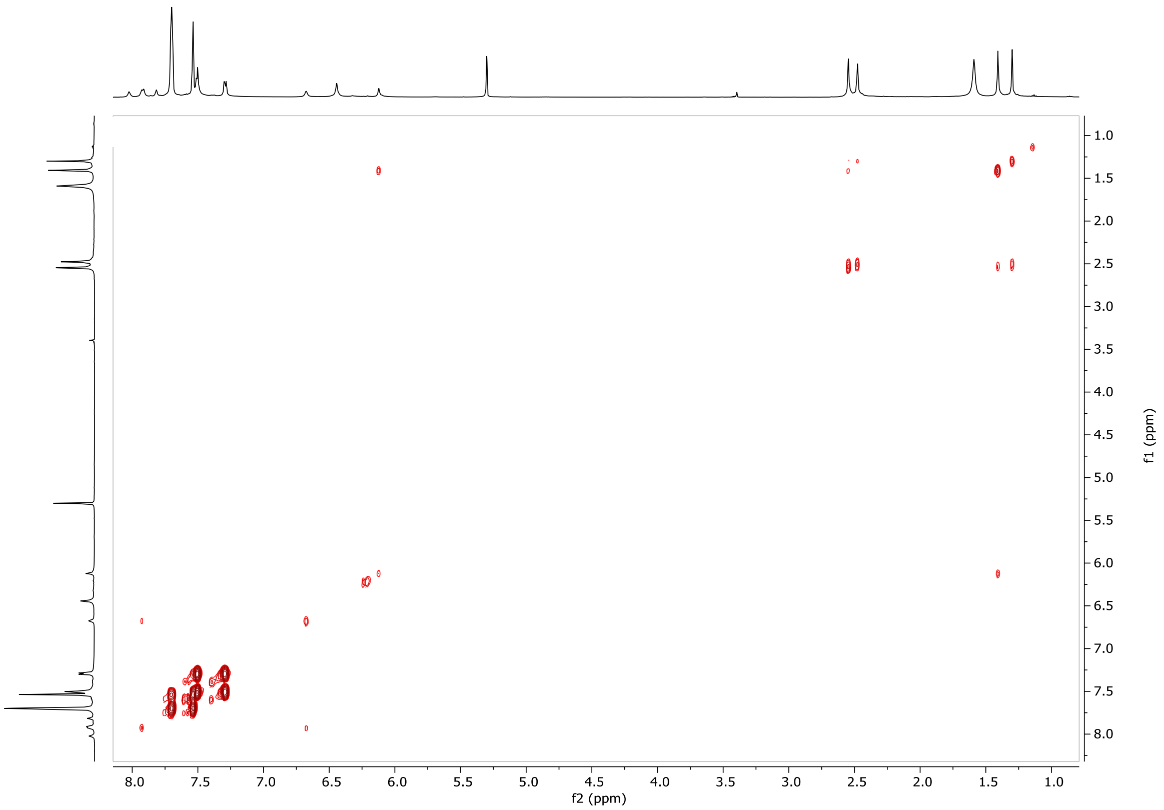

NOESY - Ir(I)-BDP SS **4** in Methylene Chloride-d_2

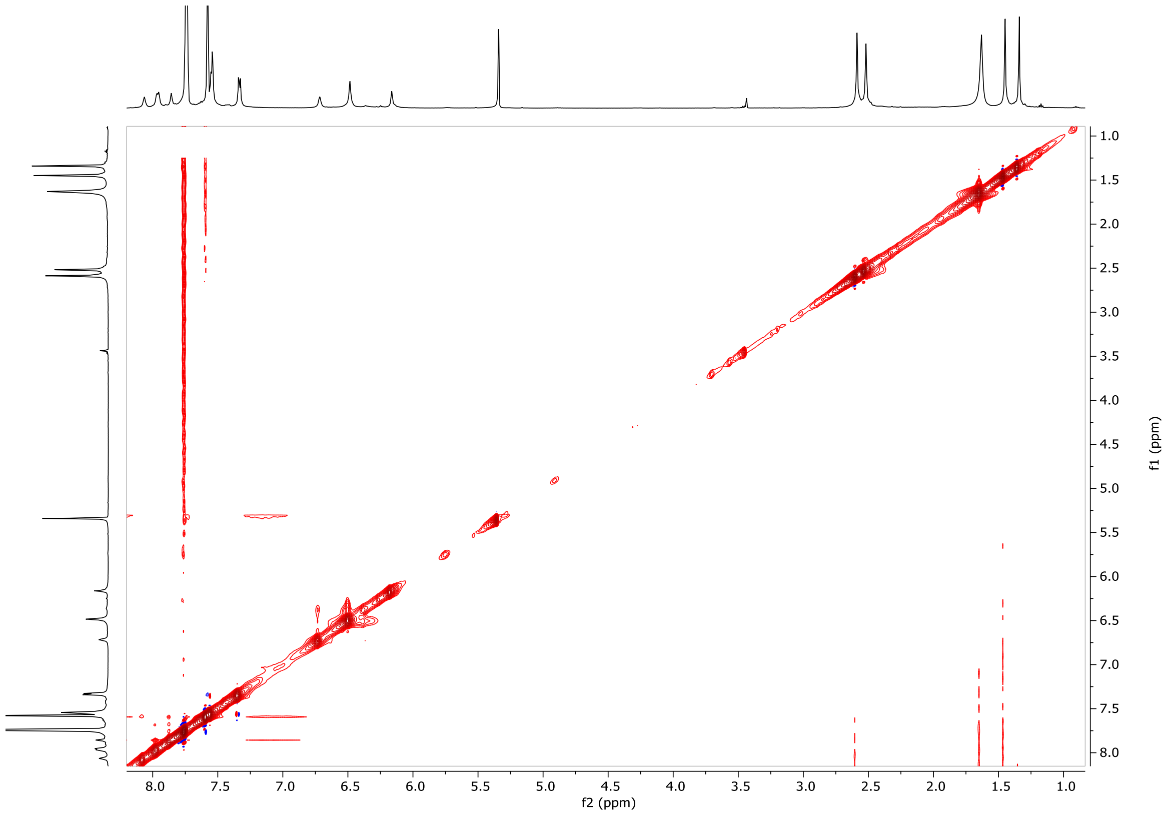

HSQC - Ir(I)-BDP SS **4** in Methylene Chloride-d_2

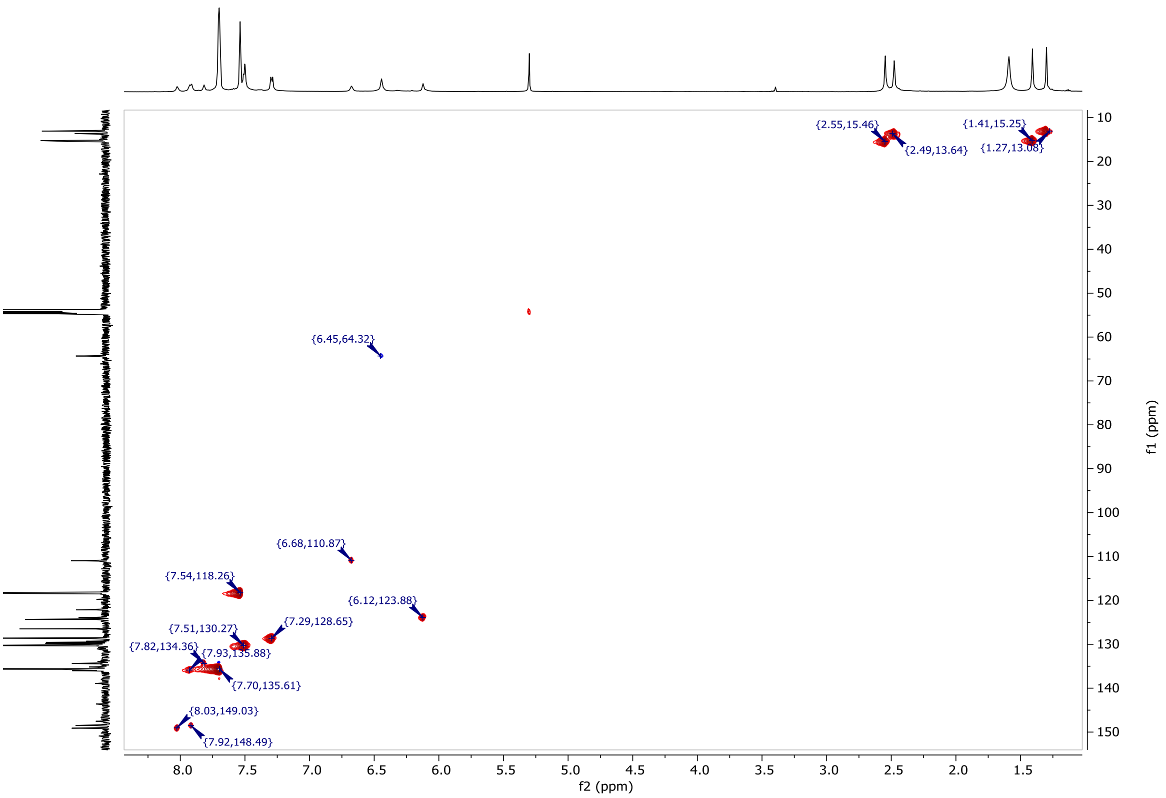

HMBC - Ir(I)-BDP SS **4** in Methylene Chloride-d_2

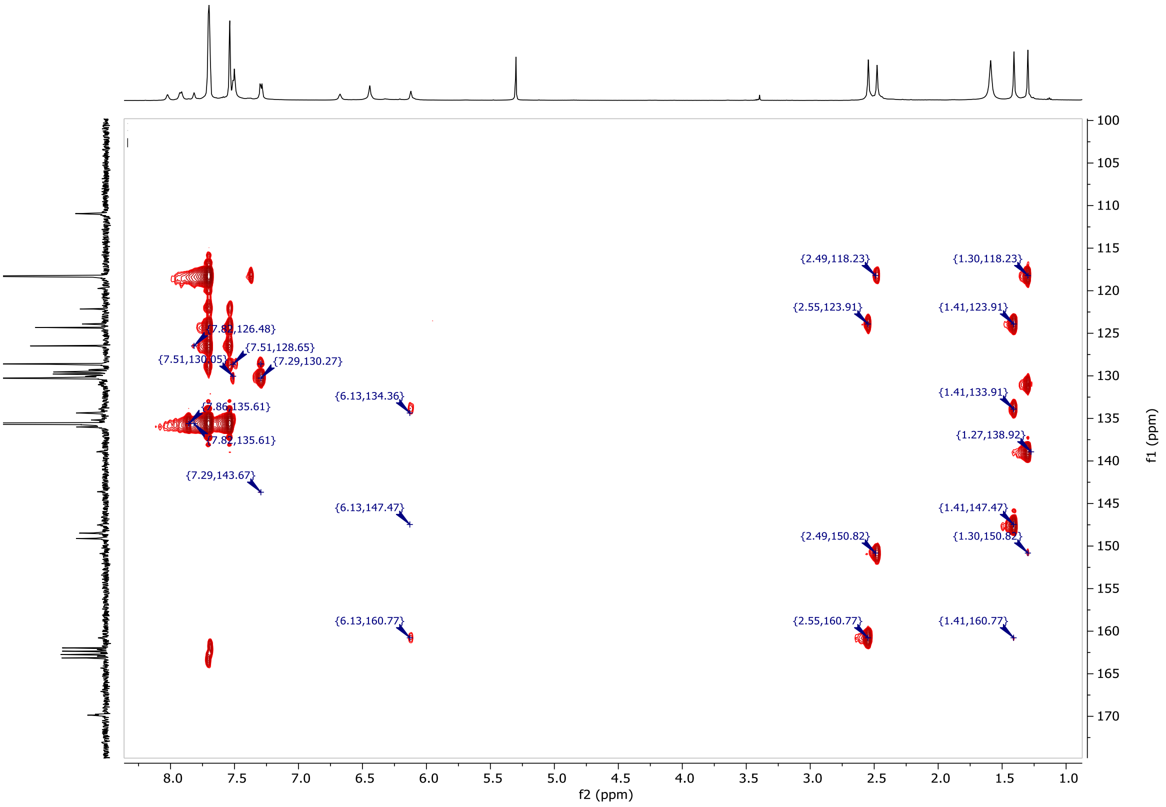

^1H (400 MHz, Methylene Chloride-d_2)

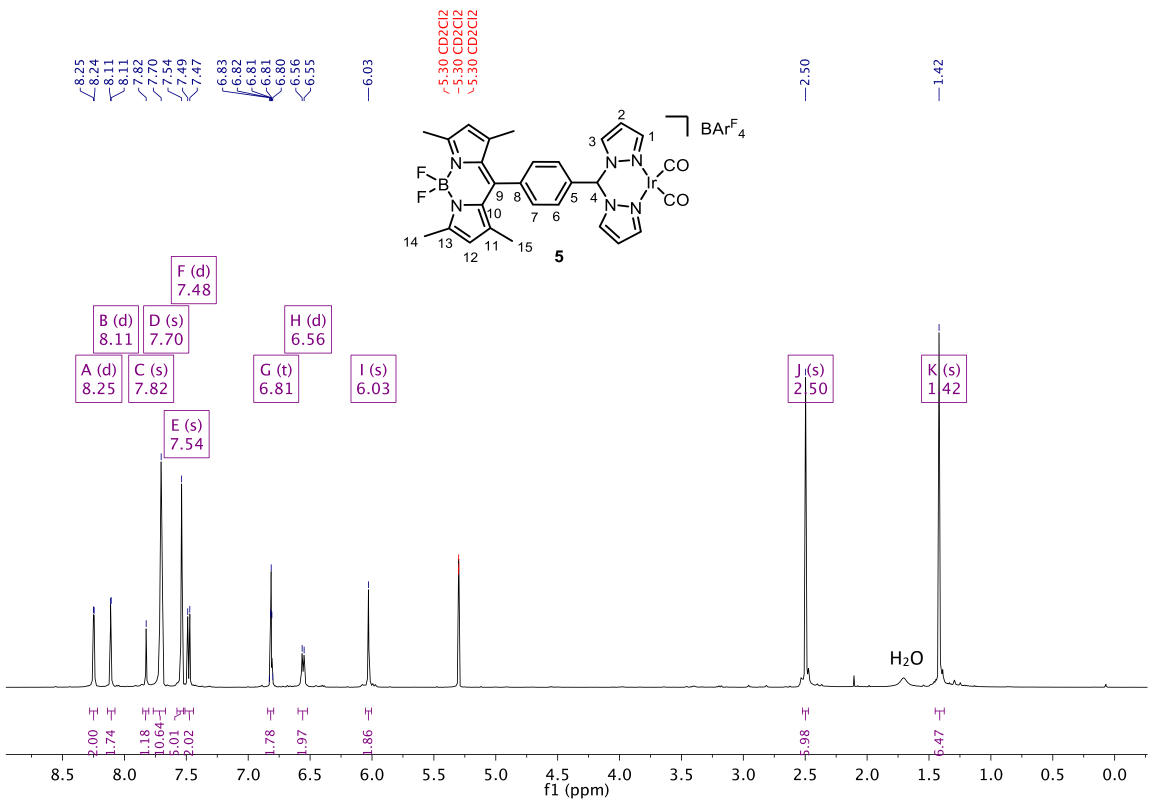

$^{13}C\{^1H\}$ (126 MHz, Methylene Chloride-d_2)

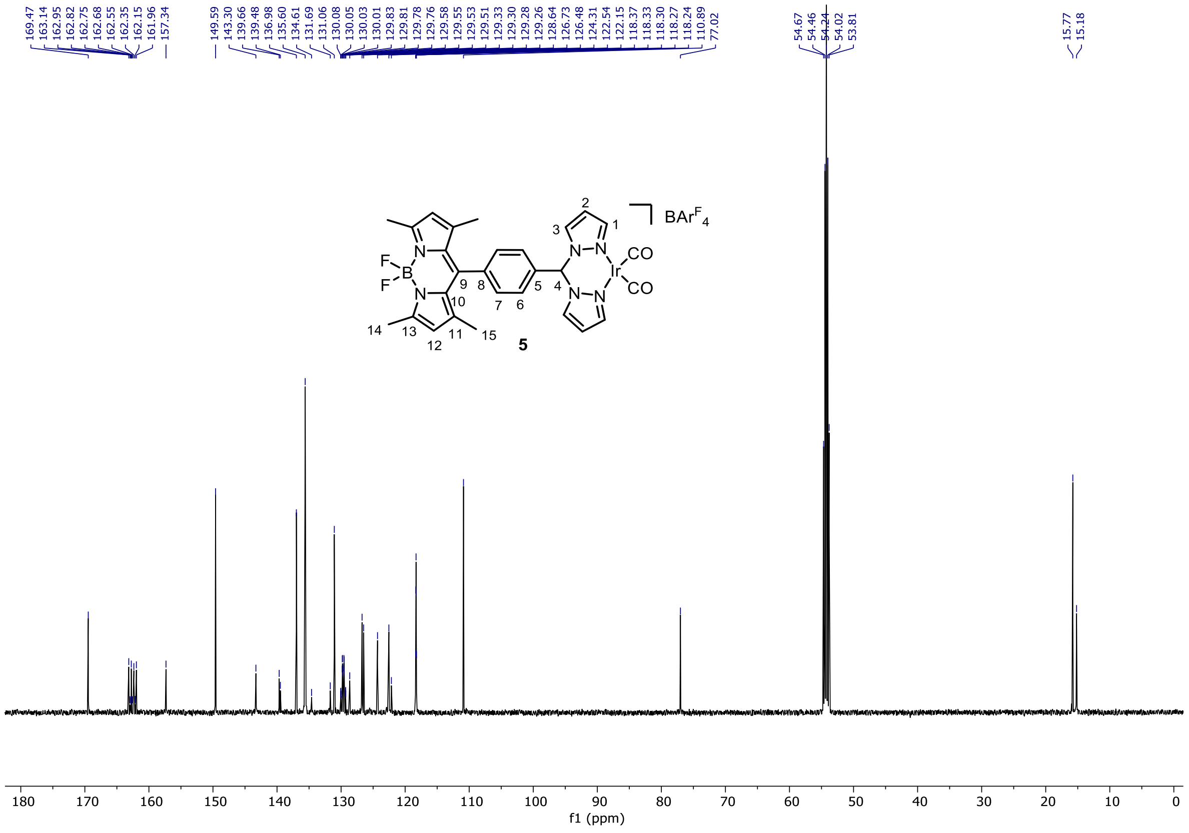

COSY - Ir(I)-BDP HH **5** in Methylene Chloride-d_2

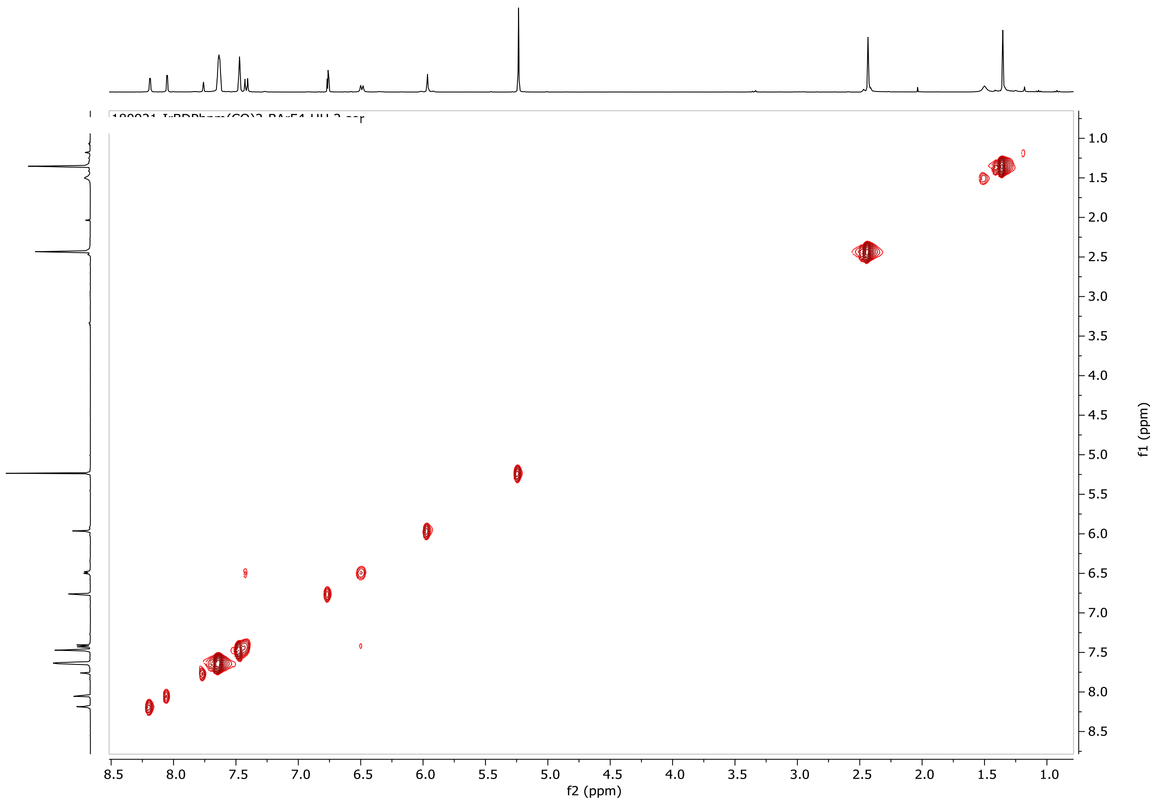

NOESY - Ir(I)-BDP HH **5** in Methylene Chloride-d_2

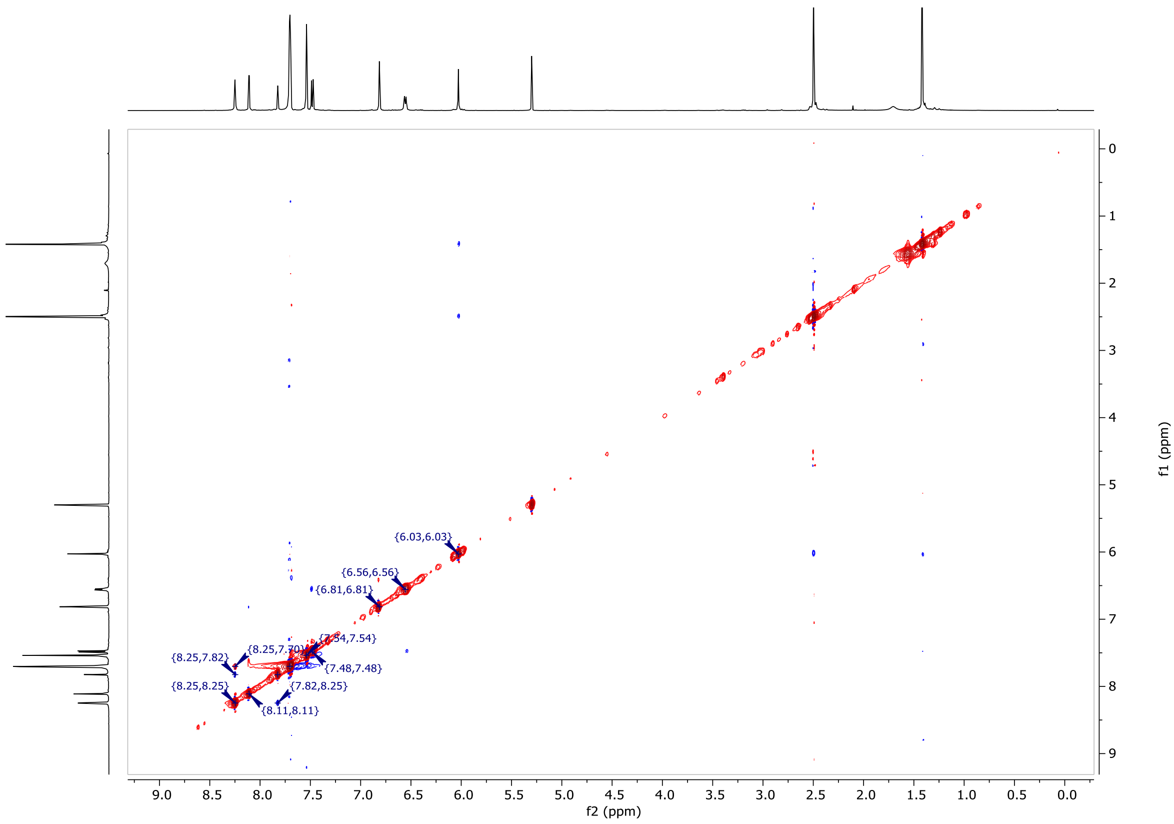

HMBC - Ir(I)-BDP HH **5** in Methylene Chloride-d_2

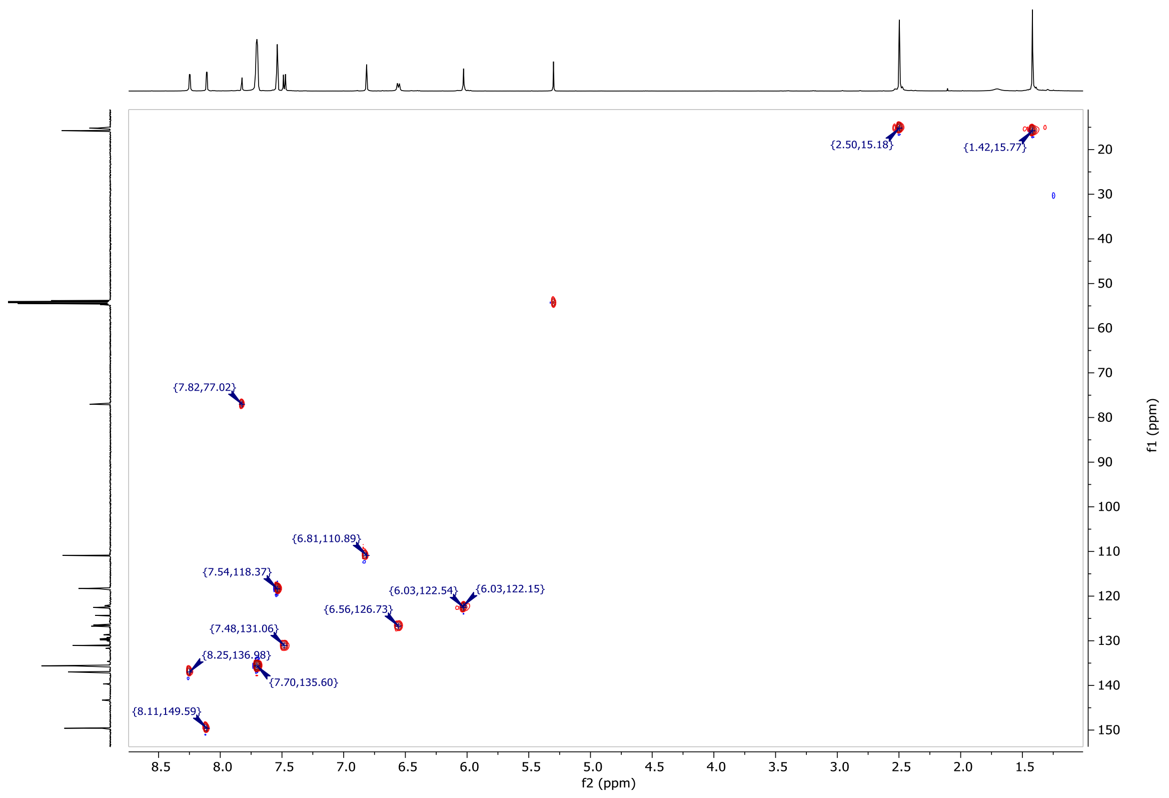

HSQC - Ir(I)-BDP HH **5** in Methylene Chloride-d_2

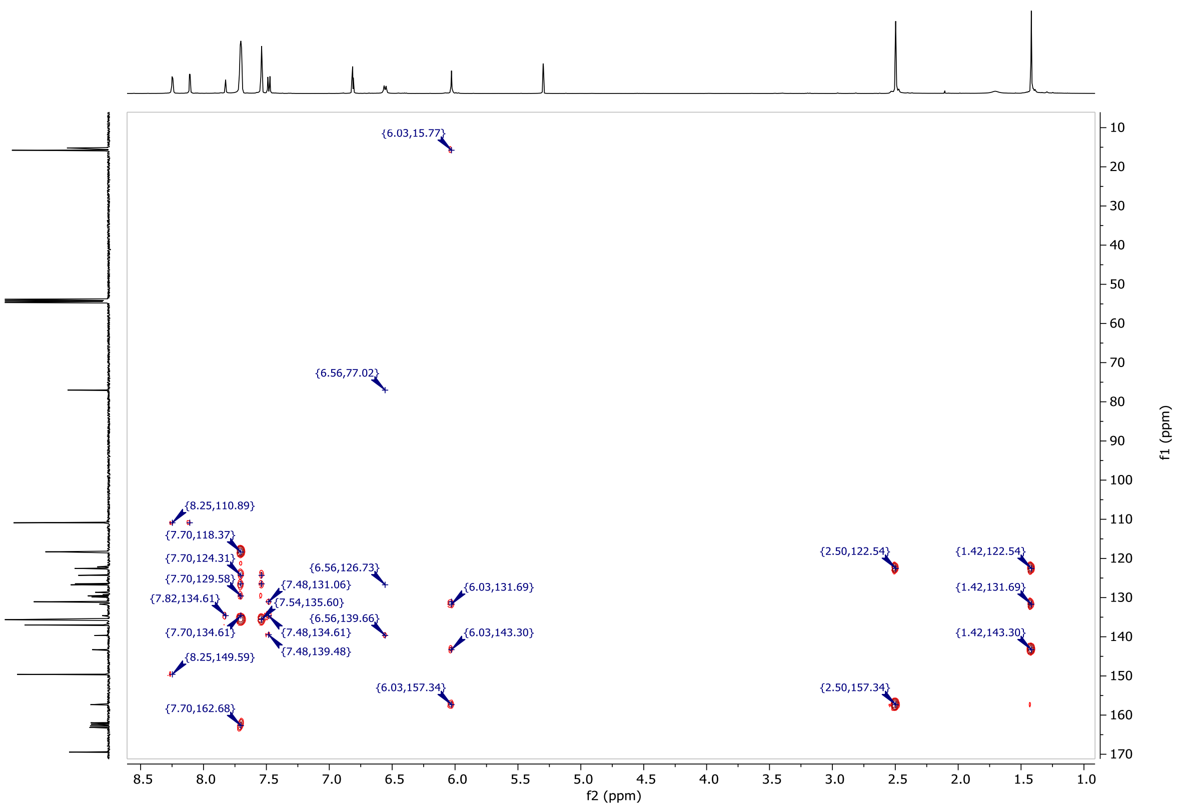

^{1}H (400 MHz, Methylene Chloride-d_2)

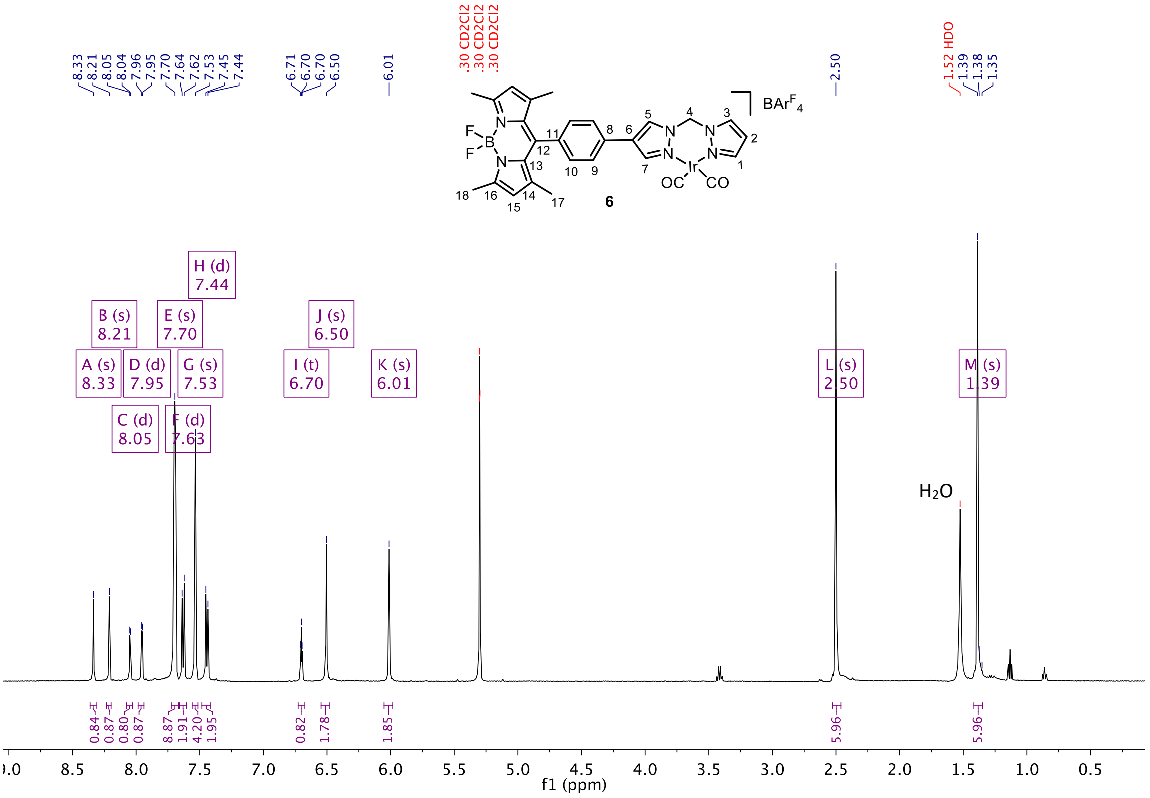

$^{13}C\{^{1}H\}$ (126 MHz, Methylene Chloride-d_2)

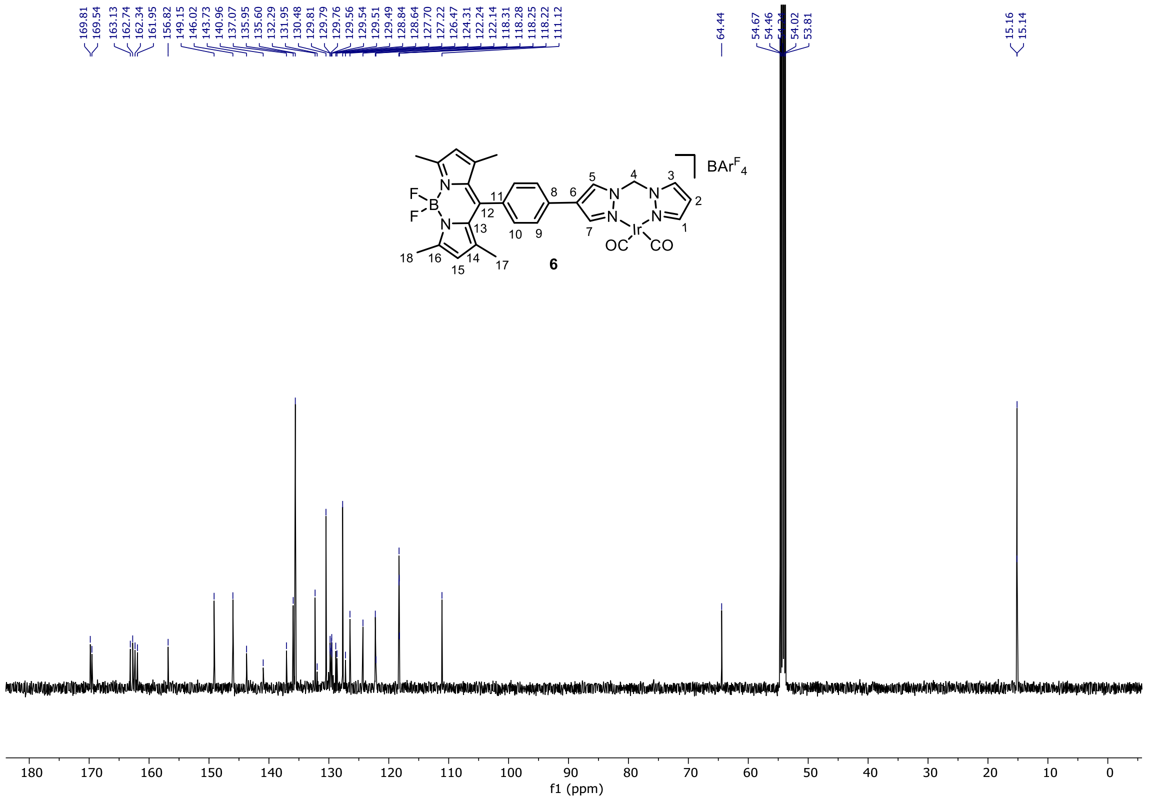

COSY - Ir(I)-BDP HS **6** in Methylene Chloride-d_2

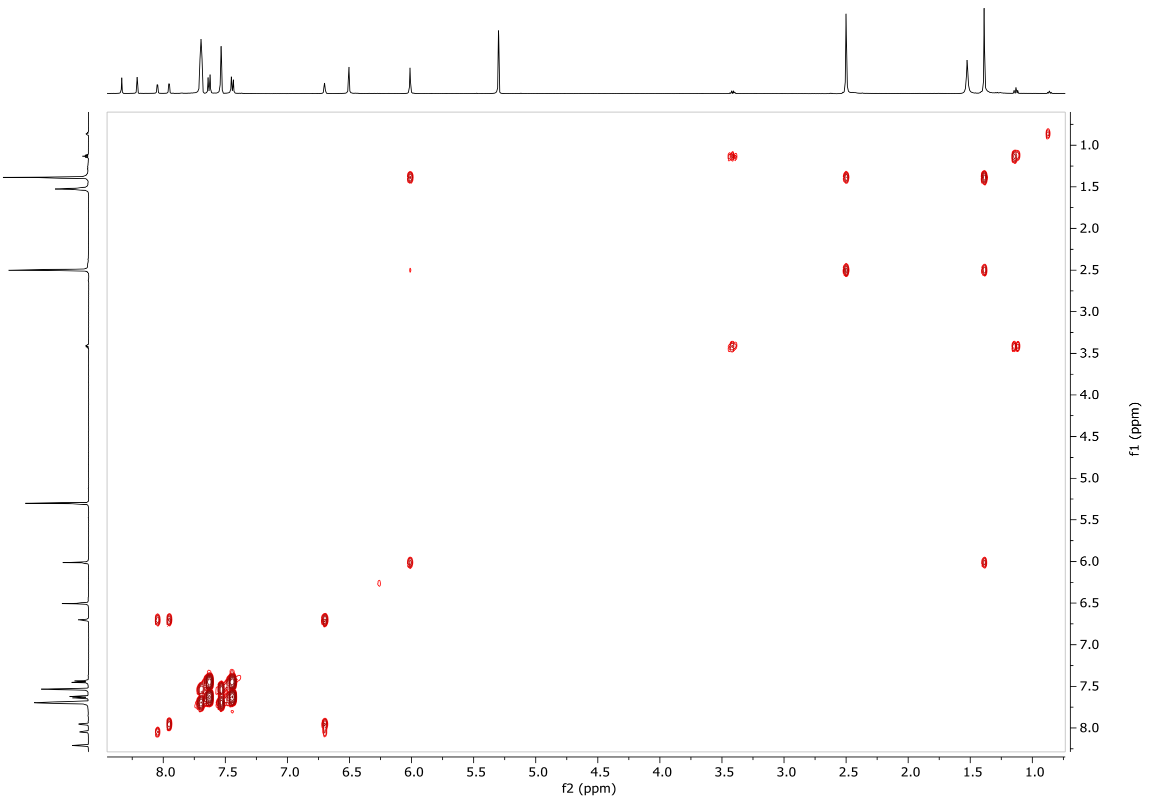

HMBC - Ir(I)-BDP HS **6** in Methylene Chloride-d_2

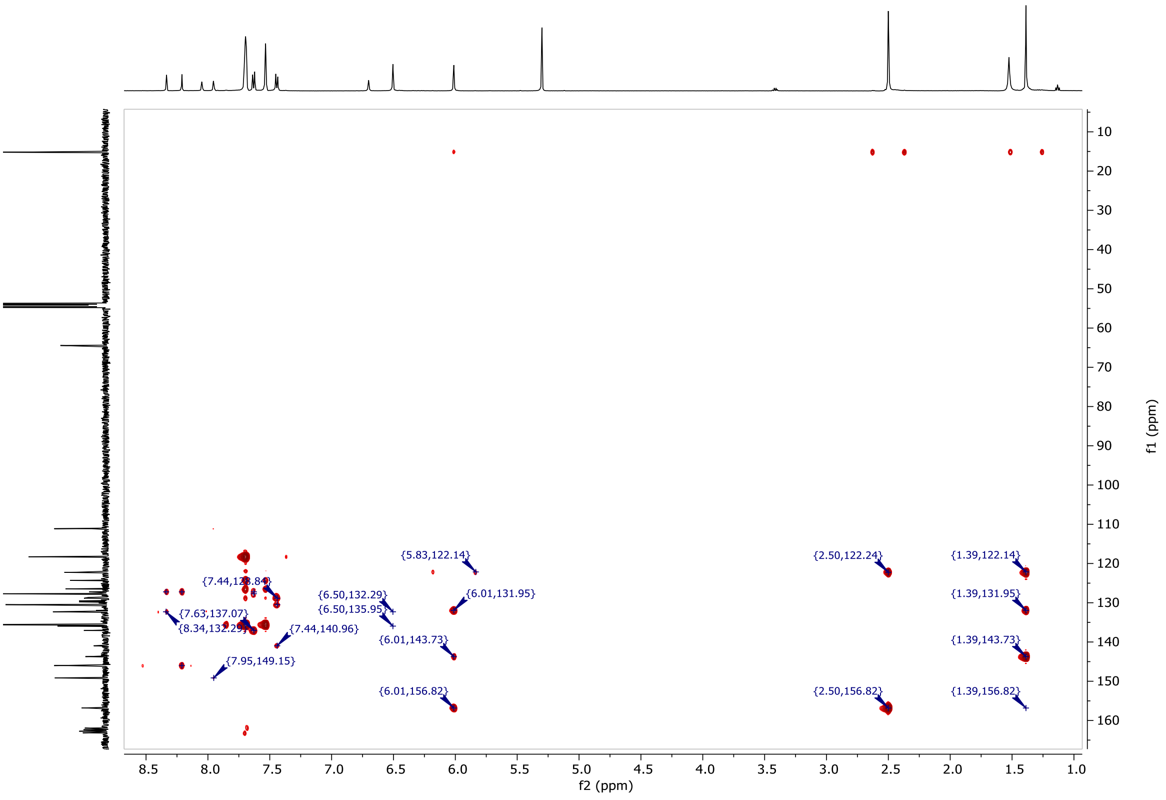

HSQC - Ir(I)-BDP HS **6** in Methylene Chloride-d_2

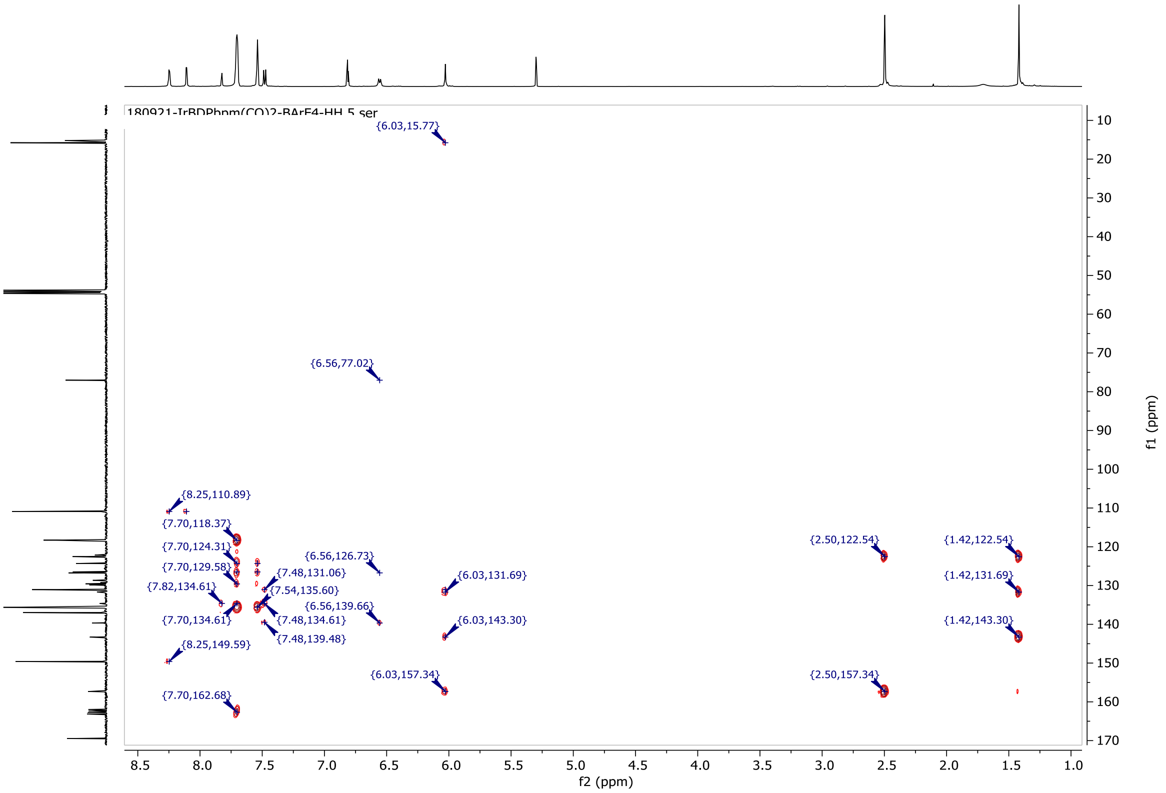

^{1}H (400 MHz, Methylene Chloride-d_2)

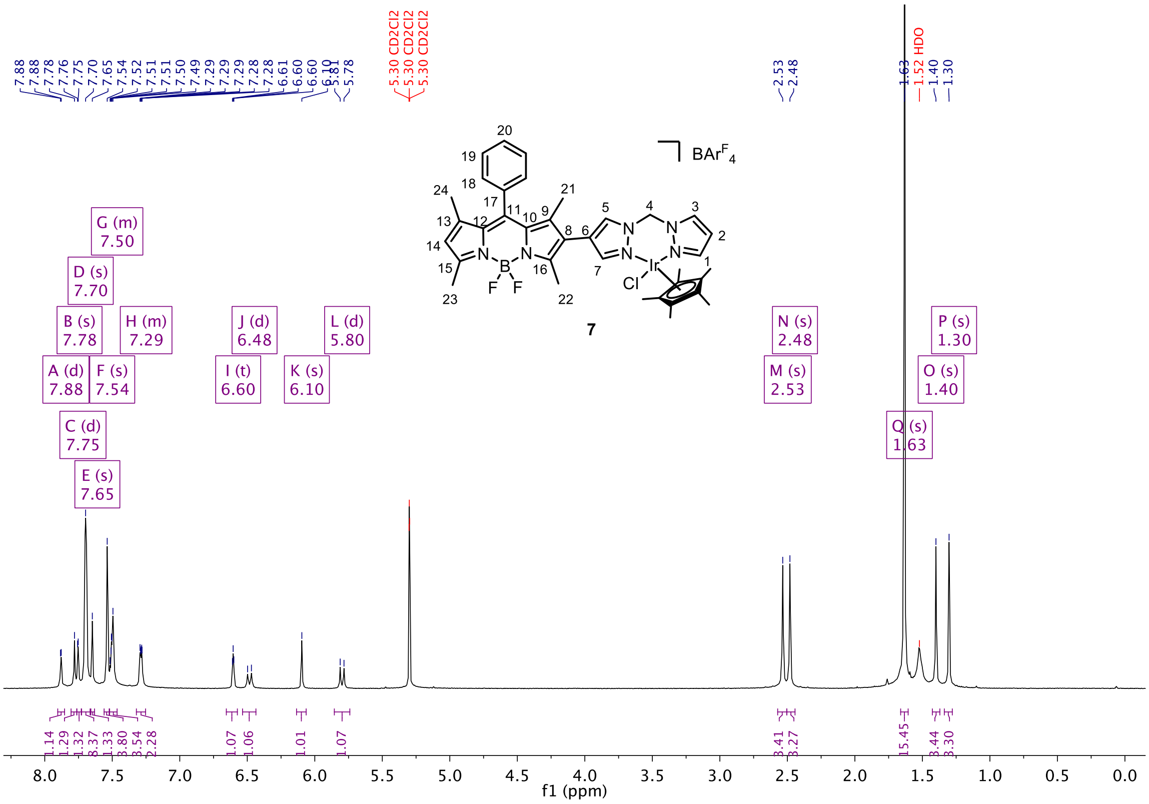

$^{13}C\{^{1}H\}$ (126 MHz, Methylene Chloride-d_2)

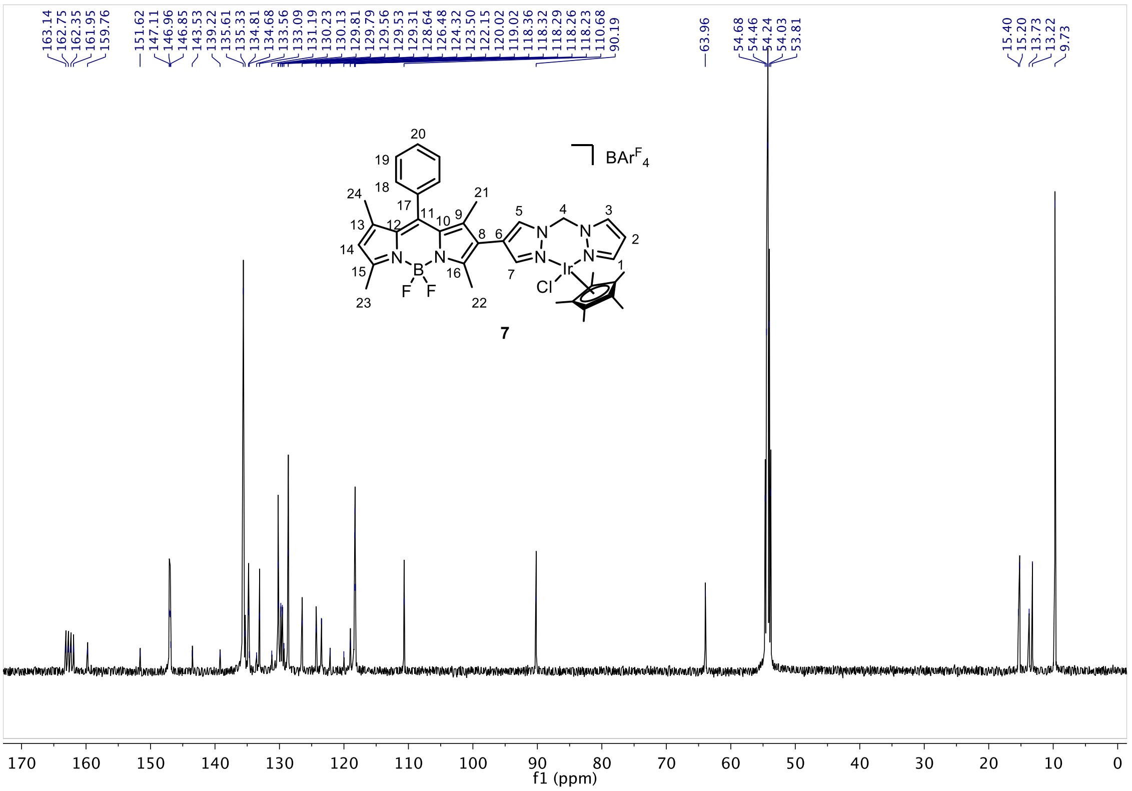

COSY - Ir(III)-BDP SS **7** in Methylene Chloride-d_2

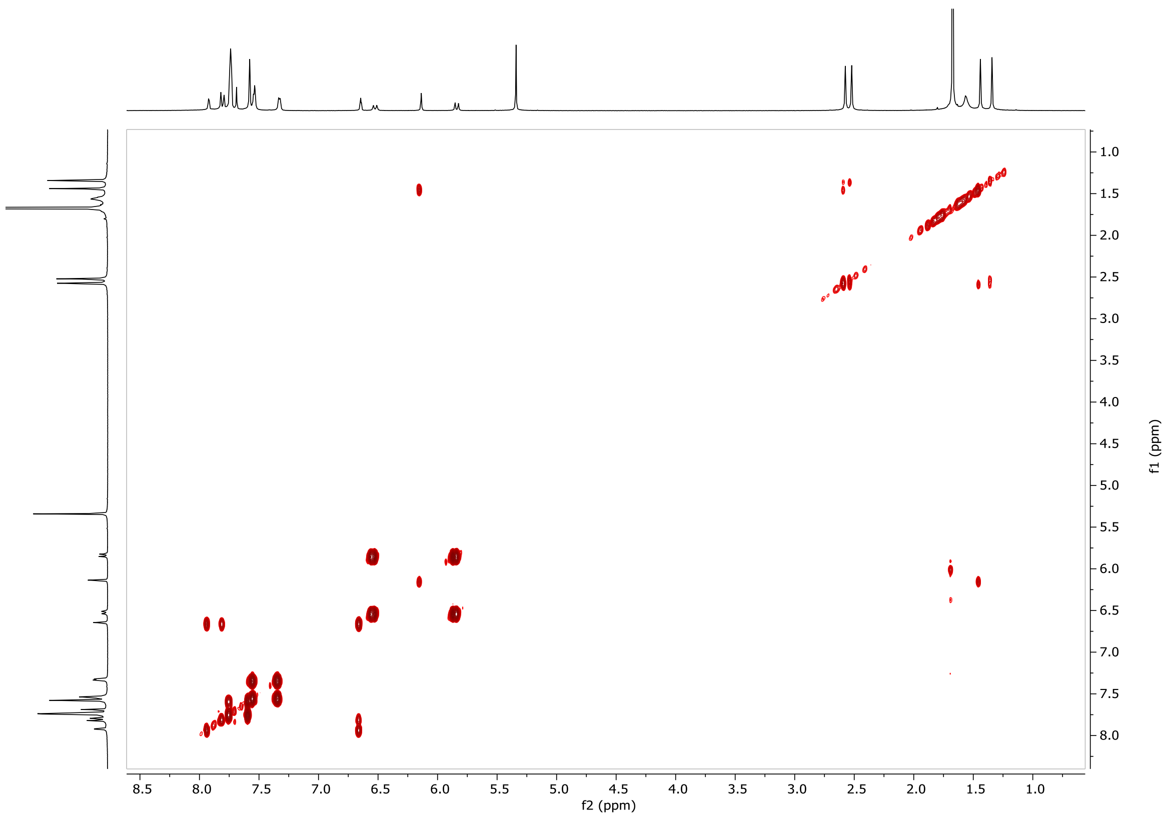

NOESY - Ir(III)-BDP SS **7** in Methylene Chloride-d_2

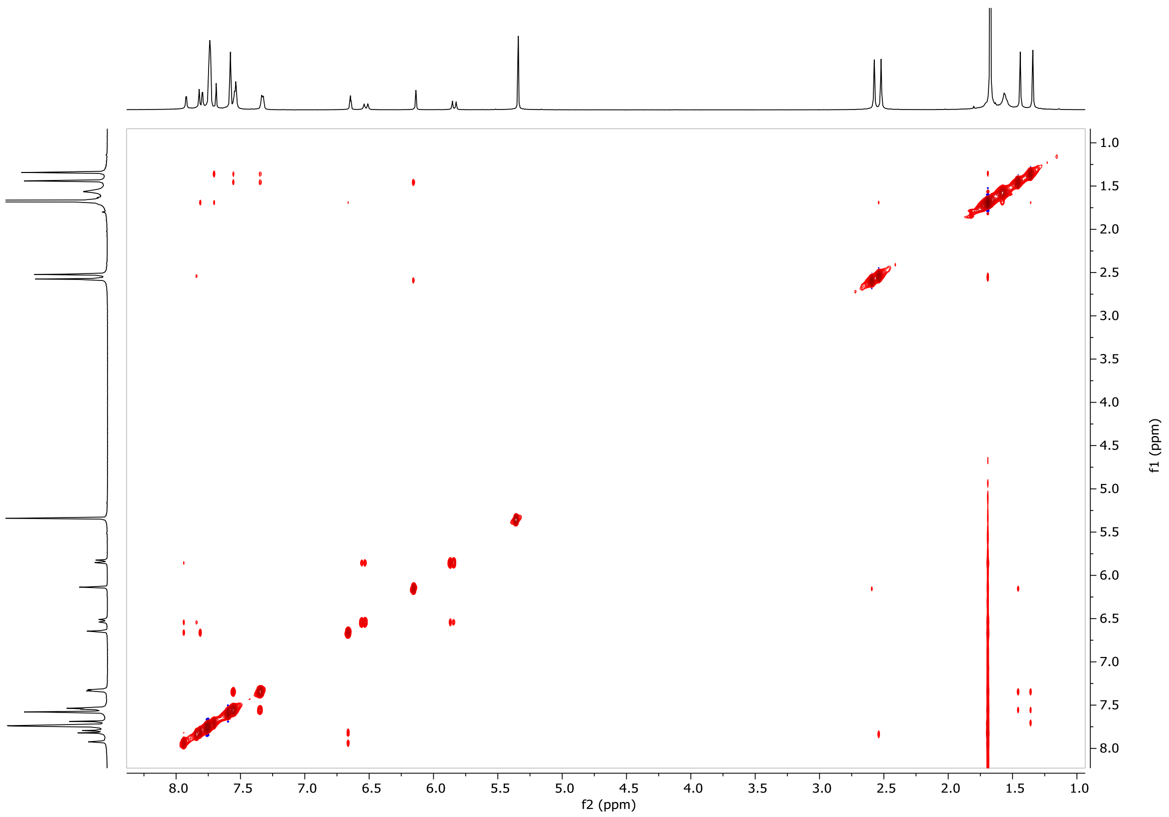

HSQC - Ir(III)-BDP SS **7** in Methylene Chloride-d_2

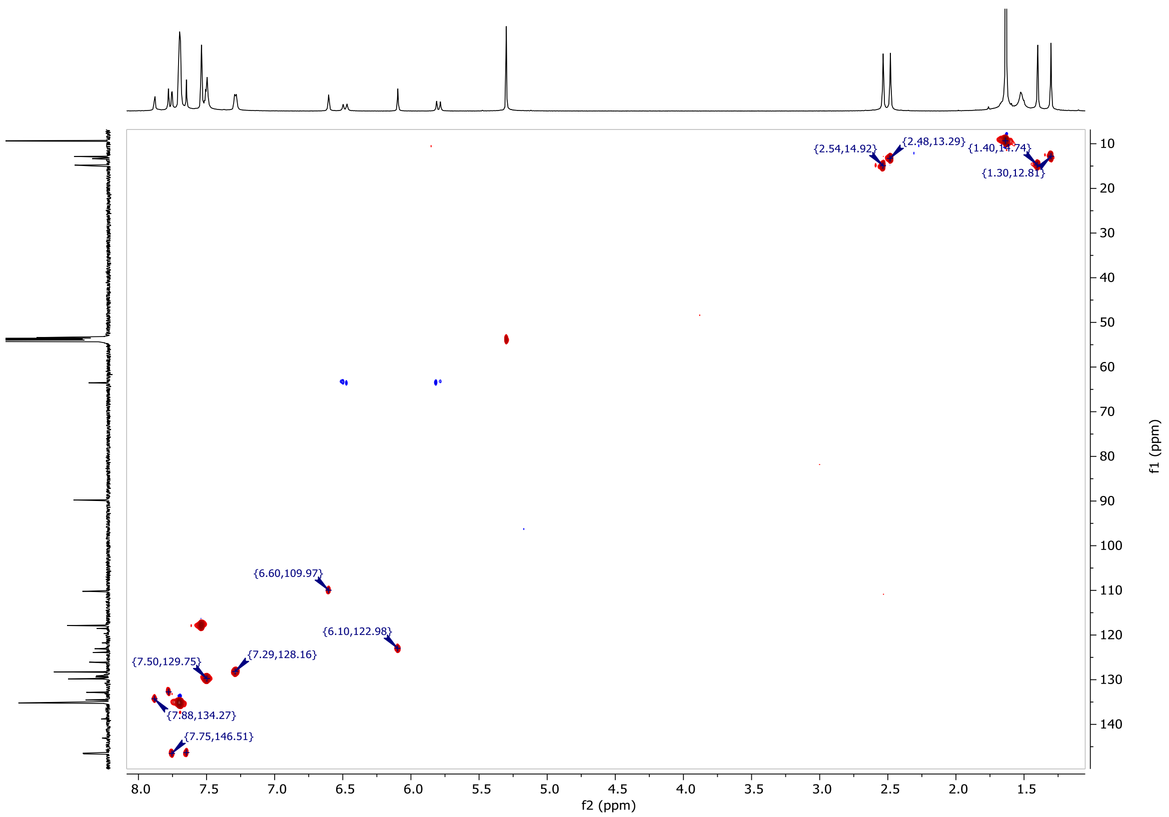

HMBC - Ir(III)-BDP SS **7** in Methylene Chloride-d_2

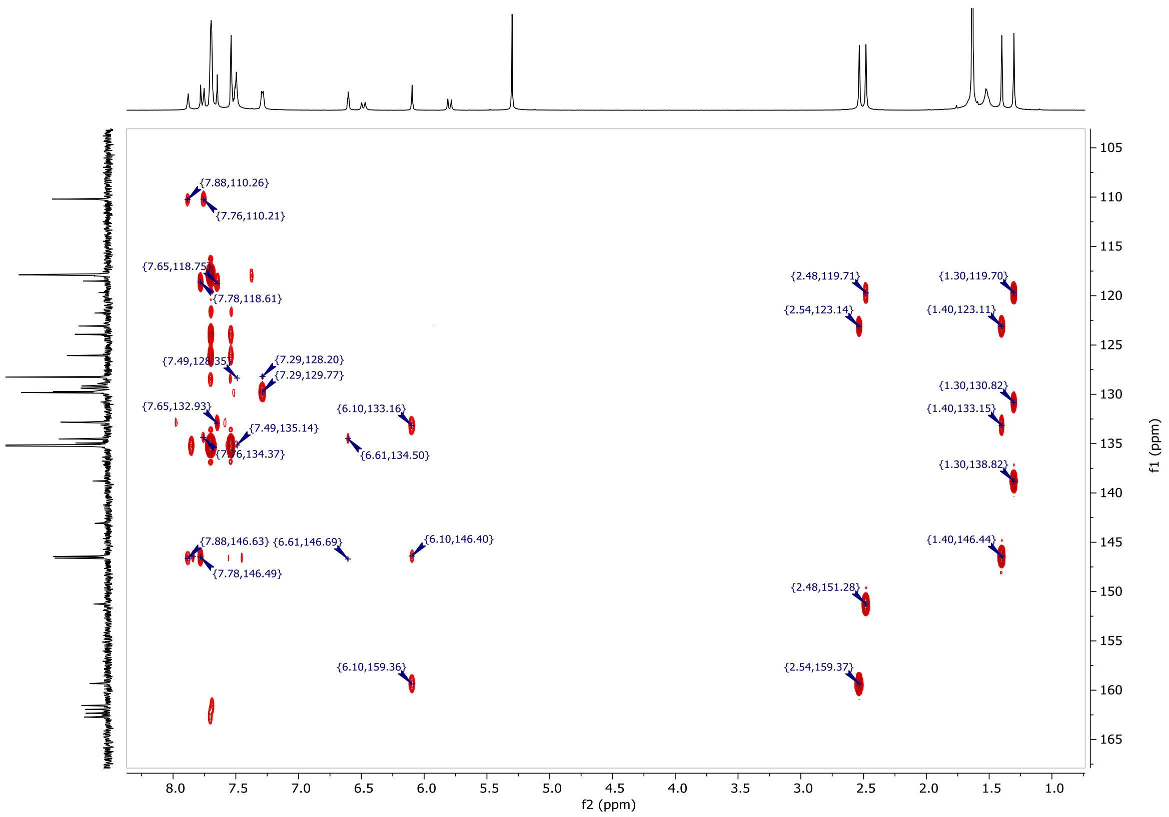

^{1}H (400 MHz, Methylene Chloride-d_2)

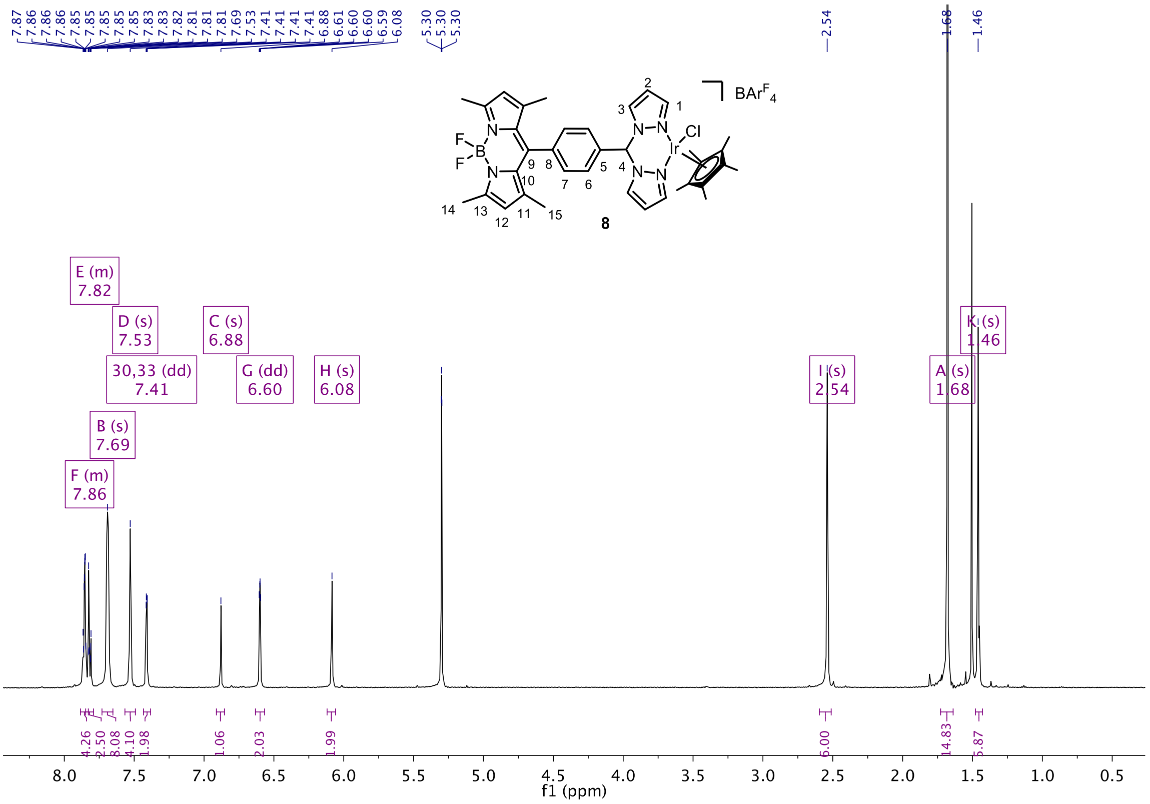

^{13}C{^{1}H} (126 MHz, Methylene Chloride-d_2)

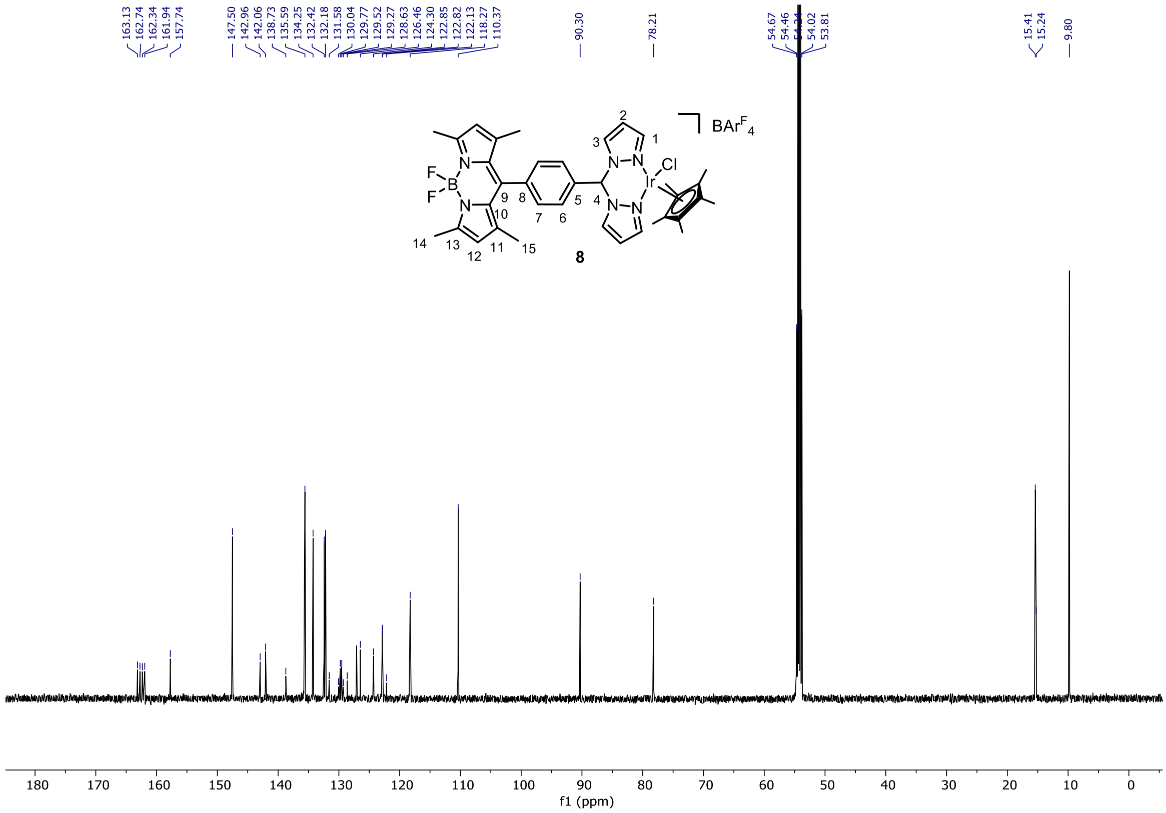

COSY - Ir(III)-BDP HH **8** in Methylene Chloride-*d*$_2$

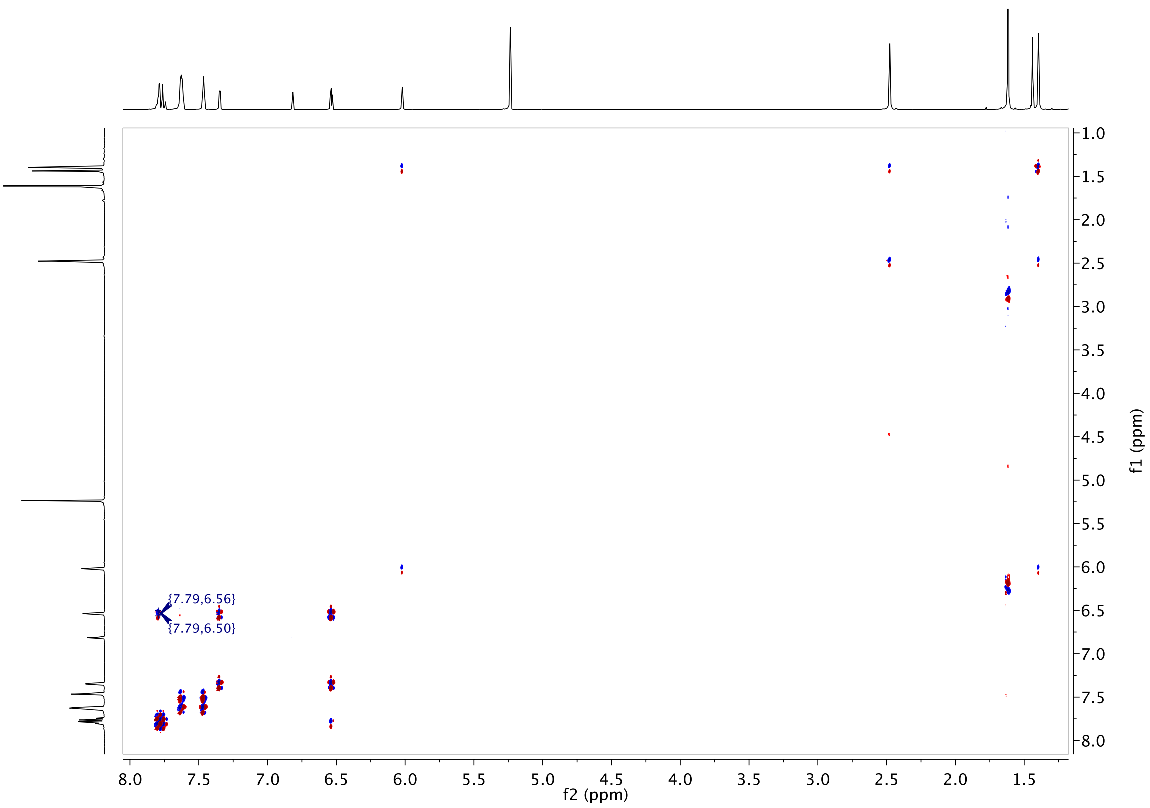

NOESY - Ir(III)-BDP HH **8** in Methylene Chloride-*d*$_2$

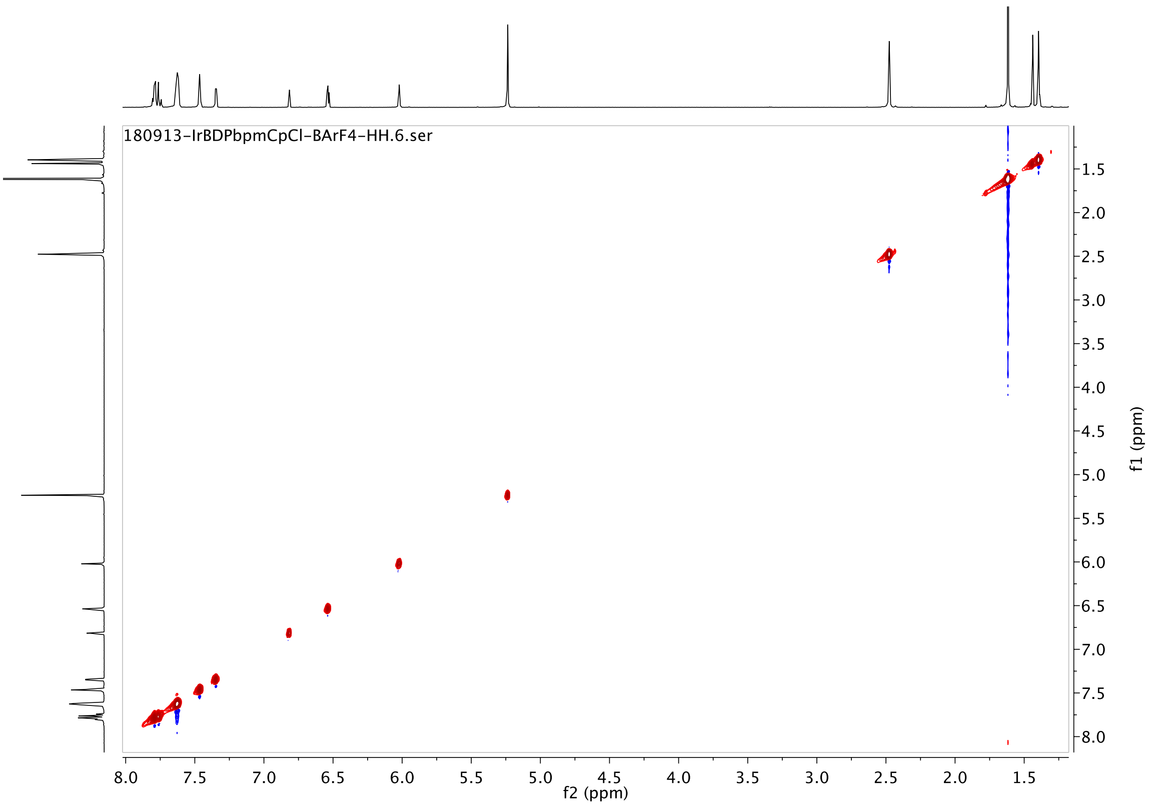

HSQC - Ir(III)-BDP HH **8** in Methylene Chloride-d_2

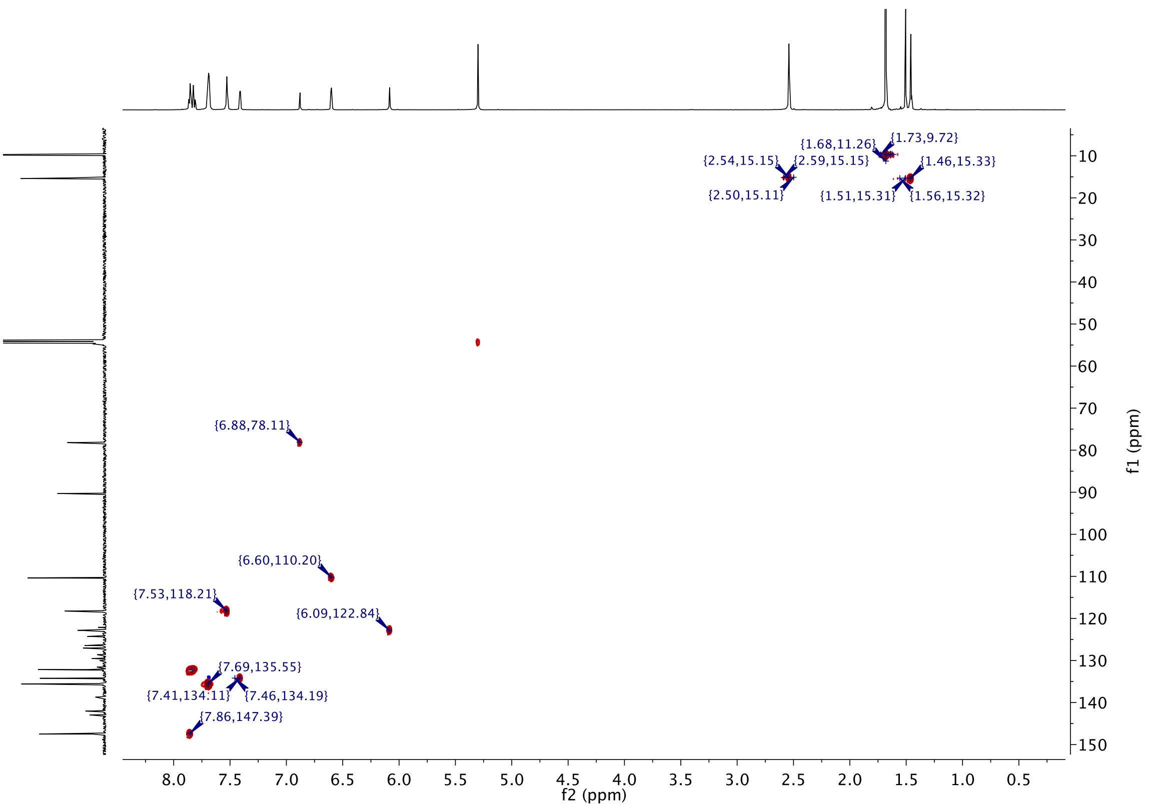

HMBC - Ir(III)-BDP HH **8** in Methylene Chloride-d_2

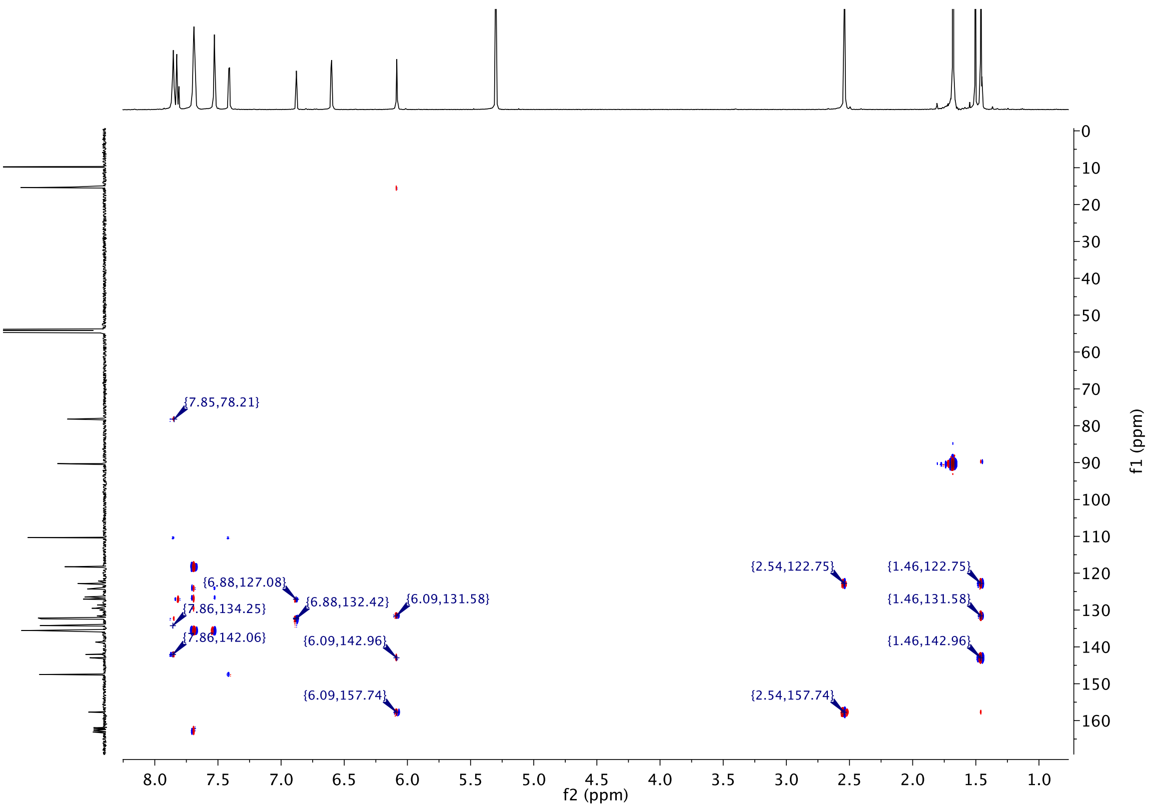

^{1}H (400 MHz, Methylene Chloride-d_2)

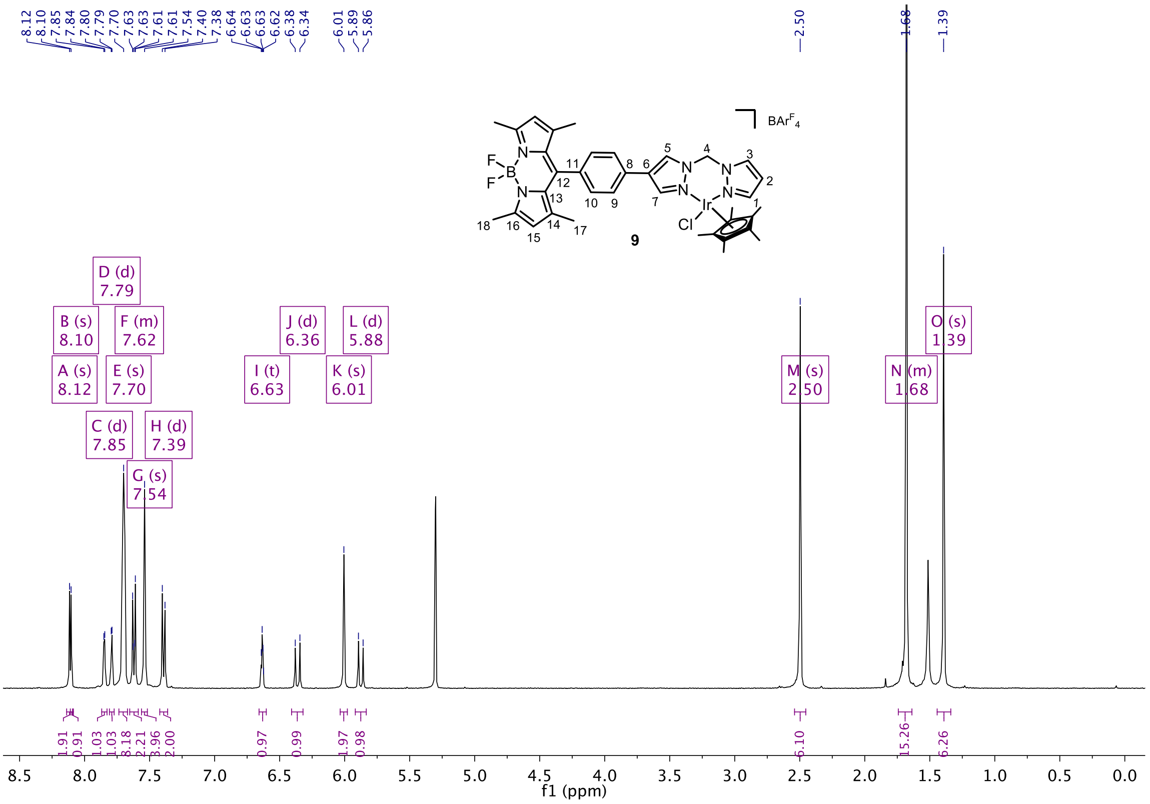

$^{13}C\{^{1}H\}$ (126 MHz, Methylene Chloride-d_2)

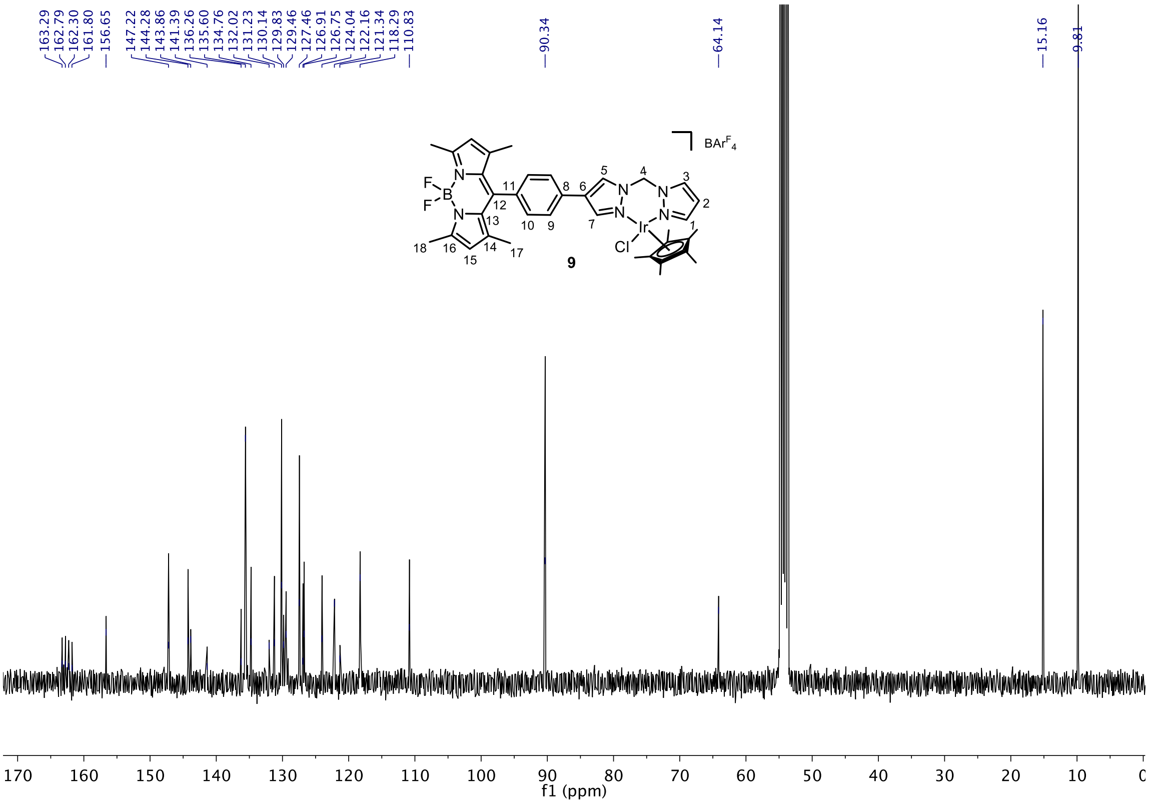

COSY - Ir(III)-BDP HS **9** in Methylene Chloride-d_2

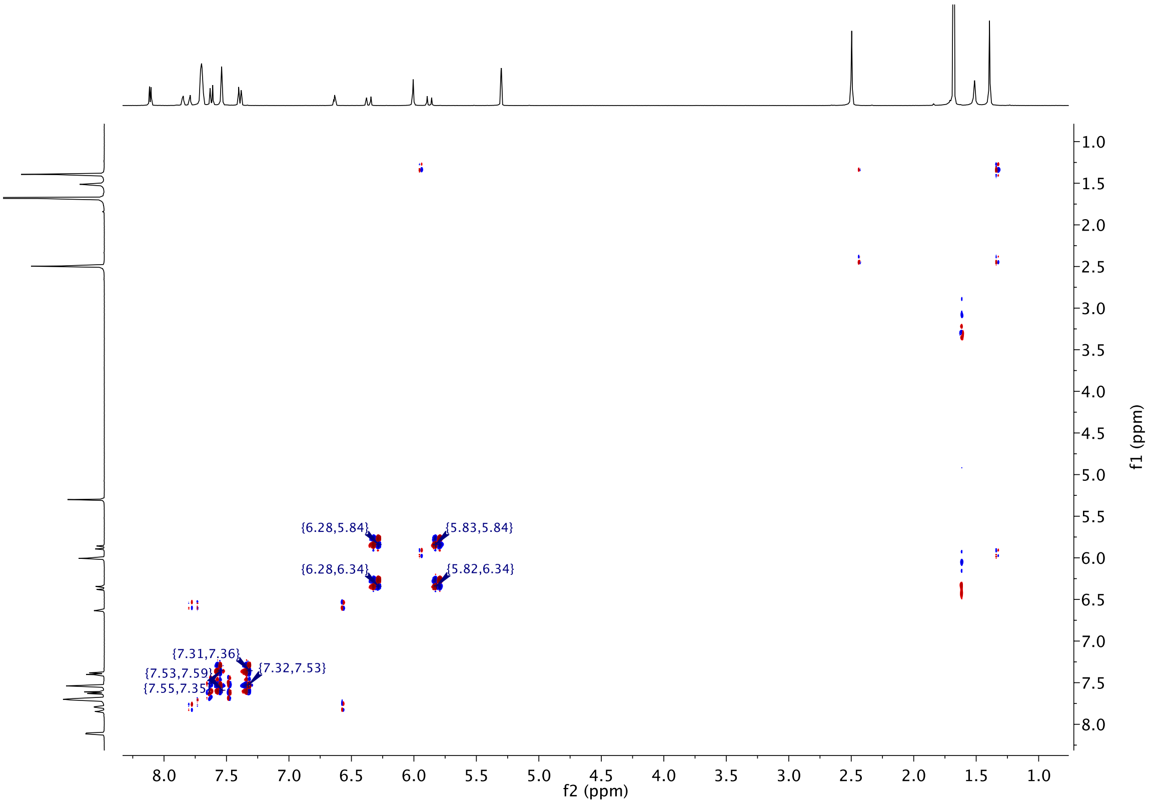

NOESY - Ir(III)-BDP HS **9** in Methylene Chloride-d_2

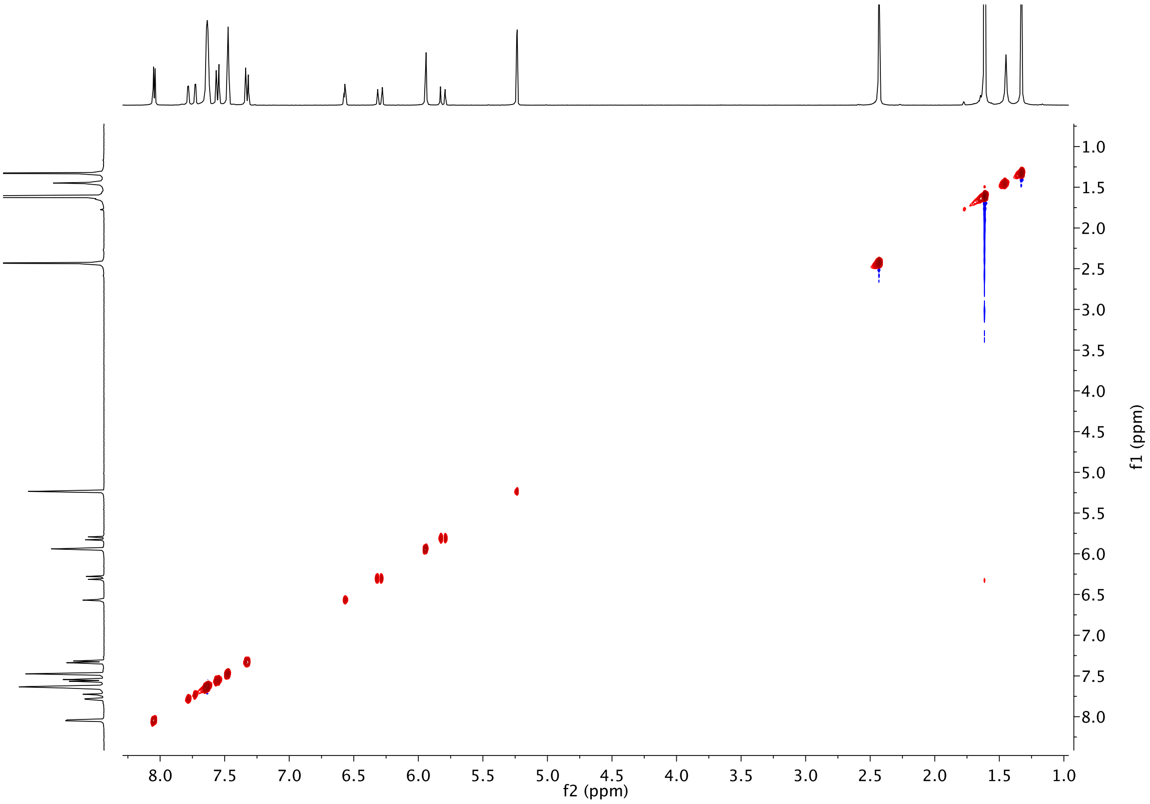

HSQC - Ir(III)-BDP HS **9** in Methylene Chloride-d_2

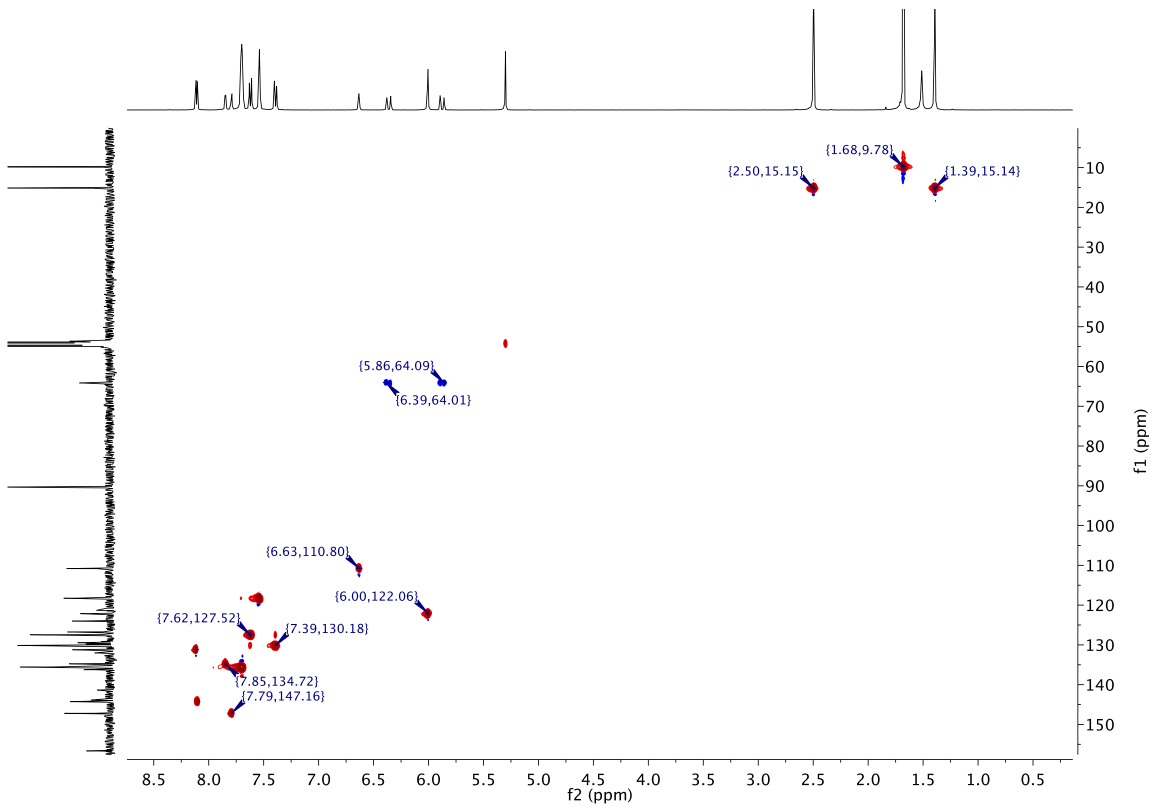

HMBC - Ir(III)-BDP HS **9** in Methylene Chloride-d_2

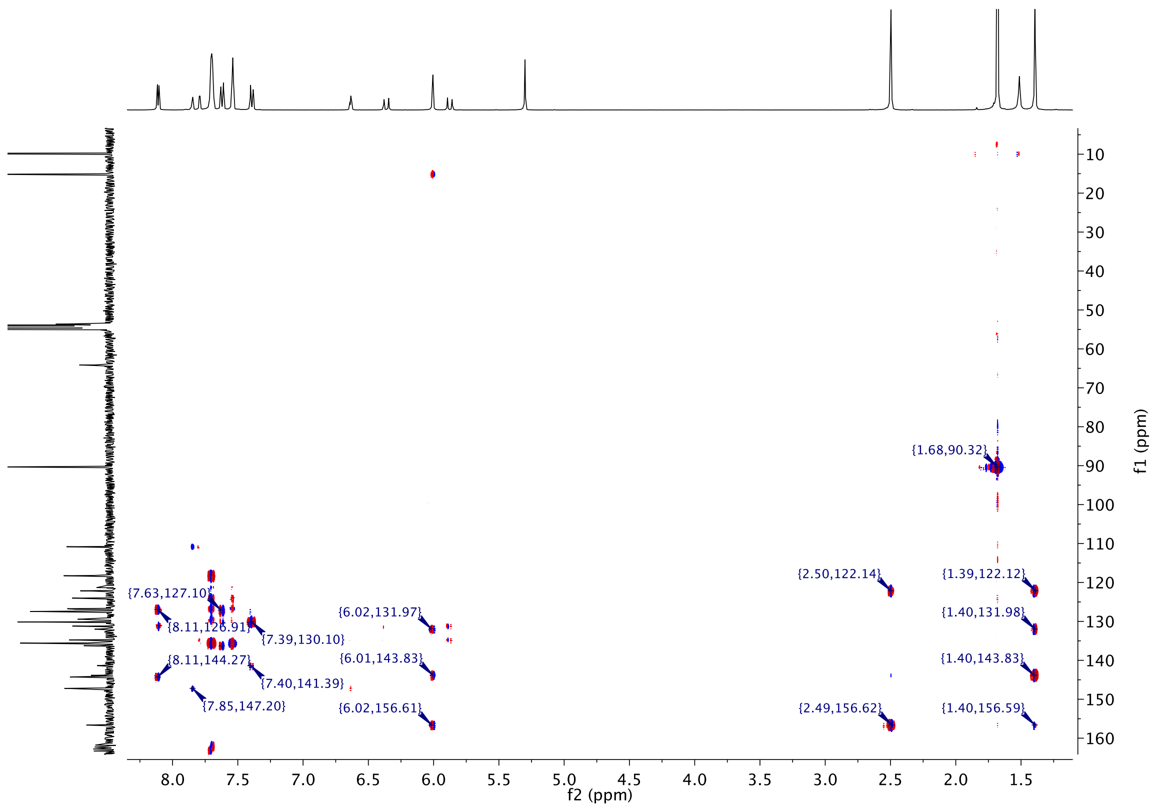

^{1}H (400 MHz, Methylene Chloride-d_2)

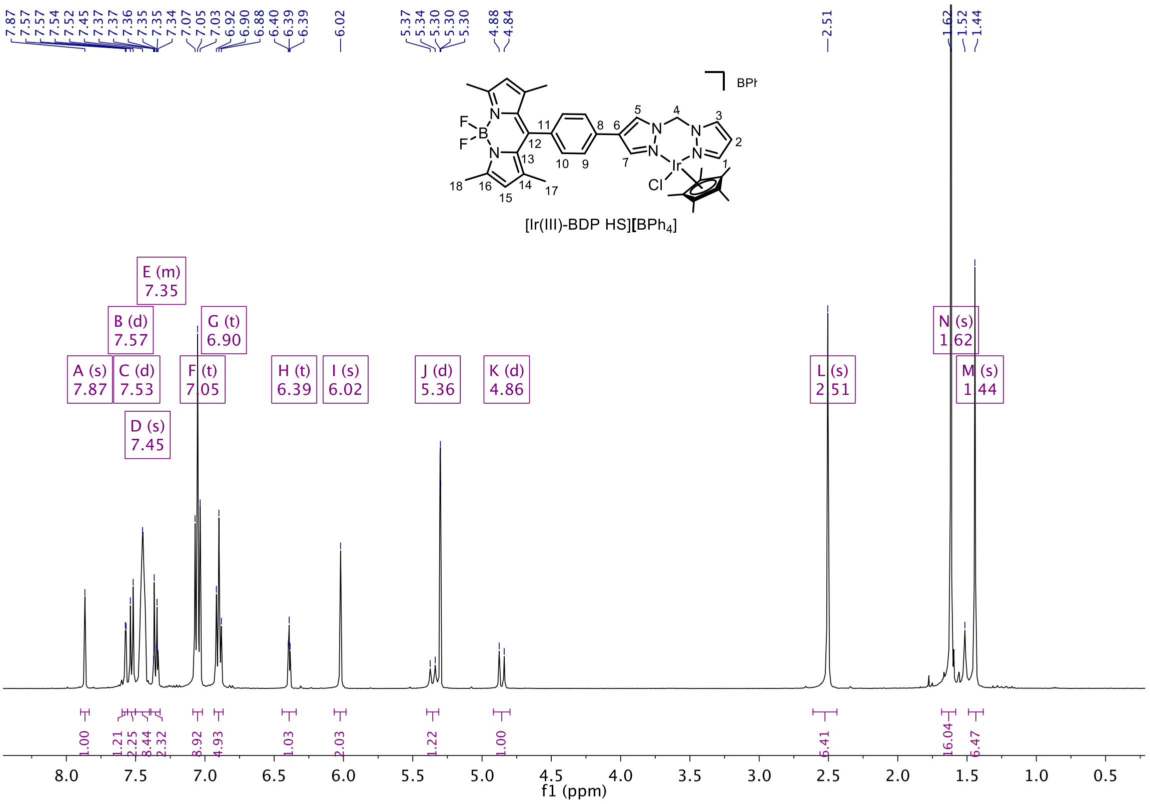

$^{13}C\{^{1}H\}$ (126 MHz, Methylene Chloride-d_2)

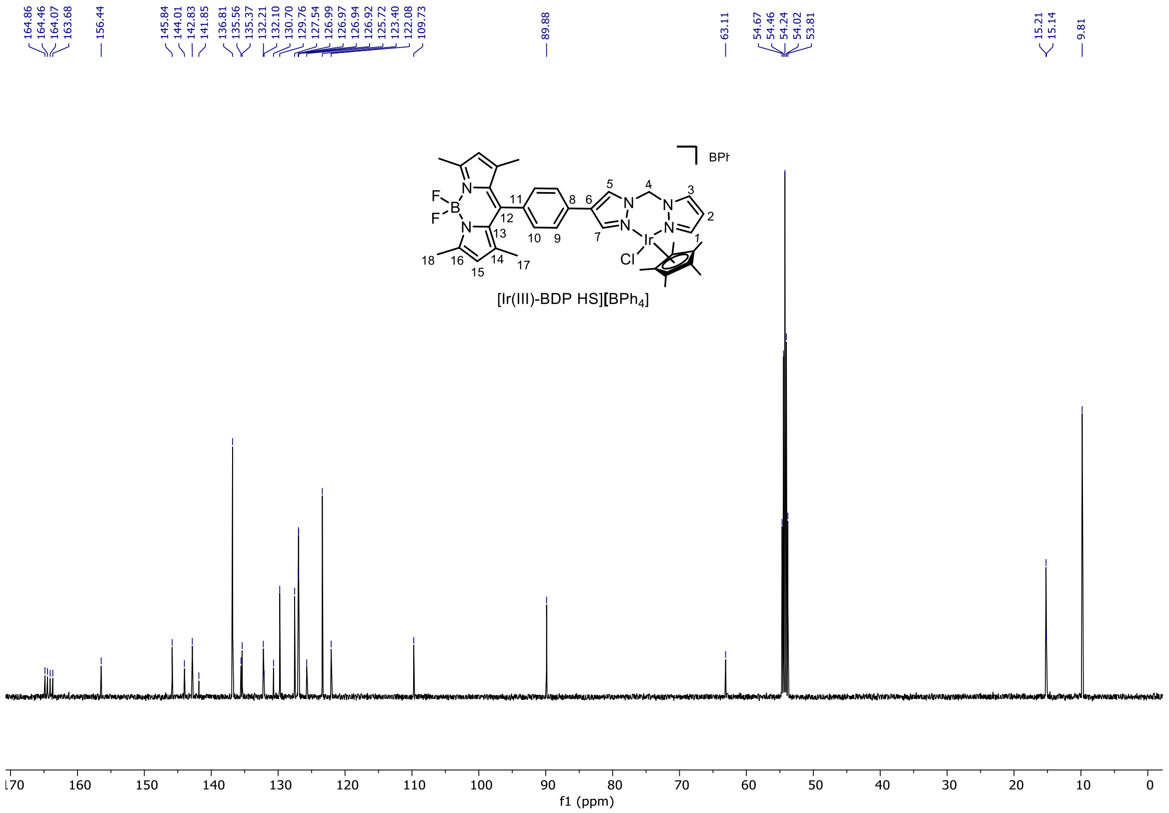

COSY – [Ir(III)-BDP HS][BPh_4] in Methylene Chloride-d_2

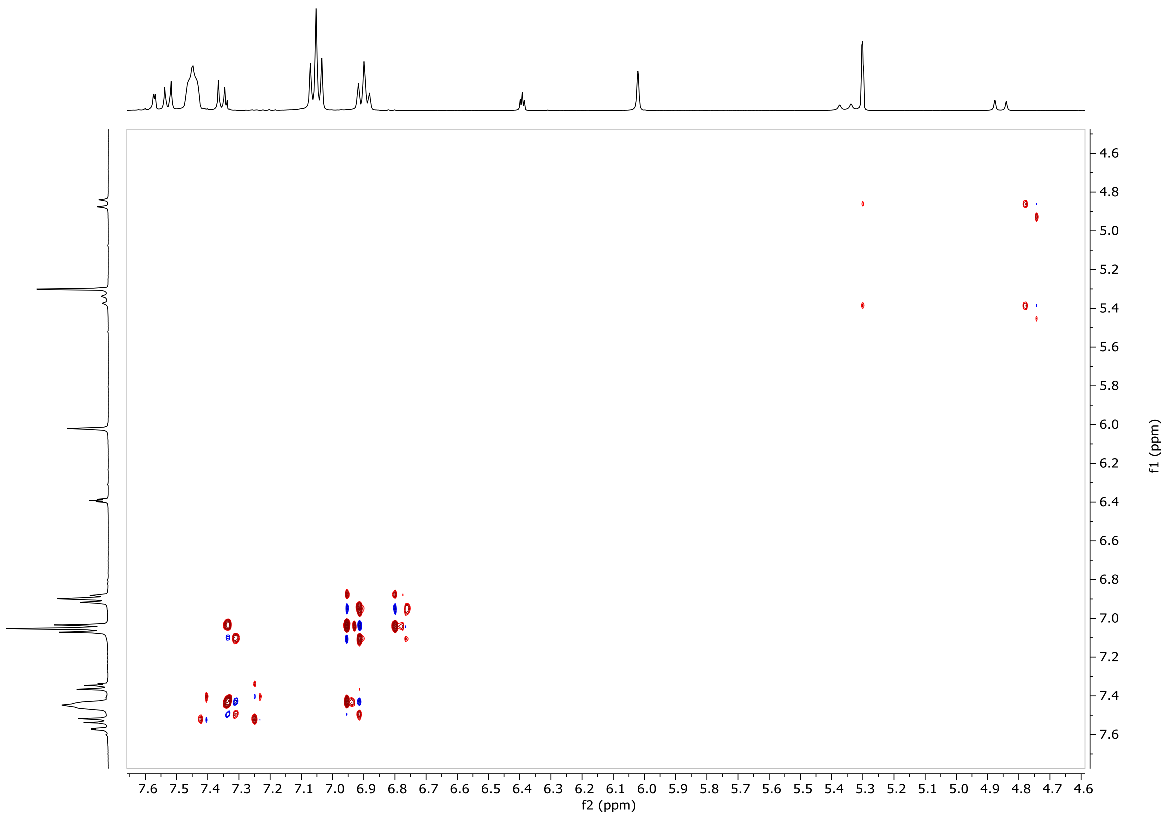

NOESY - [Ir(III)-BDP HS][BPh_4] in Methylene Chloride-d_2

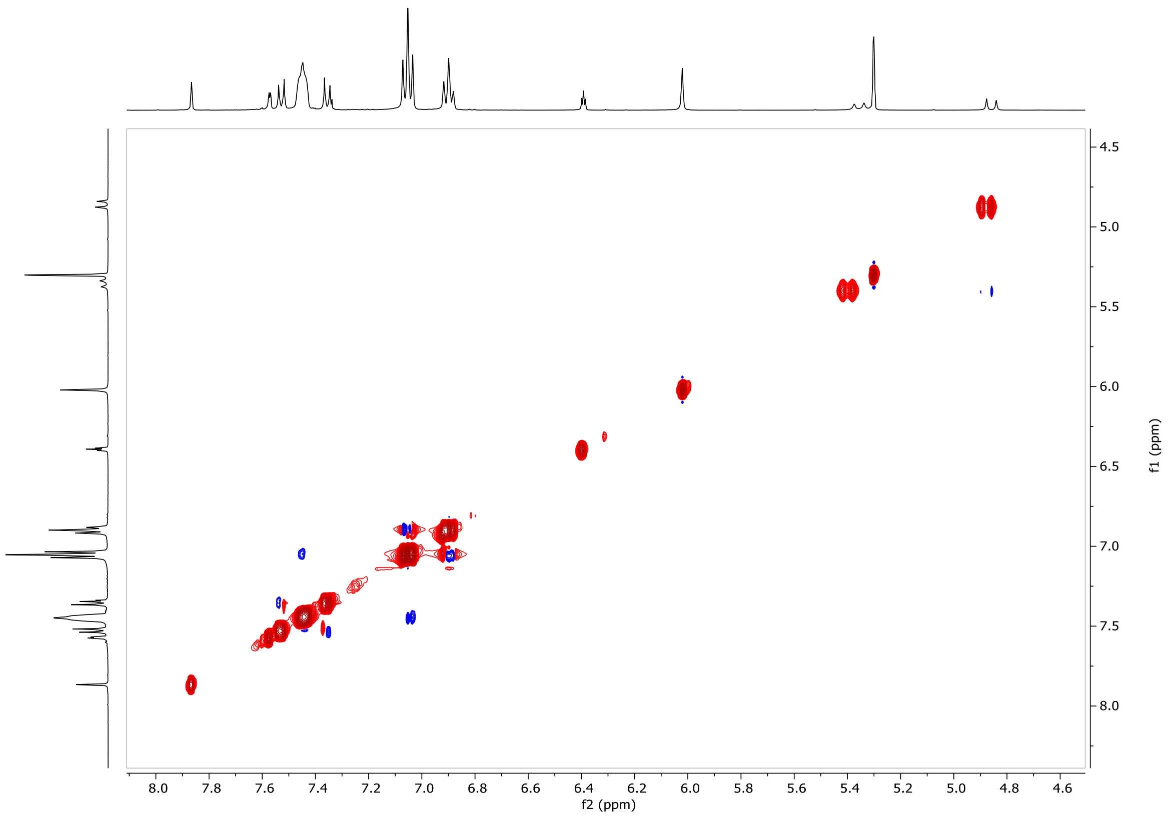

HSQC - [Ir(III)-BDP HS][BPh_4] in Methylene Chloride-d_2

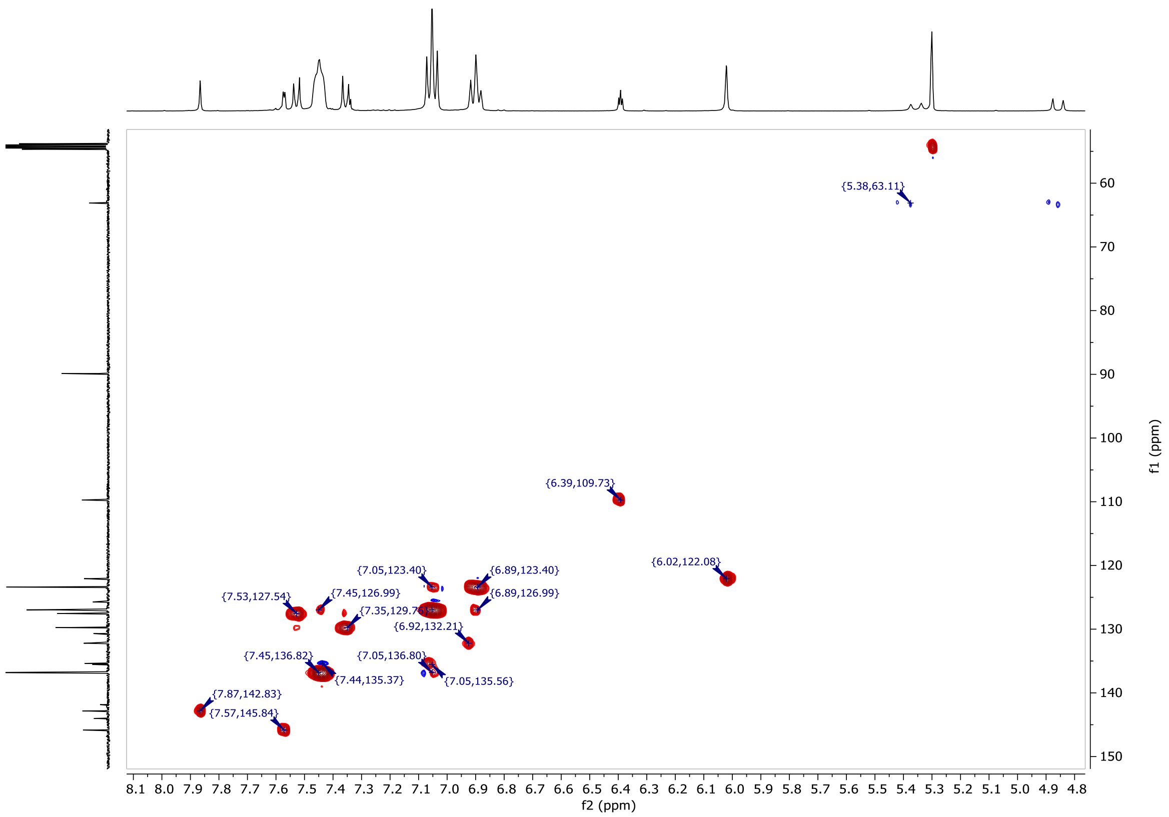

HMBC - [Ir(III)-BDP HS][BPh_4] in Methylene Chloride-d_2

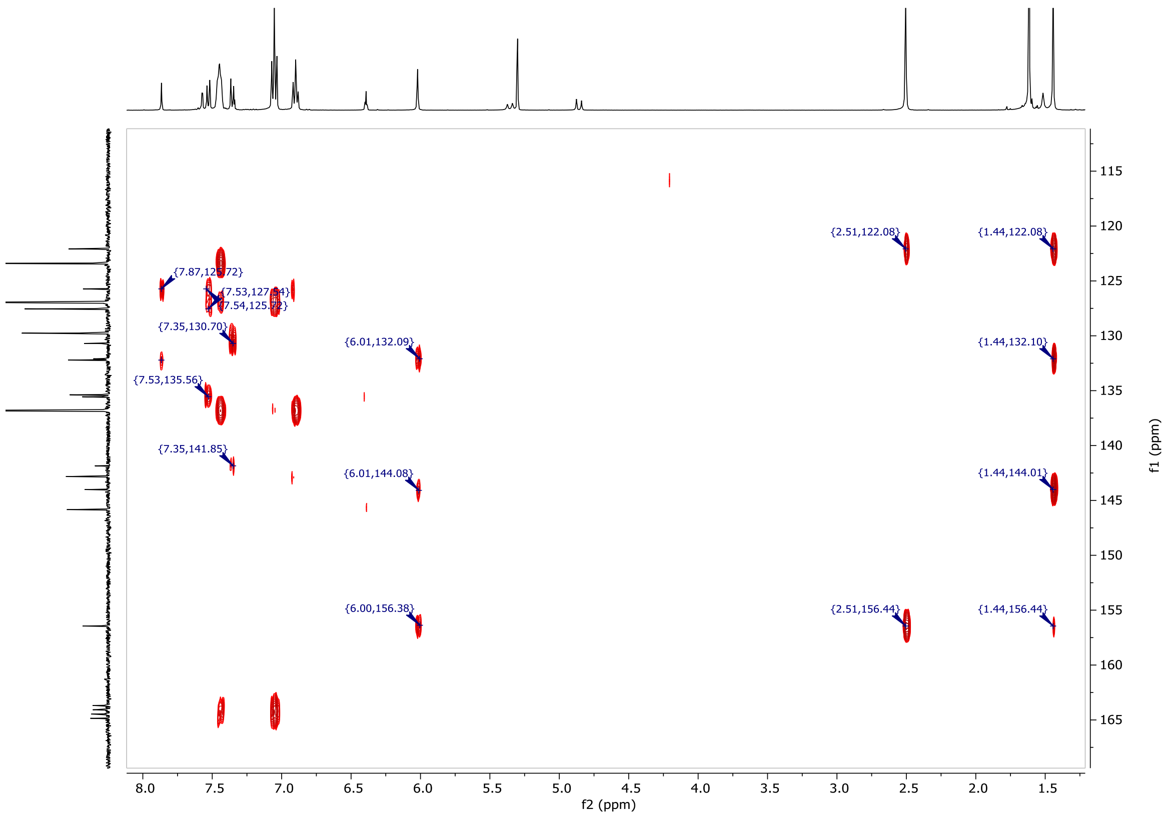

Ligands 13, 14, 17

^{1}H (400 MHz, Methylene Chloride-d_2)

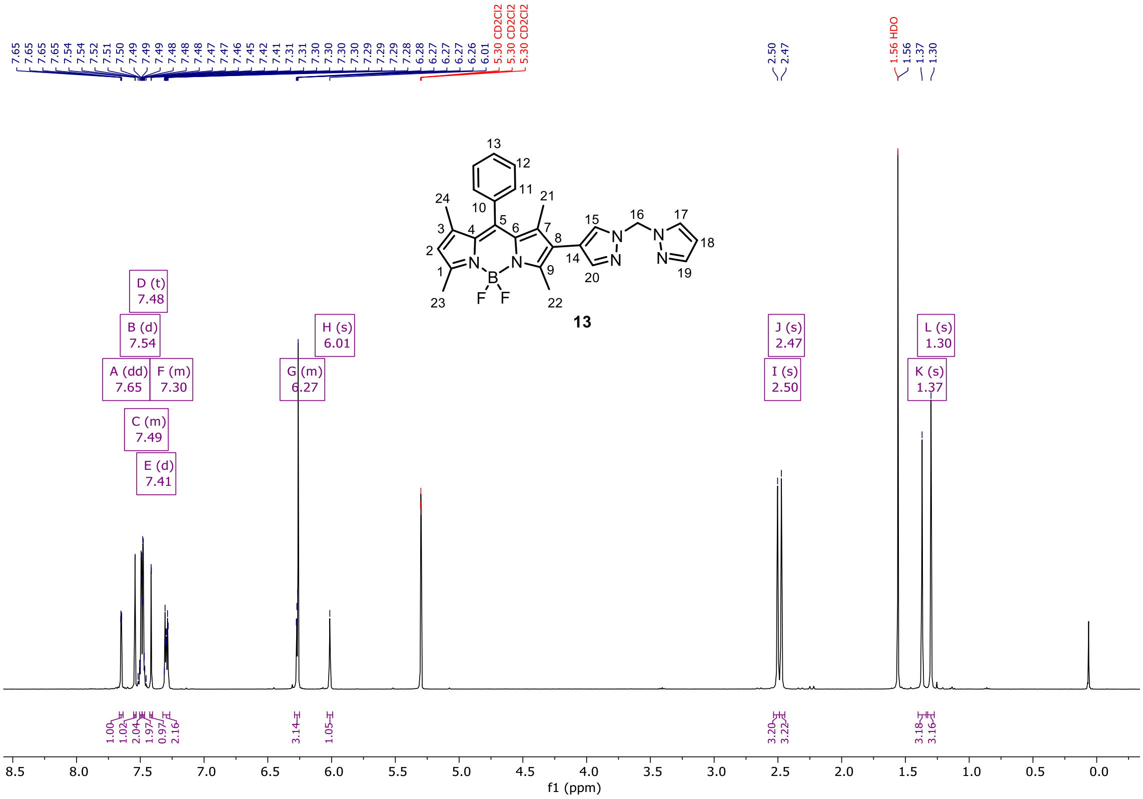

$^{13}C\{^{1}H\}$ (101 MHz, Methylene Chloride-d_2)

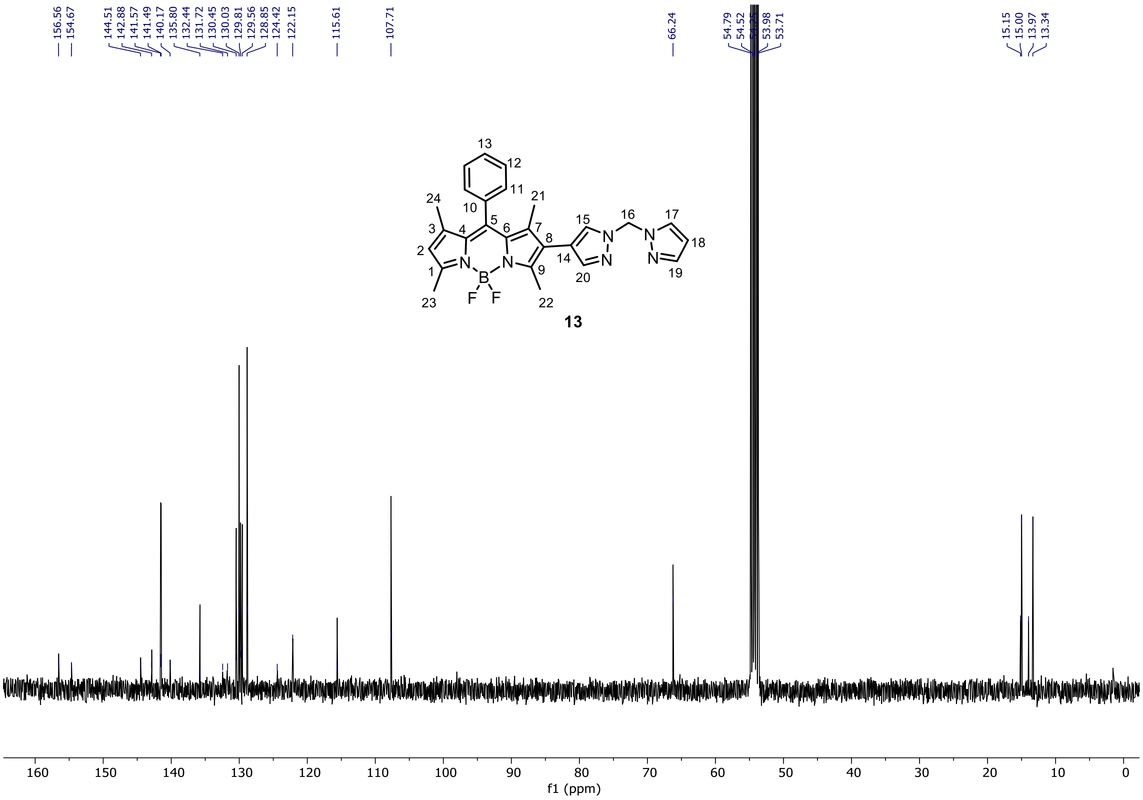

COSY - SS **13** in Methylene Chloride-d_2

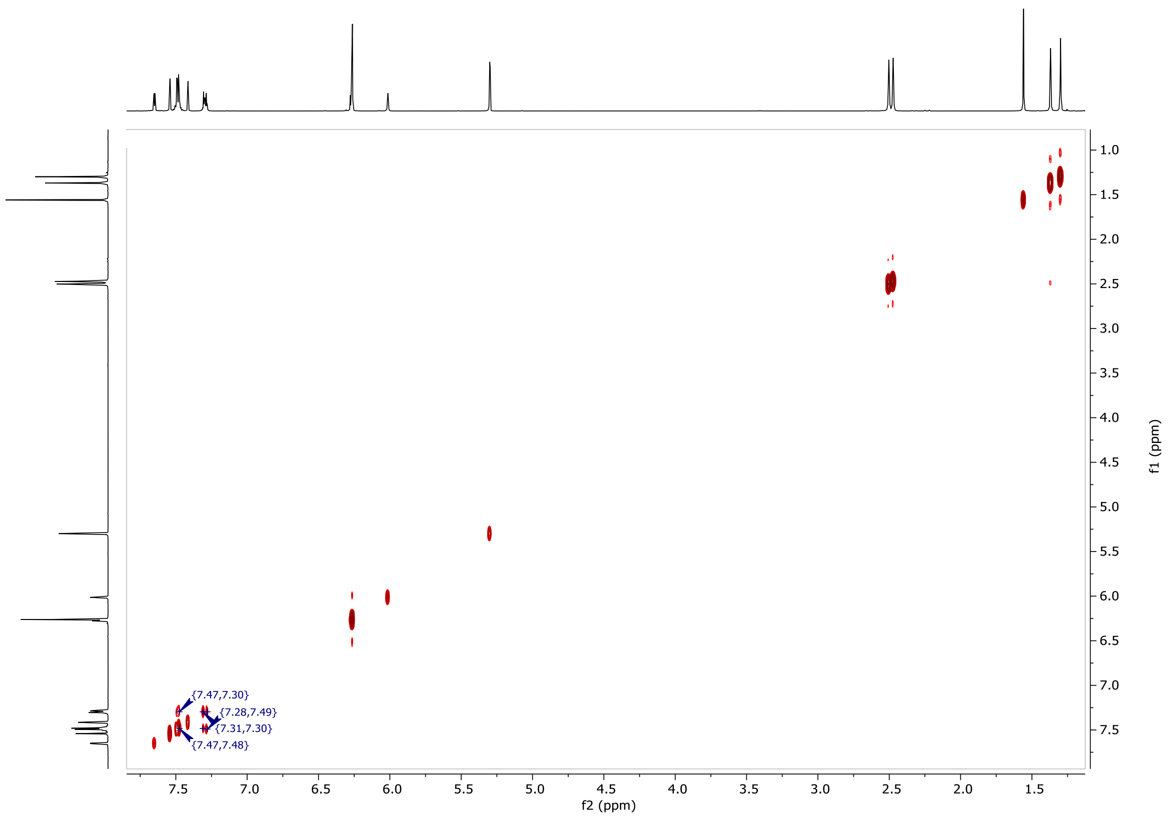

NOESY- SS **13** in Methylene Chloride-d_2

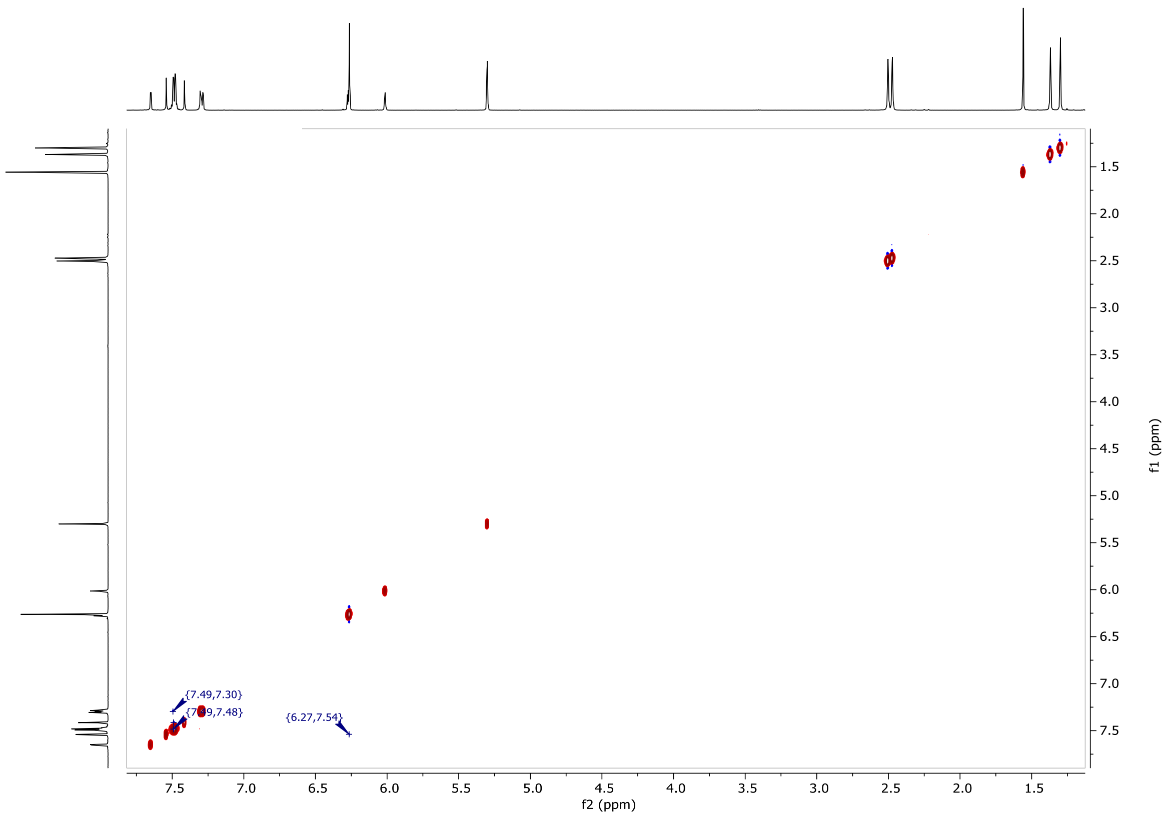

HMBC - SS **13** in Methylene Chloride-d_2

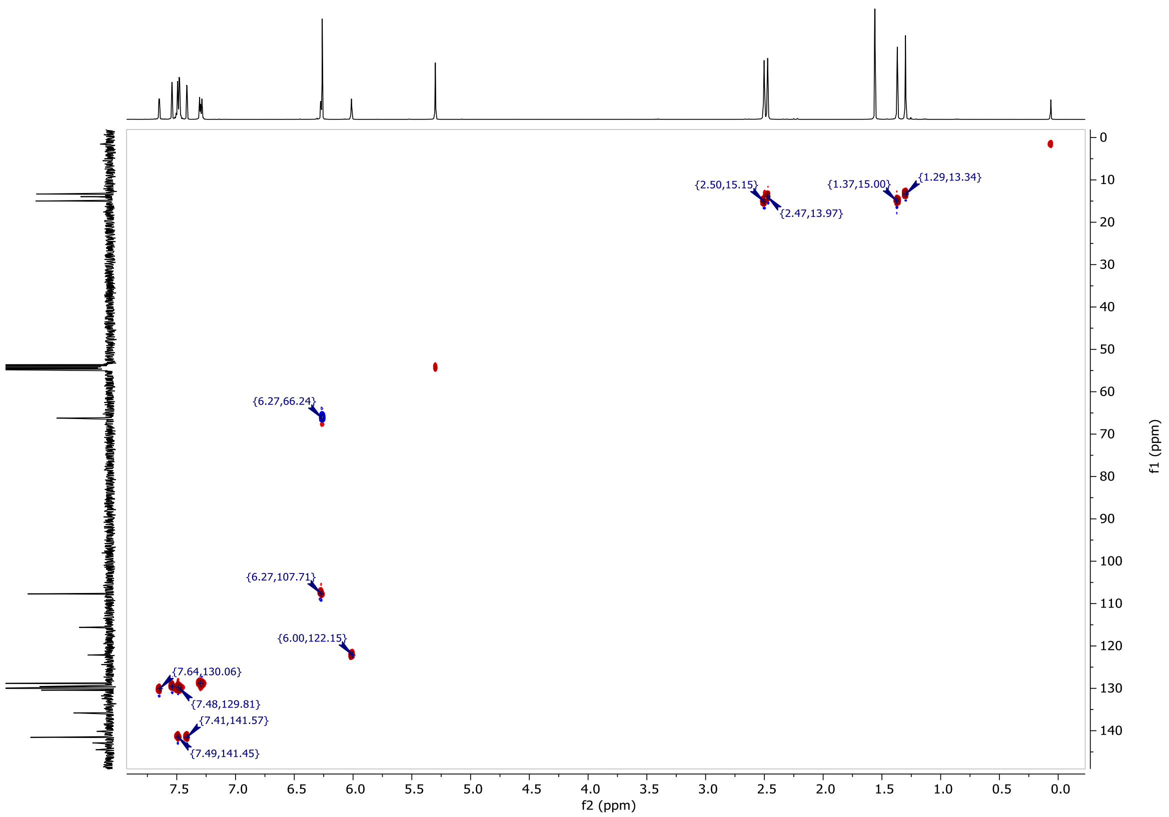

HSQC - SS **13** in Methylene Chloride-d_2

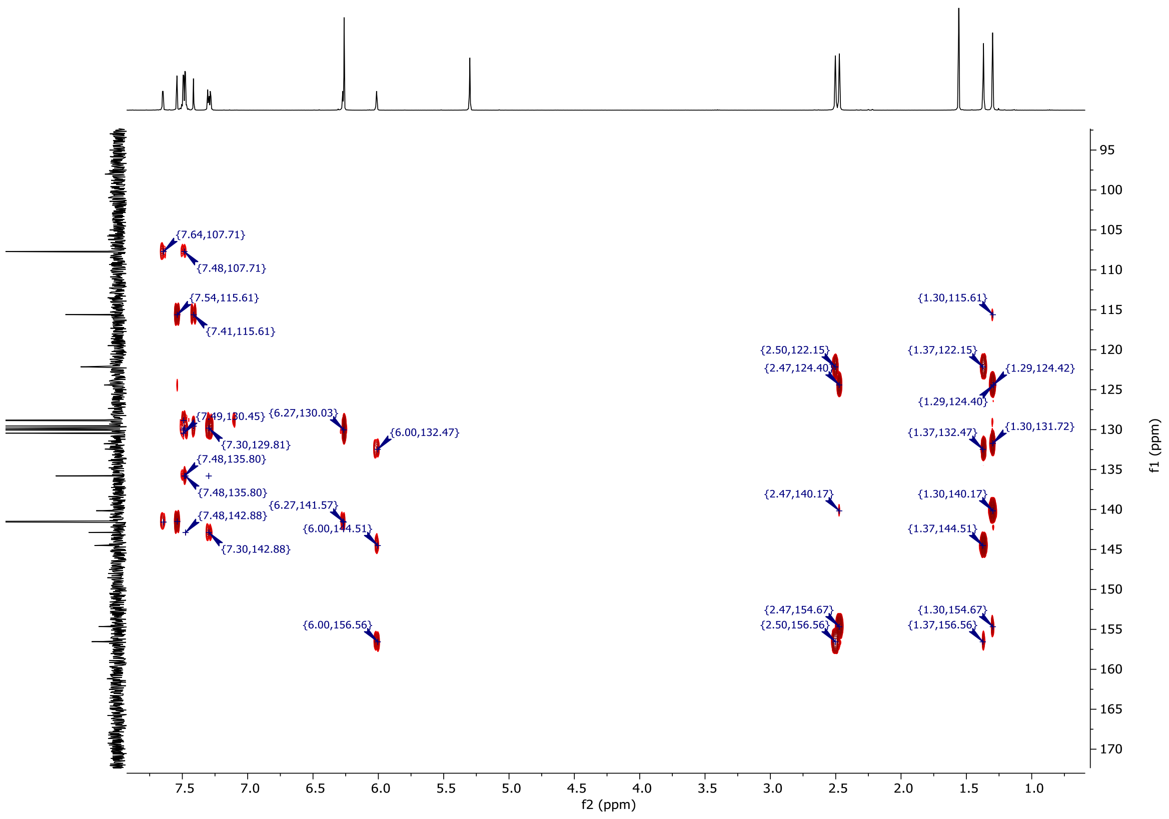

^{1}H (400 MHz, Methylene Chloride-d_2)

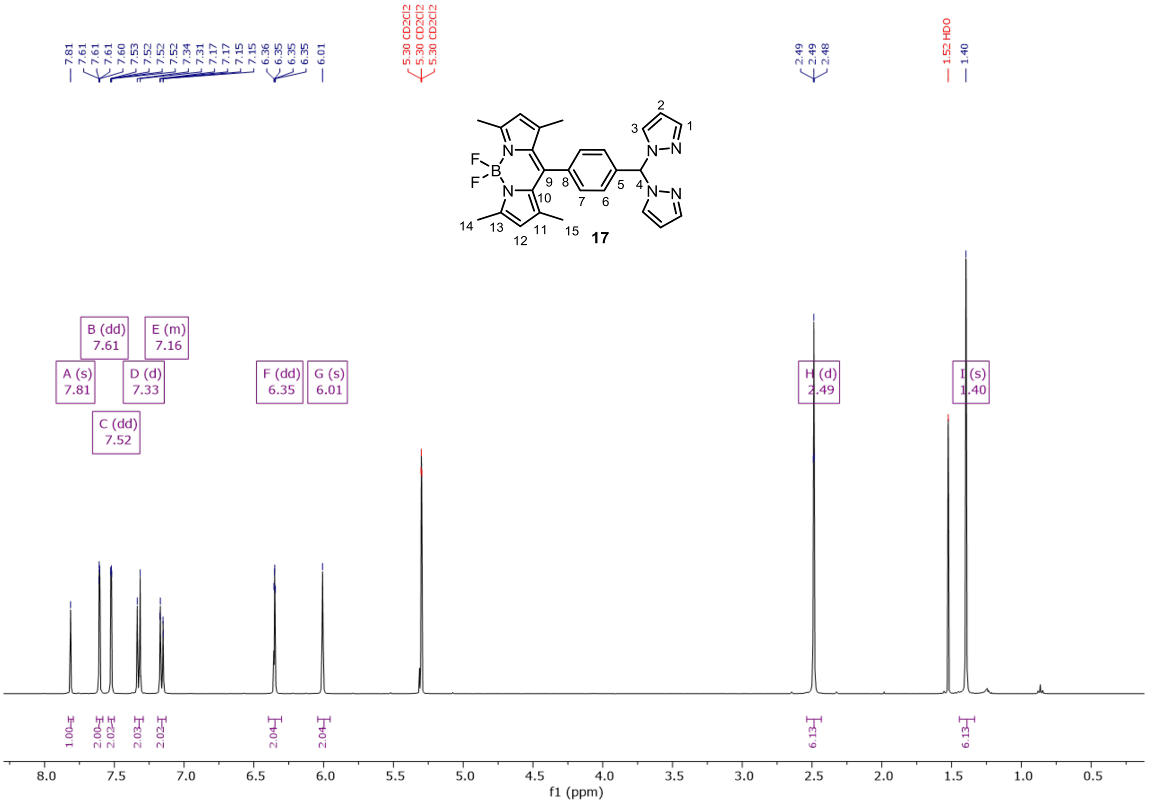

^{13}C{^{1}H} (101 MHz, Methylene Chloride-d_2)

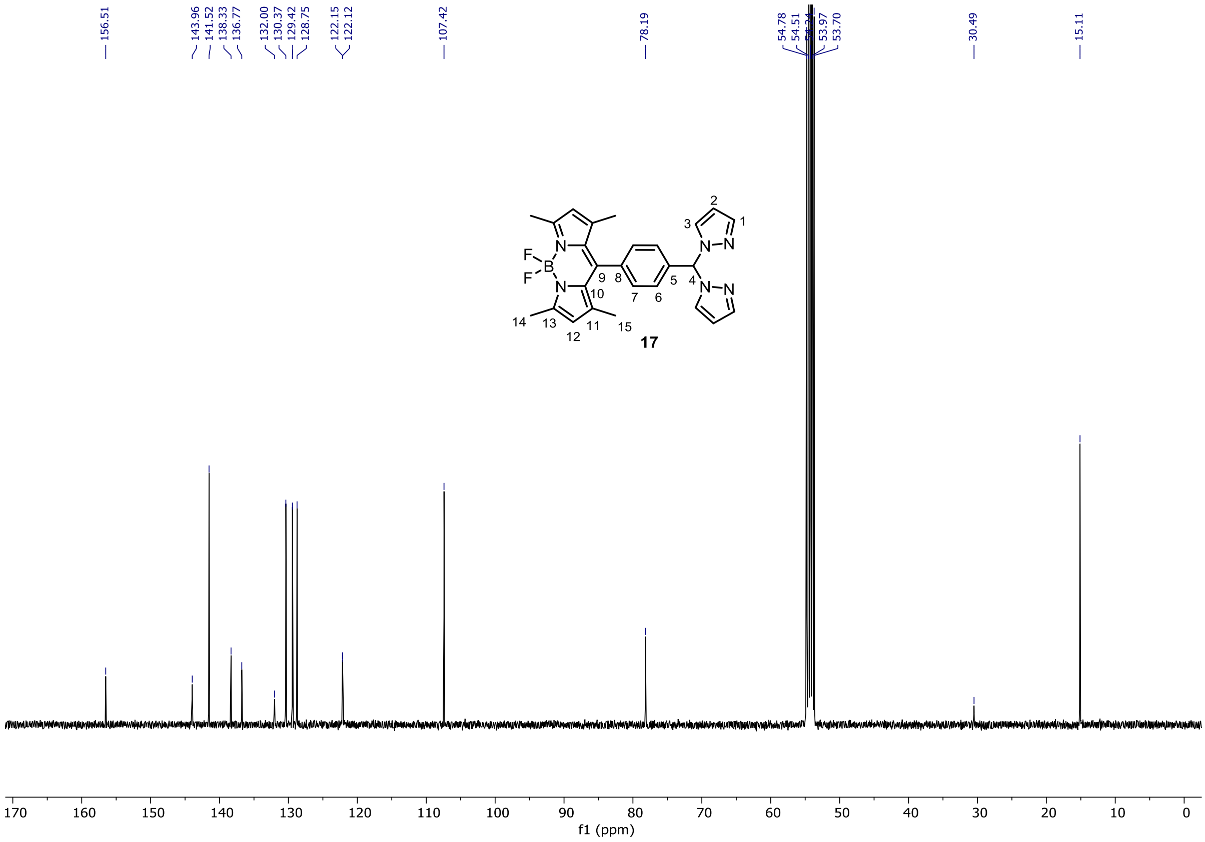

COSY - HH **17** in Methylene Chloride-d_2

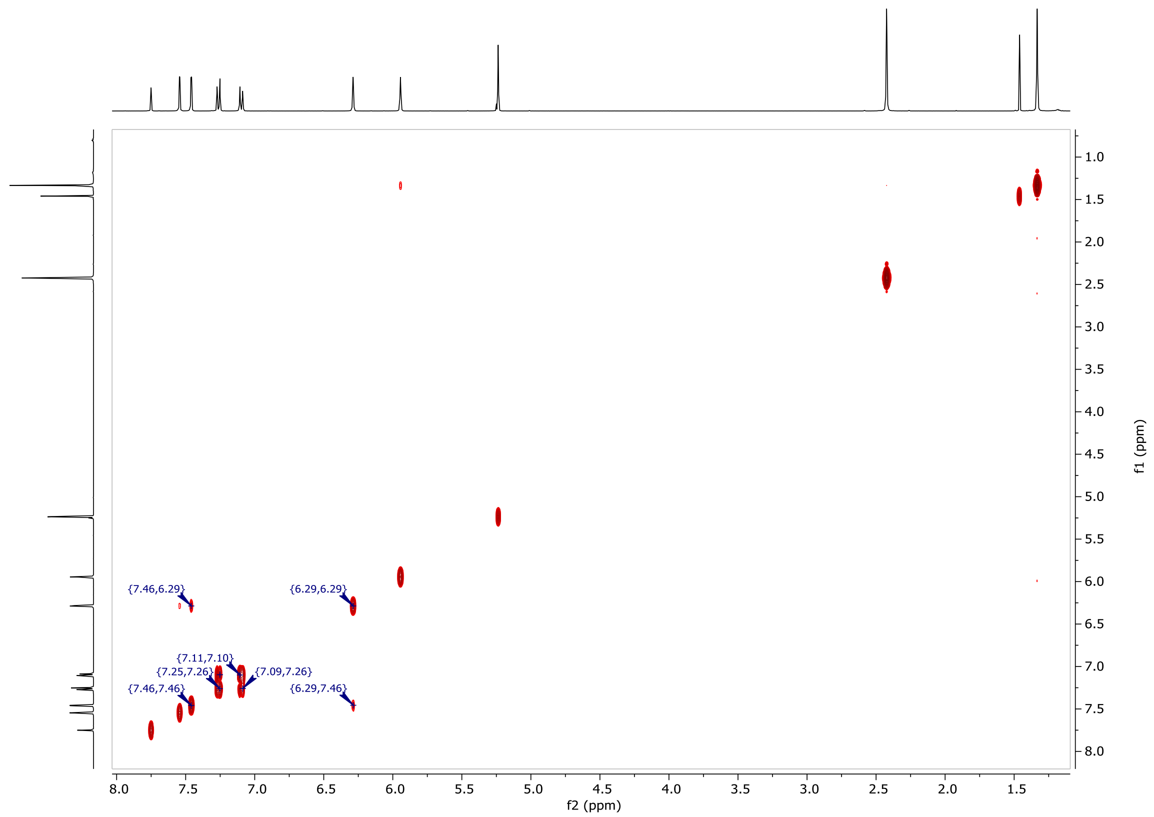

NOESY - HH **17** in Methylene Chloride-d_2

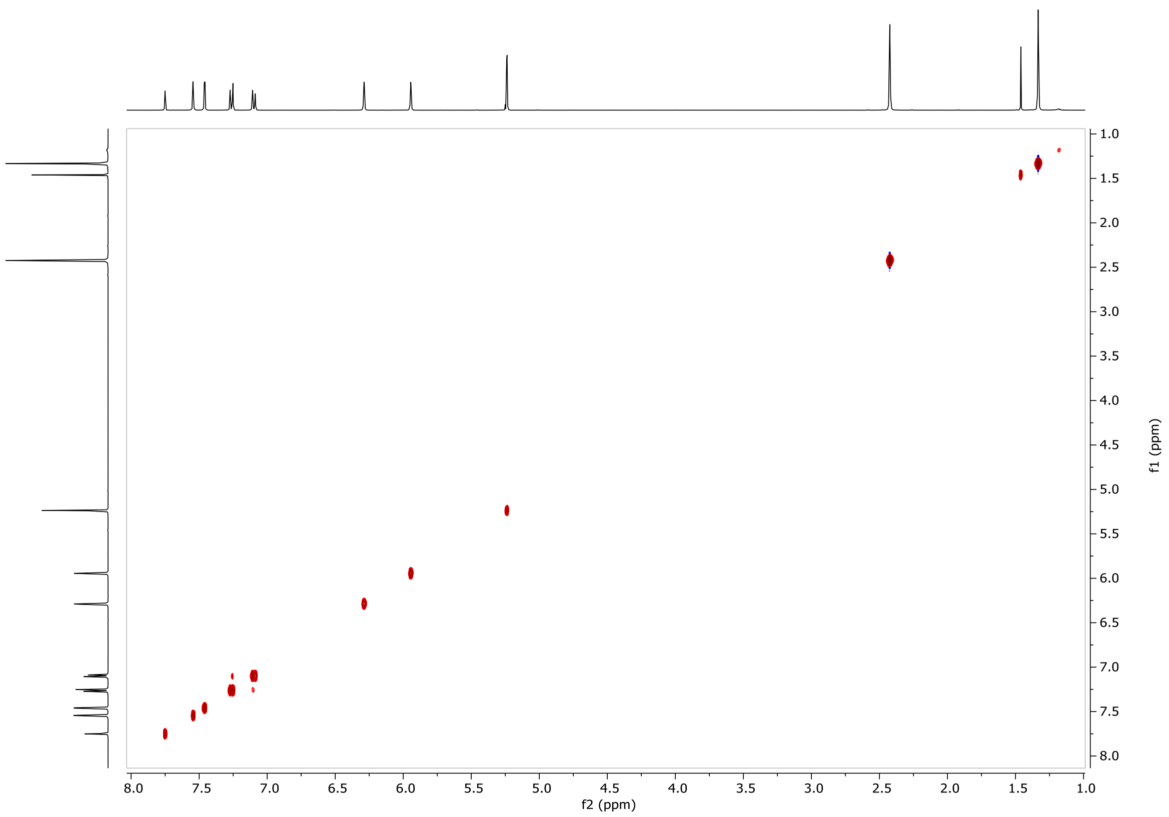

HSQC - HH **17** in Methylene Chloride-d_2

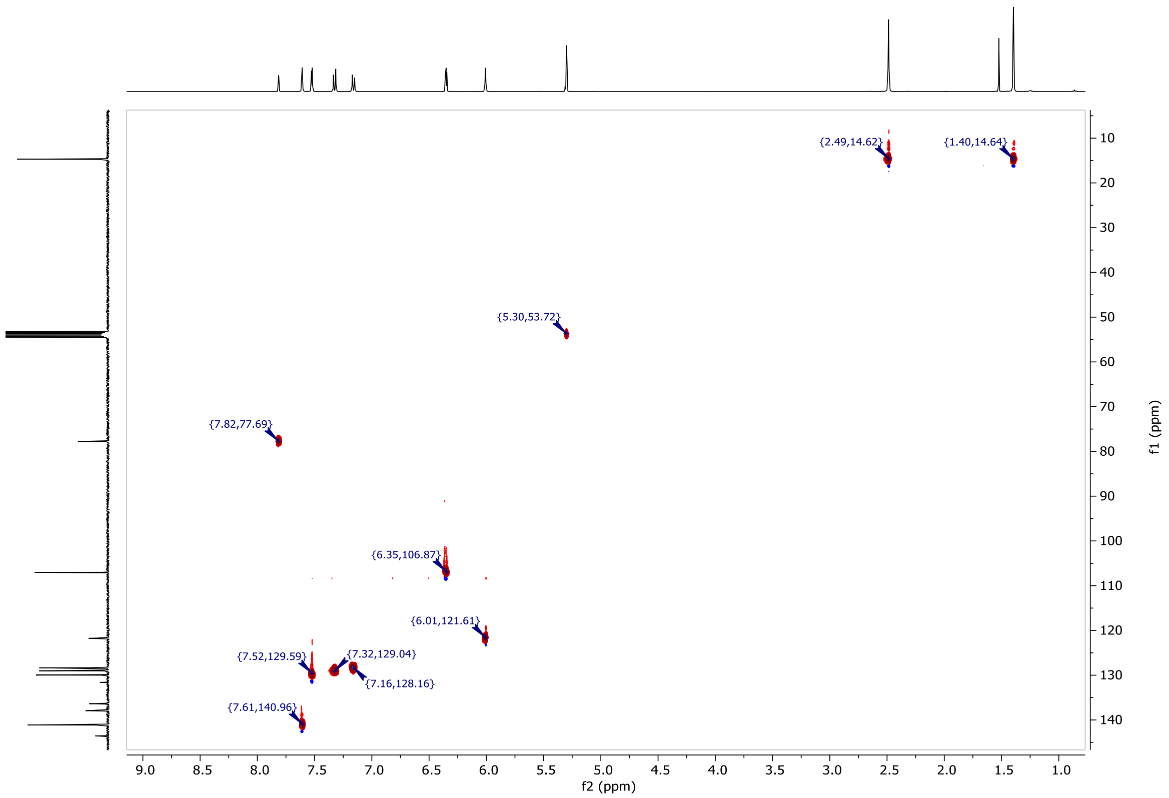

HMBC - HH **17** in Methylene Chloride-d_2

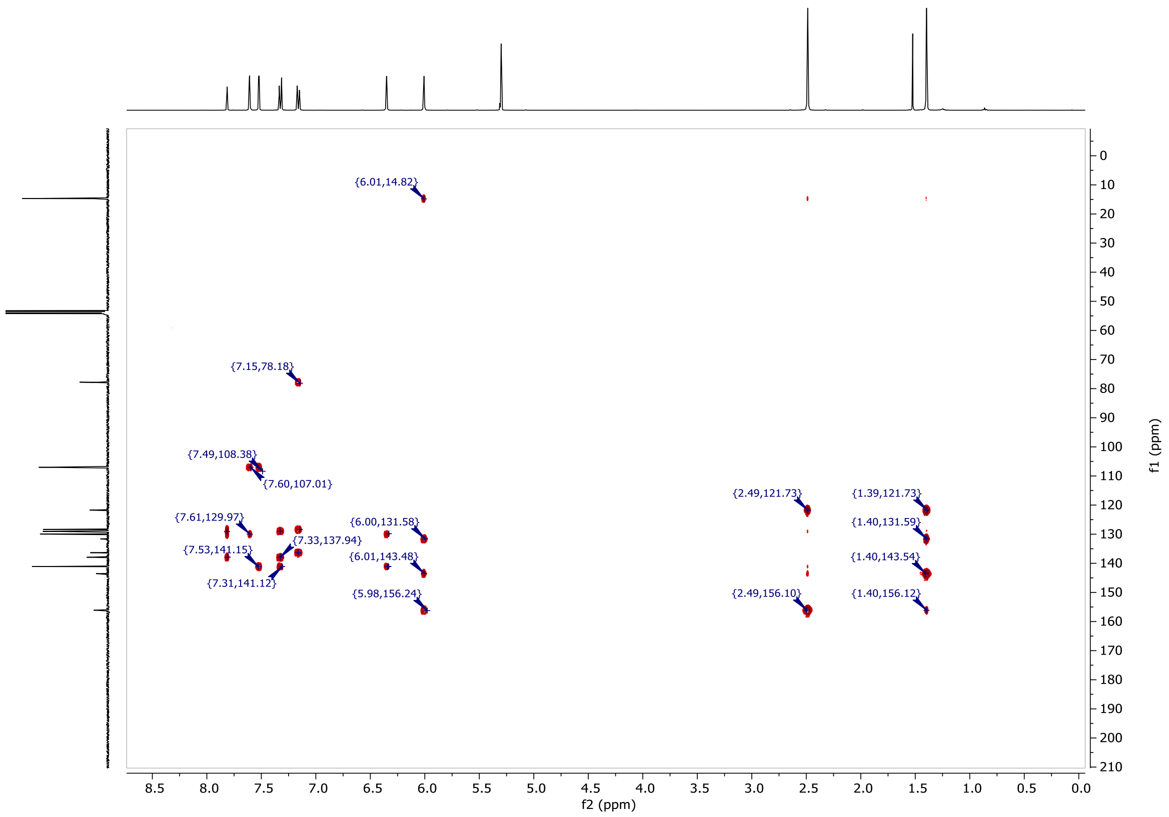

^{1}H (400 MHz, Methylene Chloride-d_2)

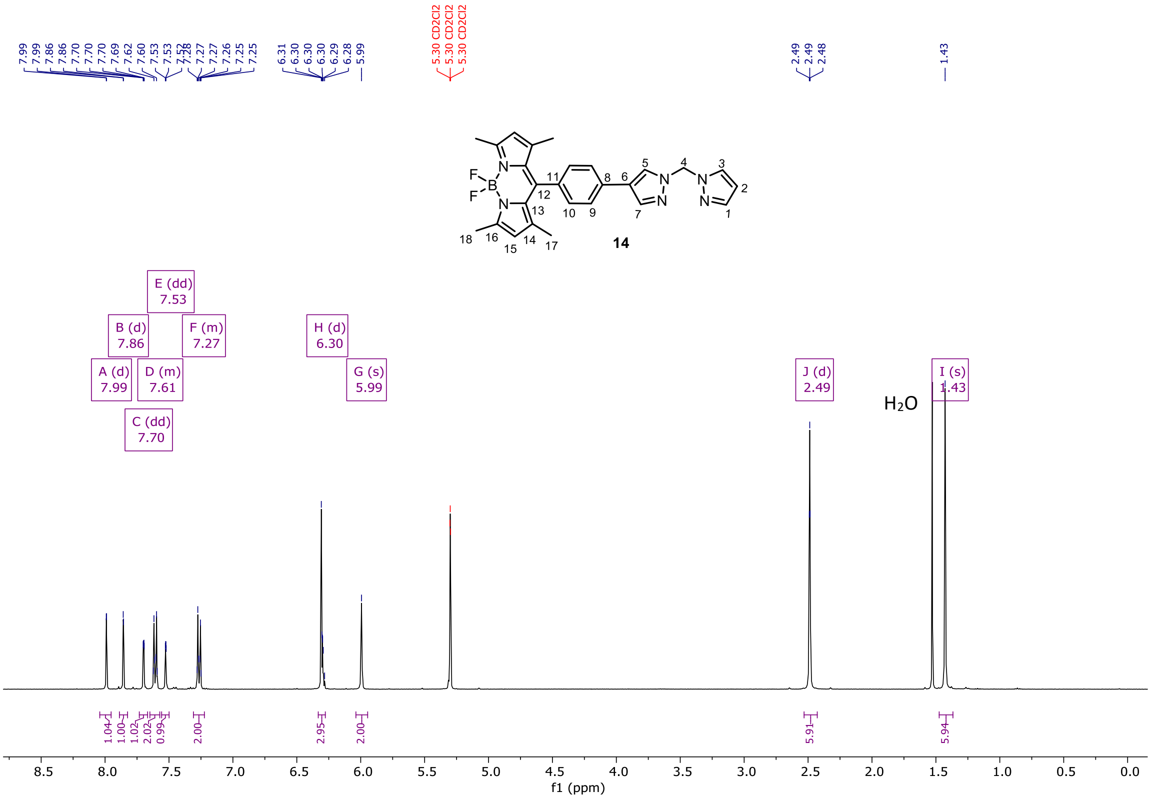

$^{13}C\{^{1}H\}$ (101 MHz, Methylene Chloride-d_2)

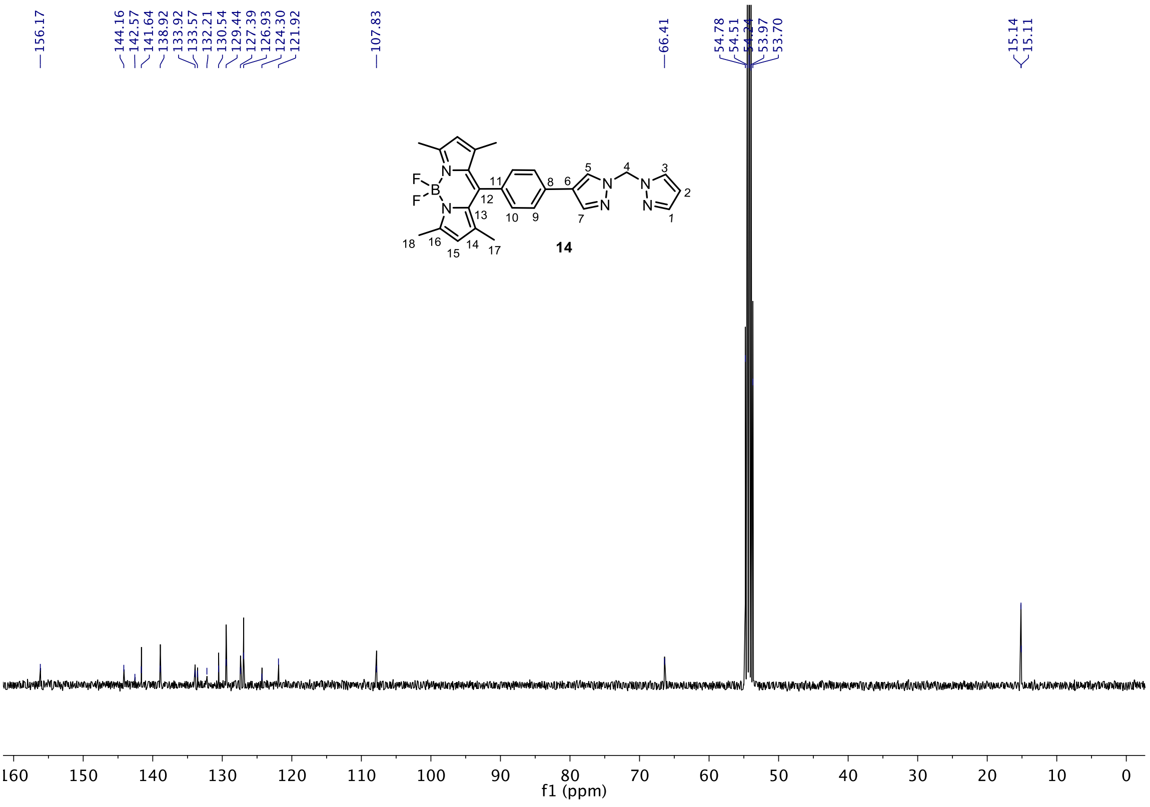

COSY - HS **14** in Methylene Chloride-d_2

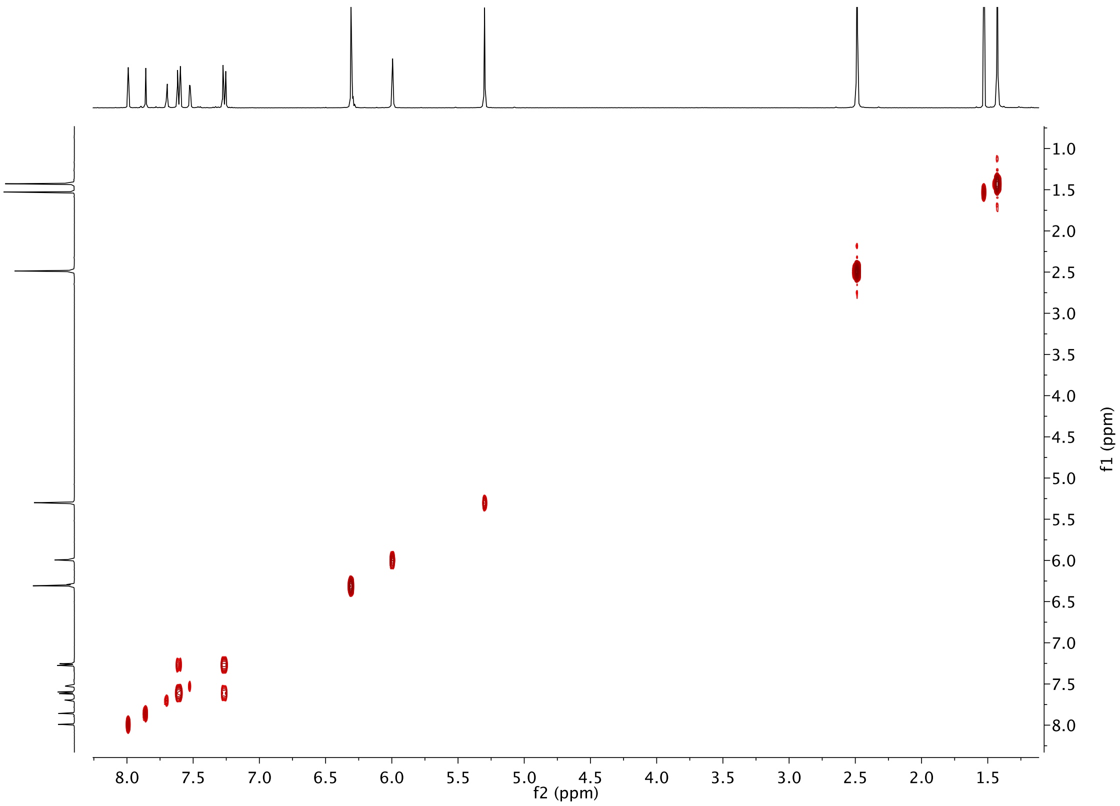

NOESY - HS **14** in Methylene Chloride-d_2

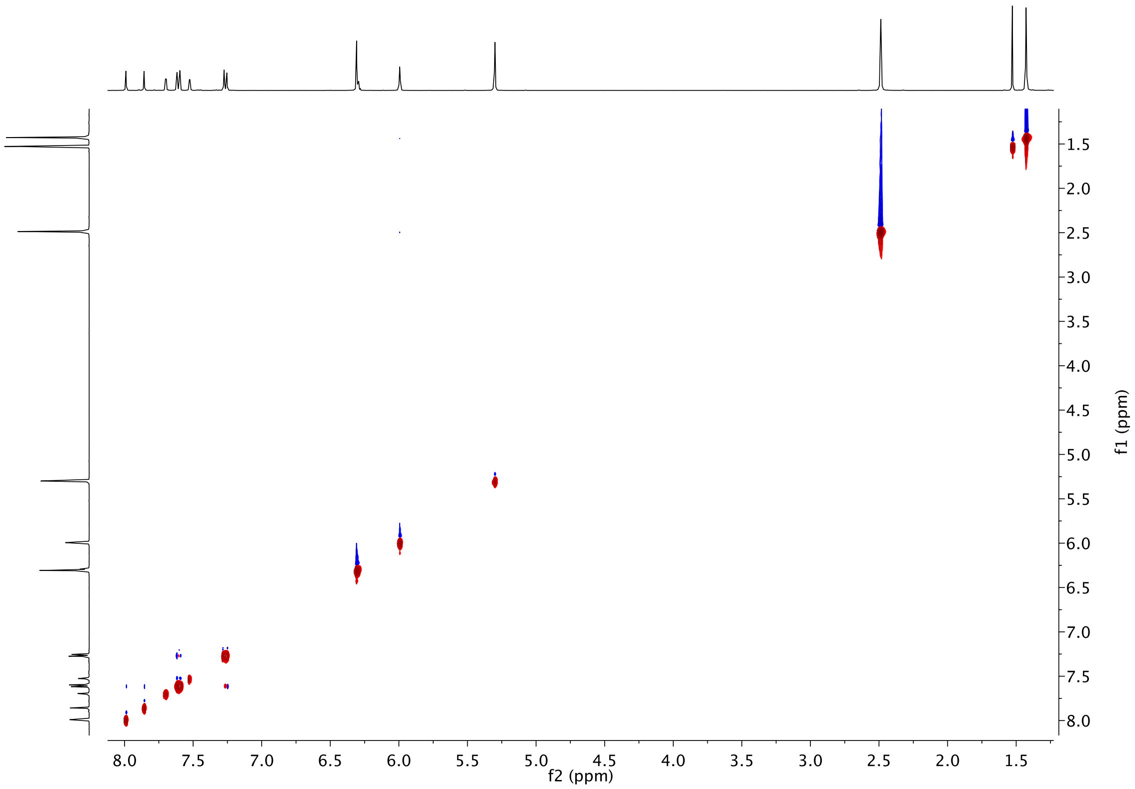

HSQC - HS **14** in Methylene Chloride-d_2

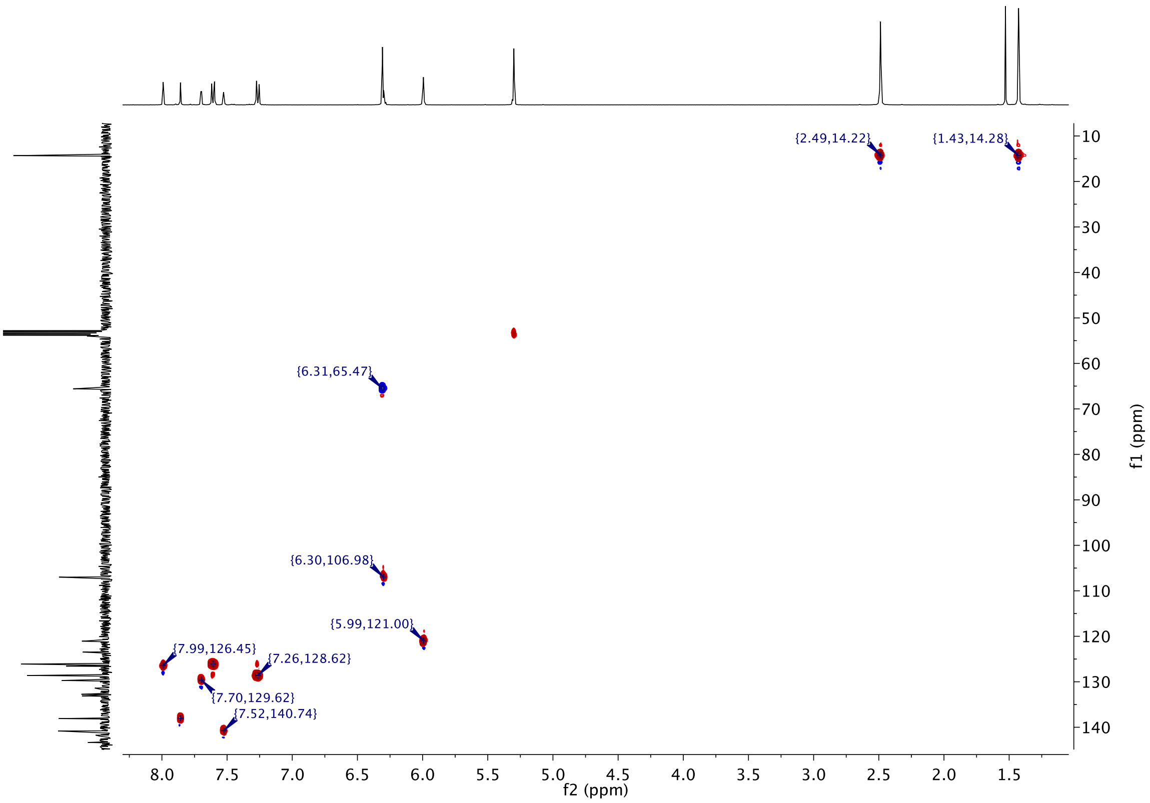

HMBC - HS **14** in Methylene Chloride-d_2

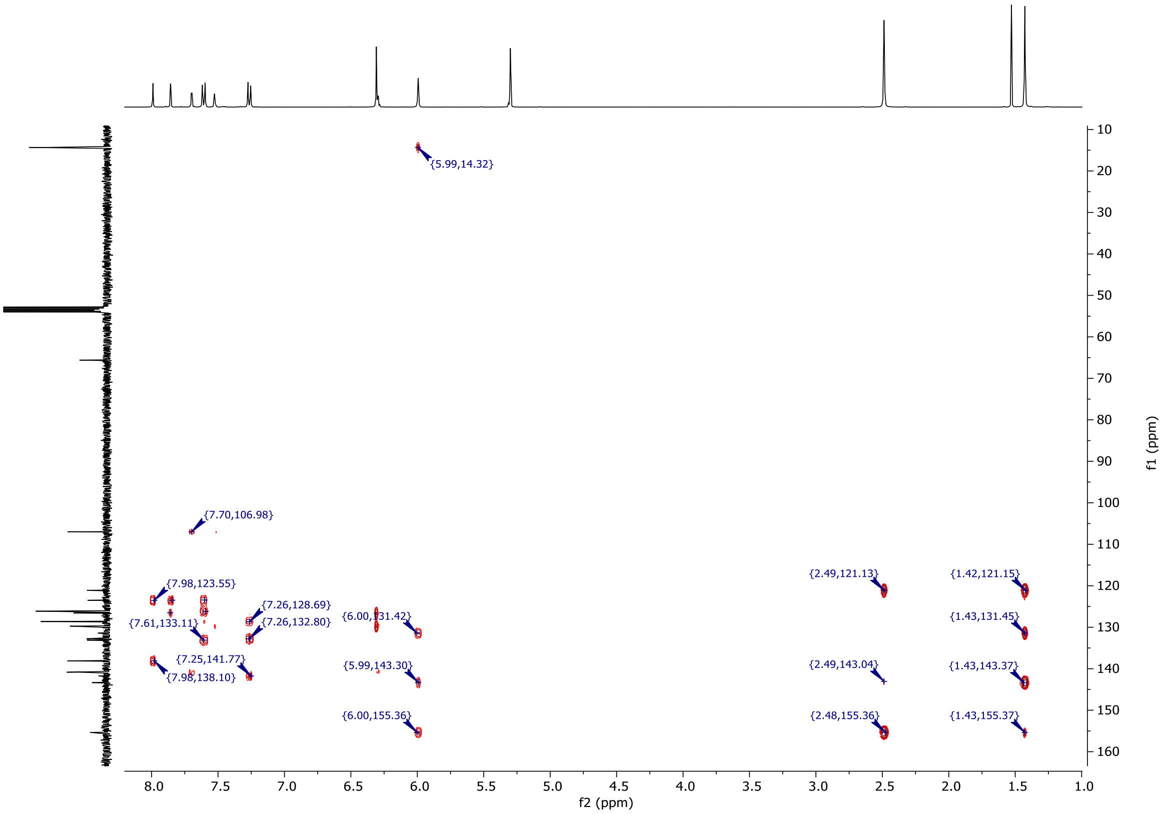

Intermediates 12, 30 and substrate 25

^{1}H (400 MHz, Methylene Chloride-d_2)

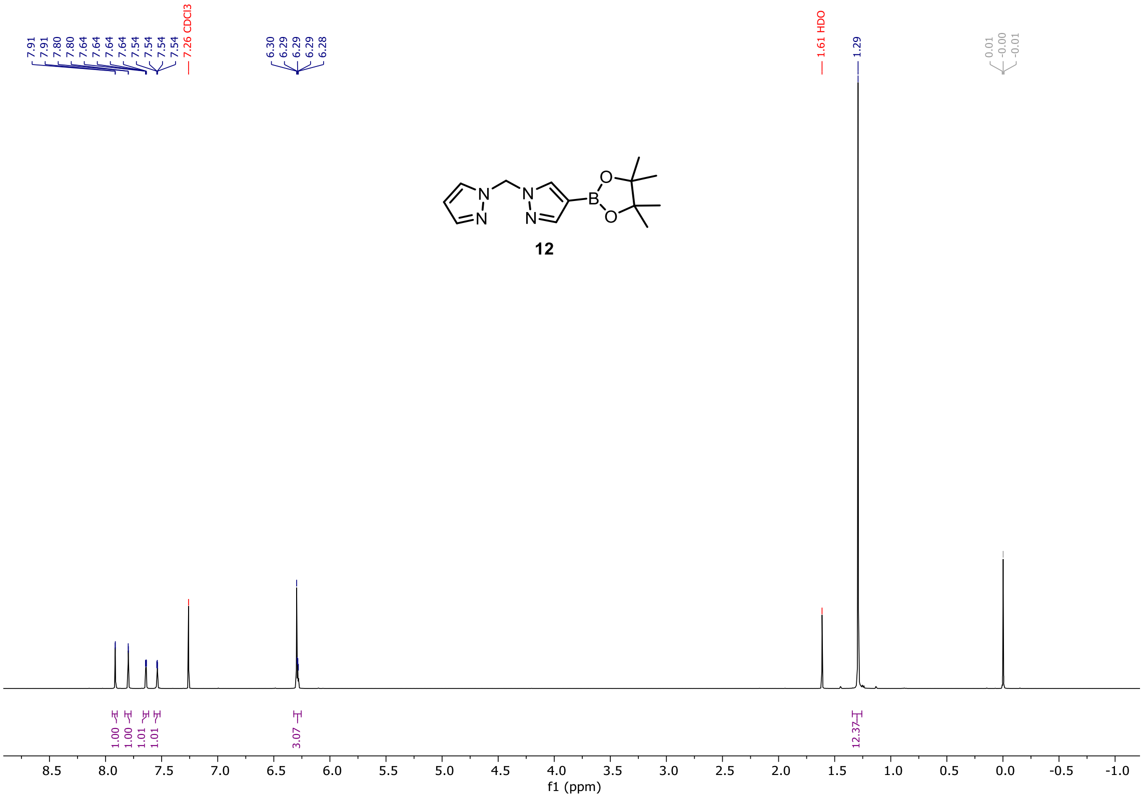

$^{13}C\{^{1}H\}$ (101 MHz, Methylene Chloride-d_2)

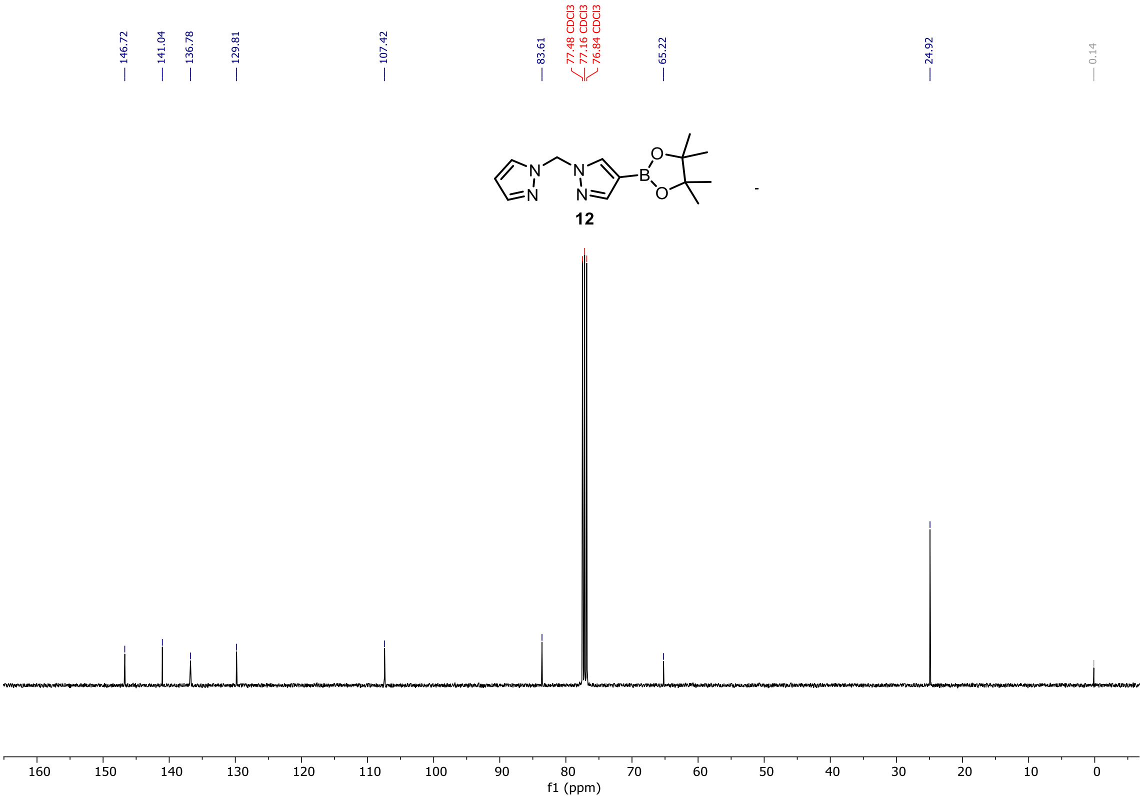

^{1}H (400 MHz, Chloroform-*d*)

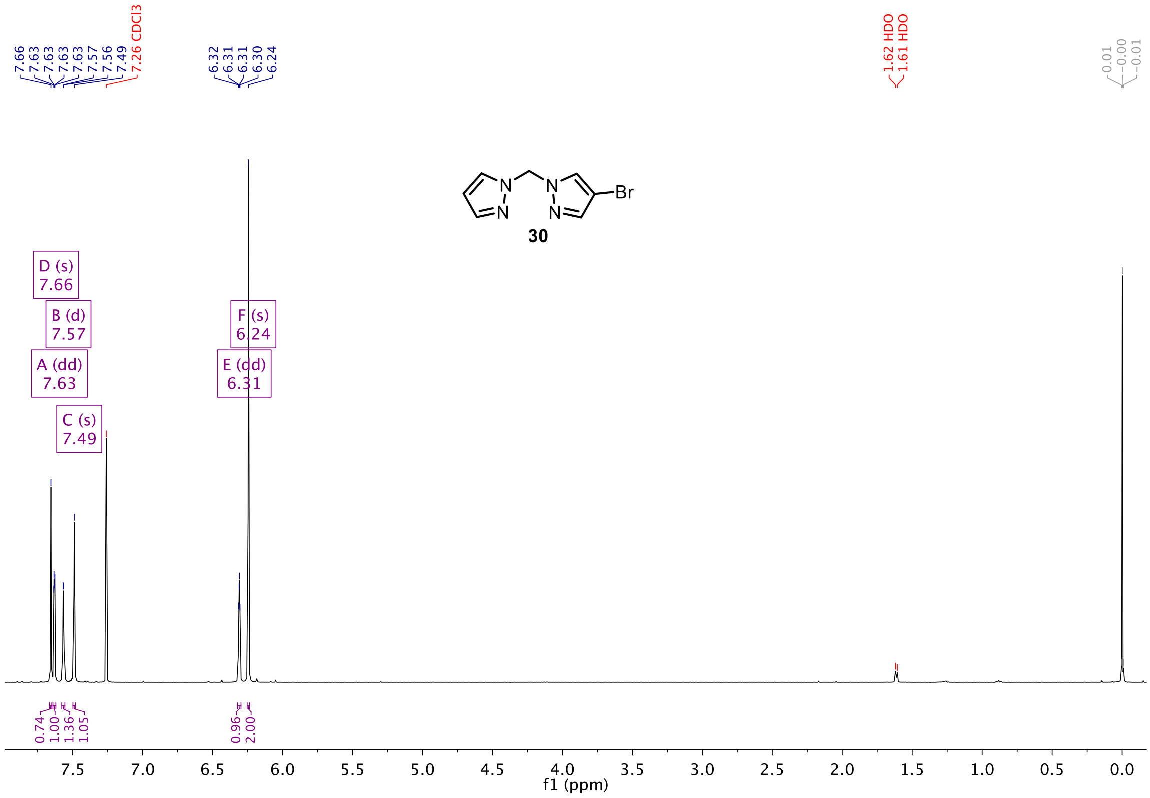

^{1}H (400 MHz, Chloroform-*d*)

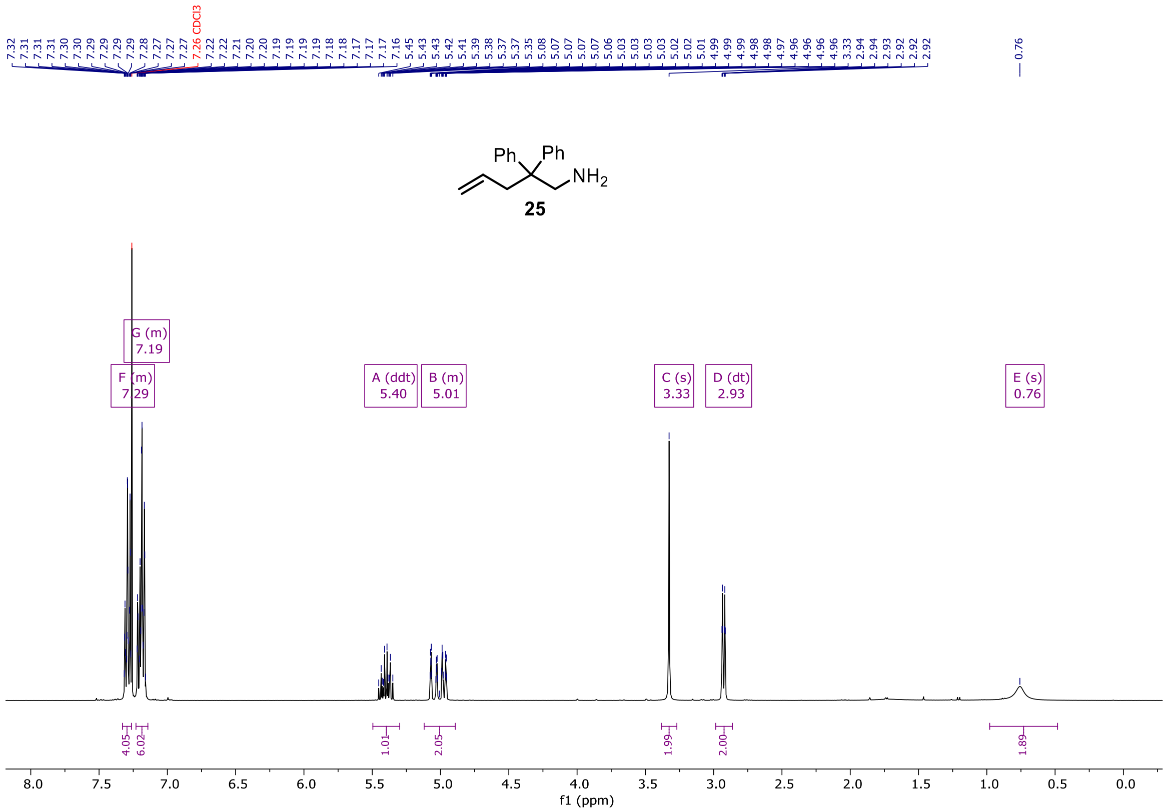

^{13}C{^{1}H} (101 MHz, Chloroform-*d*)

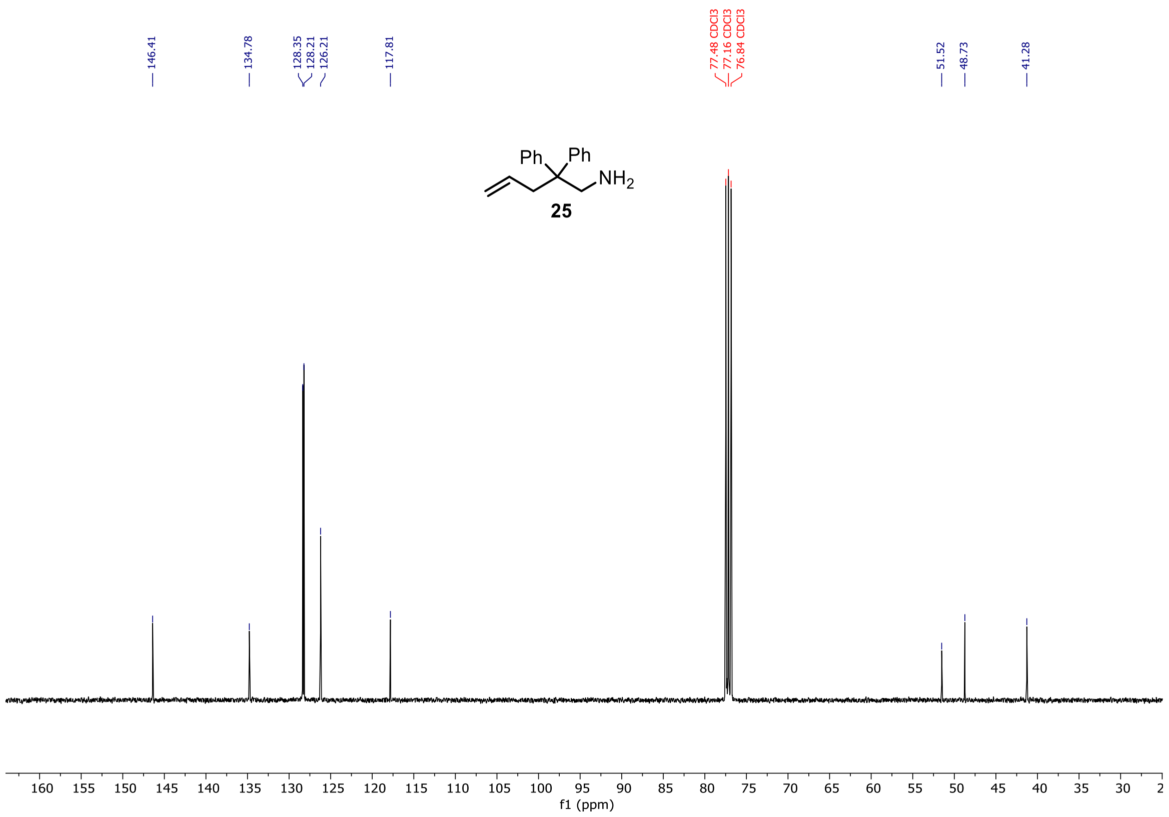

Products from the tandem reaction and switchable reaction

^{1}H (400 MHz, Chloroform-*d*)

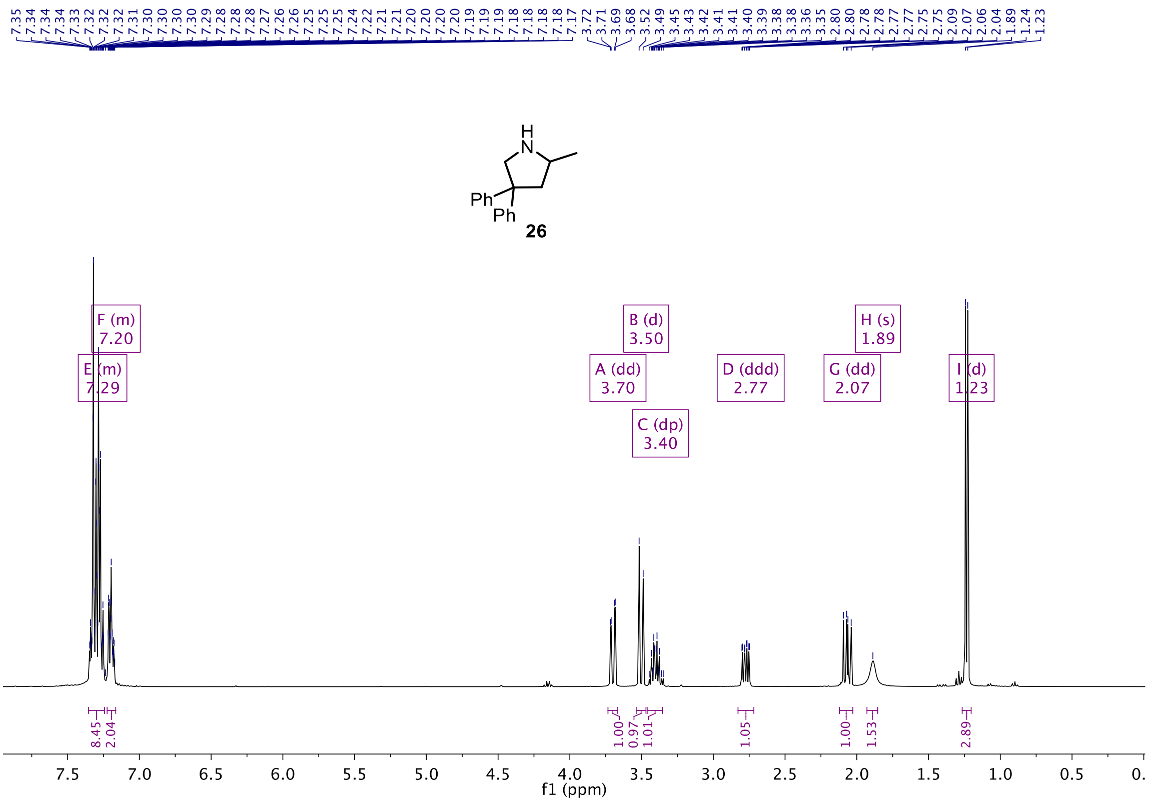

$^{13}C\{^{1}H\}$ (101 MHz, Chloroform-*d*)

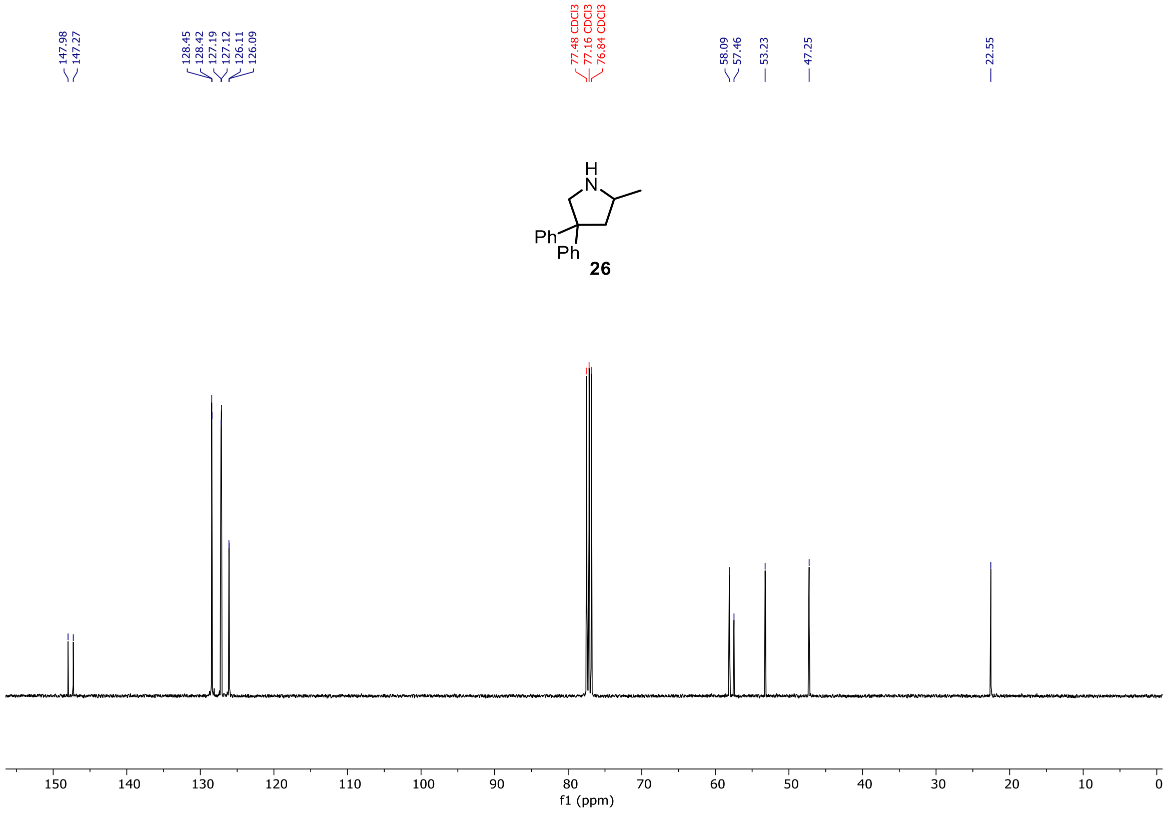

^{1}H (400 MHz, Chloroform-*d*)

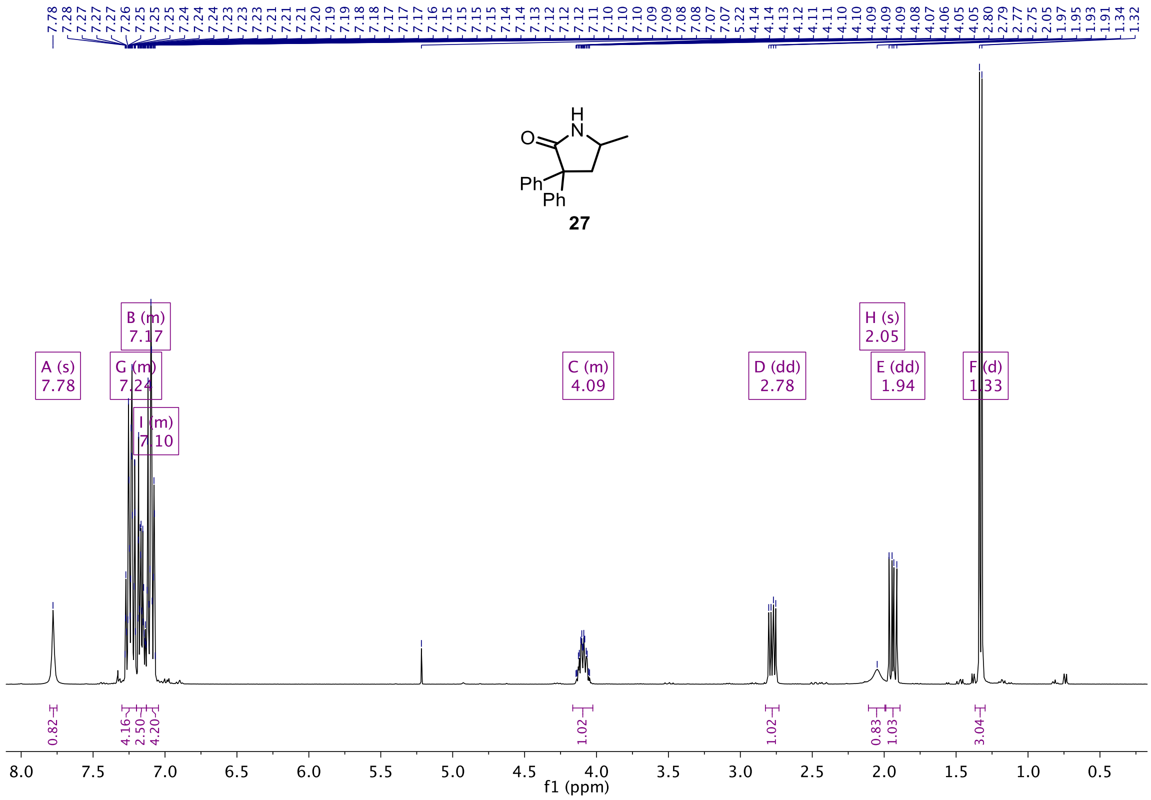

$^{13}C\{^{1}H\}$ (127 MHz, Chloroform-*d*)

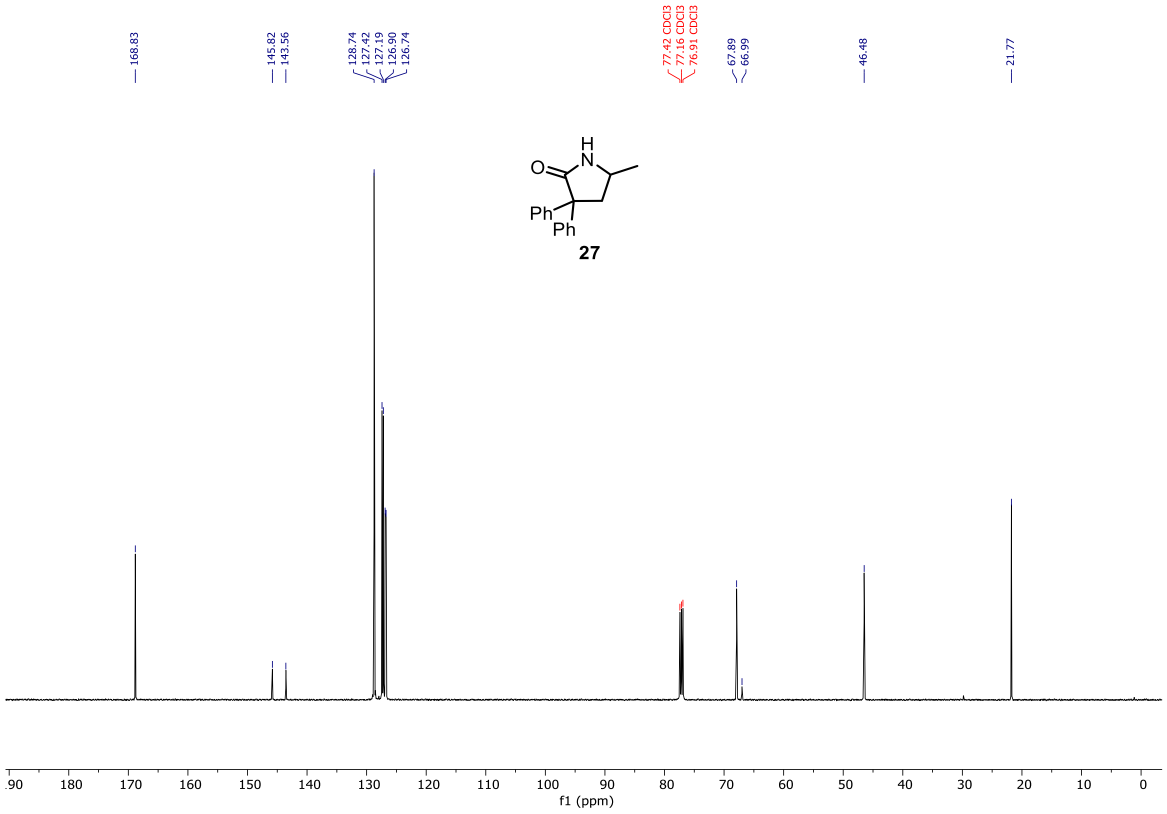

^{1}H (400 MHz, Chloroform-*d*)

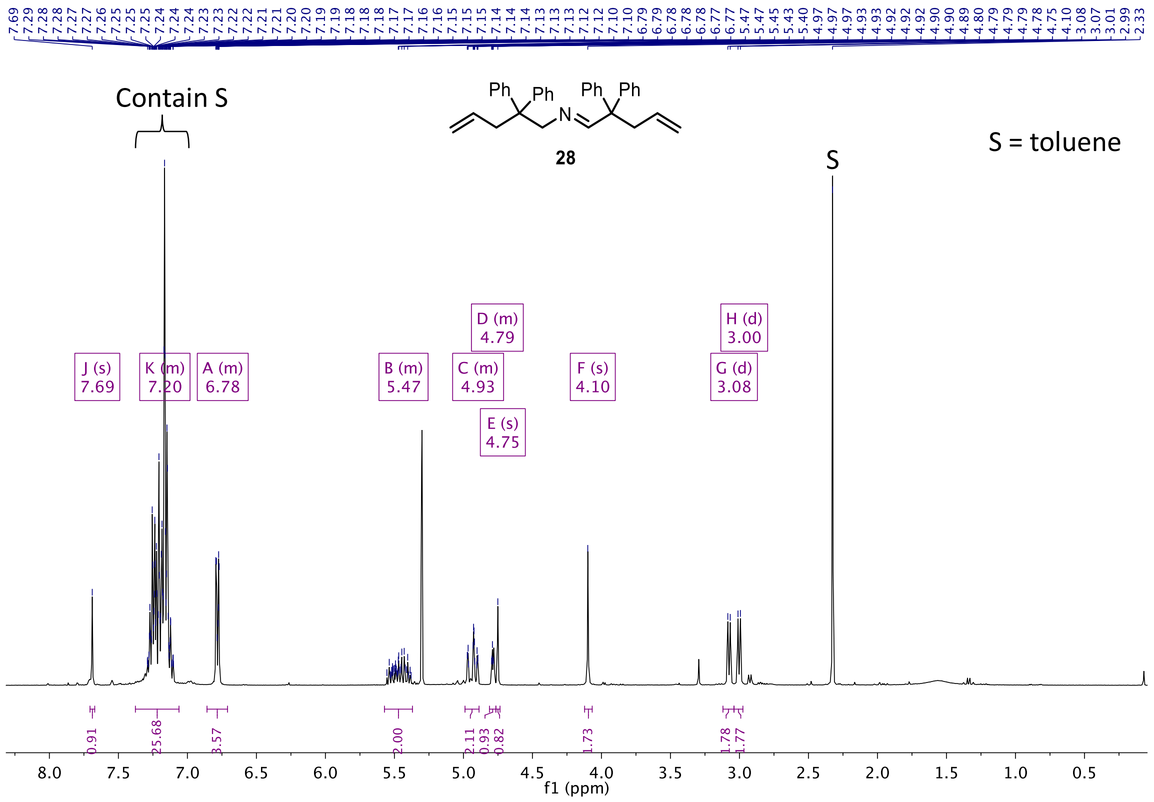

*Sample contains approximately 1.7 equivalents (based on ^{1}H NMR integration) of toluene.

$^{13}C\{^{1}H\}$ (127 MHz, Chloroform-*d*)

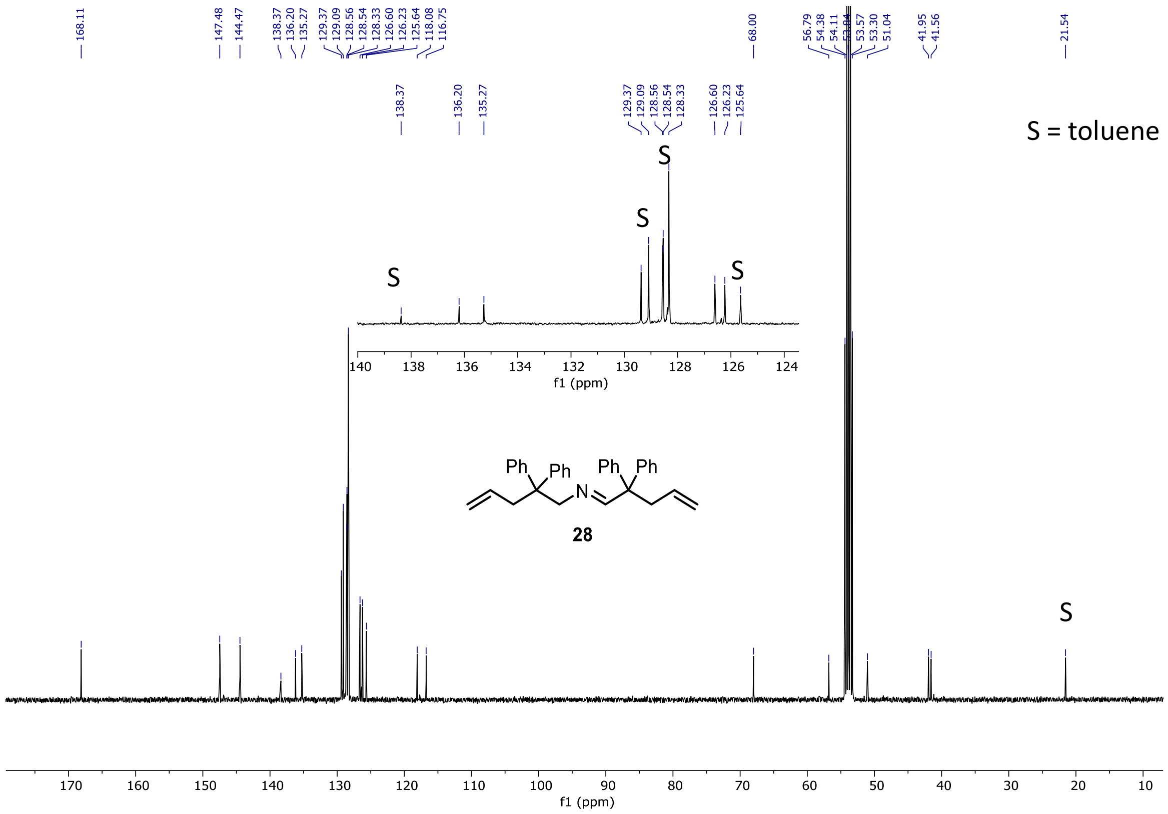

High Resolution Mass Spectra

Ir(I)-BDP SS **4**

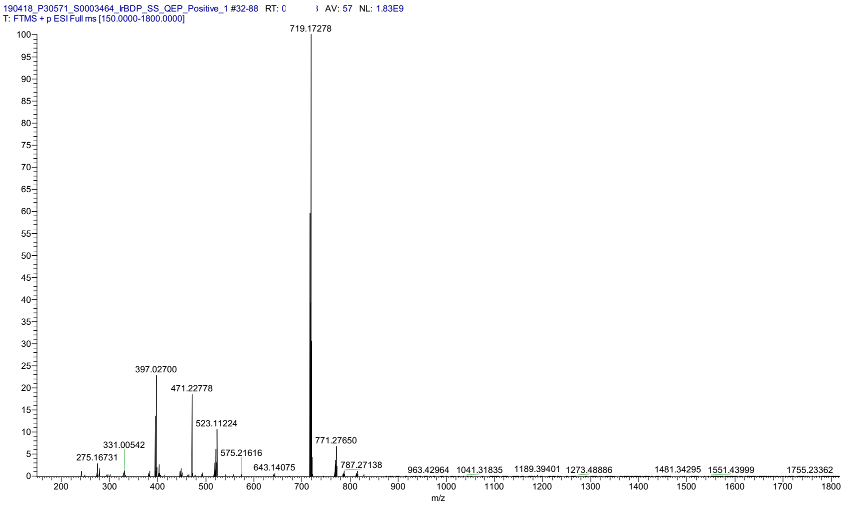

Ir(I)-BDP HH **5**

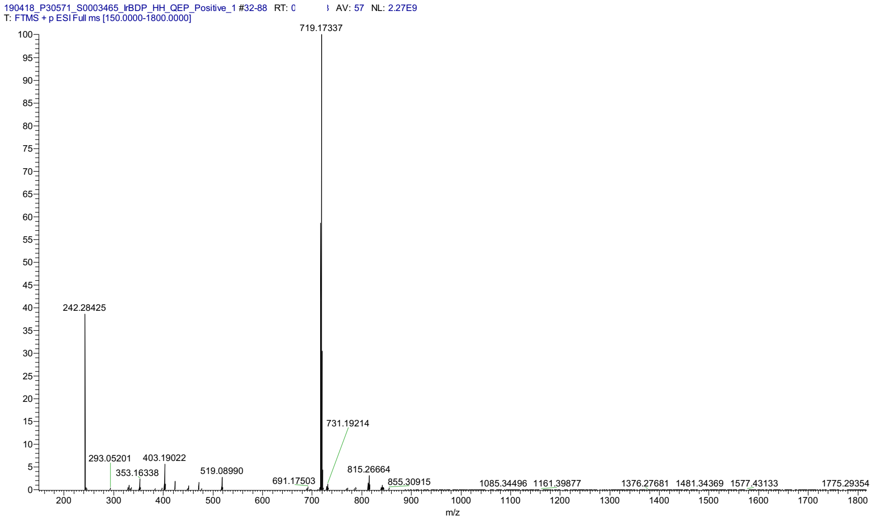

Ir(I)-BDP HS **6**

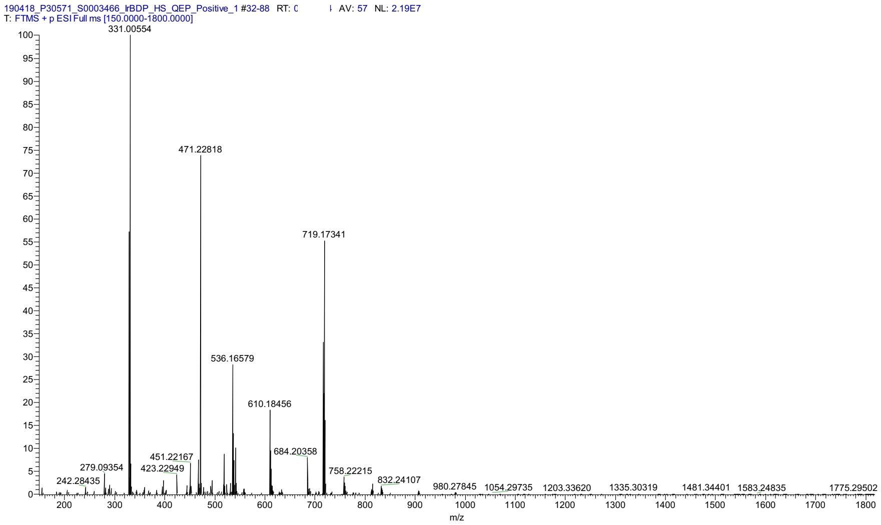

Ir(III)-BDP SS **7**

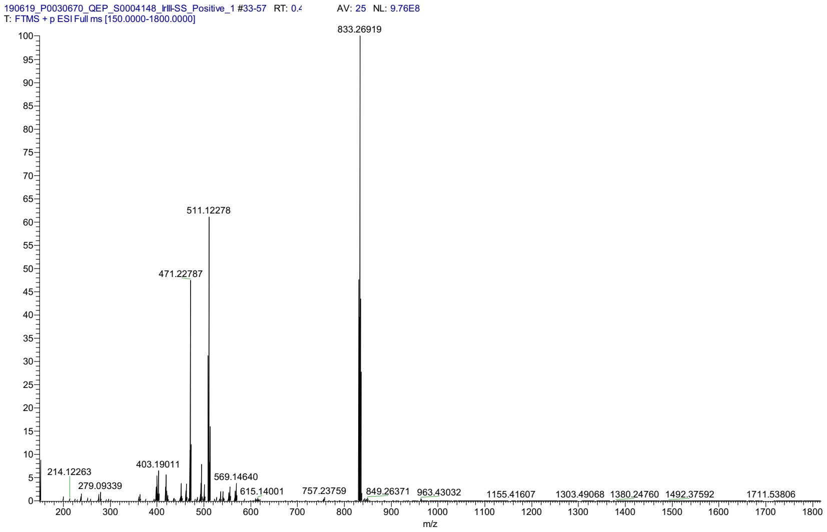

Ir(III)-BDP HH **8**

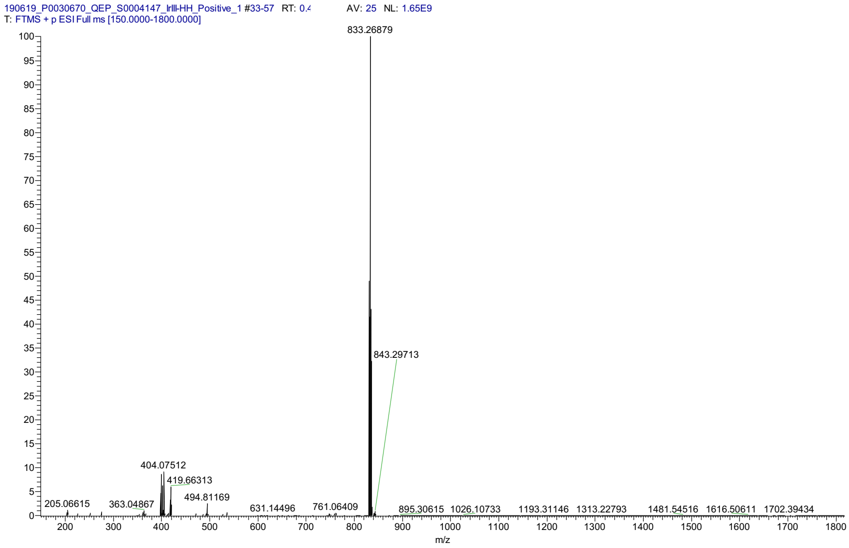

Ir(III)-BDP HS **9**

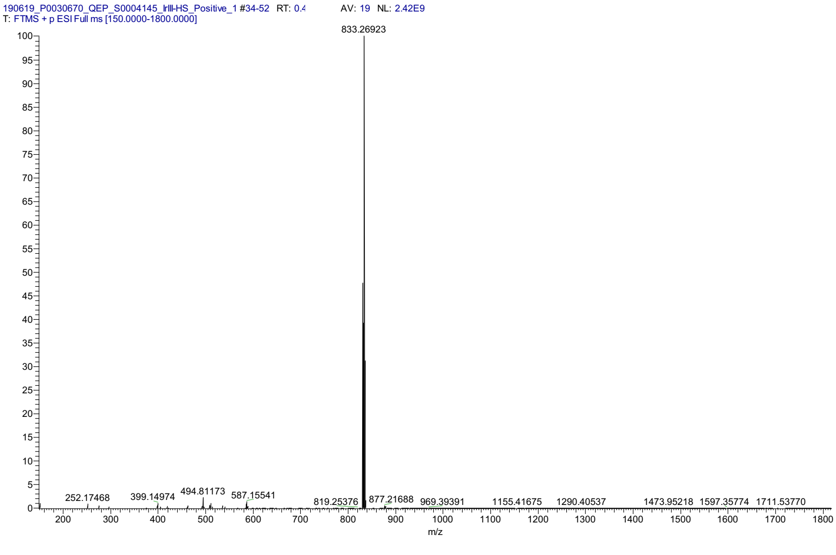

SS **13**

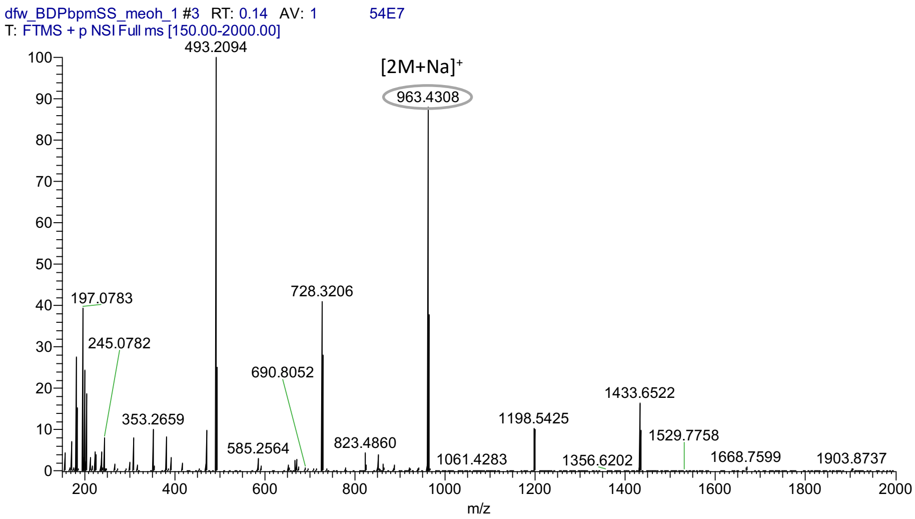

HS **14**

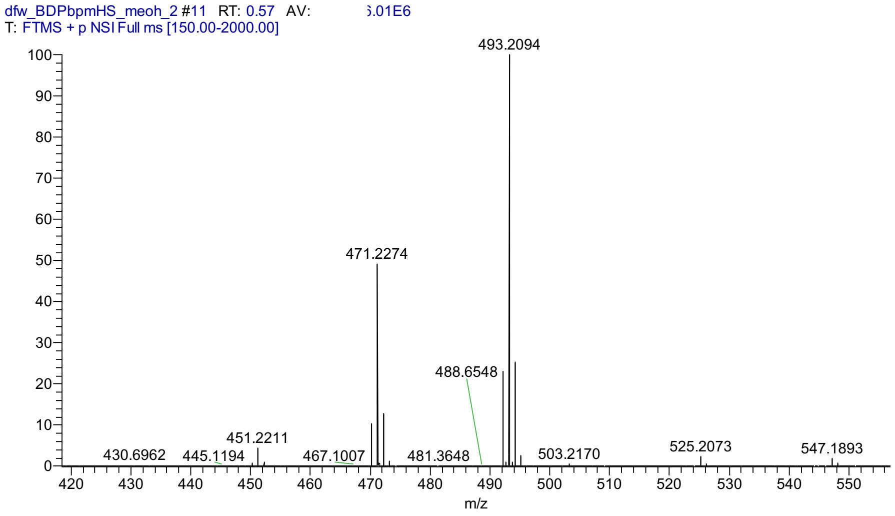

HH **17**

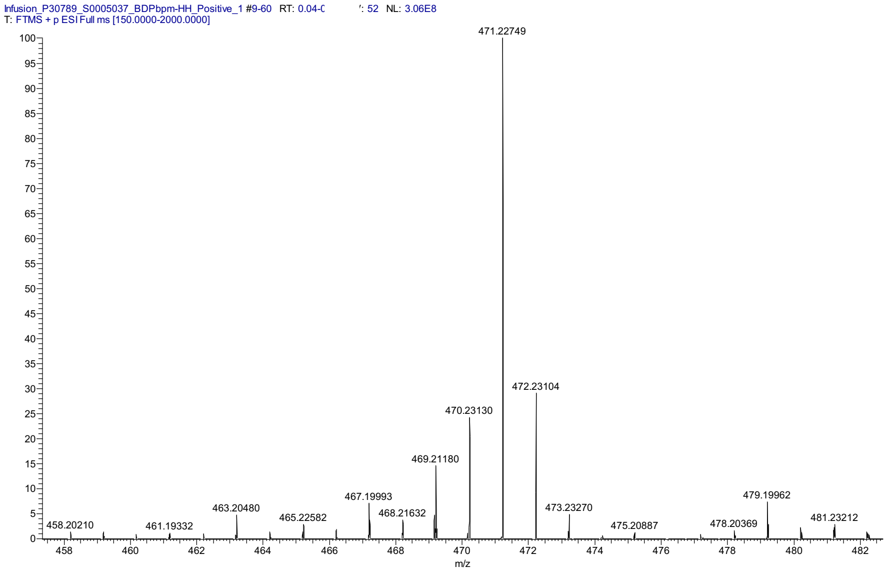

1-((1H-pyrazol-1-yl)methyl)-4-(4,4,5,5-tetramethyl-1,3,2-dioxaborolan-2-yl)-1H-pyrazole **12**

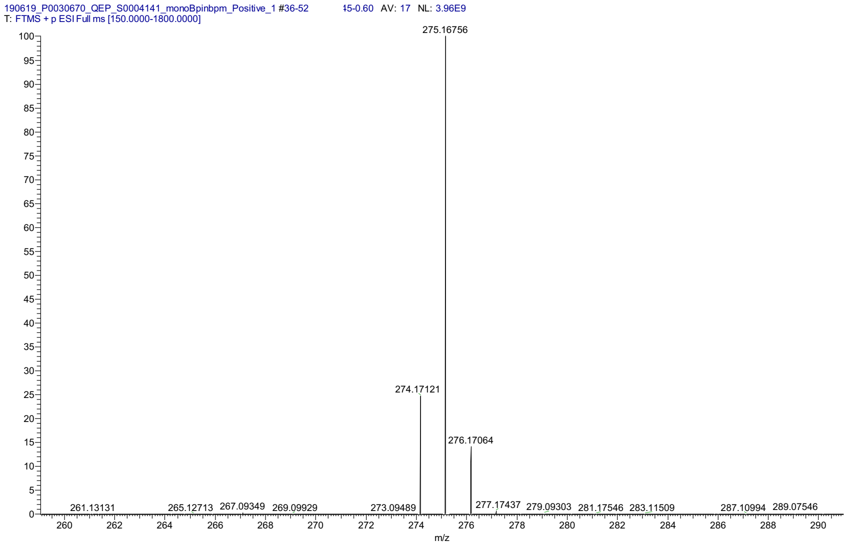

The switchable reaction product, *N*-(2,2-diphenylpent-4-en-1-ylidene)-2,2-diphenylpent-4-en-1-amine **28**

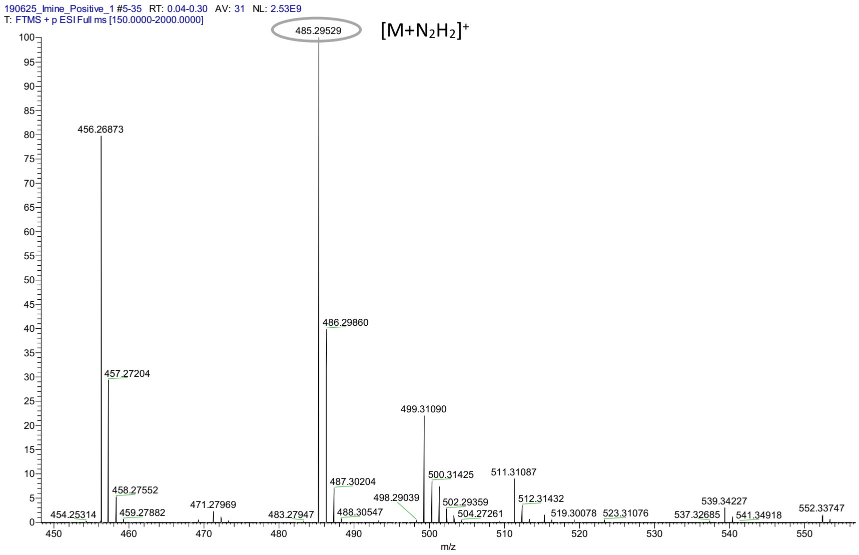

*The peak of interest is $[M+H]^+$ = 456.26873, a $[M+30]^+$ peak (485.29529) was also observed, which we postulate is due to $[M+N_2H_2]^+$. Mechanistically, the singlet oxygen promoted photooxidation involves the formation of H_2O_2 and the release of NH_3, and it is possible for NH_3 to be further oxidised to N_2H_2 under the photocatalytic conditions.[19]

1-((1H-pyrazol-1-yl)methyl)-4-bromo-1H-pyrazole **30**

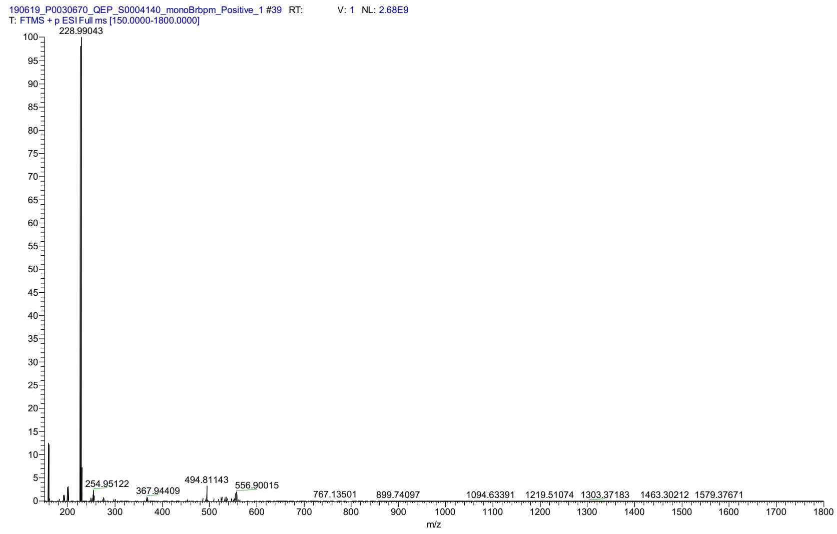

Elemental Microanalysis Reports

MACQUARIE University SYDNEY·AUSTRALIA
CHEMICAL ANALYSIS FACILITY

Test Report

Report No: 20022019

Attn: Danfeng Wang
Molecular Sciences

Phone: 8268
Email: danfeng.wang@hdr.mq.edu.au

From: Dr Remi Rouquette
Chemical Analysis Facility
Department of Molecular Sciences
Macquarie University, NSW, 2109

Phone: +61 2 9850 4219
Email: remi.rouquette@mq.edu.au

20th February, 2019

Elemental Microanalysis Report

Sample ID: Ir(III)-BDP HH **8**
Date Received: 14th February, 2019
Date Analysed: 19th February, 2019

Analysis Results:

Sample ID	Weight (mg)	N%	C%	H%
(IrBDPbpmCp*Cl)BBrF4-HH	1.033	5.16	48.58	2.356
	0.926	5.17	48.36	2.331
Theoritical values	-	4.95	48.14	3.09

Comments:

- Sample was stored at room temperature before analysis.
- Values are expressed as grams of element per 100 grams of sample. For organic standard materials the trueness 95% confidence limit of the technique is ± 0.3% with a precision of ± 0.2%. Please note that the presence of water or other solvents, filter paper fibres or other foreign substances in the sample will result in appreciable deviations from the expected theoretical values.

Dr. Remi Rouquette
Senior Analytical Technician
Chemical Analysis Facility
Department of Molecular Sciences
Macquarie University

This report shall not be reproduced, except in its entirety, without the written approval of The Chemical Analysis Facility, Macquarie University (CAFMQ). The results are valid only for the samples tested and do not apply to the bulk unless sampling was performed by the CAFMQ.

MACQUARIE University SYDNEY·AUSTRALIA

CHEMICAL ANALYSIS FACILITY

Test Report

Report No: 23082019

Attn: Danfeng Wang
Molecular Sciences

Phone: 8268
Email: danfeng.wang@hdr.mq.edu.au

From: Dr Remi Rouquette
Chemical Analysis Facility
Department of Molecular Sciences
Macquarie University, NSW, 2109

Phone: +61 2 9850 4219
Email: remi.rouquette@mq.edu.au

23rd August, 2019

Elemental Microanalysis Report

Sample ID: Ir(I)-BDP SS **4**
Date Received: 21st August, 2019
Date Analysed: 22nd August, 2019

Analysis Results:

Sample ID	Weight (mg)	N%	C%	H%
Ir1-ss	1.575	5.32	44.51	2.204
	1.71	5.23	44.90	2.104
Theoritical values	-	5.31	45.56	2.36

Sample ID: Ir(I)-BDP HH **5**
Date Received: 21st August, 2019
Date Analysed: 22nd August, 2019

Analysis Results:

Sample ID	Weight (mg)	N%	C%	H%
Ir1-HH	0.842	4.82	44.95	2.858
	0.681	4.91	45.03	2.655
Theoritical values	-	5.31	45.56	2.36

This report shall not be reproduced, except in its entirety, without the written approval of The Chemical Analysis Facility, Macquarie University (CAFMQ). The results are valid only for the samples tested and do not apply to the bulk unless sampling was performed by the CAFMQ.

Sample ID: Ir(I)-BDP HS **6**
Date Received: 21st August, 2019
Date Analysed: 22nd August, 2019

Analysis Results:

Sample ID	Weight (mg)	N%	C%	H%
Ir1-HS	1.238	5.00	45.25	2.108
	1.659	5.12	46.04	2.127
Theoritical values	-	5.31	45.56	2.36

Sample ID: Ir(III)-BDP HS **9**
Date Received: 21st August, 2019
Date Analysed: 22nd August, 2019

Analysis Results:

Sample ID	Weight (mg)	N%	C%	H%
Ir3-HS	1.406	4.83	47.99	2.750
	1.662	4.84	48.19	2.729
Theoritical values	-	4.95	48.14	3.09

Comments:

- Sample was stored at room temperature before analysis.
- Values are expressed as grams of element per 100 grams of sample. For organic standard materials the trueness 95% confidence limit of the technique is ± 0.3% with a precision of ± 0.2%. Please note that the presence of water or other solvents, filter paper fibres or other foreign substances in the sample will result in appreciable deviations from the expected theoretical values.

Dr. Remi Rouquette
Senior Analytical Technician
Chemical Analysis Facility
Department of Molecular Sciences
Macquarie University

This report shall not be reproduced, except in its entirety, without the written approval of The Chemical Analysis Facility, Macquarie University (CAFMQ). The results are valid only for the samples tested and do not apply to the bulk unless sampling was performed by the CAFMQ.

MACQUARIE University
SYDNEY·AUSTRALIA

CHEMICAL ANALYSIS FACILITY

Test Report

Report No: 24092019

Attn: Danfeng Wang
Molecular Sciences

Phone: 8268
Email: danfeng.wang@hdr.mq.edu.au

From: Dr Remi Rouquette
Chemical Analysis Facility
Department of Molecular Sciences
Macquarie University, NSW, 2109

Phone: +61 2 9850 4219
Email: remi.rouquette@mq.edu.au

24th September, 2019

Elemental Microanalysis Report

Sample ID: Ir(III)-BDP SS **7**
Date Received: 16th September, 2019
Date Analysed: 23rd September, 2019

Analysis Results:

Sample ID	Weight (mg)	N%	C%	H%
Ir3-ss	1.039	5.39	49.69	3.454
	0.857	5.19	47.35	3.159
Theoritical values	-	4.95	48.14	3.09

Comments:

- Sample was stored at room temperature before analysis.
- Values are expressed as grams of element per 100 grams of sample. For organic standard materials the trueness 95% confidence limit of the technique is ± 0.3% with a precision of ± 0.2%. Please note that the presence of water or other solvents, filter paper fibres or other foreign substances in the sample will result in appreciable deviations from the expected theoretical values.

Dr. Remi Rouquette
Senior Analytical Technician
Chemical Analysis Facility
Department of Molecular Sciences
Macquarie University

This report shall not be reproduced, except in its entirety, without the written approval of The Chemical Analysis Facility, Macquarie University (CAFMQ). The results are valid only for the samples tested and do not apply to the bulk unless sampling was performed by the CAFMQ.
